# Guanidine Derivatives: How Simple Structural Modification of Histamine H_3_R Antagonists Has Led to the Discovery of Potent Muscarinic M_2_R/M_4_R Antagonists

**DOI:** 10.1021/acschemneuro.1c00237

**Published:** 2021-06-08

**Authors:** Marek Staszewski, Dominik Nelic, Jakub Jończyk, Mariam Dubiel, Annika Frank, Holger Stark, Marek Bajda, Jan Jakubik, Krzysztof Walczyński

**Affiliations:** † Department of Synthesis and Technology of Drugs, Medical University of Lodz, ul. Muszyńskiego 1, 90-151 Łódź, Poland; ‡ Department of Neurochemistry, Institute of Physiology CAS, Videnska 1083, CZ142 20, Prague, Czech Republic; § Department of Physicochemical Drug Analysis, Faculty of Pharmacy, Jagiellonian University Medical College, Medyczna 9, 30-688 Kraków, Poland; ∥ Institute of Pharmaceutical and Medicinal Chemistry, Heinrich Heine University Düsseldorf, Universitaetsstr. 1, Duesseldorf 40225, Germany

**Keywords:** Antagonists, histamine H_3_ receptor, muscarinic M_2_ receptor, muscarinic M_4_ receptor, structure−activity relationships, guanidine derivatives

## Abstract

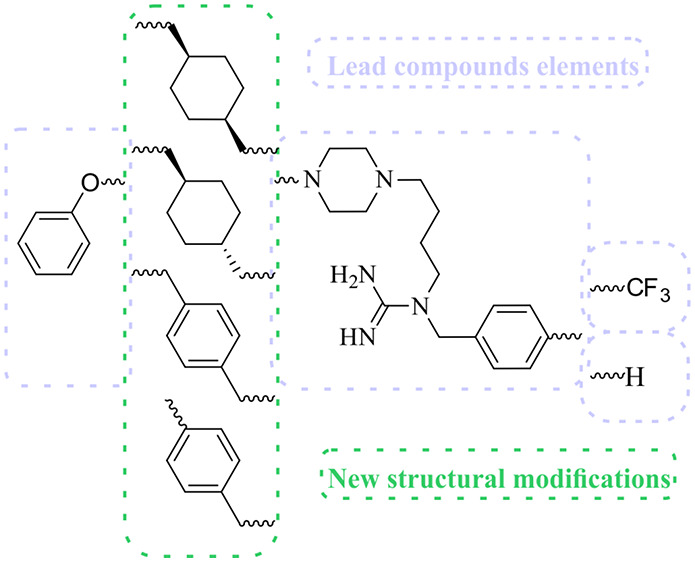

This article describes the discovery of novel potent muscarinic receptor antagonists identified during a search for more active histamine H_3_ receptor (H_3_R) ligands. The idea was to replace the flexible seven methylene linker with a semirigid 1,4-cyclohexylene or *p*-phenylene substituted group of the previously described histamine H_3_R antagonists **ADS1017** and **ADS1020**. These simple structural modifications of the histamine H_3_R antagonist led to the emergence of additional pharmacological effects, some of which unexpectedly showed strong antagonist potency at muscarinic receptors. This paper reports the routes of synthesis and pharmacological characterization of guanidine derivatives, a novel chemotype of muscarinic receptor antagonists binding to the human muscarinic M_2_ and M_4_ receptors (hM_2_R and hM_4_R, respectively) in nanomolar concentration ranges. The affinities of the newly synthesized **ADS10227** (1-{4-{4-{[4-(phenoxymethyl)cyclohexyl]methyl}piperazin-1-yl}but-1-yl}-1-(benzyl)guanidine) at hM_2_R and hM_4_R were 2.8 nM and 5.1 nM, respectively.

## Introduction

In addition to mediating the inhibition of synthesis and release of histamine from histaminergic neurons via a negative feedback loop, the histamine H_3_ receptor (H_3_R) also exerts modulatory effects on numerous other neurotransmitter systems, including the cholinergic system, in both the central and peripheral nervous system. Stimulation of H_3_ heteroreceptors in the central nervous system (CNS) by H_3_R agonists (imetit, immepip) diminishes acetylcholine (ACh) release; however, blocking H_3_ autoreceptors activity with the H_3_R antagonist (thioperamide) increases ACh release.^1^ Additionally, the ability of H_3_R antagonists to improve cognition and to increase release of ACh in rats was described.^2^ Activation of H_3_R in CNS reduces ACh release in the rat cortex, hippocampus, nucleus accumbens, and basolateral amygdala.^1,3−6^ In addition, the histaminergic neurons in the ventral striatum modulate the activity of neighboring cholinergic neurons. Histamine released from histaminergic nerve terminals inhibits dopamine release, which decreases γ-aminobutyric acid release, and, in turn, increases the release of acetylcholine. Disturbances in the CNS cholinergic system have been implicated in the pathophysiology of Alzheimer’s and Parkinson’s disease, schizophrenia, depression, or epilepsy.^7−10^ H_3_R treatment has also been found to modulate cholinergic transmission in the peripheral nervous system.^11^ H_3_R activation reduces the release of [^3^H]-ACh induced by electrical stimulation in the longitudinal smooth muscle/myenteric plexus preparations.^12,13^

**Chart 1:**
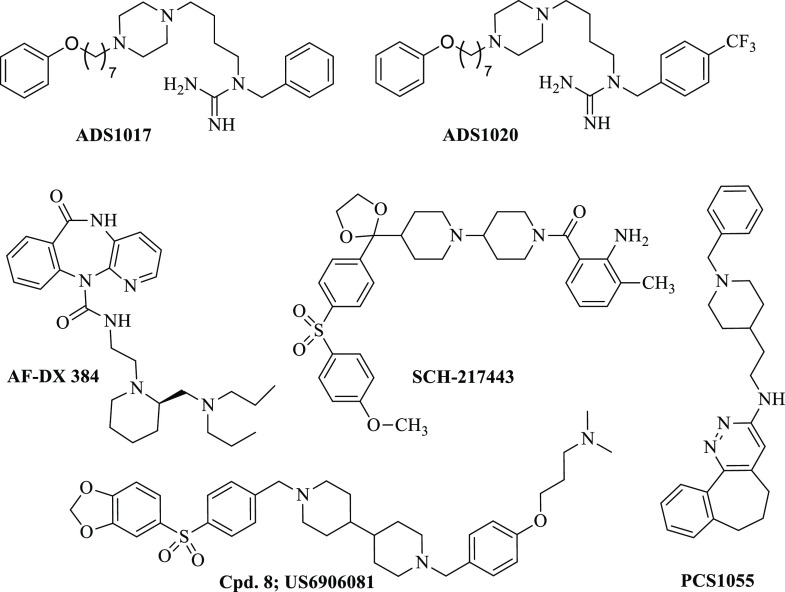
Structures of Histamine H_3_R (**ADS1017**, **ADS1020**), Muscarinic M_2_R (**SCH-217443**), Muscarinic M_4_R (**PCS 1055**), Muscarinic M_2_R/M_4_R (**AF-DX 384**), and H_3_R/M_2_R (**8**) Antagonists

Muscarinic M_2_ and M_4_ receptor (M_2_R and M_4_R, respectively) antagonists represent compounds of interest for potential drugs. Activation of M_2_R and M_4_R inhibits adenylyl cyclase via the stimulation of the G_i/o_ G-protein, whereas M_1_R, M_3_R, and M_5_R mediate the stimulation of phospholipase C via G_q/11_ G-protein.^14^ The cognitive deficits observed in aging and Alzheimer’s disease have been associated with brain cholinergic deficits. However, cognitive performance could be enhanced by selective blockade of presynaptically located M_2_ autoreceptors, which could increase ACh release into the synaptic cleft.^15^ One of the acetylcholinesterase (AChE) inhibitors approved for use across the full spectrum of these cognitive disorders is donepezil; however, numerous potent M_2_R antagonists including SCH-217443 (Chart 1) have shown efficacy in increasing ACh release and improving cognitive functions.^16^ The effective dose of SCH-217443 in the rodent cognition model was found to be 30-fold lower than that known to increase heart rate in rats.^16^ It is considered that ACh plays a crucial role in governing learning and memory processes.^17^ Therefore, AChE inhibitors constitute a major drug class in the treatment of dementia associated with Alzheimer’s disease, but provide only modest symptomatic benefit. Another approach to the therapy of Alzheimer’s disease is to inhibit the presynaptic activity of the muscarinic M_2_R expressed on the cholinergic neurons, in regions involved in learning and memory processes.^18,19^ As previously mentioned, the histamine H_3_R exerts modulatory effects on the cholinergic system, and H_3_R antagonist increases ACh release. Therefore, dual active H_3_R/M_2_R antagonists were invented and described.^2^ Those dual active ligands (Chart 1) relates to the treatment of Alzheimer’s disease, attention deficit disorder, and autism.

The muscarinic M_4_R are expressed in the striatum, prefrontal cortex, and nucleus accumbens; these areas are all related to social behaviors and cognitive functions.^20−22^ M_4_R agonists and allosteric modulators may be useful for the therapy of Alzheimer’s disease, schizophrenia, cognitive disorders, or treatment of drug abuse. Many M_4_R antagonists display limited selectivity between receptor subtypes. One recently described selective and potent M_4_R competitive antagonist is PCS1055 (Chart 1).^23^ M_4_R antagonists may produce psychotic-like symptoms (ADHD, hallucinations); nevertheless, they may be useful pharmacological tools in elucidating the M_4_R signaling mechanism. Experimental data suggest that M_4_R autoreceptors located in cholinergic interneurons may be useful in increasing ACh release in the striatum. This approach may be a promising alternative therapeutic method in Parkinson’s disease therapy.^24^

In contrast to the variety apparent in ligands, the binding sites of the H_3_R and muscarinic receptors share many common features. Studies suggest that H_3_R had a common ancestor with the muscarinic receptors.^25^ A detailed comparison of the active site sequences is summarized in the Supporting Information (Table S1).

**Figure 1:**
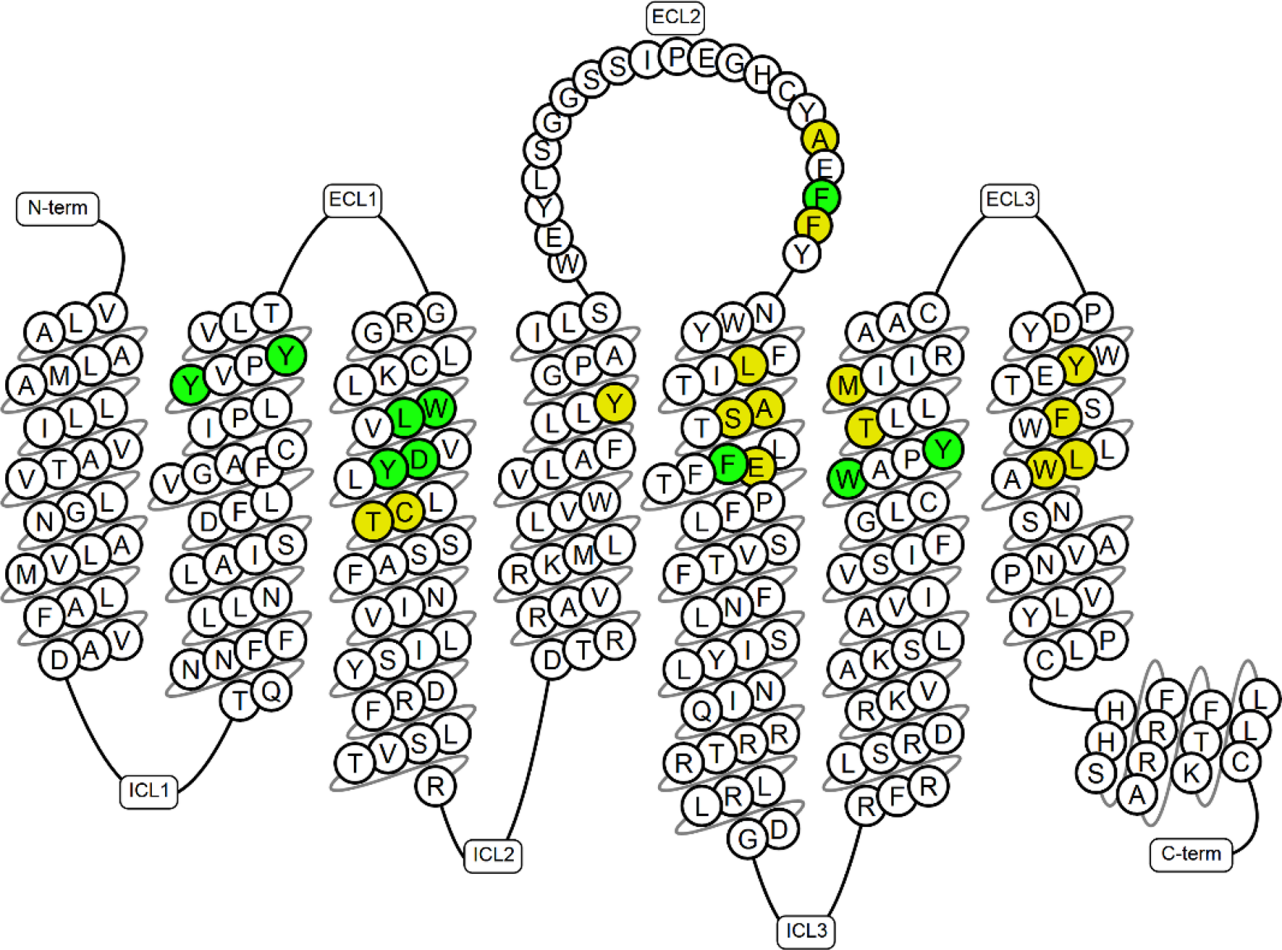
The amino acid sequence of the H_3_R, specifying the amino acids involved in the binding of the ligands. Amino acids identical to the M_2_R and M_4_R are marked in green and differing in yellow.^26^

An analysis of the complexes formed between the hM_2_R and hM_4_R and their antagonists identifies 25 amino acids of key importance for ligand binding. As shown in Figure 1, 10 of these (40%) are identical to those of the hH_3_R.^26^ Most of the remaining amino acids retain the physicochemical properties of their histamine receptor analogs. It should be emphasized that the number and distribution of aromatic amino acids significantly modify the ligand available space at the binding site. In the case of the studied receptors, the conformation of aromatic amino acids 2.61, 2.64, 3.33, 6.51, 6.48, and 7.39 seems to be of particular importance. Earlier studies also indicate that phenylalanines F192, F193 located at the end of H_3_R ECL2 and their analogs from M_2_R (F180, F181) and M_4_R (F189, L190) are involved in the binding of ligands.^27^ Another significant similarity between the analyzed receptors is the presence of an extensive amino acid at position 7.42 (H_3_R L7.42, M_2_R and M_4_R C7.42), which can modify the arrangement of W6.48 in the inactive state of the receptor.^28^

The differences between the two pairs of amino acids seem particularly important due to their participation in the binding of endogenous ligands. The first is the glutamic acid present in the H_3_R, E5.46, which is replaced by alanine in all muscarinic receptors. Mutagenesis studies carried out on the histamine H_3_R within this site indicate that such a replacement significantly weakens or completely prevents the effective binding of both agonists and antagonists. The second pair is the T6.52 from the H_3_R which replaces the N6.52 present in all muscarinic receptor subtypes. This limits the number of potential hydrogen bonds formed at this site while reducing the polarity of the surroundings. It is worth noting that mutagenesis studies indicate that the presence of asparagine at this position is essential for ligand binding at the muscarinic receptors binding site.^29,30^ Such differences merit particular consideration when designing new ligands for both histamine H_3_R and M_2_R and M_4_R; slight structural changes may significantly determine the activity profile of the new compounds.

## Results and Discussion

**Chart 2:**
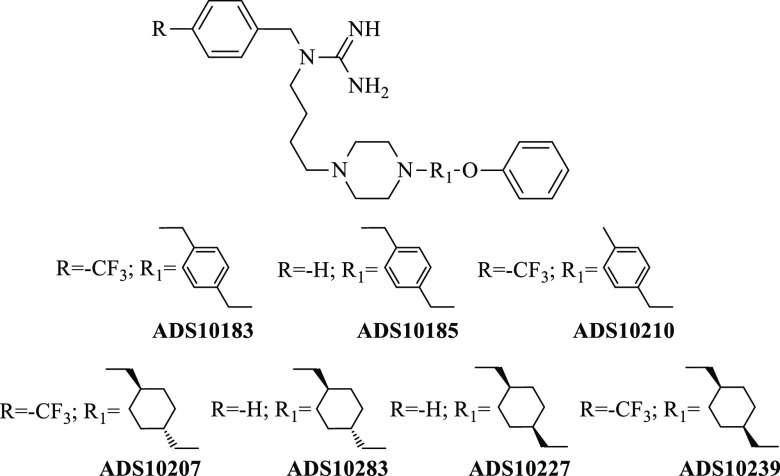
Target Molecules of This Study

This article describes the discovery of novel potent muscarinic receptor antagonists identified during a search for more active H_3_R ligands. Our previous study clarified whether both nitrogen atoms of the piperazine ring are necessary to maintain a high activity in the H_3_R antagonists, **ADS1017** and **ADS1020** (Chart 1), and determined the influence of moving the benzyl- and 4-trifluoromethylbenzyl substituents from the *N*^*1*^ to the *N*^*3*^ position of the guanidine.^31^ Finally, two symmetrical compounds, 1,4-bis{4-[1-(4-trifluoromethylbenzyl)guandin-1-yl]but-1-yl}piperazine (**ADS1030**) and 1,4-bis(7-phenoxyheptyl)piperazine (**ADS1031**), were synthesized to identify the part of the parent compound that plays a key role in blocking H_3_R. The most potent derivatives we found had piperazine as a central core with disubstitution to *N*^*1*^ of guanidine. Compounds based on 1-[4-(piperazin-1-yl)but-1-yl]guanidine proved to be key to maintaining a high affinity at the histamine H_3_R. Based on previously obtained data of the guanidine series, two representative of the lead compounds, that is, **ADS1017** and **ADS1020**, were selected for further structural optimization.^31,32^ In this paper, we have focused on the synthesis and pharmacological evaluation of a guanidine series where a flexible alkyl chain consisting of seven methylene groups, present in the lead compounds, is replaced by 1,4-cyclohexylene or *p*-phenylene group connected directly or by a methylene group to piperazine and phenoxy moieties. Additionally, for derivatives bearing a 1,4-disubstituted cyclohexylene group, the (*E*) and (*Z*) isomers were separated and pharmacologically tested independently (Chart 2).

The idea of synthesizing compounds in which the flexible alkyl chain was replaced by a semirigid aryl or cycloalkyl ring arose from our previous experiments and literature data.^33^ Previous studies have demonstrated that replacement of the alkyl chain with more rigid moieties such as aryl or heterocyclic rings results in the formation of highly affine H_3_R ligands.^34^ Furthermore, a moiety with a more restricted conformation may be better fitted to the receptor-binding site due to reduced flexibility (i.e., degrees of freedom).^28^ A key design element was to conserve the number of atoms between the phenoxy moiety and the basic nitrogen of piperazine and maintain an overall reduction in the number of rotatable bonds, thus producing more conformationally restricted compounds.

All newly synthesized compounds were evaluated as antagonists at H_3_R on guinea pig ileum (gpH_3_R), following which their selectivity to histamine H_1_R (gpH_1_R) and muscarinic M_2_R/M_3_R (gpM_2_R/M_3_R) was investigated. During these bioactivity profiling studies, the tested compounds demonstrated high affinities at muscarinic receptor subtypes. Therefore, all compounds were subjected to radioligand displacement assay in membrane fractions of HEK-293 cells stably expressing human H_3_R (hH_3_R). Finally, hM_1_–hM_5_ radioligand binding experiments were carried out for selected ligands. For two compounds, the intracellular Ca^2+^ level was measured as a functional response to ACh. Further investigation of the potent H_3_R antagonist **ADS1017** also revealed additional moderate affinity to hM_2_R and hM_4_R, which may justify searching for other dual-active ligands in the guanidine group. The present paper describes the discovery of the novel potent muscarinic receptor antagonist **ADS10227** (Chart 1), which demonstrates particular activity against the hM_2_R and hM_4_R. To understand the molecular basis of the unexpected muscarinic activity, *in silico* studies were also conducted.

### Chemistry

#### Synthesis of (*E*)-1,4-Bis(bromomethyl)cyclohexane (**3a**) and (*Z*)-1,4-Bis(bromomethyl)cyclohexane (**3b**)

To synthesize **3a** and **3b**, we started with a commercially available mixture of (*Z*)- and (*E*)-1,4-cyclohexanedimethanol. The NMR spectrum indicated that the ratio of (*Z*) and (*E*) isomers was 35:65%. The *E/Z* 1,4-cyclohexanedimethanol mixture was reacted with benzoyl chloride to give a mixture of (*E*)-1,4-cyclohexanedimethanol dibenzoate (**1a**) and (*Z*)-1,4-cyclohexanedimethanol dibenzoate (**1b**). The isomers were separated by multiple recrystallizations from ethyl acetate. Pure (>99% purity) isomer (*E*) was isolated as transparent plaques by two recrystallizations. Isolation of isomer (*Z*) was more complicated. The filtrate obtained after the first recrystallization was evaporated and recrystallized again. The crystals were discarded, and the filtrate containing 87% of (*Z*)-isomer was evaporated and recrystallized. The resulting crystals contained high-purity isomer (*Z*) (>99% purity). 1,4-Cyclohexanedimethanol **2a** (*E*-isomer) and **2b** (*Z*-isomer) were obtained from **1a** and **1b**, respectively, by de-esterification with sodium hydroxide. 1,4-Bis(bromomethyl)cyclohexane **3a** and **3b** were obtained by bromination of **2a** and **2b** with phosphorus tribromide.

#### Synthesis of Guanidines **ADS10207**, **ADS10239**, **ADS10183**, **ADS10210**, **ADS10283**, **ADS10227**, and **ADS10185**

Further synthetic procedures were similar for all newly synthesized compounds and analogous to the previously described routes of synthesis.^32^ Etherification of **3a** and **3b** and commercially available 1,4-bis(bromomethyl)benzene with sodium phenoxide in anhydrous ethanol led to **4a**, **4b**, and **11**. The 1-(substituted)piperazines **5a**, **5b**, **12**, and **18** were obtained from **4a**, **4b**, and **11**, commercially available 1-(bromomethyl)-4-phenoxybenzeneby alkylation with piperazine. N-alkylation of **5a**, **5b**, **12**, and **18** with 4-bromobutyronitrile in the presence of potassium carbonate in acetonitrile led to formation of 4-[4-(substituted)piperazin-1-yl]butanenitrile **6a**, **6b**, **13**, and **19**. The 4-[4-(substituted)piperazin-1-yl]butan-1-amines **7a**, **7b**, **14**, and **20** were reduced with LiAlH_4_ in dry diethyl ether. N-acylation with benzoyl chloride or 4-(trifluoromethyl)benzoyl chloride in the presence of triethylamine led to the synthesis of *N*-{4-[4-(substituted)piperazin-1-yl]butyl}benzamides **8a**, **8c**, and **15a** or *N*-{4-[4-(substituted)piperazin-1-yl]butyl}-4-(trifluoromethyl)benzamides **8b**, **8d**, **15b**, and **21**; subsequently reduced with LiAlH_4_ in dry diethyl ether to 4-[4-(substituted)piperazin-1-yl]-*N*-(benzyl)butan-1-amines **9a**, **9c**, and **16a** or 4-[4-(substituted)piperazin-1-yl]-*N*-[4-(trifluoromethyl)benzyl]butan-1-amines **9b**, **9d**, **16b**, and **22**. Guanylation with 1,3-bis(*tert*-butoxycarbonyl)-2-methylisothiourea in the presence of triethylamine and 10% excess of mercury(II) chloride resulted in 2,3-di(*tert*-butoxycarbonyl)-1-{4-[4-(substituted)piperazin-1-yl]but-1-yl}-1-(benzyl)guanidines **10a**, **10c**, and **17a** or 2,3-di(*tert*-butoxycarbonyl)-1-{4-[4-(substituted)piperazin-1-yl]but-1-yl}-1-[4-(trifluoromethyl)benzyl]guanidines **10b**, **10d**, **17b**, and **23**.

**Scheme 1:**
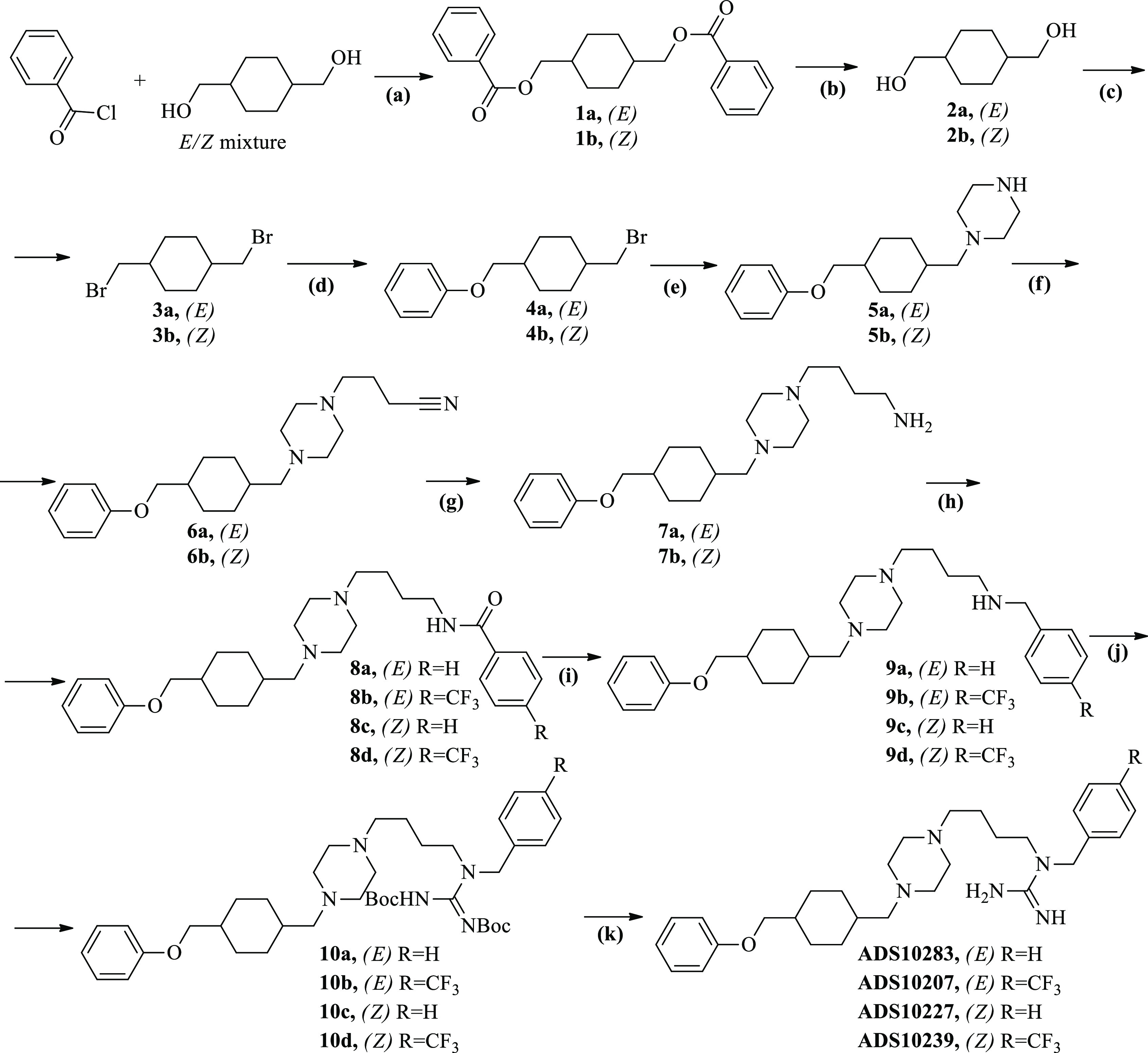
Synthesis of **ADS10283**, **ADS10207**, **ADS10227**, and **ADS10239** Reagents and conditions: (a) *E/Z* mixture 1,4-cyclohexanedimethanol (1.0 equiv), benzoyl chloride (2.0 equiv), triethylamine (2.0 equiv), DCM, 2.5 h, rt; (b) **1a**/**1b** (1.0 equiv), NaOH (10 equiv), H_2_O, MeOH, 24 h, 70 °C; (c) **2a**/**2b** (1 equiv), PBr_3_ (1.4 equiv), DMF, 90 min, 100 °C; (d) **3a**/**3b** (1 equiv), sodium phenoxide (1 equiv), EtOH, 24 h, 80 °C; (e) **4a**/**4b** (1 equiv), piperazine (5 equiv), THF, 24 h, reflux; (f) **5a**/**5b** (1 equiv), 4-bromobutyronitrile (1.3 equiv), potassium carbonate (5 equiv), MeCN, 24 h, 80 °C; (g) **6a**/**6b** (1 equiv), LiAlH_4_ (4 equiv), diethyl ether, 24 h, rt; (h) **7a**/**7b** (1 equiv), benzoyl chloride/4-(trifluoromethyl)benzoyl chloride (1.1 equiv), triethylamine (5 equiv), DCM, 3 h, rt (i) **8a**/**8b**/**8c**/**8d** (1 equiv), LiAlH_4_ (4 equiv), diethyl ether, 24 h, rt; (j) **9a**/**9b**/**9c**/**9d** (1 equiv), 1,3-bis(*tert*-butoxycarbonyl)-2-methylisothiourea (1.1 equiv), HgCl_2_ (1.1 equiv), triethylamine (5 equiv), DCM, 18 h, rt; (k) **10a**/**10b**/**10c**/**10d** (1 equiv), 4 M solution HCl-dioxan (20 equiv), CHCl_3_, 24 h, rt.

**Scheme 2:**
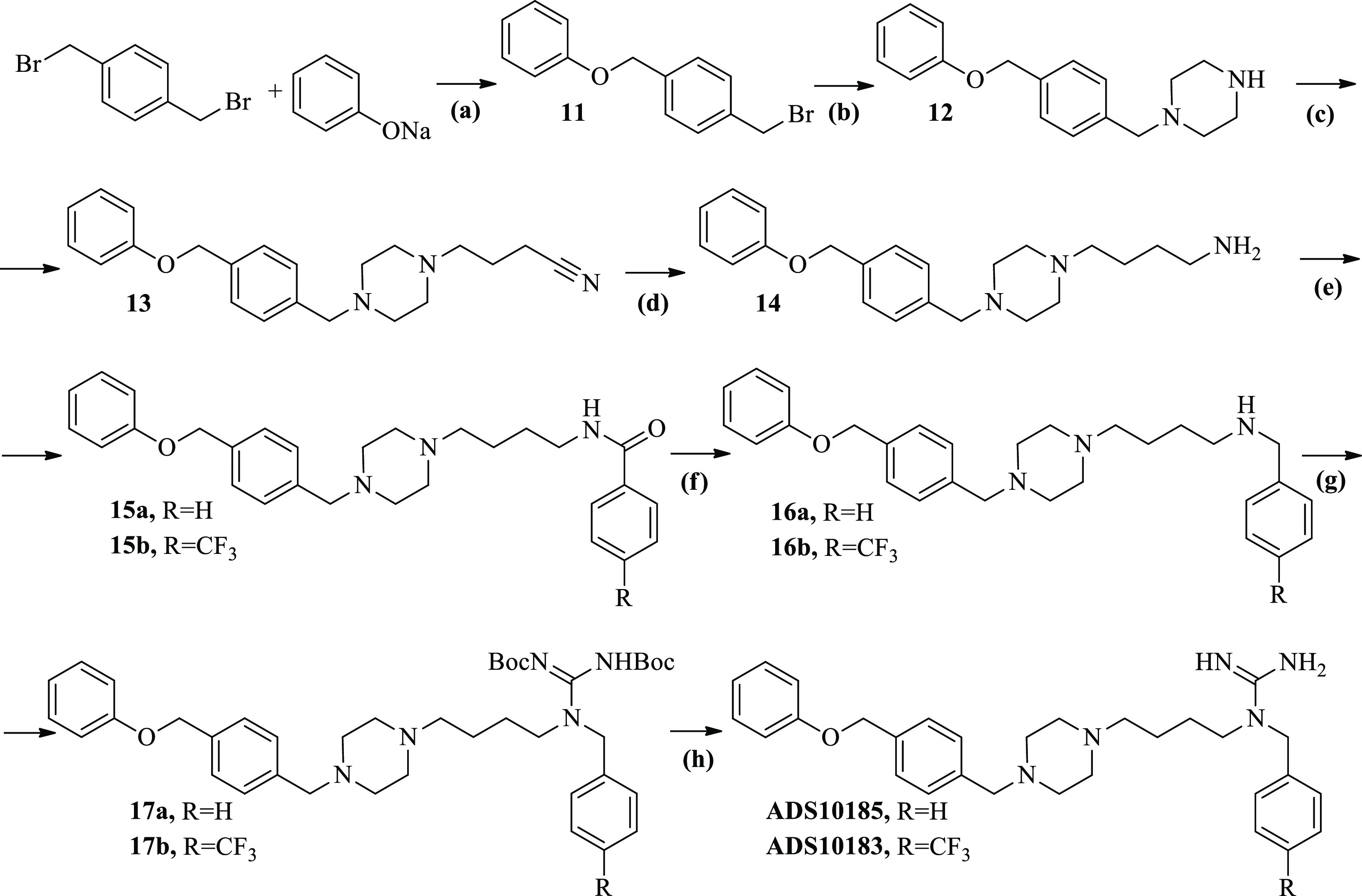
Synthesis of **ADS10183** and **ADS10185** Reagents and conditions: (a) 1,4-Bis(bromomethyl)benzene (1 equiv), sodium phenoxide (1 equiv), THF, 24 h, reflux; (b) **11** (1 equiv), piperazine (5 equiv), THF, 24 h, reflux; (c) **12** (1 equiv), 4-bromobutyronitrile (1.3 equiv), potassium carbonate (5 equiv), MeCN, 24 h, 80 °C; (d) **13** (1 equiv), LiAlH_4_ (4 equiv), diethyl ether, 24 h, rt; (e) **14** (1 equiv), benzoyl chloride/4-(trifluoromethyl)benzoyl chloride (1.1 equiv), triethylamine (5 equiv), DCM, 3 h, rt; (f) **15a**/**15b** (1 equiv), LiAlH_4_ (4 equiv), diethyl ether, 24 h, rt; (g) **16a**/**16b** (1 equiv), 1,3-bis(*tert*-butoxycarbonyl)-2-methylisothiourea (1.1 equiv), HgCl_2_ (1.1 equiv), triethylamine (5 equiv), DCM, 18 h, rt; (h) **17a**/**17b** (1 equiv), 4 M solution HCl-dioxan (20 equiv), CHCl_3_, 24 h, rt.

**Scheme 3:**
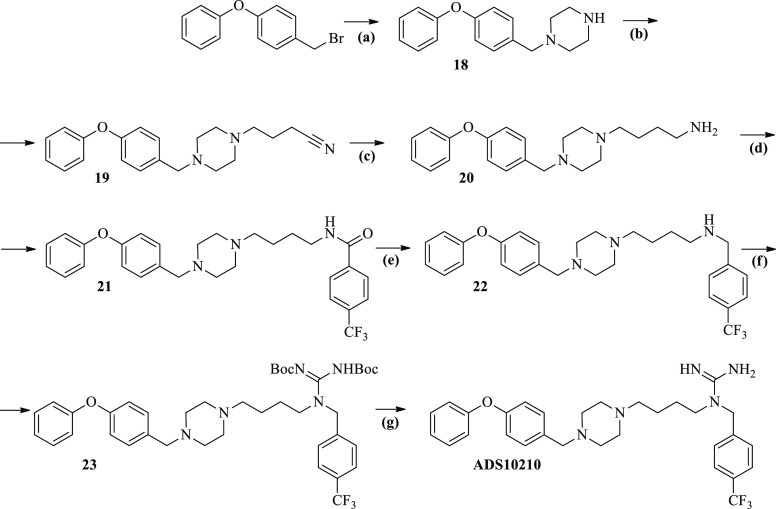
Synthesis of **ADS10210** Reagents and conditions: (a) 1-(Bromomethyl)-4-phenoxybenzene (1 equiv), piperazine (5 equiv), THF, 24 h, reflux; (b) **18** (1 equiv), 4-bromobutyronitrile (1.3 equiv), potassium carbonate (5 equiv), MeCN, 24 h, 80 °C; (c) **19** (1 equiv), LiAlH_4_ (4 equiv), diethyl ether, 24 h, rt; (d) **20** (1 equiv), 4-(trifluoromethyl)benzoyl chloride (1.1 equiv), triethylamine (5 equiv), DCM, 3 h, rt; (e) **21** (1 equiv), LiAlH_4_ (4 equiv), diethyl ether, 24 h, rt; (f) **22** (1 equiv), 1,3-bis(*tert*-butoxycarbonyl)-2-methylisothiourea (1.1 equiv), HgCl_2_ (1.1 equiv), triethylamine (5 equiv), DCM, 18 h, rt; (h) **23** (1 equiv), 4 M solution HCl-dioxan (20 equiv), CHCl_3_, 24 h, rt.

The final compounds were obtained by acidic deprotection of Boc groups from the guanidine moiety, resulting in 1-{4-[4-(substituted)piperazin-1-yl]but-1-yl}-1-[4-(trifluoromethyl)benzyl]guanidines **ADS10207**, **ADS10239**, **ADS10183**, and **ADS10210** or 1-{4-[4-(substituted)piperazin-1-yl]but-1-yl}-1-(benzyl)guanidines **ADS10283**, **ADS10227**, and **ADS10185.** An overview of the procedures is presented in the Methods section. For more details, see the Chemical synthesis and data analysis section in the Supporting Information. The structures and purity of the synthesized final products were confirmed by ^1^H NMR, ^13^C NMR spectra and elemental analysis (see Supporting Information). The synthesis of the **ADS10183**, **ADS10185**, **ADS10207**, **ADS10210**, **ADS10227**, **ADS10239**, and **ADS10283** compounds is depicted in Schemes 1, 2, and 3. **ADS1017** was synthesized according to Staszewski et al.^32^

### Pharmacology

Pharmacological results are assembled in Tables 1 and 2 including the previously described data for compound **ADS1017**.^32^ All graphs of the *ex vivo* assays including the inhibitory effect on the contraction of guinea pig ileum strips and hH_3_, hM_1_-hM_5_ radioligand binding assays are presented in the Supporting Information.

**Table 1:**
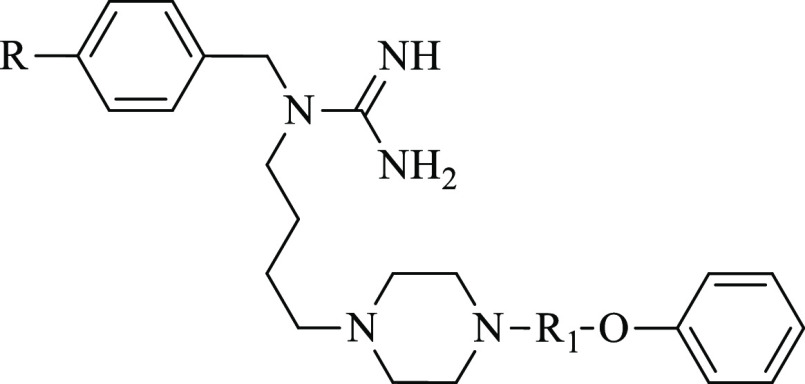

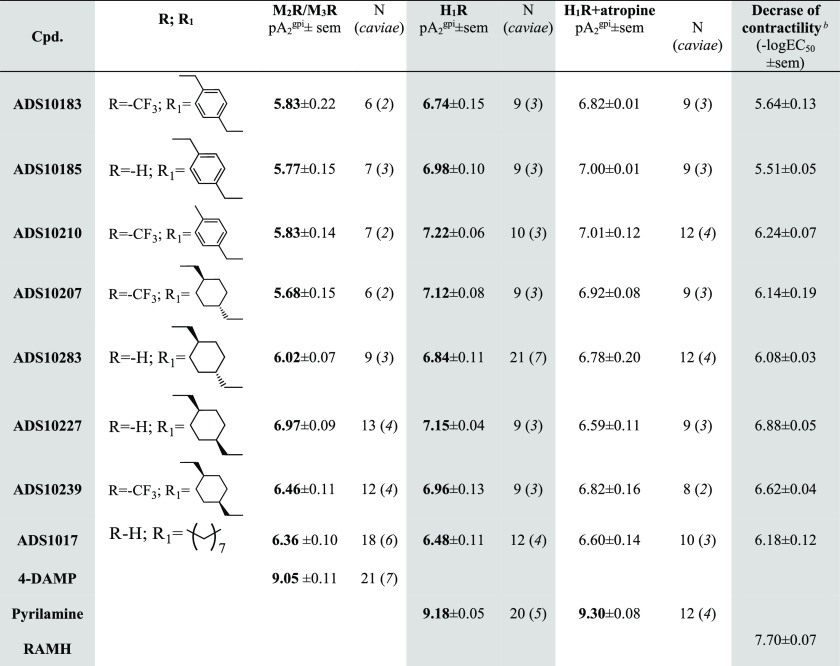
*Ex Vivo* Screening on the Isolated Guinea Pig Ileum^a^

a Values are means ± sem from at least three independent experiments; sem: standard error of the mean; *N*: number of different animal preparations; *caviae*: number of animals; gpi: guinea pig ileum; 4-DAMP: 1,1-dimethyl-4-diphenylacetoxypiperidinium iodide; RAMH: (*R*)(−)-α-methylhistamine.

b Decrease of contractility in electrically stimulated guinea pig ileum.

**Table 2:**
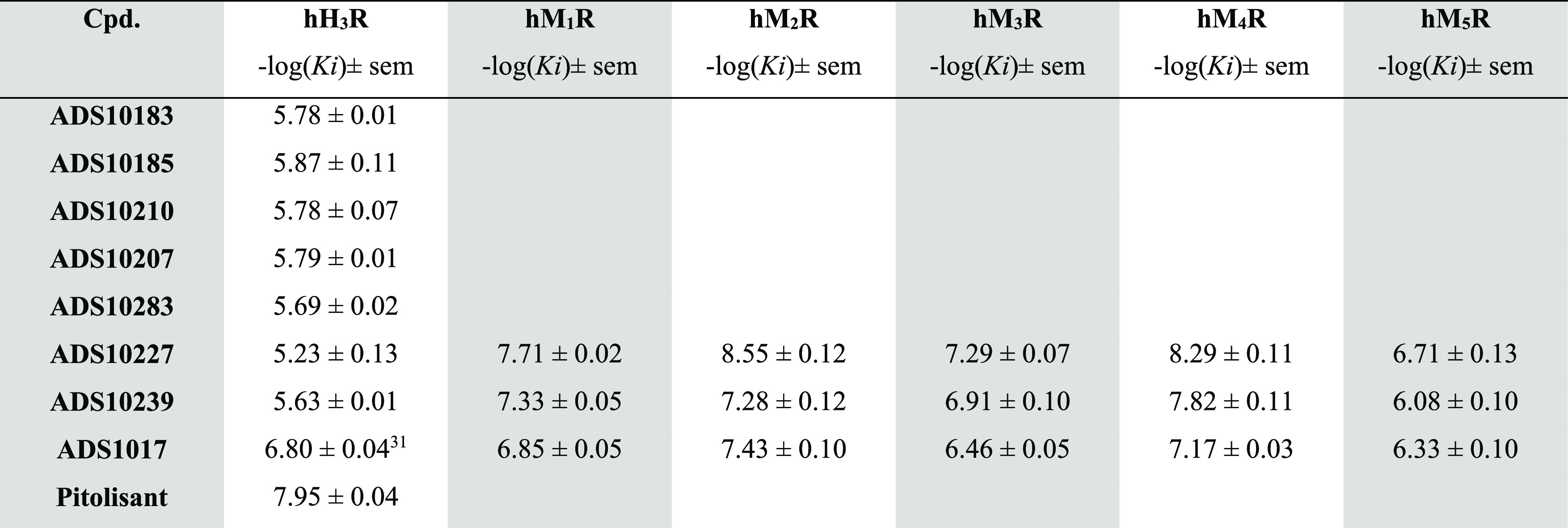
Radioligand Binding Results at Human Histamine H_3_R and Muscarinic M_1_R–M_5_R^a^

a Inhibition constants *K*_*i*_ are expressed as negative logarithms. Values are means ± sem from at least three independent experiments; sem: standard error of the mean; h: human.

#### *Ex Vivo* Screening of Histamine H_3_R Antagonist on Guinea Pig Ileum

The H_3_R antagonist potency of the newly synthesized compounds was measured on the isolated guinea pig ileum electrically stimulated to the contractions according to Vollinga et al.^35^ During the *ex vivo* assay, some additional effects were noticed. Concentrations of tested compounds of 0.3 μM and lower shifts the concentration–response curve very slightly to the right compared to the reference (*R*)(−)-α-methylhistamine (RAMH) curve, while the higher concentrations necessary to determine the pA_2_ value significantly decreased electrically evoked tissue contractions. This effect prevented the applied functional assay from determining the pA_2_ value for the H_3_R. Nevertheless, the tested compounds were found to modify the contractility of the guinea pig ileum. This effect could be related to H_3_R agonism as well as on the action on the muscarinic receptors present in the tested tissue. It is worth noting that M_2_R and M_3_R antagonists may decrease the electrically evoked contractility of ileum smooth muscles. In contrast to cell line methods, the functional *ex vivo* tests on the guinea pig ileum allows the effect on a single receptor to be tested, by blocking other receptors as well as interactions with other receptors due to the physiological complexity of animal tissues. In the next stage of the study, we evaluated the impact on the histamine and muscarinic receptors.

#### Decrease of Contractility in Electrically Stimulated Guinea Pig Ileum

A standard *ex vivo* assay based on the relaxant response of histamine H_3_R agonists to electrically driven guinea pig ileum was used to test the influence of **ADS** compounds on the reduction of electrically evoked tissue contraction. This study, standardly used for screening H_3_R agonists, was recruited to confirm or exclude **ADS** compounds as potential H_3_R agonists. Testing agonists common measurement to quantify the potency is −log EC_50_, defined as a molar concentration of an agonist required to produce 50% of the maximal response to the agonist.^36^ The −log EC_50_ value for RAMH and **ADS** compounds were evaluated. The results ranged from 5.51 (**ADS10185**) to 6.88 (**ADS10227**) and 7.70 for RAMH (Table 1). (*Z*)-Isomers (**ADS10227**, **ADS10239**) were the most effectively reduced contractility. Compounds with disubstituted *p*-phenylene group and (*E*)-isomers containing a 1,4-cyclohexylene group demonstrated a lower influence on contractility reduction. Another unique value that describes an agonist is intrinsic activity (the maximal response to an agonist expressed as a fraction of the maximal response for the entire system), where α = 1 indicates that the agonist produces the maximal response.^36^ Based on the concentration–response curves, a significant difference was observed between the intrinsic activity of RAMH and **ADS** compounds (Supporting Information): The values were 0.82 for RAMH, 0.97 for **ADS10227** (Supporting Information; Figure S44), and 0.98 for **ADS10239** (Supporting Information; Figure S45). To confirm or exclude **ADS** compounds as H_3_R agonists, the compound with the highest −log EC_50_ was selected for further studies. Therefore, **ADS10227** was used as a potential H_3_R agonist and thioperamide as the H_3_R antagonist. The study excluded **ADS10227** as an H_3_R agonist, as no shifts of the concentration–response curve of **ADS10227** were observed, compared to those with or without thioperamide. The observed reduction of electrically evoked tissue contraction cannot be associated with H_3_R agonism, and the values obtained for **ADS** compounds are not intrinsic activity of H_3_R agonist. However, observed effects must be related to a different contractility reduction mechanism. At this stage, we associated observed effects with muscarinic receptors, which are able to decrease electrically evoked ileum to the contraction. Following this, we decided to evaluate the **ADS** series as potential M_2_R/M_3_R ligands on the isolated guinea pig ileum.

#### *Ex Vivo* Screening of Histamine H_1_R Antagonist on Guinea Pig Ileum

The second histamine receptor located in the guinea pig ileum is H_1_R. Because previously described data for compound **ADS1017** showed weak, competitive H_1_R antagonist potency, all newly synthesized compounds were also evaluated for H_1_R. The H_1_R antagonistic effect was measured on isolated guinea pig ileum stimulated to contract by histamine.^37^ To assess and exclude the influence of the **ADS** compounds on muscarinic receptors, two test variations were used: one with the addition of 0.05 μM atropine and one without. The atropine method indicated an effect on the H_1_R, while the atropine-free method demonstrated effects on H_1_R and M_2_R/M_3_R. The highest pA_2_ ratio between the two methods was observed for **ADS10227**, which further confirmed our presumption about the effect on muscarinic receptors.

#### *Ex Vivo* Screening of Muscarinic M_2_R/M_3_R Antagonist on Guinea Pig Ileum

Another group of guinea pig intestinal receptors able to regulate smooth muscle contraction is that of the muscarinic receptors, including M_2_R and M_3_R. All compounds were tested on the muscarinic receptors using the same animal model. This approach is similar to measuring histamine H_1_R antagonist activity. Methacholine was used as a receptor agonist, while 1,1-dimethyl-4-diphenylacetoxypiperidinium iodide (4-DAMP) was used as a reference M_3_R antagonist. As methacholine is not selective according to M_2_R and M_3_R, it is not possible to precisely specify which one was responsible for the effect observed on the tissue preparations. Some authors often attribute the results to the minor M_3_R subtype (gpM_2_R:M_3_R = 4:1 or 5:1), which is generally associated with the contractions,^38^ but such an overinterpretation may be misleading, as indicated by our further hM_1_R-hM_5_R radioligand binding assay results shown in Table 2. The tested series demonstrates low to moderate affinities at the muscarinic receptors (pA_2_ = 5.61–6.97) (Table 1). The two (*Z*)-isomers, **ADS10227** and **ADS10239**, were the most potent muscarinic receptor antagonists in the series. Compounds with the highest pA_2_ value against the muscarinic receptor demonstrated the greatest reduction of electrically evoked contraction as well (Table 1). As it was mentioned above, the highest pA_2_ ratio between the two screening methods of histamine H_1_R antagonist was observed for **ADS10227**, which was also the most potent muscarinic M_2_R/M_3_R antagonist. These findings partially explain the influence of **ADS10227** on the contractility of guinea pig ileum following electrical stimulation. The potent H_3_R antagonist **ADS1017** also demonstrated moderate affinity (pA_2_ = 6.36) to muscarinic receptors. This observation explains the effect of tested compounds on the isolated guinea pig ileum, but it does not identify the muscarinic receptor subtype the **ADS** compounds act on. In subsequent studies, we tried to clarify this.

#### hH_3_ Radioligand Binding Assay

As it was not possible to determine the pA_2_ value by measuring the potency of H_3_R on electrically stimulated guinea pig ileum *ex vivo*, another research method was needed to evaluate the affinity of the **ADS** compounds for the histamine H_3_R. Hence, the displacement binding assay was performed in membrane fractions of HEK-293 cells stably expressing hH_3_R to determine the hH_3_R binding affinities of the final compounds. [^3^H]-*N*^α^-Methylhistamine was used as a radiolabeled ligand. Compounds with a 1,4-cyclohexylene or *p*-phenylene group incorporated into a 7-phenoxyheptyl residue showed at least a 10-fold decrease of affinity relative to **ADS1017**: The p*K*_i_ of **ADS1017** was 6.80, while that of **ADS10227** was 5.23, this being the lowest in the series (Table 2). Measuring the displacement curve obtained for [^3^H]-*N*^α^-methylhistamine from the human histamine H_3_R in HEK-293 cell membranes, we confirmed a decrease in affinity to the hH_3_R.

#### hM_1_R–hM_5_R Radioligand Binding Assays

Unexpectedly, the tests carried out on the guinea pig ileum showed that the rigidity of the seven-carbon alkyl chain by 1,4-cyclohexylene or *p*-phenylene group not only decreases affinity at the histamine H_3_R but also significantly increases activity at muscarinic receptors. However, the previously employed method does not strictly elucidate on which one of the muscarinic receptor subtypes the tested compounds act on. To clearly explain the obtained outcome, we engaged the radioligand binding experiment. The hM_1_–hM_5_ radioligand binding experiments were performed in the membrane fractions of Chinese hamster ovary cells (CHO) stably expressing human variants of muscarinic receptors. *N*-[^3^H]Methylscopolamine was used as the radiolabeled ligand.^39^ Three compounds were selected from *ex vivo* screening on guinea pig ileum. The potent histamine H_3_R antagonist, **ADS1017**, showed nanomolar affinities to the hM_2_R (37 nM; −log *K*_*i*_ = 7.43) and hM_4_R (68 nM; −log *K*_*i*_ = 7.17). This compound is also not completely selective for the remaining muscarinic receptor subtypes with −log *K*_i_ values over 6. **ADS10227** demonstrated the highest affinity to hM_2_R and hM_4_R: 2.8 nM and 5.1 nM, respectively. It is also a potent muscarinic M_1_R antagonist, and the M_1_R/M_2_R selectivity ratio is <10. It is worth noting that such nonspecific anticholinergics are often associated with neuropsychiatric and cognitive disturbances and that nonselective muscarinic M_1_R/M_2_R antagonists, such as scopolamine, can produce cognitive deficits. Further structural modifications of **ADS10227** should be used to increase selectivity toward the M_2_R, as these may present fewer potential side effects associated with the activation of the phospholipase C signaling pathway. The third tested compound **ADS10239** showed affinity to hM_1_R, hM_2_R, and hM_4_R: 45 nM, 52 nM, and 15 nM, respectively.

#### Intracellular Ca^2+^ Measurement

The intracellular calcium level was determined fluorometrically to indicate the potency of **ADS10227** to antagonize the functional response of muscarinic M_2_R (p*K*_B_ = 8.17) and M_3_R (p*K*_B_ = 7.43) expressed on CHO cells to ACh. Linear Schild plot indicates the competitive interaction (slope = 1) of the tested compound (Supporting Information, Figure S55).

#### *In Silico* Studies on Receptor Bindings and Selectivity

We conducted *in silico* molecular modeling studies to understand the molecular basis of the unexpected muscarinic activity of **ADS1017** and its newly obtained derivatives. In this way, we wanted to determine the binding mode of the tested ligands to H_3_R, M_2_R, and M_4_R and reveal the structural elements that are key to the interaction with these biological targets. From our point of view, it seems crucial to understand the effects of linker cyclization, which resulted in a strong shift of the activity profile of guanidine derivatives toward muscarinic receptors. Therefore, the next part of the study examined the binding mode of the analyzed compounds to the M_2_R (PDB: 5ZKB) in an inactive state and to the remodeled M_4_R and H_3_R.

**Figure 2:**
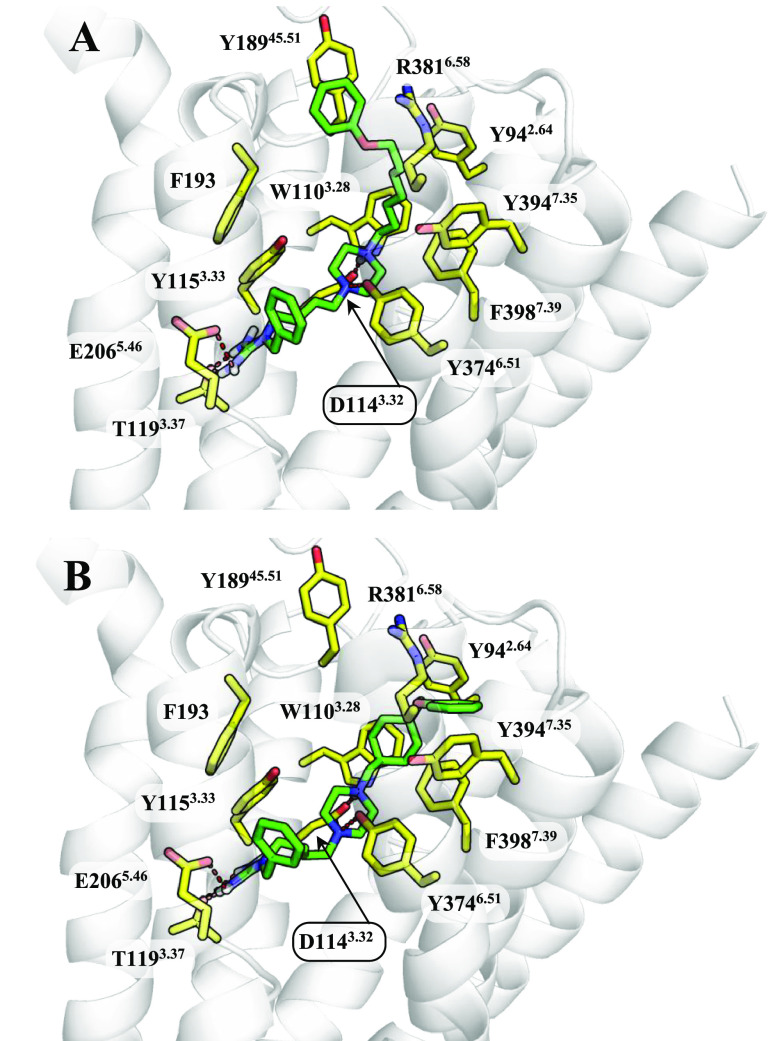
Binding mode of (A) **ADS1017** and (B) **ADS10227** to the histamine H_3_R. The ligands are shown as green sticks and the most important amino acids for interaction with the ligand as yellow sticks.

As the tested compounds were designed and optimized for the inhibition of the histamine H_3_R, this was taken as our reference point. The comparison of the binding modes of the **ADS1017** (−log *K*_*i*_ (H_3_R) = 6.80) and **ADS10227** (−log *K*_*i*_ (H_3_R) = 5.23) provided some information that could explain the observed decrease in affinity. The final poses of the **ADS1017** and **ADS10227** at the histamine H_3_R binding site are shown in Figure 2.

Docking studies indicated a very consistent binding mode. The locations of key ligand moieties were reproducible for all tested compounds. One of the most important features of this binding mode was the involvement of the entire orthosteric receptor binding site in the interaction with ligands.

The participation of two elements of the orthosteric binding site requires special emphasis. The first is the recognition site for the histamine imidazole fragment.^13^ It is composed of the amino acids from three transmembrane domains: TM3, TM5, and TM6. In the case of the histamine H_3_R, E206^5.46^ plays a very important role. The test compounds interact with this site via the benzyl-bound guanidino moiety. The positively charged guanidine forms an ionic bond with E206^5.46^ and a cation−π with Y167^4.57^. In addition, T119^3.37^ stabilizes the system through hydrogen bonding. The benzyl fragment occupies a hydrophobic pocket built by F193, L199^5.39^, W371^6.48^, and M378^6.55^. The second important element of the binding site is D114^3.32^ which physiologically interacts with the protonated amine moiety of histamine.^13^ The tested compounds bind to D114^3.32^ via the piperazine ring. According to our prediction, the piperazine ring of **ADS1017** and **ADS10227** was protonated at the nitrogen atom substituted by the 7-phenoxyheptyl or 4-(phenoxymethyl)cyclohexylmethyl substituent, respectively. This described binding mode indicates the participation of the first, protonated amine in the ionic interactions with D114^3.32^ (salt bridge) and W110^3.28^ (cation−π). The second, nonionized amino group was an acceptor of the hydrogen bond formed with the hydroxyl group Y374^6.51^. Based on these observations, we can assume that both piperazine nitrogen atoms are essential, and removal of any of them may lead to weakening of ligand binding. This observation is consistent with the results of previous experiments.^31^

We found that the activity differences in the studied group of compounds are related to ligand alignment in the hydrophobic regions between TM2, TM3, and TM7. This area includes a number of aromatic amino acids such as Y91^2.61^, Y94^2.64^, W110^3.28^, F398^7.39^, and W402^7.43^. The arrangement in this space determines the interaction of the phenoxy group in the so-called allosteric binding site. The long and flexible aliphatic linkage of **ADS1017** allows the phenoxy group to create interactions with the amino acids of the outer part of the so-called allosteric site such as Y189^45.51^ (π–π) and R381^6.58^ (cation−π). Similar interactions were not observed in any of the newly synthesized compounds, which may be the reason for their much weaker interaction with H_3_R.

**Figure 3:**
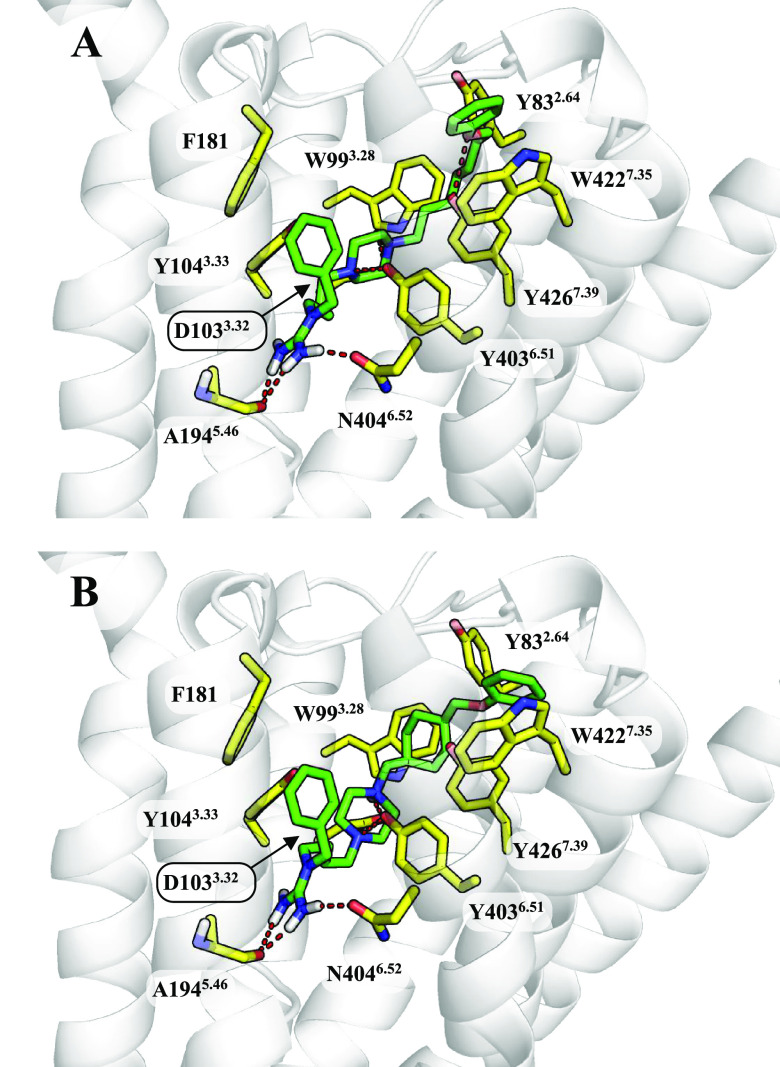
Binding modes of (A) **ADS1017** and (B) **ADS10227** to the muscarinic M_2_R. The ligands are shown as green sticks and the most important amino acids for interaction with the ligand as yellow sticks.

In the case of muscarinic receptors, changes in the TM3, TM5, and TM6 regions responsible for selectivity for physiological ligands (ACh) alter the position of the guanidine fragment and benzyl group. The final binding poses of the **ADS1017** and **ADS10227** at the muscarinic M_2_ receptor binding site are shown in Figure 3.

**Figure 4:**
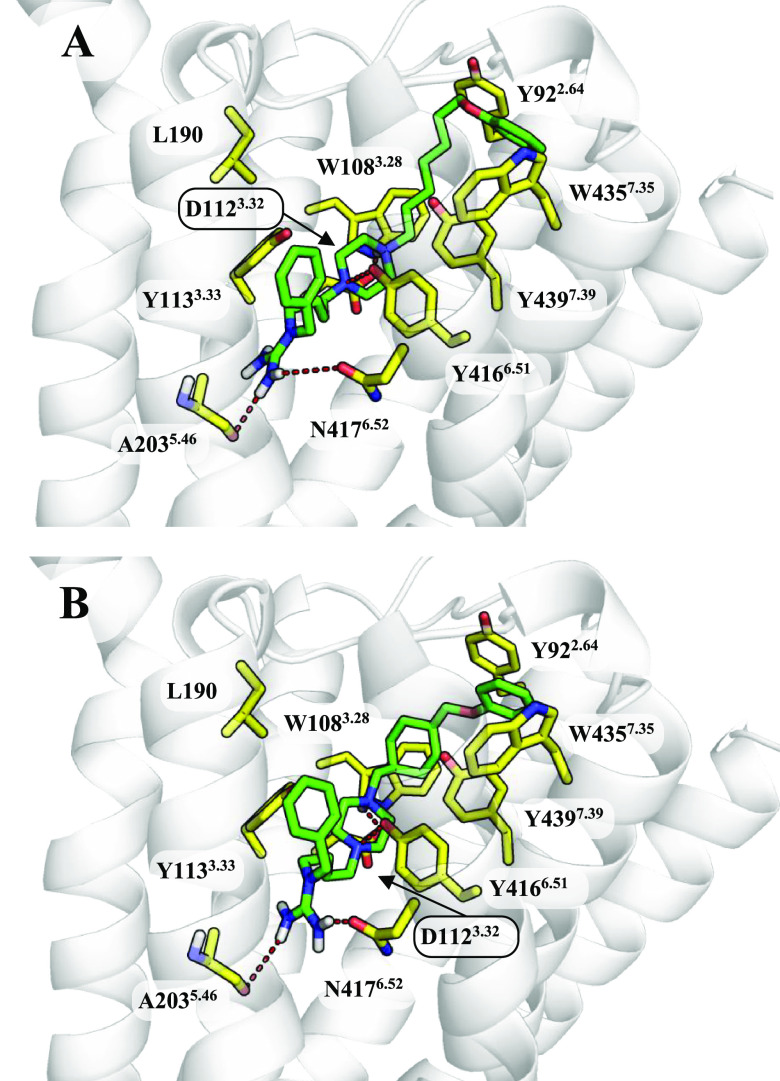
Binding modes of (A) **ADS1017** and (B) **ADS10227** to the muscarinic M_4_R. The ligands are shown as green sticks and the most important amino acids for interaction with the ligand as yellow sticks.

The key amino acids for both the muscarinic M_2_R and M_4_R (amino acid numeration M_2_R/M_4_R) are N404^6.52^/N417^6.52^ and A194^5.46^/A203^5.46^. They form a hydrogen-bond network with the guanidine group of the ligand. The associated aromatic ring was placed higher than during the binding of the same compound to the histamine H_3_R, which strengthens the interactions with F181 and Y403^6.51^ in the muscarinic M_2_R. On this basis, we assumed that the switch from F181 in M_2_R to L190 in M_4_R may explain the slight difference between the activities at these receptors. The final binding poses of the **ADS1017** and **ADS10227** at the muscarinic M_4_R binding site are shown in Figure 4.

The piperazine ring located in the middle of the ligand creates similar interactions in the active sites of the M_2_R and M_4_R to those observed in the H_3_R. These regions are nearly identical between those receptors, which explains the affinity of the tested compounds against these biological targets. Again, the area between TM2 and TM7 contributes to ligand binding in this region and influences the position of the phenoxy group at the so-called allosteric binding site. 1,4-Cyclohexylene or *p*-phenylene group bind more strongly to the muscarinic receptor due to the slight differences in the structure of the outer part of the receptors. One of the key factors responsible for the stronger affinity of **ADS1017** to H_3_R seems to be the involvement of R381^6.58^ in the creation of cation−π interactions. This interaction is possible thanks to a long flexible aliphatic chain between the piperazine ring and the *p*-phenylene group. Change to N410^6.58^/N423^6.58^ present in M_2_R and M_4_R weakens the binding of **ADS1017** to these receptors. The binding mode of compounds with the cyclized linker indicates a preference for aromatic interactions with amino acids located on TM2 and TM7 of muscarinic receptors. In the compound **ADS10227**, Y80^2.61^/Y89^2.61^, Y83^2.64^/Y92^2.64^, W422^7.35^/W435^7.35^, and Y426^7.39^/Y439^7.39^ participate in the phenoxy group bonding. In this case, the change from H_3_R Y394^7.35^ to W422^7.35^/W435^7.35^ present in M_2_R and M_4_R allows a stronger binding of the aromatic system in ligands with cyclized linkers. A two-dimensional map of interactions between the **ADS1017** and **ADS10227** ligands and H_3_, M_2_, and M_4_ receptors is presented in Supporting Information.

The *in silico* research yields two important observations. The first is the benzylguanidine fragment demonstrates a universal match for the recognition sites of specific ligands of the H_3_R, M_2_R, and M_4_R located between TM3, TM5, and TM6. This is an important finding for scientists developing similar compounds because of their potential interaction with unintended biological targets (off-target determination). On-targeted optimization of such fragments can direct the affinity/activity profile of developed compounds. Second, the piperazine ring demonstrates a very stable position and may serve as a good core for new compounds with strong affinity to both the H_3_R and muscarinic receptors. The key interactions created by **ADS1017** and **ADS10227** within H_3_R, M_2_R, and M_4_R binding sites are presented in Table 3.

**Table 3:**
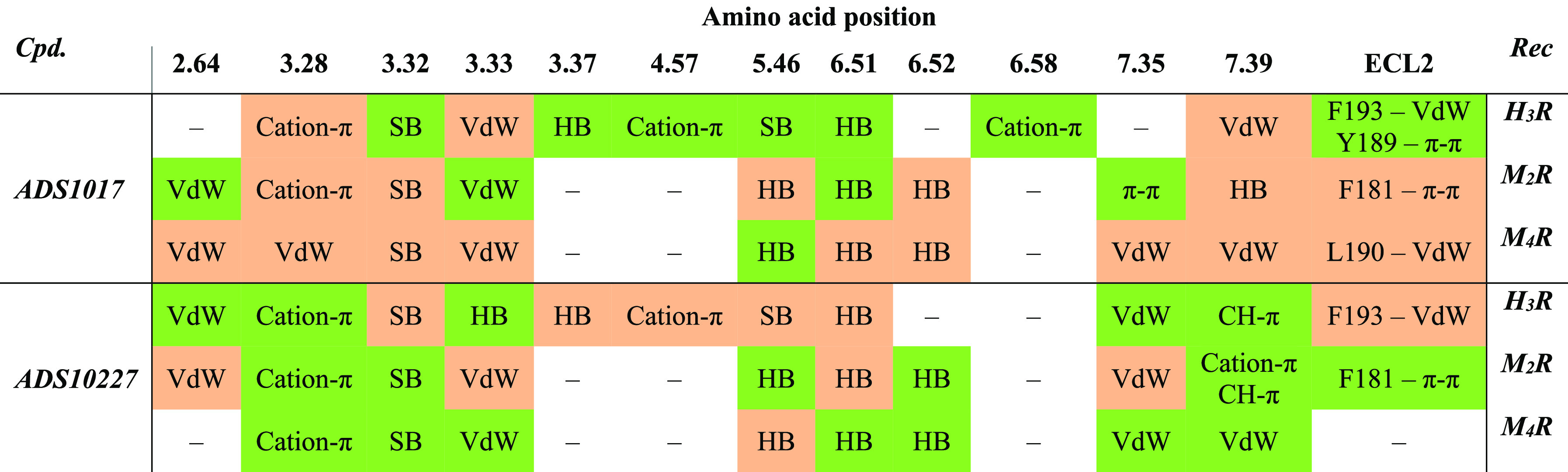
Interactions Created by **ADS1017** and **ADS10227** within H_3_, M_2_, and M_4_ Receptors Binding Sites^a^

a The amino acids with the strongest impact on the ligand binding are presented according to the Ballesteros–Weinstein convention. The strength of individual interactions was assessed using the Emodel evaluation function.^40^ Stronger interactions are marked in green and the weaker ones in red. Blanks indicate no significant contribution to ligand binding. VdW: van der Waals force; SB: salt bridge; HB: hydrogen bonds.

## Conclusion

A series of new guanidine derivatives was synthesized to identify new nonimidazole histamine H_3_R antagonists. The seven-carbon chain present in the lead compounds **ADS1017** and **ADS1020** (Chart 1) was replaced by a semirigid moiety containing 1,4-cyclohexylene or *p*-phenylene group. In all cases, the substitution resulted in the significant decrease of H_3_R antagonistic activity as well as the formation of potent muscarinic M_2_R and M_4_R antagonists which showed antagonist affinities in single-digit nanomolar concentration ranges. It is noteworthy that the histamine H_3_R and muscarinic receptors have very similar binding sites (Table S1). The pharmacological profiling of the newly synthesized compounds led us to the identification of the most promising compound **ADS10227**, demonstrating the highest affinity to the hM_2_R (2.8 nM; −log *K*_*i*_ = 8.55) and hM_4_R (5.1 nM; −log *K*_*i*_ = 8.29) compared to the low affinity at H_3_R and the other muscarinic receptors. This compound favoring muscarinic M_2_R and M_4_R may serve as a new lead structure for further structural modifications to develop a novel class of selective M_2_R antagonists useful in the treatment of cognition deficit diseases such as Alzheimer’s disease, schizophrenia, or CNS learning disorders, such as autism or attention deficit disorder. **ADS1017** also remains within the scope of interest as a dual-active H_3_R/M_2_R antagonist in relation to the treatment of cognition deficit disorders.

All the newly synthesized compounds were guanidine derivatives, which is the new chemotype of muscarinic receptor antagonists. Previous studies have described guanidine-containing compounds that can act as muscarinic receptor antagonists; however, the correct chemical nomenclature should classify them as carboximiamides.^41^ All muscarinic receptor subtypes share a high sequence homology in the binding site, which hinders the discovery of subtype-selective ligands. A small number of pharmacological agents that are selective to muscarinic receptors subtypes still remains challenging in the development of therapeutics that target muscarinic receptors. Such selective muscarinic ligands are needed to prevent undesired side-effects.

The major achievement of this study is the development of the **ADS10227** nonselective muscarinic receptor antagonist. The separation of the *E/Z*-isomers of derivatives bearing a 1,4-cyclohexylene group provides a clearer picture of the spatial conformation of (*Z*)-isomers to fit the binding site of the M_2_R and M_4_R. The novel set of obtained ligands may constitute a promising toolbox to study the requirements of muscarinic receptors and could serve as starting points for further structural modifications, leading to the design of compounds with nanomolar affinity at muscarinic M_2_R or M_4_R.

## Methods

### Chemistry

All solvents were purchased from commercial suppliers (e.g., Avantor Performance Materials Poland S.A., PPH Stanlab Sp. z o.o. Lublin, Chempur Piekary Slaskie) and were used without further purification. The *E/Z* mixture of 1,4-cyclohexanedimethanol, phosphorus bromide, piperazine, phenol, sodium, 4-bromobutyronitrile,4-(trifluoromethyl)benzoyl chloride, benzyl bromide, 1,3-bis(*tert*-butoxycarbonyl)-2-methylisothiourea, 1,4-bis(bromomethyl)benzene, sodium, phenol, 1-(bromomethyl)-4-phenoxybenzene, and 4 M solution HCl in dioxane were purchased from commercial suppliers (Aldrich, TCI, Fluorochem, Fluka) and used without further purification. Nuclear magnetic resonance (NMR) spectra (^1^H NMR, ^13^H NMR) were recorded on a Bruker Avance III 600 MHz (^1^H NMR spectra were run at 600 MHz, while ^13^C NMR spectra were run at 150.95 MHz) spectrometer in CDCl_3_, CD_3_OD, and deuterium oxide. Chemical shifts were expressed in δ values, parts per million (ppm) using the solvent signal as an internal standard, and coupling constants (*J*) were given in hertz (Hz). Spectra obtained in deuterated chloroform were referenced to tetramethylsilane at 0.00 ppm for ^1^H spectra and 77.02 ppm for ^13^C spectra. Spectra obtained in CD_3_OD were referenced to residual CD_3_OD at 3.31 ppm for ^1^H spectra and 49.0 ppm for ^13^C spectra. Spectra obtained in deuterium oxide were referenced to residual deuterium oxide at 4.76 ppm for ^1^H spectra. Signal multiplicities were characterized as br (broad), s (singlet), d (doublet), t (triplet), q (quartet), qt (quintet), m (multiplet), and * (exchangeable by deuterium oxide). Elemental analysis (C, H, and N) for all compounds were measured on PerkinElmer Series II CHNS/O analyzer 2400 and were within ±0.4% of the theoretical values. Reactions were monitored by thin-layer chromatography (TLC) on silica gel 60 F254 plates (Merck) and visualized using a UV Lamp (254 nm) and cerium molybdate stain. Flash column chromatography was performed using silica gel 60 Å 50 mm (J. T. Baker B. V.) and Normasil 60 silca gel 40–63 μm (VWR Chemicals), employing eluent indicated by TLC. Melting points (mp) were measured in open capillaries on an Electrothermal apparatus (Electrothermal, Southend, England) and are uncorrected.

#### Preparation of (*E*)-1,4-Cyclohexanedimethanol dibenzoate (**1a**) and (Z)-1,4-Cyclohexanedimethanol dibenzoate (**1b**)

A solution of benzoyl chloride (40.97 g, 0.29 mol) in 80 mL of DCM was added dropwise to an ice-cooled mixture of 1,4-cyclohexanedimethanol (*E*/*Z* mixture) (20.03 g; 0.14 mol) and triethylamine (84.00 g; 0.83 mol) in 200 mL of DCM. The reaction was stirred for 2.5 h at room temperature, then the mixture was washed sequentially twice with 200 mL of water. The water phase was washed four times with 50 mL of DCM, then the combined organic phases were dried over Na_2_SO_4_. The solvent was removed under vacuum, and the crude product was recrystallized twice from ethyl acetate to yield the pure products as a plaques (*E-*isomer). The filtrate obtained after the first recrystallization was collected, evaporated, and recrystallized twice from ethyl acetate collecting a (*Z*)-isomer-rich fraction (evaluated base on the NMR spectra) to yield the pure products as needles (*Z*-isomer).

##### (*E*)-1,4-Cyclohexanedimethanol dibenzoate (**1a**)

C_22_H_24_O_4_. M = 352.43. Transparent plaques. 39.43% yield. Retardation factor (*R*_*f*_) = 0.43 (hexane/EtOAc 9:1). Mp: 125.3–127.0 °C. ^1^H NMR (600 MHz, CDCl_3_) δ ppm 8.05–8.04 (m, 4H^arom.^, CH(CHCH)_2_C), 7.56–7.54 (m, 2H^arom.^, CH(CHCH)_2_C), 7.44–7.42 (m, 4C^arom.^, CH(CHCH)_2_C), 4.18 (d, 4H, OCH_2_, *J* = 6.42 Hz), 1.95–1.94 (m, 4H^cyclohexyl.^, CH_2_), 1.81 (m, 2H ^cyclohexyl.^, OCH_2_CH), 1.20–1.13 (m, 4H^cyclohexyl.^, CH_2_). ^13^C NMR (150.95 MHz, *CDCl*_*3*_) δ ppm 166.61 (2C, C=O), 132.84 (2C^arom.^, CH(CHCH)_2_C), 130.50 (2C^quat./arom..^, CO), 129.55 (4C^arom.^, CH(CHCH)_2_C), 128.35 (4C^arom.^, CH(CHCH)_2_C), 69.77 (2C, OCH_2_), 37.32 (2C ^cyclohexyl.^, OCH_2_CH), 29.02 (4C^cyclohexyl.^, CH_2_).

##### (*Z*)-1,4-Cyclohexanedimethanol dibenzoate (**1b**)

C_22_H_24_O_4_. M = 352.43. Transparent needles. 16.35% yield. *R*_*f*_ = 0.46 (hexane/EtOAc 9:1). Mp: 84.8–86.4 °C. ^1^H NMR (600 MHz, *CDCl*_*3*_) δ ppm 8.06–8.04 (m, 4H^arom.^, CH(CHCH)_2_C), 7.56–7.54 (m, 2H^arom.^, CH(CHCH)_2_C), 7.45–7.43 (m, 4H^arom.^, CH(CHCH)_2_C), 4.28 (d, 4H, OCH_2_, *J* = 7.26 Hz), 2.06–2.04 (m, 2H^cyclohexyl.^, OCH_2_CH), 1.69–1.65 (m, 4H^cyclohexyl.^, CH_2_), 1.62–1.56 (m, 4H^cyclohexyl.^, CH_2_). ^13^C NMR (150.95 MHz, CDCl_3_) δ ppm 166.60 (2C, C=O), 132.84 (2C^arom.^, CH(CHCH)_2_C), 130.45 (2C^quat./arom..^, CO), 129.54 (4C^arom.^, CH(CHCH)_2_C), 128.34 (4C^arom.^, CH(CHCH)_2_C), 67.59 (2C, OCH_2_), 34.69 (2C ^cyclohexyl.^, OCH_2_CH), 25.46 (4C^cyclohexyl.^, CH_2_).

#### Preparation of (*E*)-1,4-cyclohexanedimethanol (**2a**)

A solution of sodium hydroxide (8.80 g; 0.22 mol) in 10.8 mL of water was added to a mixture of (*E*)-1,4-cyclohexanedimethanol dibenzoate (**1a**) (7.90 g; 2.2 × 10^–2^ mol) in 250 mL of methanol. The reaction was stirred overnight at 70 °C. The solvents were removed under vacuum. The residue was diluted by 50 mL of water and extracted 5 × 50 mL with EtOAc. The organic phase was dried over anhydrous Na_2_SO_4_. The solvent was removed under vacuum to yield the pure product.

##### (*E*)-1,4-Cyclohexanedimethanol (**2a**)

C_8_H_16_O_2_. M = 144.21. White waxy solid. 88.55% yield. *R*_*f*_ = 0.59 (EtOAc). Mp: 65.4–67.4 °C. ^1^H NMR (600 MHz, *CDCl*_*3*_) δ ppm 3.47 (d, 4H, HOCH_2_, *J* = 6.27 Hz), 1.85–1.84 (m, 4H^cyclohexyl.^, CH_2_), 1.46 (br, 4H: 2H^cyclohexyl.^, OCH_2_CH; OH), 1.02–0.94 (m, 4H^cyclohexyl.^, CH_2_). ^13^C NMR (150.95 MHz, *CDCl*_*3*_) δ ppm 68.62 (2C, OCH_2_), 40.65 (2C^cyclohexyl.^, CH), 28.91 (4C^cyclohexyl.^, CH_2_).

#### Preparation of (*Z*)-1,4-Cyclohexanedimethanol (**2b**)

A solution of sodium hydroxide (6.00 g; 0.15 mol) in 9 mL of water was added to a mixture of (*Z*)-1,4-cyclohexanedimethanol dibenzoate (**1b**) (5.33 g; 1.5 × 10^–2^ mol) in 200 mL of methanol. The reaction was stirred overnight at 70 °C. The solvents were removed under vacuum. The residue was diluted by 50 mL of water and extracted 5 × 50 mL with EtOAc. The organic phase was dried over anhydrous Na_2_SO_4_. The solvent was removed under vacuum, and the crude product was purified by column chromatography (EtOAc) to yield the pure product.

##### (*Z*)-1,4-Cyclohexanedimethanol (**2b**)

C_8_H_16_O_2_. M = 144.21. Colorless sticky oil. 91.10% yield. *R*_*f*_ = 0.49 (EtOAc). Mp: 65.4–67.4 °C. ^1^H NMR (600 MHz, *CDCl*_*3*_) δ ppm 3.55 (d, 4H, HOCH_2_), 1.70–1.69 (m, 2H^cyclohexyl.^, OCH_2_CH), 1.57–1.53 (m, 4H^cyclohexyl.^, CH_2_), 1.46–1.39 (m, 6H: m, 4H^cyclohexyl.^, CH_2_, OH). ^13^C NMR (150.95 MHz, *CDCl*_*3*_) δ ppm 66.09 (2C, CH_2_OH), 38.12 (2C^cyclohexyl.^, CH), 25.31 (4C^cyclohexyl.^, CH_2_).

#### Preparation of (*E*)-1,4-Bis(bromomethyl)cyclohexane (**3a**)

A solution of (*E*)-1,4-cyclohexanedimethanol (**2a**) (3.20 g; 2.22 × 10^–2^ mol) in 10 mL of DMF was added dropwise to an ice-cooled mixture of phosphorus bromide (8.45 g; 3.12 × 10^–2^ mol) in 10 mL of toluene. Then, the reaction was stirred for 90 min at 100 °C. The mixture was cooled, and 50 mL of crushed ice was added and then extracted 3 × 20 mL with DCM. The organic phases were combined and dried over anhydrous Na_2_SO_4_. The solvent was removed under vacuum, and the crude product was purified by column chromatography (hexane) to yield the pure product.

##### (*E*)-1,4-Bis(bromomethyl)cyclohexane (**3a**)

C_8_H_14_Br_2_. M = 270.00. Transparent crystals. 71.28% yield. *R*_*f*_ = 0.61 (hexane). Mp: 54.2–55.2 °C. ^1^H NMR (600 MHz, *CDCl*_*3*_) δ ppm 3.29 (d, 4H, BrCH_2_, *J* = 6.28 Hz), 1.95–1.94 (m, 4H^cyclohexyl.^, CH_2_), 1.61 (m, 2H^cyclohexyl.^, BrCH_2_CH), 1.08–1.05 (m, 4H^cyclohexyl.^, CH_2_). ^13^C NMR (150.95 MHz, *CDCl*_*3*_) δ ppm 39.89 (2C, BrCH_2_), 39.81 (2C^cyclohexyl.^, CH), 31.06 (4C^cyclohexyl.^, CH_2_).

#### Preparation of (*Z*)-1,4-Bis(bromomethyl)cyclohexane (**3b**)

A solution of (*Z*)-1,4-cyclohexanedimethanol (**2b**) (1.92 g; 1.33 × 10^–2^ mol) in 6 mL of DMF was added dropwise to an ice-cooled mixture of phosphorus bromide (4.33 g; 1.60 × 10^–2^ mol) in 5 mL of toluene. Then, the reaction was stirred for 90 min at 100 °C. The mixture was cooled, and 30 mL of crushed ice was added and then extracted 3 × 15 mL with DCM. The organic phases were combined and dried over anhydrous Na_2_SO_4_. The solvent was removed under vacuum, and the crude product was purified by column chromatography (hexane) to yield the pure product.

##### (*Z*)-1,4-Bis(bromomethyl)cyclohexane (**3b**)

C_8_H_14_Br_2_. M = 270.00. Colorless liquid. 84.58% yield. *R*_*f*_ = 0.72 (hexane). ^1^H NMR (600 MHz, *CDCl*_*3*_) ppm 3.40 (d, 4H, BrCH_2_), 1.94–1.82 (m, 2H^cyclohexyl.^, BrCH_2_CH), 1.69–1.58 (m, 4H^cyclohexyl.^, CH_2_), 1.56–1.49 (m, 4H^cyclohexyl.^, CH_2_). ^13^C NMR (150.95 MHz, *CDCl*_*3*_) δ ppm 38.06 (2C, BrCH_2_), 37.64 (2C^cyclohexyl.^, CH), 27.05 (4C^cyclohexyl^, CH_2_).

#### General Procedure for the Preparation of Compounds **4a**, **4b**, and **11**

Phenol (1 equiv) was added to sodium (1.05 equiv) dissolved in anhydrous ethanol and stirred for 30 min at room temperature, yielding sodium phenoxide solution. A freshly prepared sodium phenoxide solution (or evaporated sodium phenoxide solution dissolved in THF) was added dropwise to a solution of the corresponding bromide (1 equiv) (**3a**, **3b**) in anhydrous ethanol and heated to 65 °C (or in anhydrous THF heated to 66 °C for compounds 1,4-bis(bromomethyl)benzene). The reaction was stirred overnight at 80 °C. The solvent was removed under vacuum, and the mixture was purified by column chromatography to yield the pure product.

#### General Procedure for the Preparation of Compounds **5a**, **5b**, **12**, and **18**

A mixture of corresponding bromide (1 equiv) (**4a**, **4b**, **11**, and 1-(bromomethyl)-4-phenoxybenzene) in anhydrous THF was added dropwise to a solution of piperazine (5 equiv) in THF heated to 66 °C. The reaction was stirred overnight at 66 °C. The precipitate was discarded. The solvent was removed under vacuum, and the residue was diluted by water, alkalized by 5% NaOH solution, and extracted with DCM. The combined organic phases were dried over anhydrous Na_2_SO_4_. The solvent was removed under vacuum, and the crude product was purified by column chromatography to yield the pure product.

#### General Procedure for the Preparation of Compounds **6a**, **6b**, **13**, and **19**

Potassium carbonate (5 equiv) and 4-bromobutyronitrile (1.3 equiv) were added to a solution of the corresponding 1-substituted piperazine (1 equiv) (**5a**, **5b**, **12**, and **18**) in acetonitrile. The reaction was stirred overnight at 80 °C and then filtered. The precipitate was discarded. The solvent was removed under vacuum, and the crude product was purified by column chromatography to yield the pure product.

#### General Procedure for the Preparation of Compounds **7a**, **7b**, **14**, and **20**

LiAlH_4_ (4 equiv) was slowly added to a solution of the corresponding nitrile (1 equiv) (**6a**, **6b**, **13**, and **19**) in 50 mL of anhydrous diethyl ether. The reaction was stirred overnight at room temperature, and the mixture was quenched by dropwise addition of water (16 equiv) and 10% NaOH solution (16 equiv) stirred for 2 h and then filtered. The precipitate was discarded. The organic layer was dried over Na_2_SO_4_, the solvent was removed under vacuum, and the crude product was purified by column chromatography to yield the pure product.

#### General Procedure for the Preparation of Compounds **8a**, **8b**, **8c**, **8d**, **15a**, **15b**, and **21**

4-(Trifluoromethyl)benzoyl chloride (1.1 equiv) or benzoyl chloride (1.1 equiv) in DCM was added dropwise to a solution of the corresponding primary amine (1 equiv) (**7a**, **7b**, **14**, and **20**) and triethylamine (5 equiv) in DCM. The reaction was stirred for 3 h at room temperature, and then the mixture was washed three times with water and dried over Na_2_SO_4_. The solvent was removed under vacuum, and the crude product was purified by column chromatography to yield the pure product.

#### General Procedure for the Preparation of Compounds **9a**, **9b**, **9c**, **9d**, **16a**, **16b**, and **22**

LiAlH_4_ (4 equiv) was added to a solution of the corresponding amide (1 equiv) in anhydrous diethyl ether (**8a**, **8b**, **8c**, **8d**, **15a**, **15b**, and **21**) or THF (**8b**). The reaction was stirred overnight at room temperature, and the mixture was quenched by dropwise addition of water (16 equiv) and 10% NaOH solution (16 equiv) stirred for 2 h and then filtered. The precipitate was discarded. The organic layer was dried over Na_2_SO_4_, the solvent was removed under vacuum, and the crude product was purified by column chromatography to yield the pure product.

#### General Procedure for the Preparation of Compounds **10a**, **10b**, **10c**, **10d**, **17a**, **17b**, and **23**(42)

1,3-Bis(*tert*-butoxycarbonyl)-2-methylisothiourea (1.1 equiv) and mercury II chloride (1.1 equiv) were sequentially added to an ice-cooled mixture of the corresponding secondary amine (1 equiv) (**9a**, **9b**, **9c**, **9d**, **16a**, **16b**, and **22**) and triethylamine (5 equiv) in DCM. The ice bath was removed, and the reaction was stirred 18 h at room temperature and then filtered. The precipitate was discarded. The filtrate was washed sequentially twice with H_2_O and twice with brine. The combined organic phases were dried over Na_2_SO_4_, the solvent was removed under vacuum, and the crude product was purified by column chromatography to yield the pure product.

#### General Procedure for the Preparation of Compounds **ADS10207**, **ADS10239**, **ADS10283**, **ADS10227**, **ADS10183**, **ADS10185**, and **ADS10210**(42)

4 M solution HCl in 1,4-dioxane (20 equiv) was added dropwise to a solution of the corresponding Boc-protected guanidine (1 equiv) (**10a**, **10b**, **10c**, **10d**, **17a**, **17b**, and **23**) in chloroform. The reaction was stirred overnight at room temperature, and the solvent was removed under vacuum. The crude product was evaporated twice from chloroform and twice from EtOAc and then recrystallized from anhydrous ethanol or 2-propanol to yield the pure product.

### Biological Evaluation

#### *Ex Vivo* Assay for Screening Histamine H_3_R Antagonists on Guinea Pig Ileum

Male guinea pigs, weighing 300–400 g, were euthanized by a blow to the neck. Following this, a 20–30 cm length of the distal ileum, apart from the terminal 5 cm, was rapidly removed and placed in phosphate buffer at room temperature (pH 7.4) containing (mM) NaCl (136.9), KCl (2.6), KH_2_PO_4_ (1.47), Na_2_HPO_4_ (9.58), and indomethacin (1 × 10^–6^ mol/L)). The intraluminal content was rinsed, and the isolated intestine was cut into 1.5–2 cm segments. The preparations were mounted between two platinum electrodes isotonically in a 20 mL organ bath filled with Krebs buffer: composition (mM) NaCl (118), KCl (5.6), MgSO_4_ (1.18), CaCl_2_ (2.5), NaH_2_PO_4_·H_2_O (1.28), NaHCO_3_ (25), glucose (5.55), and indomethacin (3 × 10^–7^ mol/L). The solution was continuously bubbled with a 95% O_2_:5% CO_2_ mixture and maintained at 37 °C under a constant load of 1.0 g (Hugo Sachs Hebel–Messvorsatz (Tl-2)/HF-modem; Hugo Sachs Elektronik, Hugstetten, Germany) connected to a pen recorder (Kipp & Zonen BD41, Delft, Holland). During an equilibration period of 60 min, the Krebs buffer was changed every 10 min. The preparations were then continuously stimulated at 15–20 V at a frequency of 0.1 Hz for a duration of 0.5 ms, with rectangular-wave electrical pulses (Grass Stimulator S-88; Grass Instruments Co., Quincy, Massachusetts, USA). After about 30 min, the twitches were recurrent. Five min before RAMH administration, pyrilamine (1 × 10^–5^ mol/L concentration in organ bath) was added. The first cumulative concentration–response curve was determined for RAMH (10 nM – 10 mM) at increasing concentrations spaced by 3- or 3.3-fold. The second to the fourth curves were measured against increasing antagonist concentrations (incubation time 20 min). The pA_2_ values were calculated according to Arunlakshana and Schild.^37^ Statistical analysis was carried out with the Students’ *t* test. In all tests, a *p* < 0.05 was considered statistically significant. The pA_2_ values were compared with the affinity of thioperamide.

#### *Ex Vivo* Assay for Screening Histamine H_3_R Agonists: Determination of the −log EC_50_ Coefficient on Guinea Pig Ileum

Male guinea pigs, weighing 300–400 g, were euthanized by a blow to the neck. A 20–30 cm length of the distal ileum, apart from the terminal 5 cm, was rapidly removed and placed in phosphate buffer at room temperature (pH 7.4) containing (mM) NaCl (136.9), KCl (2.6), KH_2_PO_4_ (1.47), Na_2_HPO_4_ (9.58), and indomethacin (1 × 10^–6^ mol/L)). The intraluminal content was rinsed, and the isolated intestine was cut into 1.5–2 cm segments. The preparations were mounted between two platinum electrodes isotonically in a 20 mL organ bath filled with Krebs buffer: composition (mM) NaCl (118), KCl (5.6), MgSO_4_ (1.18), CaCl_2_ (2.5), NaH_2_PO_4_·H_2_O (1.28), NaHCO_3_ (25), glucose (5.55), and indomethacin (3 × 10^–7^ mol/L). The solution was continuously bubbled with a 95% O_2_:5% CO_2_ mixture and maintained at 37 °C under a constant load of 1.0 g (Hugo Sachs Hebel–Messvorsatz (Tl-2)/HF-modem; Hugo Sachs Elektronik, Hugstetten, Germany) connected to a pen recorder (Kipp & Zonen BD41, Delft, Holland). During an equilibration period of 60 min, Krebs buffer was changed every 10 min. Following this, the preparations were continuously stimulated at 15–20 V, at a frequency of 0.1 Hz for a duration of 0.5 ms, with rectangular-wave electrical pulses (Grass Stimulator S-88; Grass Instruments Co., Quincy, Massachusetts, USA). After about 30 min, twitches were recurrent. Thirty min before RAMH or tested agonist administration, famotidine (1 × 10^–5^ mol/L concentration in organ bath) and pyrilamine (1 × 10^–5^ mol/L concentration in organ bath) was added. Cumulative concentration–response curve was determined to RAMH or tested agonist (10 nM to 10 mM) at increasing concentrations spaced by 3- or 3.3-fold. The agonist potency is expressed as pD_2_ value (−log EC_50_) ± sem. The −log EC_50_ differences were not corrected since three successive curves were superimposable.

#### *Ex Vivo* Assay for Screening Histamine H_1_R Antagonists on Guinea Pig Ileum

Male guinea pigs, weighing 300–400 g, were euthanized by a blow to the neck. A 20–30 cm length of the distal ileum, apart from the terminal 5 cm, was rapidly removed and placed in phosphate buffer at room temperature (pH 7.4) containing (mM) NaCl (136.9), KCl (2.6), KH_2_PO_4_ (1.47), Na_2_HPO_4_ (9.58), and indomethacin (1 × 10^–6^ mol/L). The intraluminal content was rinsed, and the isolated intestine was cut into 1.5–2 cm segments. The preparations were mounted isotonically in a 20 mL organ bath filled with Krebs buffer: composition (mM) NaCl (118), KCl (5.6), MgSO_4_ (1.18), CaCl_2_ (2.5), NaH_2_PO_4_·H_2_O (1.28), NaHCO_3_ (25), glucose (5.55), and indomethacin (3 × 10^–7^ mol/L). Depending on the type of assay, Krebs buffer additionally contained or did not contain 0.05 μM of atropine. The solution was continuously bubbled with a 95% O_2_:5% CO_2_ mixture and maintained at 37 °C under a constant load of 0.5 g (Hugo Sachs Hebel–Messvorsatz (Tl-2)/HF-modem; Hugo Sachs Elektronik, Hugstetten, Germany) connected to a pen recorder (Kipp & Zonen BD41, Delft, Holland). During an equilibration period of 40 min, Krebs buffer was changed every 10 min. The first cumulative concentration–response curve was determined for histamine (10 nM to 10 mM) at increasing concentrations spaced by 3- or 3.3-fold. The second to the fourth (or fifth) curves were measured in the presence of an increasing concentrations of antagonist (incubation time 10 min). The pA_2_ values were calculated according to Arunlakshana and Schild.^37^ Statistical analysis was carried out with the Students’ *t* test. In all tests, a *p* < 0.05 was considered statistically significant. The pA_2_ values were compared with the affinity of pyrilamine.

#### *Ex Vivo* Assay for Screening M_2_R/M_3_R Antagonists on Guinea Pig Ileum

Male guinea pigs, weighing 300–400 g, were euthanized by a blow to the neck. A 20–30 cm length of the distal ileum, apart from the terminal 5 cm, was rapidly removed and placed in phosphate buffer at room temperature (pH 7.4) containing (mM) NaCl (136.9), KCl (2.6), KH_2_PO_4_ (1.47), Na_2_HPO_4_ (9.58), and indomethacin (1 × 10^–6^ mol/L)). The intraluminal content was rinsed, and the isolated intestine was cut into 1.5–2 cm segments. The preparations were mounted isotonically in a 20 mL organ bath filled with Tyrode’s buffer: composition (mM) NaCl (137), KCl (2.7), MgCl_2_·6H_2_O (1.0), CaCl_2_ (1.8), NaH_2_PO_4_·H_2_O (0.4), NaHCO_3_ (11.9), glucose (5.61), and indomethacin (3 × 10^–7^ mol/L). The solution was continuously bubbled with a 95% O_2_:5% CO_2_ mixture and maintained at 37 °C under a constant load of 0.5 g (Hugo Sachs Hebel–Messvorsatz (Tl-2)/HF-modem; Hugo Sachs Elektronik, Hugstetten, Germany) connected to a pen recorder (Kipp & Zonen BD41, Delft, Holland). During an equilibration period of 40 min, Tyrode’s buffer was changed every 10 min. The first cumulative concentration–response curve was determined for methacholine (10 nM to 3 mM) at increasing concentrations spaced by 3- or 3.3-fold. The second to the fourth (or fifth) curves were measured in the presence of an increasing concentration of antagonist (incubation time 20 min). The pA_2_ values were calculated according to Arunlakshana and Schild.^37^ Statistical analysis was carried out with the Students’ *t* test. In all tests, a *p* < 0.05 was considered statistically significant. The pA_2_ values were compared with the affinity of 4-DAMP.

#### Determination of hH_3_R Affinity

The radioligand displacement binding assay was performed in membrane fractions of HEK-293 cells stably expressing hH_3_R. Cell cultivation and membrane preparation were performed according to Kottke et al.^43^ For the radioligand displacement assay, radioactively labeled [^3^H]*N*^α^-methylhistamine was used at a final concentration of 2 nM (*K*_D_ = 3.08 nM). The total assay volume was set to 200 μL. The compounds were tested in several appropriate concentrations between 100 μM and 0.1 nM. Pipetting was partly done by Freedom Evo (Tecan). Pitolisant was used to determine nonspecific binding at a concentration of 10 μM. The membrane fraction (20 μg/well), test compounds, and radiolabeled ligand were incubated for 90 min at 25 °C while shaking. The bound radioligand was separated from free radioligand by filtration through GF/B filters pretreated with 0.3% (m/v) polyethylenimine using a cell harvester. Radioactivity was determined by liquid scintillation counting using a MicroBeta Trilux (PerkinElmer). The data were obtained in duplicates in at least three independent experiments. Nonspecific binding was subtracted from the raw data to calculate specific-binding values. The evaluation was performed with GraphPad Prism 6.1 (San Diego, CA, USA) using nonlinear regression (one-site competition with a logarithmic scale). The *K*_i_ values were calculated from the IC_50_ values using the Cheng–Prusoff equation.^44^ The statistical calculations were performed on −log *K*_i_. The mean values and 95% confidence intervals were transformed to nanomolar concentrations.

#### Cell Culture and Membrane Preparation

The culture was derived from CHO cells stably transfected with the genes of human variants of muscarinic receptors. These were purchased from Missouri S&T cDNA Resource Center (Rolla, MO, USA). Cell cultures and crude membranes were prepared as described previously.^45^ The cells were grown to confluence in 75 cm^2^ flasks in Dulbecco’s modified Eagle’s medium (DMEM) supplemented with 10% fetal bovine serum, and 2 million cells were subcultured to 100 mm Petri dishes. The medium was supplemented with 5 mM butyrate for the last 24 h of cultivation to increase receptor expression. Cells were detached by mild trypsinization on day 5 after subculture. Detached cells were washed twice in 50 mL of phosphate-buffered saline and 3 min centrifugation at 250*g*. Washed cells were suspended in 20 mL of ice-cold incubation medium (100 mM NaCl, 20 mM Na-HEPES, 10 mM MgCl_2_, pH = 7.4) supplemented with 10 mM EDTA and homogenized on ice by two 30 s strokes using a Polytron homogenizer (Ultra-Turrax; Janke & Kunkel GmbH & Co. KG, IKA-Labortechnik, Staufen, Germany) with a 30 s pause between strokes. Cell homogenates were centrifuged for 30 min at 30,000*g*. Supernatants were discarded, and pellets suspended in the fresh incubation medium, incubated on ice for 30 min, and centrifuged again. The resulting membrane pellets were kept at −80 °C until assay within a maximum of 10 weeks.

#### Determination of hM_1_R–hM_5_R Affinities

All radioligand binding experiments were optimized and carried out according to general guidelines.^39^ Briefly, membranes were incubated in 96-well plates at 30 °C in the incubation medium described above. Incubation volume was 400 μL or 800 μL for competition and saturation experiments with [^3^H]NMS, respectively. Approximately 30 μg of membrane proteins per sample were used. *N*-Methylscopolamine binding was measured directly in saturation experiments using six concentrations (30–1000 pM) of [^3^H]NMS during incubation for 1 h (M_2_R), 3 h (M_1_R, M_3_R, and M_4_R), or 5 h (M_5_R). For calculations of the equilibrium dissociation constant (*K*_D_), concentrations of free [^3^H]NMS were calculated by subtraction of bound radioactivity from total radioactivity in the sample and fitting eq 1 (Experimental Data analysis section). The binding of the tested ligands was determined in competition experiments with 100 pM [^3^H]NMS. The IC_50_ value was computed according to eq 2, and the inhibition constant *K*_i_ according to eq 3. Samples were first preincubated for 1 h with [^3^H]NMS. Then the tested compound was added and incubation continued for an additional 5 h. Nonspecific binding was determined in the presence of 1 μM unlabeled atropine. Incubations were terminated by filtration through Whatman GF/C glass fiber filters (Whatman) using a Brandel cell harvester (Brandel, Gaithersburg, MD, USA). The filters were dried in a microwave oven, and then solid scintillator Meltilex A was melted on filters (105 °C, 70 s) using a hot plate. The filters were cooled and counted in the Wallac Microbeta scintillation counter.

#### Intracellular Ca^2+^ Measurement

Intracellular Ca^2+^ level was taken as a functional response to ACh. Black 96-well plates were seeded with 12,000 CHO cells per well. After two days of cultivation in DMEM at 37 °C under a humidified atmosphere containing 5% CO_2_, cells were washed with Krebs-HEPES buffer (KHB) (composition: (mM) NaCl (138), KCl (4), CaCl_2_ (1.3), MgCl_2_ (1), NaH_2_PO_4_ (1.2), HEPES (20), glucose (10), pH adjusted to 7.4) KHB was loaded with 5 μM Fura-2 (Sigma-Aldrich) for 1 h. The cells were washed with fresh KHB and preincubated with tested compounds for 1 h. Then plates were placed in a Cytation 3 reader. The first basal level (fluorescence dual excitation at 340 and 380 nm, emission at 510 nm) was measured. Following this, ACh was added to the desired concentration (ranging from 10 pM to 100 μM) by instrument injectors, and fluorescence was measured immediately. Intracellular Ca^2+^ level was calculated as a ratio of 340 to 380 nm excitation fluorescence. The changes in intracellular Ca^2+^ level were compared as a fold increase over the basal level of the corresponding well.

### Experimental Data Analysis

#### Saturation of [^3^H]NMS Binding

The binding of [^3^H]NMS at various concentrations was measured. After subtraction of nonspecific binding and calculation of free radioligand concentration, eq 1 was fitted to the data:
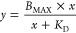
1where *y* is specific binding at free concentration *x*, *B*_MAX_ is maximum binding capacity, and *K*_D_ is the equilibrium dissociation constant of [^3^H]NMS.

#### Binding Parameters of Tested Compounds

Tested compounds are competitive antagonists of [^3^H]NMS binding. [^3^H]NMS binding was determined in the presence of tested compounds at various concentrations. After subtraction of nonspecific binding and normalization to binding in the absence of the tested compound, eq 2 was fitted to the data:
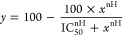
2where *y* is a specific radioligand binding at concentration *x* of the tested compound expressed as a percent of binding in its absence, IC_50_ is the concentration of tested compound inhibiting 50% of [^3^H]NMS binding, and nH is the Hill coefficient.
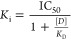
3where *K*_i_ is the inhibition constant of the tested compound, *K*_D_ is the equilibrium dissociation constant, and [*D*] is the concentration of [^3^H]NMS.

#### Concentration Response to Acetylcholine

The intracellular level of calcium at various concentrations of ACh was measured. After subtraction of background values and normalization to the level in the absence of ACh, eq 4 was fitted to the data:
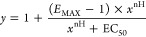
4where *y* is the normalized response at ACh concentration *x*, *E*_MAX_ is the maximal effect, EC_50_ is the concentration of ACh causing half-maximal effect, and nH is Hill coefficient. From a series of EC_50_ values of apparent inhibition constant K_B_ was calculated by fitting eq 5 to dose ratio (DR) induced by tested compound at a given concentration:
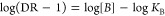
5where DR is ratio of EC_50_ in the presence of tested compound at concentration [*B*] to EC_50_ in its absence.

#### *In Silico* Studies

All tested ligands were prepared with the appropriate spatial configuration in the Maestro program (Schrödinger) and then charged at pH 7.4 ± 0.2 using the LigPrep program (Schrödinger Release 2017–1; Maestro-Schrödinger, LLC, New York).

All docking experiments were performed with the Glide program (Maestro-Schrödinger) with the SP level of calculation accuracy.^46^ For the docking purposes, grids centered on the **AF-DX 384** (Chart 1) ligand position, sized to dock ligands with length ≤25 Å, were prepared.

The *in silico* research used the M_2_R (PDB: 5ZKB) and M_4_R (PDB: 5DSG) complexes available in the Protein Data Base (PDB) and the previously published homology model of the histamine H_3_R.^28,47,48^ All proteins used in the study were prepared with the ProteinPrepare (PlayMolecule-Acellera) website.^49^ The structure of the ligand has a significant influence on the conformation of the amino acids in the binding site of GPCRs. Being aware of the large differences in the size of the studied ligands and compounds present in the crystallized complexes, it was decided to optimize the structure of the M_4_R and H_3_R based on the M_2_R complex with the compound **AF-DX 384** (PDB: 5ZKB) which demonstrated conformational changes in the aromatic amino acids Y104^3.33^ and Y426^7.39^ and W99^3.28^.^47^ Shifting these residues opens up the space needed for larger ligands to interact. This pattern was used to remodel the arrangement of the amino acids in the previously published homologous model of the histamine H_3_R^28^ and the structure of the M_4_R (PDB: 5DSG). The key differences in the position of the most important H_3_ amino acids are given in Figure S56.

The ligands **ADS1017** and **ADS10227** were docked to the receptors prepared in this way. Complexes showing consistent binding modes were optimized using the Refine Protein–Ligand Complex function with the Monte Carlo minimization method (Maestro-Schrödinger). Such optimized complexes were used for the final analyzes, and all tested ligands were docked to them. The final results were visualized using PyMol 0.99 rc6 software (DeLano Scientific LLC).

## Supporting Information Available

Chemical synthesis and data analysis. NMR spectra. Pharmacological assay results. *Ex vivo* assay for histamine H_1_R antagonists (variant with 0.05 μM atropine addition). *Ex vivo* assay for histamine H_1_R antagonists (variant without atropine addition). *Ex vivo* assay for histamine M_2_R/M_3_R antagonists. Decrease of contractility in electrically stimulated guinea pig ileum - determination of the −log EC_50_ coefficient. hH_3_R radioligand binding assay. hM_1_R-hM_5_R radioligand binding assay. Intracellular Ca^2+^ measurement. *In silico* assay results: Comparison of active sites of histamine and muscarinic receptors to H_3_R. Change of amino acid conformation in the H_3_R binding site on the example of the homology model. Two-dimensional map of interactions between the ADS1017 and ADS10227 ligands and H_3_, M_2_ and M_4_ receptors (PDF)
H3_ADS1017 (PDB)
H3_ADS10227 (PDB)
M2_ADS1017 (PDB)
M2_ADS10227 (PDB)
M4_ADS1017 (PDB)
M4_ADS10227 (PDB)
Molecular formula strings (CSV)

## Supplementary Material

cn1c00237_si_001.pdf

cn1c00237_si_002.pdb

cn1c00237_si_003.pdb

cn1c00237_si_004.pdb

cn1c00237_si_005.pdb

cn1c00237_si_006.pdb

cn1c00237_si_007.pdb

## Abbreviations Used

CNS: Central nervous system
ACh: acetylcholine
H_3_R: histamine H_3_ receptor
H_1_R: histamine H_1_ receptor
gpH_3_R: guinea pig histamine H_3_ receptor
gpH_1_R: guinea pig histamine H_1_ receptor
gpM_2_R/M_3_R: guinea pig muscarinic M_2_R/M_3_R
hH_3_R: human histamine H_3_ receptor
hM_1–5_: human muscarinic M_1–5_ receptors
GPCR: G protein-coupled receptor
ECL2: the second extracellular loop
RAMH: (*R*)-(−)-α-methylhistamine
4-DAMP: 1,1-dimethyl-4-diphenylacetoxypiperidinium iodide
NMR: nuclear magnetic resonance
[^3^H]NMS: *N*-[^3^H] methylscopolamine
rt: room temperature
EtOAc: ethyl acetate
DCM: dichloromethane
THF: tetrahydrofuran
Boc: *tert*-butoxycarbonyl
