# Supplementary material for: Guanidine Derivatives: How Simple Structural Modification of Histamine H3R Antagonists Has Led to the Discovery of Potent Muscarinic M2R/M4R Antagonists
Source: ACS Chem Neurosci. 2021 Jun 8;12(13):2503–19. doi: 10.1021/acschemneuro.1c00237 (PMC8291587; doi:10.1021/acschemneuro.1c00237)
Supplement: Supplementary file 1 — cn1c00237_si_001.pdf [file cn1c00237_si_001.pdf]

Supporting information

Guanidine derivatives: How simple structural modification of histamine H_3R antagonists has led to the discovery of potent muscarinic M_2R/M_4R antagonists.

Marek Staszewski[†*], Dominik Nelic[‡], Jakub Jończyk[§], Mariam Dubiel[#], Annika Frank[#], Holger Stark[#], Marek Bajda[§], Jan Jakubik[‡], Krzysztof Walczyński[†]

[†] Department of Synthesis and Technology of Drugs, Medical University of Lodz, ul. Muszyńskiego 1, 90-151 Łódź, Poland

[‡] Department of Neurochemistry, Institute of Physiology CAS, Videnska 1083, CZ142 20, Prague, Czech Republic

[§] Department of Physicochemical Drug Analysis, Faculty of Pharmacy, Jagiellonian University Medical College, Medyczna 9, 30–688 Kraków, Poland

[#] Institute of Pharmaceutical and Medicinal Chemistry, Heinrich Heine University Düsseldorf, Universitaetsstr. 1, Duesseldorf 40225, Germany

Table of contents

Entry	Section	Page
1	**Chemical synthesis and data analysis.**	S-2
2	**NMR spectra.**	S-21
3	**Pharmacological assay results.**	S-35
3.1	*Ex vivo* assay for histamine H_1R receptor antagonists (variant with 0.05 µM atropine addition).	S-35
3.2	*Ex vivo* assay for histamine H_1R receptor antagonists (variant without atropine addition).	S-39
3.3	*Ex vivo* assay for histamine M_2R/M_3R receptor antagonists.	S-43
3.4	Decrease of contractility in electrically-stimulated guinea pig ileum. - determination of the -logEC_{50} coefficient.	S-47
3.5	hH_3R radioligand binding assay.	S-51
3.6	hM_1R-hM_5R radioligand binding assay.	S-55
3.7	Intracellular Ca^{2+} measurement.	S-56
4	***In silico* assay results.**	S-57
4.1	Comparison of active sites of histamine and muscarinic receptors to H_3 receptor.	S-57
4.2	Change of amino acid conformation in the H_3 receptor binding site on the example of the homology model.	S-58
4.3	Two-dimensional map of interactions between the ADS1017 and ADS10227 ligands and H_3, M_2 and M_4 receptors	S-59

1. Chemical synthesis and data analysis

Preparation of (*E)*-1-bromomethyl-4-phenoxymethylcyclohexane (**4a**)

Phenol (1.49 g; $1.58 \cdot 10^{-2}$ mol) was added to a sodium (0.38 g; $1.65 \cdot 10^{-2}$ mol) dissolved in anhydrous ethanol (10 mL) and stirred for 30 minutes at room temperature. The previously prepared sodium phenoxide solution was added dropwise to a solution of *(E)*-1,4-Bis(bromomethyl)cyclohexane (**3a**) (4.27 g; $1.58 \cdot 10^{-2}$ mol) in 100 mL anhydrous ethanol heated to 65 °C. The reaction was stirred overnight at 80 °C. The solvent was removed under vacuum and the crude product was purified by column chromatography (hexane) to yield the pure product.

(E)-1-bromomethyl-4-phenoxymethylcyclohexane (**4a**): $C_{14}H_{19}BrO$. M=283.20. Transparent crystals. 37.86 % yield. R_F=0.31 (hexane). mp: 68.5-70.0 °C. ^1H NMR (600 MHz, *CDCl₃*) δ ppm 7.28-7.26 (m, $2H^{phenoxy.}$, $C(CHC\underline{H})_2CH$), 6.94-6.91 (m, $1H^{phenoxy.}$, $C(CHCH)_2C\underline{H}$), 6.89-6.88 (m, $2H^{phenoxy.}$, $C(C\underline{H}CH)_2CH$), 3.77 (d, 2H, $OC\underline{H}_2$, *J*=6.35 Hz), 3.30 (d, 2H, $BrC\underline{H}_2$, *J*=6.35 Hz), 1.97-1.95 (m, $4H^{cyclohexyl.}$, $C\underline{H}_2$), 1.77-1.74 (m, $1H^{cyclohexyl.}$, $OCH_2C\underline{H}$), 1.66-1.63 (m, $1H^{cyclohexyl.}$, $BrCH_2C\underline{H}$), 1.15-1.05 (m, $4H^{cyclohexyl.}$, $C\underline{H}_2$). ^{13}C NMR (150.95 MHz, *CDCl₃*) δ ppm 159.11 ($1C^{quat./phenoxy.}$, $\underline{C}O$), 129.39 ($2C^{phenoxy.}$, $C(CH\underline{C}H)_2CH$), 120.47 ($1C^{phenoxy.}$, $C(CHCH)_2\underline{C}H$), 114.42 ($2C^{phenoxy.}$, $C(\underline{C}HCH)_2CH$), 72.86 (1C, $O\underline{C}H_2$), 40.36 (1C, $Br\underline{C}H_2$), 40.09 ($1C^{cyclohexyl.}$, $BrCH_2\underline{C}H$), 37.48 ($1C^{cyclohexyl.}$, $OCH_2\underline{C}H$), 31.01 ($2C^{cyclohexyl.}$, $\underline{C}H_2$), 29.19 ($2C^{cyclohexyl.}$, $\underline{C}H_2$).

Preparation of (*Z*)-1-bromomethyl-4-phenoxymethylcyclohexane (**4b**)

Phenol (1.06 g; $1.13 \cdot 10^{-2}$ mol) was added to a sodium (0.26 g; $1.13 \cdot 10^{-2}$ mol) dissolved in anhydrous ethanol (10 mL) and stirred for 30 minutes at room temperature. The previously prepared sodium phenoxide solution was added dropwise to a solution of (*Z*)-1,4-Bis(bromomethyl)cyclohexane (**3b**) (3.04 g; $1.12 \cdot 10^{-2}$ mol) in 120 mL anhydrous ethanol heated to 65 °C. The reaction was stirred overnight at 80 °C. The solvent was removed under vacuum and the crude product was purified by column chromatography (hexane/DCM 8:2) to yield the pure product.

(*Z*)-1-bromomethyl-4-phenoxymethylcyclohexane (**4b**): $C_{14}H_{19}BrO$. M=283.20. Colorless oil. 22.11 % yield. R_F=0.67 (hexane/DCM 8:2). ^1H NMR (600 MHz, *CDCl₃*) δ ppm 7.28-7.27 (m, $2H^{phenoxy.}$, $C(CHC\underline{H})_2CH$), 6.94-6.91 (m , $1H^{phenoxy.}$, $C(CHCH)_2C\underline{H}$), 6.89-6.88 (m, $2H^{phenoxy.}$, $C(C\underline{H}CH)_2CH$), 3.86 (d, 2H, $OC\underline{H}_2$), 3.40 (d, 2H, $BrC\underline{H}_2$), 2.07-1.98 (m, $1H^{cyclohexyl.}$, $OCH_2C\underline{H}$), 1.94-1.85 (m, $1H^{cyclohexyl.}$, $BrCH_2C\underline{H}$), 1.70-1.61 (m, $4H^{cyclohexyl.}$, $C\underline{H}_2$), 1.59-1.50 (m, $4H^{cyclohexyl.}$, $C\underline{H}_2$). ^{13}C NMR (150.95 MHz, *CDCl₃*) δ ppm 159.19 ($1C^{quat./phenoxy.}$, $\underline{C}O$), 129.42 ($2C^{phenoxy.}$, $C(CH\underline{C}H)_2CH$), 120.57 ($1C^{phenoxy.}$, $C(CHCH)_2\underline{C}H$), 114.58 ($2C^{phenoxy.}$, $C(\underline{C}HCH)_2CH$), 70.60 (1C, $O\underline{C}H_2$), 38.53 (1C, $Br\underline{C}H_2$), 38.01 ($1C^{cyclohexyl.}$, $BrCH_2\underline{C}H$), 34.84 ($1C^{cyclohexyl.}$, $OCH_2\underline{C}H$), 27.38 ($2C^{cyclohexyl.}$, $\underline{C}H_2$), 25.43 ($2C^{cyclohexyl.}$, $\underline{C}H_2$).

Preparation of (*E*)-1-((4-(phenoxymethyl)cyclohexyl)methyl)piperazine (**5a**)

(*E)*-1-bromomethyl-4-phenoxymethylcyclohexane (**4a**) (0.92 g; $3.25 \cdot 10^{-3}$ mol) in 30 mL THF was added dropwise to a solution of piperazine (1.40 g; $1.62 \cdot 10^{-2}$ mol) in 40 mL THF heated to 60 °C. The reaction was stirred overnight at 66 °C. The solvent was removed under vacuum and the residue was diluted by 100 mL water, alkalized with 5 % NaOH solution and extracted 3x50 mL with DCM. The combined organic phases were dried over anhydrous Na_2SO_4. The solvent was removed under vacuum and the crude product was purified by column chromatography (DCM/MeOH/25% NH_3 aq. 89:10:1) to yield the pure product.

(*E)*-1-((4-(phenoxymethyl)cyclohexyl)methyl)piperazine (**5a**): $C_{18}H_{28}N_2O$. M=288.43. White solid. 74.79 % yield. R_F=0.67 (DCM/MeOH/25% NH_3 aq. 89:10:1). mp: 98.5-99.5 °C. ^1H NMR (600 MHz, *CDCl₃*) δ ppm 7.28-7.25 (m, $2H^{phenoxy.}$, $C(CHC\underline{H})_2CH$), 6.93-6.88 (m, $3H^{phenoxy.}$, $C(C\underline{H}CH)_2C\underline{H}$), 3.76 (d, 2H, $OC\underline{H}_2$, *J*=6.32 Hz), 2.89-2.87 (m, $4H^{piperazine}$), 2.36 (br, , $4H^{piperazine}$), 2.13 (d, 2H, $C\underline{H}_2N^{piperazine}$, *J* = 7.07 Hz), 1.93-1.87 (m, $4H^{cyclohexyl.}$, $C\underline{H}_2$), 1.81-1.71 (m, $1H^{cyclohexyl.}$, $C\underline{H}$), 1.65 (s, 1H, $N\underline{H}$, *), 1.54-1.45 (m, $1H^{cyclohexyl.}$, $C\underline{H}$), 1.09-1.02 (m, $2H^{cyclohexyl.}$, $C\underline{H}_2$), 0.97-0.90 (m, $2H^{cyclohexyl.}$, $C\underline{H}_2$). ^{13}C NMR (150.95 MHz, *CDCl₃*) δ ppm 159.16 ($1C^{quat./phenoxy.}$, $\underline{C}O$), 129.31 ($2C^{phenoxy.}$, $C(CH\underline{C}H)_2CH$), 120.33 ($1C^{phenoxy.}$, $C(CHCH)_2\underline{C}H$), 114.39 ($2C^{phenoxy.}$, $C(\underline{C}HCH)_2CH$), 73.21(1C, $O\underline{C}H_2$), 65.97 (1C, $\underline{C}H_2N^{piperazine}$), 54.23 ($2C^{piperazine}$), 45.55 ($2C^{piperazine}$), 37.96 ($1C^{cyclohexyl.}$, $OCH_2\underline{C}H$), 34.87 ($1C^{cyclohexyl.}$, $NCH_2C\underline{H}$), 31.06 ($2C^{cyclohexyl.}$, $\underline{C}H_2$), 29.47 ($2C^{cyclohexyl.}$, $\underline{C}H_2$).

Preparation of (*Z*)-1-((4-(phenoxymethyl)cyclohexyl)methyl)piperazine (**5b**)

(*Z*)-1-bromomethyl-4-phenoxymethylcyclohexane (**4b**) (0.69 g; $2.44\cdot10^{-3}$ mol) in 20 mL THF was added to a solution of piperazine (1.04 g; $1.21\cdot10^{-2}$ mol) in 30 mL THF heated to 60 °C. The reaction was stirred overnight at 66 °C. The solvent was removed under vacuum and the residue was diluted by 60 mL water, alkalized with 5 % NaOH solution and extracted 3x50 mL with DCM. The combined organic phases were dried over anhydrous Na_2SO_4. The solvent was removed under vacuum and the crude product was purified by column chromatography (DCM/MeOH/25% NH_3 aq. 139:10:1) to yield the pure product.

(*Z*)-1-((4-(phenoxymethyl)cyclohexyl)methyl)piperazine (**5b**): $C_{18}H_{28}N_2O$. M=288.43. White solid. 57.11 % yield. R_F=0.48 (DCM/MeOH/25% NH_3 aq. 139:10:1). mp: 58.1-59.3 °C. ^{1}H NMR (600 MHz, *CDCl$_3$*) δ ppm 7.27-7.25 (m, $2H^{phenoxy.}$, $C(CHC\underline{H})_2CH$), 6.93-6.87 (m, $3H^{phenoxy.}$, $C(C\underline{H}CH)_2C\underline{H}$), 3.86 (d, 2H, $OC\underline{H}_2$), 2.89-2.87 (m, $4H^{piperazine}$), 2.38 (br, $4H^{piperazine}$), 2.21 (d, 2H, $C\underline{H}_2N^{piperazine}$), 2.04 (s, 1H, $N\underline{H}$, *), 2.02-1.96 (m, $1H^{cyclohexyl.}$, $OCH_2C\underline{H}$), 1.77-1.73 (m, $1H^{cyclohexyl.}$, $C\underline{H}CH_2N^{piperazine}$), 1.62-1.54 (m, $4H^{cyclohexyl.}$, $C\underline{H}_2$), 1.53-1.48 (m, $2H^{cyclohexyl.}$, $C\underline{H}_2$), 1.41-1.37 (m, $2H^{cyclohexyl.}$, $C\underline{H}_2$). ^{13}C NMR (150.95 MHz, *CDCl$_3$*) δ ppm 159.34 ($1C^{quat./phenoxy.}$, $\underline{C}O$), 129.37 ($2C^{phenoxy.}$, $C(CH\underline{C}H)_2CH$), 120.45 ($1C^{phenoxy.}$, $C(CHCH)_2\underline{C}H$), 114.64 ($2C^{phenoxy.}$, $C(\underline{C}HCH)_2CH$), 71.22 (1C, $O\underline{C}H_2$), 63.22 (1C, $\underline{C}H_2N^{piperazine}$), 54.99 ($2C^{piperazine}$), 46.12 ($2C^{piperazine}$), 35.42 ($1C^{cyclohexyl.}$, $OCH_2\underline{C}H$), 32.17 ($1C^{cyclohexyl.}$, $NCH_2C\underline{H}$), 27.31 ($2C^{cyclohexyl.}$, $\underline{C}H_2$), 25.75 ($2C^{cyclohexyl.}$, $\underline{C}H_2$).

Preparation of (*E)*-4-(4-((4-(phenoxymethyl)cyclohexyl)methyl)piperazin-1-yl)butanenitrile (**6a**)

Potassium carbonate (1.37 g; $9.91\cdot10^{-3}$ mol) and 4-bromobutyronitrile (0.39 g; $2.63\cdot10^{-3}$ mol) was added to a solution of (*E)*-1-((4-(phenoxymethyl)cyclohexyl)methyl)piperazine (**5a**) (0.72 g; $2.49\cdot10^{-3}$ mol) in 20 mL acetonitrile. The reaction was stirred overnight at 80 °C, then filtered. The precipitate was discarded. The solvent was removed under vacuum and the crude product was purified by column chromatography (EtOAc/MeOH/Triethylamine 89:10:1) to yield the pure product.

(*E)*-4-(4-((4-(phenoxymethyl)cyclohexyl)methyl)piperazin-1-yl)butanenitrile (**6a**): $C_{22}H_{33}N_3O$. M=355.52. White solid. 99.21 % yield. R_F=0.86 (EtOAc/MeOH/Triethylamine 89:10:1). mp: 66.5-68.5 °C. ^{1}H NMR (600 MHz, *CDCl$_3$*) δ ppm 7.28-7.25 (m, $2H^{phenoxy.}$, $C(CHC\underline{H})_2CH$), 6.93-6.91 (m, $1H^{phenoxy.}$, $C(CHCH)_2C\underline{H}$), 6.89-6.88 (m, $2H^{phenoxy.}$, $C(C\underline{H}CH)_2CH$), 3.76 (d, 2H, $OC\underline{H}_2$, *J*=6.34 Hz), 2.49-2.39 (m, 12H: $N^{piperazine}C\underline{H}_2CH_2C\underline{H}_2CN$; $8H^{piperazine}$), 2.16 (d, 2H, $CHC\underline{H}_2N^{piperazine}$, *J*=7.09 Hz), 1.93-1.91 (m, $2H^{cyclohexyl.}$, $C\underline{H}_2$), 1.88-1.86 (m, $2H^{cyclohexyl.}$, $C\underline{H}_2$), 1.82 (qt, 2H, $CH_2C\underline{H}_2CH_2$, *J*=7.00 Hz), 1.78-1.74 (m, $1H^{cyclohexyl.}$, $OCH_2C\underline{H}$), 1.51-1.46 (m, $1H^{cyclohexyl.}$, $C\underline{H}CH_2N^{piperazine}$), 1.09-1.03 (m, $2H^{cyclohexyl.}$, $C\underline{H}_2$), 0.97-0.91 (m, $2H^{cyclohexyl.}$, $C\underline{H}_2$). ^{13}C NMR (150.95 MHz, *CDCl$_3$*) δ ppm 159.93 ($1C^{quat./phenoxy.}$, $\underline{C}O$), 129.34 ($2C^{phenoxy.}$, $C(CH\underline{C}H)_2CH$), 120.36 ($1C^{phenoxy.}$, $C(CHCH)_2\underline{C}H$), 119.78 (1C, $\underline{C}N$), 114.42 ($2C^{phenoxy.}$, $C(\underline{C}HCH)_2CH$), 73.25 (1C, $O\underline{C}H_2$), 65.47 (1C, $CH\underline{C}H_2N^{piperazine}$), 56.31(1C, $N^{piperazine}\underline{C}H_2CH_2$), 53.56 ($2C^{piperazine}$), 53.01 ($2C^{piperazine}$), 37.97 ($1C^{cyclohexyl.}$, $OCH_2\underline{C}H$), 35.08 ($1C^{cyclohexyl.}$, $\underline{C}HCH_2N^{piperazine}$), 31.13 ($2C^{cyclohexyl.}$, $\underline{C}H_2$), 29.50 ($2C^{cyclohexyl.}$, $\underline{C}H_2$), 22.71 (1C, $CH_2\underline{C}H_2CH_2$), 14.90 (1C, $\underline{C}H_2CN$).

Preparation of (*Z*)-4-(4-((4-(phenoxymethyl)cyclohexyl)methyl)piperazin-1-yl)butanenitrile (**6b**)

Potassium carbonate (1.20 g; $8.68\cdot10^{-3}$ mol) and 4-bromobutyronitrile (0.34 g; $2.29\cdot10^{-3}$ mol) was added to a solution of (*Z*)-1-((4-(phenoxymethyl)cyclohexyl)methyl)piperazine (**5b**) (0.63 g; $2.18\cdot10^{-5}$ mol) in 20 mL acetonitrile. The reaction was stirred overnight at 80 °C, then filtered. The precipitate was discarded. The solvent was removed under vacuum and the crude product was purified by column chromatography (EtOAc/MeOH/Triethylamine 139:10:1) to yield the pure product.

(*Z*)-4-(4-((4-(phenoxymethyl)cyclohexyl)methyl)piperazin-1-yl)butanenitrile (**6b**): $C_{22}H_{33}N_3O$. M=355.52. White solid. 92.42 % yield. R_F=0.51 (EtOAc/MeOH/Triethylamine 139:10:1). mp: 72.5-73.7 °C. ^{1}H NMR (600 MHz, *CDCl$_3$*) δ ppm 7.28-7.25 (m, $2H^{phenoxy.}$, $C(CHC\underline{H})_2CH$), 6.93-6.88 (m, $3H^{phenoxy.}$, $C(C\underline{H}CH)_2C\underline{H}$), 3.85 (d, 2H, $OC\underline{H}_2$, *J*=6.94 Hz), 2.45-2.39 (m, 12H: $8H^{piperazine}$, $C\underline{H}_2CH_2C\underline{H}_2CN$), 2.22 (d, 2H, $CHC\underline{H}_2N^{piperazine}$, *J*=7.36 Hz), 2.00-1.97 (m, $1H^{cyclohexyl.}$, $OCH_2C\underline{H}$), 1.81 (qt, 2H, $CH_2C\underline{H}_2CH_2$, *J*=7.00 Hz), 1.75-1.72 (m, $1H^{cyclohexyl.}$, $C\underline{H}CH_2N^{piperazine}$), 1.63-1.47 (m, $6H^{cyclohexyl.}$, $C\underline{H}_2$), 1.41-1.36 (m, $2H^{cyclohexyl.}$, $C\underline{H}_2$). ^{13}C NMR (150.95 MHz, *CDCl$_3$*) δ ppm 159.29 ($1C^{quat./phenoxy.}$, $\underline{C}O$), 129.36 ($2C^{phenoxy.}$: $C(CH\underline{C}H)_2CH$), 120.43 ($1C^{phenoxy.}$, $C(CHCH)_2\underline{C}H$), 119.76 ($1C^{quat.}$, $\underline{C}N$), 114.59 ($2C^{phenoxy.}$, $C(\underline{C}HCH)_2CH$), 71.16 (1C, $O\underline{C}H_2$), 62.51 (1C, $CH\underline{C}H_2N^{piperazine}$), 56.36 (1C,$CH_2\underline{C}H_2N^{piperazine}$), 53.63 ($2C^{piperazine}$), 53.19 ($2C^{piperazine}$), 35.38 ($1C^{cyclohexyl.}$, $OCH_2\underline{C}H$), 32.32 ($1C^{cyclohexyl.}$, $\underline{C}HCH_2N^{piperazine}$), 27.29 ($2C^{cyclohexyl.}$, $\underline{C}H_2$), 25.72 ($2C^{cyclohexyl.}$, $\underline{C}H_2$), 22.82 (1C, $CH_2\underline{C}H_2CH_2$), 14.92 (1C, $\underline{C}H_2CN$).

Preparation of (*E)*-4-(4-((4-(phenoxymethyl)cyclohexyl)methyl)piperazin-1-yl)butan-1-amine (**7a**)

$LiAlH_4$ (0.42 g; $1.11\cdot10^{-2}$ mol) was added to a solution of (*E)*-4-(4-((4-(phenoxymethyl)cyclohexyl)methyl)piperazin-1-yl)butanenitrile (**6a**) (0.85 g; $2.39\cdot10^{-3}$ mol) in 40 mL THF. The reaction was stirred overnight at room temperature, then the mixture was quenched by dropwise addition of water (16 eq.) and 10 % NaOH solution (16 eq.) stirred for two hours, then filtered. The precipitate was discarded. The organic layer was dried over Na_2SO_4, then the solvent was removed under vacuum and the crude product was purified by column chromatography (DCM/MeOH/25% NH_3 aq. 49:10:1) to yield the pure product.

(*E)*-4-(4-((4-(phenoxymethyl)cyclohexyl)methyl)piperazin-1-yl)butan-1-amine (**7a**): $C_{22}H_{37}N_3O$. M=359.55. White solid. 58.17 % yield. R_F=0.35 (DCM/MeOH/25% NH_3 aq. 49:10:1). mp: 95.5-96.5 °C. ^1H NMR (600 MHz, *CDCl₃*) δ ppm 7.27-7.24 (m, $2H^{phenoxy.}$, $C(CHC\underline{H})_2CH$), 6.93-6.85 (m, $3H^{phenoxy.}$, $C(C\underline{H}CH)_2C\underline{H}$), 3.76 (d, 2H, $OC\underline{H}_2$, J=6.32 Hz), 2.70 (t, 2H, $C\underline{H}_2NH_2$, J=6.91 Hz), 2.48-2.32 (m, 10H: $8H^{piperazine}$, $CH_2\underline{C}H_2N^{piperazine}$), 2.15 (d, 2H, $CHC\underline{H}_2N^{piperazine}$, J=7.08 Hz), 1.93-1.90 (m, $2H^{cyclohexyl.}$, $C\underline{H}_2$), 1.88-1.86 (m, $2H^{cyclohexyl.}$, $C\underline{H}_2$), 1.79-1.72 (m, $1H^{cyclohexyl.}$, $C\underline{H}$), 1.55-1.27 (m, 7H: $1H^{cyclohexyl.}$, $C\underline{H}$; $CH_2C\underline{H}_2CH_2$, $N\underline{H}_2$), 1.09-1.02 (m, $2H^{cyclohexyl.}$, $C\underline{H}_2$), 0.97-0.90 (m, $2H^{cyclohexyl.}$, $C\underline{H}_2$). ^{13}C NMR (150.95 MHz, *CDCl₃*) δ ppm 159.22 ($1C^{quat./phenoxy.}$, $\underline{C}O$), 129.30 ($2C^{phenoxy.}$, $C(CH\underline{C}H)_2CH$), 120.33 ($1C^{phenoxy.}$, $C(CHCH)_2\underline{C}H$), 114.44 ($2C^{phenoxy.}$, $C(\underline{C}HCH)_2CH$), 73.29 (1C, $O\underline{C}H_2$), 65.57 (1C, $CH\underline{C}H_2N^{piperazine}$), 58.56 (1C,$CH_2\underline{C}H_2N^{piperazine}$), 53.69 ($2C^{piperazine}$), 53.28 ($2C^{piperazine}$), 42.13 (1C, $\underline{C}H_2NH_2$), 38.01 ($1C^{cyclohexyl.}$, $OCH_2\underline{C}H$), 35.16 ($1C^{cyclohexyl.}$, $\underline{C}HCH_2N^{piperazine}$), 31.86 (1C, $CH_2\underline{C}H_2CH_2$), 31.16 ($2C^{cyclohexyl.}$, $\underline{C}H_2$), 29.53 ($2C^{cyclohexyl.}$, $\underline{C}H_2$), 24.30 (1C, $CH_2\underline{C}H_2CH_2$).

Preparation of (*Z*)-4-(4-((4-(phenoxymethyl)cyclohexyl)methyl)piperazin-1-yl)butan-1-amine (**7b**)

$LiAlH_4$ (0.35 g; $9.22\cdot10^{-3}$ mol) was added to a solution of (*Z*)-4-(4-((4-(phenoxymethyl)cyclohexyl)methyl)piperazin-1-yl)butanenitrile (**6b**) (0.75 g; $2.11\cdot10^{-3}$ mol) in 40 mL anhydrous diethyl ether. The reaction was stirred overnight at room temperature, then the mixture was quenched by dropwise addition of water (16 eq.) and 10 % NaOH solution (16 eq.) stirred for two hours, then filtered. The precipitate was discarded. The organic layer was dried over Na_2SO_4, then the solvent was removed under vacuum and the crude product was purified by column chromatography (DCM/MeOH/25% NH_3 aq. 49:10:1) to yield the pure product.

(*Z*)-4-(4-((4-(phenoxymethyl)cyclohexyl)methyl)piperazin-1-yl)butan-1-amine (**7b**): $C_{22}H_{37}N_3O$. M=359.55. Colorless oil. 92.18 % yield. R_F=0.54 (DCM/MeOH/25% NH_3 aq. 49:10:1). ^1H NMR (600 MHz, *CDCl₃*) δ ppm 7.28-7.25 (m, $2H^{phenoxy.}$, $C(CHC\underline{H})_2CH$), 6.93-6.88 (m, $3H^{phenoxy.}$, $C(C\underline{H}CH)_2C\underline{H}$), 3.85 (d, 2H, $OC\underline{H}_2$, J=6.95 Hz), 2.70 (t, 2H, $C\underline{H}_2NH_2$, J=6.90 Hz), 2.44 (br, $8H^{piperazine}$), 2.33 (t, 2H, $CH_2C\underline{H}_2N^{piperazine}$), 2.22 (d, 2H, $CHC\underline{H}_2N^{piperazine}$, J=7.34 Hz), 2.03-1.95 (m, $1H^{cyclohexyl.}$, $OCH_2C\underline{H}$), 1.79-1.69 (m, $1H^{cyclohexyl.}$, $C\underline{H}CH_2N^{piperazine}$), 1.63-1.20 (m, 14H: $8H^{cyclohexyl.}$, $CH_2C\underline{H}_2CH_2$, $N\underline{H}_2$). ^{13}C NMR (150.95 MHz, *CDCl₃*) δ ppm 159.31 ($1C^{quat./phenoxy.}$, $\underline{C}O$), 129.36 ($2C^{phenoxy.}$, $C(CH\underline{C}H)_2CH$), 120.42 ($1C^{phenoxy.}$, $C(CHCH)_2\underline{C}H$), 114.59 ($2C^{phenoxy.}$, $C(\underline{C}HCH)_2CH$), 71.17 (1C, $O\underline{C}H_2$), 62.62 (1C, $CH\underline{C}H_2N^{piperazine}$), 58.61 (1C, $CH_2\underline{C}H_2N^{piperazine}$), 55.73 ($2C^{piperazine}$), 53.79 ($2C^{piperazine}$), 42.19 (1C, $\underline{C}H_2NH_2$), 35.37 ($1C^{cyclohexyl.}$, $OCH_2\underline{C}H$), 32.34 ($1C^{cyclohexyl.}$, $\underline{C}HCH_2N^{piperazine}$), 31.93 (1C, $CH_2\underline{C}H_2CH_2$) 27.32 ($2C^{cyclohexyl.}$, $\underline{C}H_2$), 25.74 ($2C^{cyclohexyl.}$, $\underline{C}H_2$), 24.36 (1C, $CH_2\underline{C}H_2CH_2$).

Preparation of (*E)-N*-(4-(4-((4-(phenoxymethyl)cyclohexyl)methyl)piperazin-1-yl)butyl)benzamide (**8a**)

Benzoyl chloride (0.26 g; $1.84\cdot10^{-3}$ mol) in 10mL DCM was added dropwise to a solution of (*E)*-4-(4-((4-(phenoxymethyl)cyclohexyl)methyl)piperazin-1-yl)butan-1-amine (**7a**) (0.60 g; $1.67\cdot10^{-3}$ mol) and triethylamine (0.67g $6.67\cdot10^{-3}$ mol) in 20 mL DCM. The reaction was stirred for three hours at room temperature. The mixture was washed 3-times with 20 mL water and dried over Na_2SO_4. The solvent was removed under vacuum and the crude product was purified by column chromatography (DCM/MeOH/25% NH_3 aq. 189:10:1) to yield the pure product.

(*E)-N*-(4-(4-((4-(phenoxymethyl)cyclohexyl)methyl)piperazin-1-yl)butyl)benzamide (**8a**): $C_{29}H_{41}N_3O_2$. M=463.65. White solid. 76.62 % yield. R_F=0.66 (DCM/MeOH/25% NH_3 aq. 189:10:1). mp: 121.0-123.0 °C. ^1H NMR (600 MHz, *CDCl₃*) δ ppm 7.76-7.74 (m, $2H^{arom.}$, $C(C\underline{H}CH)_2CH$), 7.48-7.46 (m, $1H^{arom.}$, $C(CHCH)_2C\underline{H}$), 7.42-7.39 (m, $2H^{arom.}$, $C(CHC\underline{H})_2CH$), 7.27-7.25 (m, $2H^{phenoxy.}$, $C(CHC\underline{H})_2CH$), 6.92-6.88 (m, $3H^{phenoxy.}$,

$C(C\underline{H}CH)_2C\underline{H}$), 6.79 (s, 1H, N$\underline{H}$), 3.76 (d, 2H, $OC\underline{H}_2$, J=6.33 Hz), 3.47-3.44 (m, 2H, $C\underline{H}_2NH$), 2.44 (m, $8H^{piperazine}$), 2.38 (t, 2H, $CH_2C\underline{H}_2N^{piperazine}$), 2.11 (d, 2H, $CHC\underline{H}_2N^{piperazine}$, J = 7.08Hz), 1.92-1.90 (m, $2H^{cyclohexyl.}$, $C\underline{H}_2$), 1.87-1.84 (m, $2H^{cyclohexyl.}$, $C\underline{H}_2$), 1.78-1.72 (m, $1H^{cyclohexyl.}$, $C\underline{H}$), 1.66 (qt, 2H, $CH_2C\underline{H}_2CH_2$), 1.61 (qt, 2H, $CH_2C\underline{H}_2CH_2$), 1.49-1.42 (m, $1H^{cyclohexyl.}$, $C\underline{H}$), 1.07-1.02 (m, $2H^{cyclohexyl.}$, $C\underline{H}_2$), 0.96-0.89 (m, $2H^{cyclohexyl.}$, $C\underline{H}_2$). ^{13}C NMR (150.95 MHz, *$CDCl_3$)* δ ppm 167.67 ($1C^{quat.}$, $\underline{C}$=O), 159.24 ($1C^{quat./phenoxy.}$, $\underline{C}O$), 135.03 ($1C^{quat./arom.}$, $\underline{C}C(O)$), 131.15 ($1C^{arom.}$, $C(CHCH)_2\underline{C}H$), 129.33 ($2C^{phenoxy.}$, $C(CH\underline{C}H)_2CH$), 128.42 ($2C^{arom.}$, $C(CH\underline{C}H)_2CH$), 126.92 ($2C^{arom.}$, $C(\underline{C}HCH)_2CH$), 120.36 ($1C^{phenoxy.}$, $C(CHCH)_2\underline{C}H$), 114.46 ($2C^{phenoxy.}$, $C(\underline{C}HCH)_2CH$), 73.30 (1C, $O\underline{C}H_2$), 65.51 (1C, $CH\underline{C}H_2N^{piperazine}$), 57.99 (1C, $CH_2\underline{C}H_2N^{piperazine}$), 53.49 ($2C^{piperazine}$), 53.24 ($2C^{piperazine}$), 39.91 (1C, $\underline{C}H_2NH$), 38.02 ($1C^{cyclohexyl.}$, $OCH_2\underline{C}H$), 35.16 ($1C^{cyclohexyl.}$, $\underline{C}HCH_2N^{piperazine}$), 31.16 ($2C^{cyclohexyl.}$, $\underline{C}H_2$), 29.53 ($2C^{cyclohexyl.}$, $\underline{C}H_2$), 27.43 (1C, $CH_2\underline{C}H_2CH_2$), 24.47 (1C, $CH_2\underline{C}H_2CH_2$).

Preparation of (*E)-N*-(4-(4-((4-(phenoxymethyl)cyclohexyl)methyl)piperazin-1-yl)butyl)-4-(trifluoromethyl)benzamide (**8b**)

4-(Trifluoromethyl)benzoyl chloride (0.16 g; $7.67 \cdot 10^{-4}$ mol) in 5 mL DCM was added dropwise to a solution of (*E*)-4-(4-((4-(phenoxymethyl)cyclohexyl)methyl)piperazin-1-yl)butan-1-amine (**7a**) (0.25 g; $6.95 \cdot 10^{-4}$ mol) and triethylamine (0.28 g; $2.78 \cdot 10^{-3}$ mol) in 10 mL DCM. The reaction was stirred for three hours at room temperature. The mixture was washed 3-times with 10 mL water and dried over Na_2SO_4. The solvent was removed under vacuum and the crude product was purified by column chromatography (DCM/MeOH/25% NH_3 aq. 189:10:1) to yield the pure product.

(*E)-N*-(4-(4-((4-(phenoxymethyl)cyclohexyl)methyl)piperazin-1-yl)butyl)-4-(trifluoromethyl)benzamide (**8b**): $C_{30}H_{40}F_3N_3O_2$. M=513.65. White solid. 99.37 % yield. R_F=0.52 (DCM/MeOH/25% NH_3 aq. 189:10:1). mp: 143.0-145.5 °C. ^1H NMR (600 MHz, *$CDCl_3$)* δ ppm 7.86-7.85 (m, $2H^{arom.}$, $C(C\underline{H}CH)_2CCF_3$), 7.69-7.68 (m, $2H^{arom.}$, $C(CHC\underline{H})_2CCF_3$), 7.28-7.25 (m, $2H^{phenoxy.}$, $C(CHC\underline{H})_2CH$), 7.03 (br, 1H, N$\underline{H}$), 6.95-6.85 (m, $3H^{phenoxy.}$, $C(C\underline{H}CH)_2C\underline{H}$), 3.76 (d, 2H, $OC\underline{H}_2$, J=6.31 Hz), 3.49-3.46 (m, 2H, $C\underline{H}_2NH$), 2.63-2.19 (m, 10H: $8H^{piperazine}$, $CH_2C\underline{H}_2N^{piperazine}$), 2.08 (d, 2H, $CHC\underline{H}_2N^{piperazine}$, J=7.06 Hz), 1.92-1.90 (m, $2H^{cyclohexyl.}$, $C\underline{H}_2$), 1.85-1.84 (m, $2H^{cyclohexyl.}$, $C\underline{H}_2$), 1.80-1.66 (m, 3H: $1H^{cyclohexyl.}$, $C\underline{H}$; $CH_2C\underline{H}_2CH_2$), 1.66-1.59 (m, 2H, $CH_2C\underline{H}_2CH_2$), 1.48-1.39 (m, $1H^{cyclohexyl.}$, $C\underline{H}$), 1.09-1.02 (m, $2H^{cyclohexyl.}$, $C\underline{H}_2$), 0.95-0.89 (m, $2H^{cyclohexyl.}$, $C\underline{H}_2$). ^{13}C NMR (150.95 MHz, *$CDCl_3$)* δ ppm 166.55 ($1C^{quat.}$, $\underline{C}$=O), 159.33 ($1C^{quat./phenoxy.}$, $\underline{C}O$), 138.59 ($1C^{quat./arom.}$, $\underline{C}C(O)$), 133.33, 133.11, 132.89, 132.68 ($1C^{quat./arom.}$, $\underline{C}CF_3$), 129.38 ($2C^{phenoxy.}$, $C(CH\underline{C}H)_2CH$), 127.51 ($2C^{arom.}$, $(\underline{C}HCH)_2CCF_3$), 126.44, 124.64, 122.83, 121.02 (1C, $\underline{C}F_3$), 125.57,125.55 ($2C^{arom.}$, $(CH\underline{C}H)_2CCF_3$), 120.43 ($1C^{phenoxy.}$, $C(CHCH)_2\underline{C}H$), 114.55 ($2C^{phenoxy.}$, $C(\underline{C}HCH)_2CH$), 73.39 (1C, $O\underline{C}H_2$), 65.55 (1C, $CH\underline{C}H_2N^{piperazine}$), 58.01 (1C,$CH_2\underline{C}H_2N^{piperazine}$), 53.50 ($2C^{piperazine}$), 53.32 ($2C^{piperazine}$), 40.18 (1C, $\underline{C}H_2NH$), 38.11 ($1C^{cyclohexyl.}$, $OCH_2\underline{C}H$), 35.26 ($1C^{cyclohexyl.}$, $\underline{C}HCH_2N^{piperazine}$), 31.21 ($2C^{cyclohexyl.}$, $\underline{C}H_2$), 29.59 ($2C^{cyclohexyl.}$, $\underline{C}H_2$), 27.42 (1C, $CH_2\underline{C}H_2CH_2$), 24.57 (1C, $CH_2\underline{C}H_2CH_2$).

Preparation of (*Z*)-*N*-(4-(4-((4-(phenoxymethyl)cyclohexyl)methyl)piperazin-1-yl)butyl)benzamide (**8c**)

Benzoyl chloride (0.13 g; $9.18 \cdot 10^{-4}$ mol) in 5 mL DCM was added dropwise to a solution of (*Z*)-4-(4-((4-(phenoxymethyl)cyclohexyl)methyl)piperazin-1-yl)butan-1-amine (**7b**) (0.30 g; $8.34 \cdot 10^{-4}$ mol) and triethylamine (0.34 g; $3.34 \cdot 10^{-3}$ mol) in 10 mL DCM. The reaction was stirred for three hours at room temperature. The mixture was washed 3-times with 10 mL water and dried over Na_2SO_4. The solvent was removed under vacuum and the crude product was purified by column chromatography (EtOAc/MeOH/Triethylamine 49:10:1) to yield the pure product.

(*Z*)-*N*-(4-(4-((4-(phenoxymethyl)cyclohexyl)methyl)piperazin-1-yl)butyl)benzamide (**8c**): $C_{29}H_{41}N_3O_2$. M=463.65. White solid. 95.12 % yield. R_F=0.36 (EtOAc/MeOH/Triethylamine 49:10:1). mp: 76.8-78.7 °C. ^1H NMR (600 MHz, *$CDCl_3$)* δ ppm 7.76-7.75 (m, $2H^{arom.}$, $C(C\underline{H}CH)_2CH$), 7.49-7.46 (m, $1H^{arom.}$, $C(CHCH)_2C\underline{H}$), 7.43-7.40 (m, $2H^{arom.}$, $C(CHC\underline{H})_2CH$), 7.28-7.25 (m, $2H^{phenoxy.}$, $C(CHC\underline{H})_2CH$, 6.95-6.88 (m, $3H^{phenoxy.}$, $C(C\underline{H}CH)_2C\underline{H}$), 6.72 (s, 1H, N$\underline{H}$), 3.85 (d, 2H, $OC\underline{H}_2$, J=6.93 Hz), 3.48-3.45 (m, 2H, $C\underline{H}_2NH$), 2.46-2.38 (m, 10H: $8H^{piperazine}$, $CH_2C\underline{H}_2N^{piperazine}$), 2.20 (d, 2H, $CHC\underline{H}_2N^{piperazine}$, J = 7.33Hz), 2.07-1.96 (m, $1H^{cyclohexyl.}$, $OCH_2C\underline{H}$), 1.73-1.70 (m, $1H^{cyclohexyl.}$, $C\underline{H}CH_2N^{piperazine}$), 1.69-1.35 (m, 12H: $8H^{cyclohexyl.}$, $C\underline{H}_2$; $CH_2C\underline{H}_2CH_2$). ^{13}C NMR (150.95 MHz, *$CDCl_3$)* δ ppm 167.71 ($1C^{quat.}$, $\underline{C}$=O), 159.31 ($1C^{quat./phenoxy.}$, $\underline{C}O$), 135.09 ($1C^{quat./arom.}$, $\underline{C}C(O)$),

131.21 ($1C^{arom.}$, $C(CHCH)_2\underline{C}H$), 129.38 ($2C^{phenoxy.}$, $C(CH\underline{C}H)_2CH$), 128.49 ($2C^{arom.}$, $C(CH\underline{C}H)_2CH$), 126.96 ($2C^{arom.}$, $C(\underline{C}HCH)_2CH$), 120.45 ($1C^{phenoxy.}$, $C(CHCH)_2\underline{C}H$), 114.60 ($2C^{phenoxy.}$, $C(\underline{C}HCH)_2CH$), 71.18 (1C, $O\underline{C}H_2$), 62.50 (1C, $CH\underline{C}H_2N^{piperazine}$), 57.99 (1C, $CH_2\underline{C}H_2N^{piperazine}$), 53.44 ($2C^{piperazine}$), 53.30 ($2C^{piperazine}$), 39.93 (1C, $\underline{C}H_2NH$), 35.39 ($1C^{cyclohexyl.}$, $OCH_2\underline{C}H$), 32.31 ($1C^{cyclohexyl.}$, $\underline{C}HCH_2N^{piperazine}$), 27.46 (1C, $CH_2\underline{C}H_2CH_2$), 27.31 ($2C^{cyclohexyl.}$, $\underline{C}H_2$), 25.72 ($2C^{cyclohexyl.}$, $\underline{C}H_2$), 24.46 (1C, $CH_2\underline{C}H_2CH_2$).

Preparation of (*Z*)-*N*-(4-(4-((4-(phenoxymethyl)cyclohexyl)methyl)piperazin-1-yl)butyl)-4-(trifluoromethyl)benzamide (**8d**)

4-(Trifluoromethyl)benzoyl chloride (0.21 g $1.01 \cdot 10^{-3}$ mol) in 5 mL DCM was added dropwise to a solution of (*Z*)-4-(4-((4-(phenoxymethyl)cyclohexyl)methyl)piperazin-1-yl)butan-1-amine (**7b**) (0.33 g; $9.18 \cdot 10^{-4}$ mol) and triethylamine (0.37 g; $3.67 \cdot 10^{-3}$ mol) in 10 mL DCM. The reaction was stirred for three hours at room temperature. The mixture was washed 3-times with 10 mL water and dried over Na_2SO_4. The solvent was removed under vacuum and the crude product was purified by column chromatography (EtOAc/MeOH/Triethylamine 39:10:1) to yield the pure product.

(*Z*)-*N*-(4-(4-((4-(phenoxymethyl)cyclohexyl)methyl)piperazin-1-yl)butyl)-4-(trifluoromethyl)benzamide (**8d**): $C_{30}H_{40}F_3N_3O_2$. M=513.65. White solid. 82.08 % yield. R_F=0.74 (EtOAc/MeOH/Triethylamine 39:10:1). mp: 100.8-102.0 °C. ^{1}H NMR (600 MHz, *CDCl3*) δ ppm 7.86-7.85 (m, $2H^{arom.}$, $C(C\underline{H}CH)_2CCF_3$), 7.68-7.66 (m, $2H^{arom.}$, $C(CHC\underline{H})_2CCF_3$), 7.27-7.25 (m, $2H^{phenoxy.}$, $C(CHC\underline{H})_2CH$), 7.15 (br, 1H, $N\underline{H}$), 6.93-6.88 (m, $3H^{phenoxy.}$, $C(C\underline{H}CH)_2C\underline{H}$), 3.85 (d, 2H, $OC\underline{H}_2$, *J*=6.91 Hz), 3.48-3.45 (m, 2H, $C\underline{H}_2NH$), 2.44-2.33 (m, 10H: $8H^{piperazine}$, $CH_2C\underline{H}_2N^{piperazine}$), 2.16 (d, 2H, $CHC\underline{H}_2N^{piperazine}$, *J* = 7.32Hz), 2.00-1.97 (m, $1H^{cyclohexyl.}$, $OCH_2C\underline{H}$), 1.71-1.47 (m, 11H: $7H^{cyclohexyl.}$, $C\underline{H}CH_2N^{piperazine}$, $CH_2^{cyclohexyl}$; $CH_2C\underline{H}_2CH_2$), 1.39-1.35 (m, 2H, $CH_2^{cyclohexyl}$). ^{13}C NMR (150.95 MHz, *CDCl3*) δ ppm 166.53 ($1C^{quat.}$, $\underline{C}$=O), 159.27 ($1C^{quat./phenoxy.}$, $\underline{C}O$), 138.53 ($1C^{quat./arom.}$, $\underline{C}C(O)$)), 133.24, 133.02, 132.81, 132.59 ($1C^{quat./arom.}$, $\underline{C}CF_3$), 129.43 ($2C^{phenoxy.}$, $C(CH\underline{C}H)_2CH$), 127.50 ($2C^{arom.}$, $(\underline{C}HCH)_2C$ CF_3), 126.40, 124.59, 122.79, 120.99 (1C, $\underline{C}F_3$), 125.50,125.47 ($2C^{arom.}$, $(\underline{C}H)_2CCF_3$), 120.42 ($1C^{phenoxy.}$, $C(CHCH)_2\underline{C}H$), 114.57 ($2C^{phenoxy.}$, $C(\underline{C}HCH)_2CH$), 71.14 (1C, $O\underline{C}H_2$), 62.44 (1C, $CH\underline{C}H_2N^{piperazine}$), 57.95 (1C, $CH_2\underline{C}H_2N^{piperazine}$), 53.38 ($2C^{piperazine}$), 53.28 ($2C^{piperazine}$), 40.11 (1C, $\underline{C}H_2NH$), 35.35 ($1C^{cyclohexyl.}$: $OCH_2\underline{C}H$), 32.26 ($1C^{cyclohexyl.}$: $\underline{C}HCH_2N^{piperazine}$), 27.35 (1C, $CH_2\underline{C}H_2CH_2$), 27.23 ($2C^{cyclohexyl.}$: $\underline{C}H_2$), 25.67 ($2C^{cyclohexyl.}$: $\underline{C}H_2$), 24.48 (1C, $CH_2\underline{C}H_2CH_2$).

Preparation of (*E)*-4-(4-((4-(phenoxymethyl)cyclohexyl)methyl)piperazin-1-yl)-*N*-(benzyl)butan-1-amine (**9a**)

$LiAlH_4$ (0.19 g; $5.09 \cdot 10^{-3}$ mol) was added to a solution of (*E)*-*N*-(4-(4-((4-(phenoxymethyl)cyclohexyl)methyl)piperazin-1-yl)butyl)benzamide (**8a**) (0.59 g; $1.27 \cdot 10^{-3}$ mol) in 40 mL diethyl ether. The reaction was stirred overnight at room temperature, then the mixture was quenched by dropwise addition of water (16 eq.) and 10 % NaOH solution (16 eq.) stirred for two hours, then filtered. The precipitate was discarded. The organic layer was dried over Na_2SO_4, then the solvent was removed under vacuum and the crude product was purified by column chromatography (DCM/MeOH/25% NH_3 aq. 89:10:1) to yield the pure product.

(*E)*-4-(4-((4-(phenoxymethyl)cyclohexyl)methyl)piperazin-1-yl)-*N*-(benzyl)butan-1-amine (**9a**): $C_{29}H_{43}N_3O$. M=449.67. Light yellow solid. 68.42 % yield. R_F=0.63 (DCM/MeOH/25% NH_3 aq. 89:10:1). mp: 68.8-70.8 °C. ^{1}H NMR (600 MHz, *CDCl3*) δ ppm 7.34-7.30 (m, $4H^{arom.}$, $C(C\underline{H}C\underline{H})_2CH$), 7.26-7.25 (m, 3H: $2H^{phenoxy.}$, $C(CHC\underline{H})_2CH$; $1H^{arom.}$, $C(CHCH)_2C\underline{H}$), 6.92-6.88 (m, $3H^{phenoxy.}$, $C(C\underline{H}CH)_2C\underline{H}$), 3.80 (s, 2H, $PhC\underline{H}_2$), 3.75 (d, 2H, $OC\underline{H}_2$, *J*=6.33Hz), 2.77 (br, 1H, NH), 2.66 (t, 2H, $CH_2C\underline{H}_2NH$, *J*=6.54Hz), 2.45-2.40 (m, $8H^{piperazine}$), 2.34 (t, 2H, $CH_2C\underline{H}_2N^{piperazine}$, *J*=7.04Hz), 2.13 (d, 2H, $CHC\underline{H}_2N^{piperazine}$, *J*=7.07Hz), 1.92-1.90 (m, $2H^{cyclohexyl.}$, $C\underline{H}_2$), 1.87-1.85 (m, $2H^{cyclohexyl.}$, $C\underline{H}_2$), 1.78-1.73 (m, $1H^{cyclohexyl.}$, $C\underline{H}$), 1.56-1.53 (m, 4H, $CH_2C\underline{H}_2CH_2$), 1.49-1.45 (m, 1H, $1H^{cyclohexyl}$), 1.09-1.02 (m, $2H^{cyclohexyl.}$, $C\underline{H}_2$), 0.96-0.89 (m, $2H^{cyclohexyl.}$, $C\underline{H}_2$). ^{13}C NMR (150.95 MHz, *CDCl3*) δ ppm 159.26 ($1C^{quat./phenoxy.}$, $\underline{C}O$), 139.71 ($1C^{quat./arom.}$, $\underline{C}CH_2$), 129.34 ($2C^{phenoxy.}$, $C(CH\underline{C}H)_2CH$), 128.41 ($2C^{arom.}$, $C(CH\underline{C}H)_2CH$), 128.22 ($2C^{arom.}$, $C(\underline{C}HCH)_2CH$), 127.04 ($1C^{arom.}$, $C(CHCH)_2\underline{C}H$), 120.37 ($1C^{phenoxy.}$, $C(CHCH)_2\underline{C}H$), 114.48 ($2C^{phenoxy.}$, $C(\underline{C}HCH)_2CH$), 73.33 (1C, $O\underline{C}H_2$), 65.54 (1C, $CH\underline{C}H_2N^{piperazine}$), 58.49 (1C, $CH_2\underline{C}H_2N^{piperazine}$), 53.73 (1C, $Ph\underline{C}H_2$), 53.58 ($2C^{piperazine}$), 53.24 ($2C^{piperazine}$), 49.09 (1C, $CH_2\underline{C}H_2NH$), 38.05

($1C^{cyclohexyl.}$, $OCH_2\underline{C}H$), 35.19 ($1C^{cyclohexyl.}$, $\underline{C}HCH_2N^{piperazine}$), 31.18 ($2C^{cyclohexyl.}$, $\underline{C}H_2$), 29.56 ($2C^{cyclohexyl.}$, $\underline{C}H_2$), 27.88 (1C, $CH_2\underline{C}H_2CH_2$), 24.69 (1C, $CH_2\underline{C}H_2CH_2$).

Preparation of (*E*)-4-(4-((4-(phenoxymethyl)cyclohexyl)methyl)piperazin-1-yl)-*N*-(4-(trifluoromethyl)benzyl)butan-1-amine (**9b**)

$LiAlH_4$ (0.097 g; $2.57{\cdot}10^{-3}$ mol) was added to a solution of (*E)-N*-(4-(4-((4-(phenoxymethyl)cyclohexyl)methyl)piperazin-1-yl)butyl)-4-(trifluoromethyl)benzamide (**8b**) (0.33 g; $6.42{\cdot}10^{-4}$ mol) in 30 mL THF. The reaction was stirred overnight at room temperature, then the mixture was quenched by dropwise addition of water (16 eq.) and 10 % NaOH solution (16 eq.) stirred for two hours, then filtered. The precipitate was discarded. The organic layer was dried over Na_2SO_4, then the solvent was removed under vacuum and the crude product was purified by column chromatography (DCM/MeOH/25% NH_3 aq. 189:10:1) to yield the pure product.

(*E)*-4-(4-((4-(phenoxymethyl)cyclohexyl)methyl)piperazin-1-yl)-*N*-(4-(trifluoromethyl)benzyl)butan-1-amine (**9b**): $C_{30}H_{42}F_3N_3O$. M=517.67. Light yellow solid. 53.57 % yield. R_F=0.40 (DCM/MeOH/25% NH_3 aq. 139:10:1). mp: 51.5-53.5 °C. ^{1}H NMR (600 MHz, *$CDCl_3$)* δ ppm 7.57-7.56 (m, $2H^{arom.}$, $C(CHC\underline{H})_2CCF_3$), 7.45-7.44 (m, $2H^{arom.}$, $C(C\underline{H}CH)_2CCF_3$), 7.27-7.25 (m, $2H^{phenoxy.}$, $C(CHC\underline{H})_2CH$), 6.96-6.82 (m, $3H^{phenoxy.}$, $C(C\underline{H}CH)_2C\underline{H}$), 3.84 (s, 2H, $CF_3PhC\underline{H}_2$), 3.76 (d, 2H, $OC\underline{H}_2$, *J*=6.30Hz), 2.65-2.32 (m, 12H: $8H^{piperazine}$, $N^{piperazine}C\underline{H}_2CH_2CH_2C\underline{H}_2$), 2.14 (d, 2H, $CHC\underline{H}_2N^{piperazine}$, *J* = 7.03 Hz), 1.93-1.91 (m, $2H^{cyclohexyl.}$, $C\underline{H}_2$), 1.88-1.86 (m, $2H^{cyclohexyl.}$, $C\underline{H}_2$), 1.81-1.70 (m, $1H^{cyclohexyl.}$, $C\underline{H}$), 1.53-1.51 (m, 5H, $CH_2C\underline{H}_2CH_2$, $N\underline{H}$ *), 1.51-1.44 (m, $1H^{cyclohexyl.}$, $C\underline{H}$), 1.09-1.02 (m, $2H^{cyclohexyl.}$, $C\underline{H}_2$), 0.97-0.90 (m, $2H^{cyclohexyl.}$, $C\underline{H}_2$). ^{13}C NMR (150.95 MHz, *$CDCl_3$)* δ ppm 159.33 ($1C^{quat./phenoxy.}$, $\underline{C}O$), 129.54, 129.33, 129.11, 128.90 ($1C^{quat./arom.}$, $\underline{C}CF_3$), 129.38 ($2C^{phenoxy.}$, $C(CH\underline{C}H)_2CH$), 128.26 ($1C^{quat./arom.}$, $\underline{C}CH_2$), 127.21 ($2C^{arom.}$, $C(\underline{C}HCH)_2CCF_3$), 126.99, 125.19, 123.39, 121.59 (1C, $\underline{C}F_3$), 125.30,125.27 ($2C^{arom.}$, $C(CH\underline{C}H)_2CCF_3$), 120.42 ($1C^{phenoxy.}$, $C(CHCH)_2\underline{C}H$), 114.55 ($2C^{phenoxy.}$, $C(\underline{C}HCH)_2CH$), 70.40 (1C, $O\underline{C}H_2$), 65.63 (1C, $CH\underline{C}H_2N^{piperazine}$), 58.59 (1C,$CH_2\underline{C}H_2N^{piperazine}$), 53.73 ($2C^{piperazine}$), 53.47 (1C, $CF_3Ph\underline{C}H_2$), 53.36 ($2C^{piperazine}$), 49.36 (1C, $CH_2\underline{C}H_2NH$), 38.12 ($1C^{cyclohexyl.}$, $OCH_2\underline{C}H$), 35.28 ($1C^{cyclohexyl.}$, $\underline{C}HCH_2N^{piperazine}$), 31.25 ($2C^{cyclohexyl.}$, $\underline{C}H_2$), 29.62 ($2C^{cyclohexyl.}$, $\underline{C}H_2$), 28.16 (1C, $CH_2\underline{C}H_2CH_2$), 24.75 (1C, $CH_2\underline{C}H_2CH_2$).

Preparation of (*Z*)-4-(4-((4-(phenoxymethyl)cyclohexyl)methyl)piperazin-1-yl)-*N*-(benzyl)butan-1-amine (**9c**)

$LiAlH_4$ (0.12 g; $3.27{\cdot}10^{-3}$ mol) was added to a solution of (*Z*)-*N*-(4-(4-((4-(phenoxymethyl)cyclohexyl)methyl)piperazin-1-yl)butyl)benzamide (**8c**) (0.38 g; $8.19{\cdot}10^{-4}$ mol) in 40 mL diethyl ether. The reaction was stirred overnight at room temperature, then the mixture was quenched by dropwise addition of water (16 eq.) and 10 % NaOH solution (16 eq.) stirred for two hours, then filtered. The precipitate was discarded. The organic layer was dried over Na_2SO_4, then the solvent was removed under vacuum and the crude product was purified by column chromatography (DCM/MeOH/25% NH_3 aq. 89:10:1) to yield the pure product.

(*Z*)-4-(4-((4-(phenoxymethyl)cyclohexyl)methyl)piperazin-1-yl)-*N*-(benzyl)butan-1-amine (**9c**): $C_{29}H_{43}N_3O$. M=449.67. Colorless sticky oil. 57.86 % yield. R_F=0.73 (DCM/MeOH/25% NH_3 aq. 89:10:1). ^{1}H NMR (600 MHz, *$CDCl_3$)* δ ppm 7.34-7.31 (m, $4H^{arom.}$, $C(C\underline{H}C\underline{H})_2CH$), 7.28-7.23 (m, 3H: $2H^{phenoxy.}$, $C(CHC\underline{H})_2CH$; $1H^{arom.}$, $C(CHCH)_2C\underline{H}$), 6.93-6.88 (m, $3H^{phenoxy.}$, $C(C\underline{H}CH)_2C\underline{H}$), 3.85 (d, 2H, $OC\underline{H}_2$, *J*=6.93 Hz), 3.80 (s, 2H, $PhC\underline{H}_2$), 2.67 (t, 2H, $CH_2C\underline{H}_2NH$, *J*=6.47Hz), 2.44-2.33 (m, 10H: $8H^{piperazine}$, $CH_2C\underline{H}_2N^{piperazine}$), 2.21 (d, 2H, $CHC\underline{H}_2N^{piperazine}$, *J*=7.32Hz), 2.01-1.97 (m, $1H^{cyclohexyl.}$, $OCH_2C\underline{H}$), 1.74-1.70 (m, $1H^{cyclohexyl.}$, $C\underline{H}CH_2N^{piperazine}$), 1.62-1.35 (m, 13H: $8H^{cyclohexyl.}$, $C\underline{H}_2$; $CH_2C\underline{H}_2CH_2$, $N\underline{H}$ *). ^{13}C NMR (150.95 MHz, *$CDCl_3$)* δ ppm 159.31 ($1C^{quat./phenoxy.}$, $\underline{C}O$), 139.94 ($1C^{quat./arom.}$, $\underline{C}CH_2$), 129.37 ($2C^{phenoxy.}$, $C(CH\underline{C}H)_2CH$), 128.43 ($2C^{arom.}$, $C(CH\underline{C}H)_2CH$), 128.22 ($2C^{arom.}$, $C(\underline{C}HCH)_2CH$), 127.03 ($1C^{arom.}$, $C(CHCH)_2\underline{C}H$), 120.43 ($1C^{phenoxy.}$, $C(CHCH)_2\underline{C}H$), 114.60 ($2C^{phenoxy.}$, $C(\underline{C}HCH)_2CH$), 71.18 (1C, $O\underline{C}H_2$), 62.57 (1C, $CH\underline{C}H_2N^{piperazine}$), 58.55 (1C, $CH_2\underline{C}H_2N^{piperazine}$), 53.85 (1C, $Ph\underline{C}H_2$), 53.61 ($2C^{piperazine}$), 53.32 ($2C^{piperazine}$), 49.21 (1C, $CH_2C\underline{H}_2NH$), 35.38 ($1C^{cyclohexyl.}$, $OCH_2\underline{C}H$), 32.33 ($1C^{cyclohexyl.}$, $\underline{C}HCH_2N^{piperazine}$), 28.00 (1C, $CH_2\underline{C}H_2CH_2$), 27.32 ($2C^{cyclohexyl.}$, $\underline{C}H_2$), 25.74 ($2C^{cyclohexyl.}$, $\underline{C}H_2$), 24.76 (1C, $CH_2\underline{C}H_2CH_2$).

Preparation of (*Z*)-4-(4-((4-(phenoxymethyl)cyclohexyl)methyl)piperazin-1-yl)-*N*-(4-(trifluoromethyl)benzyl)butan-1-amine (**9d**)

$LiAlH_4$ (0.11 g; $2.89\cdot10^{-3}$ mol) was added to a solution of (*Z*)-*N*-(4-(4-((4-(phenoxymethyl)cyclohexyl)methyl)piperazin-1-yl)butyl)-4-(trifluoromethyl)benzamide (**8d**) (0.36 g; $7.01\cdot10^{-4}$ mol) in 40 mL diethyl ether. The reaction was stirred overnight at room temperature, then the mixture was quenched by dropwise addition of water (16 eq.) and 10 % NaOH solution (16 eq.) stirred for two hours, then filtered. The precipitate was discarded. The organic layer was dried over Na_2SO_4, then the solvent was removed under vacuum and the crude product was purified by column chromatography (EtOAc/MeOH/Triethylamine 39:10:1) to yield the pure product.

(*Z*)-4-(4-((4-(phenoxymethyl)cyclohexyl)methyl)piperazin-1-yl)-*N*-(4-(trifluoromethyl)benzyl)butan-1-amine (**9d**): $C_{30}H_{42}F_3N_3O$. M=517.67. Light yellow sticky oil. 53.57 % yield. R_F=0.48 (EtOAc/MeOH/Triethylamine 39:10:1). ^1H NMR (600 MHz, *CDCl₃*) δ ppm 7.58-7.57 (m, $2H^{arom.}$, $C(CHC\underline{H})_2CCF_3$), 7.47-7.45 (m, $2H^{arom.}$, $C(C\underline{H}CH)_2CCF_3$), 7.28-7.25 (m, $2H^{phenoxy.}$, $C(CHC\underline{H})_2CH$), 6.93-6.88 (m, $3H^{phenoxy.}$, $C(C\underline{H}CH)_2C\underline{H}$), 3.85-3.84 (m, 4H, $OC\underline{H}_2$, $CF_3PhC\underline{H}_2$), 2.65 (t, 2H, $CH_2C\underline{H}_2NH$, *J*=5.75Hz), 2.46-2.34 (m, 10H: $8H^{piperazine}$, $CH_2C\underline{H}_2N^{piperazine}$), 2.21 (d, 2H, $CHC\underline{H}_2N^{piperazine}$, *J*=7.33Hz), 2.01-1.97 (m, $1H^{cyclohexyl.}$, $OCH_2C\underline{H}$), 1.74-1.71 (m, $1H^{cyclohexyl.}$, $C\underline{H}CH_2N^{piperazine}$), 1.62-1.47 (m, 11H: $6H^{cyclohexyl.}$, CH_2; $CH_2C\underline{H}_2CH_2$; $N\underline{H}$), 1.40-1.36 (m, 2H, $CH_2^{cyclohexyl}$). ^{13}C NMR (150.95 MHz, *CDCl₃*) δ ppm 159.31 ($1C^{quat./phenoxy.}$, $\underline{C}O$), 129.37 ($2C^{phenoxy.}$, $C(CH\underline{C}H)_2CH$), 129.19, 128.98, 128.84, 128.66 ($1C^{quat./arom.}$, $\underline{C}CF_3$), 128.49 ($1C^{quat./arom.}$, $\underline{C}CH_2$), 128.36 ($2C^{arom.}$, $C(\underline{C}HCH)_2CCF_3$), 126.95, 125.15, 123.34, 121.56 (1C, $\underline{C}F_3$), 125.33,125.31 ($2C^{arom.}$, $C(CH\underline{C}H)_2CCF_3$), 120.44 ($1C^{phenoxy.}$, $C(CHCH)_2\underline{C}H$), 114.59 ($2C^{phenoxy.}$, $C(\underline{C}HCH)_2CH$), 71.16 (1C, $O\underline{C}H_2$), 62.44 (1C, $CH\underline{C}H_2N^{piperazine}$), 58.49 (1C, $CH_2\underline{C}H_2N^{piperazine}$), 53.47 (1C, $CF_3Ph\underline{C}H_2$), 53.32 ($2C^{piperazine}$), 53.29 ($2C^{piperazine}$), 49.24 (1C, CH_2CH_2NH), 35.37 ($1C^{cyclohexyl.}$, $OCH_2\underline{C}H$), 32.32 ($1C^{cyclohexyl.}$, $\underline{C}HCH_2N^{piperazine}$), 28.00 (1C, $CH_2\underline{C}H_2CH_2$), 27.29 ($2C^{cyclohexyl.}$, $\underline{C}H_2$), 25.72 ($2C^{cyclohexyl.}$, $\underline{C}H_2$), 24.66 (1C, $CH_2\underline{C}H_2CH_2$).

Preparation of (*E)*-2,3-di(*tert*-butoxycarbonyl)-1-{4-{4-{[4-(phenoxymethyl)cyclohexyl]methyl}piperazin-1-yl}but-1-yl}-1-(benzyl)guanidine (**10a**)

1,3-bis(*tert*-butoxycarbonyl)-2-methylisothiourea (0.28 g; $9.54\cdot10^{-4}$ mol) and mercury II chloride (0.26 g; $9.54\cdot10^{-4}$ mol) were sequentially added to an ice-cooled mixture of (*E)*-4-(4-((4-(phenoxymethyl)cyclohexyl)methyl)piperazin-1-yl)-*N*-(benzyl)butan-1-amine (**9a**) (0.39 g; $8.67\cdot10^{-4}$ mol) and triethylamine (0.44 g; $4.33\cdot10^{-3}$ mol) in 40 mL DCM. The ice bath was removed and the reaction was stirred for eighteen hours at room temperature, then filtered. The precipitate was discarded. The filtrate was washed sequentially twice with 15 mL H_2O and twice with 15 mL brine. The combined organic phases were dried over Na_2SO_4, then the solvent was removed under vacuum and the crude product was purified by column chromatography (EtOAc/MeOH 89:10) to yield the pure product.

(*E)*-2,3-di(*tert*-butoxycarbonyl)-1-{4-{4-{[4-(phenoxymethyl)cyclohexyl]methyl}piperazin-1-yl}but-1-yl}-1-(benzyl)guanidine (**10a**): $C_{40}H_{61}N_5O_5$. M=691.94. White solid. 76.60 % yield. R_F=0.29 (EtOAc/MeOH 89:10). mp: 118.5-119.5 °C. ^1H NMR (600 MHz, *CDCl₃*) δ ppm 9.93 (br, 1H, $N\underline{H}$), 7.33-7.31 (m, $2H^{arom.}$, $C(C\underline{H}CH)_2CH$), 7.27-7.25 (m, 5H: $2H^{phenoxy.}$, $C(CHC\underline{H})_2CH$, $3H^{arom.}$, $C(CHC\underline{H})_2C\underline{H}$), 6.92-6.88 (m, $3H^{phenoxy.}$, $C(C\underline{H}CH)_2C\underline{H}$), 4.66 (br, 2H, $PhC\underline{H}_2$), 3.76 (d, 2H, $OC\underline{H}_2$, *J* = 6.31 Hz), 3.35 (m, 2H, $CH_2C\underline{H}_2C(N)N$), 2.41-2.27 (m, 10H: $8H^{piperazine}$, $CH_2C\underline{H}_2N^{piperazine}$), 2.14 (d, 2H, $CHC\underline{H}_2N^{piperazine}$, *J*=7.02Hz), 1.93-1.90 (m, $2H^{cyclohexyl.}$, $C\underline{H}_2$), 1.87-1.85 (m, $2H^{cyclohexyl.}$, $C\underline{H}_2$), 1.76-1.74 (m, $1H^{cyclohexyl.}$, $OCH_2C\underline{H}$), 1.59-1.39 (m, 23H: $CH_2C\underline{H}_2CH_2$; $C\underline{H}_3$; $1H^{cyclohexyl.}$, $C\underline{H}CH_2N^{piperazine}$), 1.09-1.02 (m, $2H^{cyclohexyl.}$, $C\underline{H}_2$), 0.96-0.30 (m, $2H^{cyclohexyl.}$, $C\underline{H}_2$). ^{13}C NMR (150.95 MHz, *CDCl₃*) δ ppm 162.68 (1C, $\underline{C}$=O), 159.25 ($1C^{quat./phenoxy.}$, $\underline{C}O$), 155.95 ($1C^{quat.}$, $\underline{C}$=N), 150.97 (1C, $\underline{C}$=O), 136.38 ($1C^{quat./arom.}$, $\underline{C}CH_2$), 129.33 ($2C^{phenoxy.}$, $C(CH\underline{C}H)_2CH$), 128.59 ($2C^{arom.}$), 127.85 ($2C^{arom.}$), 127.52 ($1C^{arom.}$, $C(CHCH)_2\underline{C}H$), 120.36 ($1C^{phenoxy}$, $C(CHCH)_2\underline{C}H$), 114.47 ($2C^{phenoxy}$, $C(\underline{C}HCH)_2CH$), 81.86 ($1C^{quat.}$ Boc), 79.36 ($1C^{quat.}$ Boc), 73.32 (1C, $OC\underline{H}_2$), 65.55 (1C, $CH\underline{C}H_2N^{piperazine}$), 58.01 (1C, $CH_2\underline{C}H_2N^{piperazine}$), 53.63 ($2C^{piperazine}$), 53.17 ($2C^{piperazine}$), 51.79 (1C, $PhC\underline{H}_2$), 47.30 (1C, $CH_2\underline{C}H_2C(N)N$), 38.04 ($1C^{cyclohexyl.}$, $OCH_2\underline{C}H$), 35.18 ($1C^{cyclohexyl.}$, $\underline{C}HCH_2N^{piperazine}$), 31.18 ($2C^{cyclohexyl.}$, $\underline{C}H_2$), 29.55 ($2C^{cyclohexyl.}$, $\underline{C}H_2$), 28.16, 28.14 (6C, $\underline{C}H_3$), 25.09 (1C, $CH_2\underline{C}H_2CH_2$), 23.78 (1C, $CH_2\underline{C}H_2CH_2$).

Preparation of (*E*)-2,3-di(*tert*-butoxycarbonyl)-1-{4-{4-{[4-(phenoxymethyl)cyclohexyl]methyl}piperazin-1-yl}but-1-yl}-1-[4-(trifluoromethyl)benzyl]guanidine (**10b**)

1,3-bis(*tert*-butoxycarbonyl)-2-methylisothiourea (0.11 g; $3.61{\cdot}10^{-4}$ mol) and mercury II chloride (0.10 g; $3.61{\cdot}10^{-4}$ mol) were sequentially added to an ice-cooled mixture of (*E*)-4-(4-((4-(phenoxymethyl)cyclohexyl)methyl)piperazin-1-yl)-*N*-(4-(trifluoromethyl)benzyl)butan-1-amine (**9b**) (0.17 g; $3.28{\cdot}10^{-4}$ mol) and triethylamine (0.17 g; $1.64{\cdot}10^{-3}$ mol) in 20 mL DCM. The ice bath was removed and the reaction was stirred for eighteen hours at room temperature, then filtered. The precipitate was discarded. The filtrate was washed sequentially twice with 15 mL H_2O and twice with 15 mL brine. The combined organic phases were dried over Na_2SO_4, then the solvent was removed under vacuum and the crude product was purified by column chromatography (EtOAc/MeOH/Triethylamine 139:10:1) to yield the pure product.

(*E*)-2,3-di(*tert*-butoxycarbonyl)-1-{4-{4-{[4-(phenoxymethyl)cyclohexyl]methyl}piperazin-1-yl}but-1-yl}-1-[4-(trifluoromethyl)benzyl]guanidine (**10b**): $C_{41}H_{60}F_3N_5O_5$. M=759.94. White waxy solid. 53.95 % yield. R_F=0.39 (EtOAc/MeOH/Triethylamine 139:10:1). mp: 117-118 °C. ^{1}H NMR (600 MHz, *CDCl₃*) δ ppm 9.95 (br, 1H, $N\underline{H}$), 7.59-7.58 (m, $2H^{arom.}$, $C(CHC\underline{H})_2CCF_3$), 7.43-7.41 (m, $2H^{arom.}$, $C(C\underline{H}CH)_2CCF_3$), 7.27-7.25 (m, $2H^{phenoxy.}$, $C(CHC\underline{H})_2CH$), 6.93-6.90 (m, $1H^{phenoxy.}$, $C(CHCH)_2C\underline{H}$), 6.89-6.88 (m, $2H^{phenoxy.}$, $C(C\underline{H}CH)_2CH$), 4.77 (br, 2H, $CF_3PhC\underline{H}_2$), 3.76 (d, 2H, $OC\underline{H}_2$, *J*=6.30Hz), 3.33 (m, 2H, $CH_2C\underline{H}_2C(N)N$), 2.41-2.28 (m, 10H: $8H^{piperazine}$, $C\underline{H}_2CH_2N^{piperazine}$), 2.14 (d, 2H, $CHC\underline{H}_2N^{piperazine}$), 1.92-1.91 (m, $2H^{cyclohexyl.}$, $C\underline{H}_2$), 1.87-1.86 (m, $2H^{cyclohexyl.}$, $C\underline{H}_2$), 1.75 (m, $1H^{cyclohexyl.}$, $C\underline{H}$), 1.57 (qt, 2H, $CH_2C\underline{H}_2CH_2$), 1.53-1.37 (m, 21H: $C\underline{H}_3$; $CH_2C\underline{H}_2CH_2$; $1H^{cyclohexyl.}$, $C\underline{H}$), 1.09-1.02 (m, $2H^{cyclohexyl.}$, $C\underline{H}_2$), 0.96-0.89 (m, $2H^{cyclohexyl.}$, $C\underline{H}_2$). ^{13}C NMR (150.95 MHz, *CDCl₃*) δ ppm 162.64 (1C, $\underline{C}$=O), 159.33 ($1C^{quat/phenoxy.}$, $\underline{C}O$) 156.18 (1C, $\underline{C}$=N), 150.97 (1C, $\underline{C}$=O), 140.78 ($1C^{quat./arom.}$, $\underline{C}CH_2NC(N)$)), 129.89, 129.70, 129.38, 129.18 (1C, $\underline{C}CF_3$), 129.38 ($2C^{phenoxy.}$, $C(CH\underline{C}H)_2CH$), 128.03 ($2C^{arom.}$, $C(\underline{C}HCH)_2CCF_3$), 126.84, 125.04, 123.23, 121.43 (1C, $\underline{C}F_3$), 125.60, 125.58 ($2C^{arom.}$, $C(CH\underline{C}H)_2CCF_3$), 120.43 ($1C^{phenoxy.}$, $C(CHCH)_2\underline{C}H$), 114.55 ($2C^{phenoxy.}$, $C(\underline{C}HCH)_2CH$), 82.124 ($1C^{quat.}$ Boc), 79.61($1C^{quat.}$ Boc), 73.40 (1C $O\underline{C}H_2$), 65.60 (1C, $CH\underline{C}H_2N^{piperazine}$), 57.96 (1C, $CH_2\underline{C}H_2N^{piperazine}$), 53.69 ($2C^{piperazine}$), 53.25 ($2C^{piperazine}$), 50.94 (1C, $CF_3Ph\underline{C}H_2$), 48.15 (1C, $CH_2\underline{C}H_2C(N)N$), 38.12 ($1C^{cyclohexyl.}$, $OCH_2\underline{C}H$), 35.28 ($1C^{cyclohexyl}$, $\underline{C}HCH_2N^{piperazine}$), 31.24 ($2C^{cyclohexyl.}$, $\underline{C}H_2$), 29.16 ($2C^{cyclohexyl.}$, $\underline{C}H_2$), 28.22, 28.16 (6C, $\underline{C}H_3$), 25.27 (1C, $CH_2\underline{C}H_2CH_2$), 23.87 (1C, $CH_2\underline{C}H_2CH_2$).

Preparation of (*Z*)-2,3-di(*tert*-butoxycarbonyl)-1-{4-{4-{[4-(phenoxymethyl)cyclohexyl]methyl}piperazin-1-yl}but-1-yl}-1-(benzyl)guanidine (**10c**)

1,3-bis(*tert*-butoxycarbonyl)-2-methylisothiourea (0.18 g; $6.12{\cdot}10^{-4}$ mol) and mercury II chloride (0.17 g; $6.12{\cdot}10^{-4}$ mol) were sequentially added to an ice-cooled mixture of (*Z*)-4-(4-((4-(phenoxymethyl)cyclohexyl)methyl)piperazin-1-yl)-*N*-(benzyl)butan-1-amine (**9c**) (0.25 g; $5.56{\cdot}10^{-4}$ mol) and triethylamine (0.28 g; $2.78{\cdot}10^{-3}$ mol) in 30 mL DCM. The ice bath was removed and the reaction was stirred for eighteen hours at room temperature, then filtered. The precipitate was discarded. The filtrate was washed sequentially twice with 15 mL H_2O and twice with 15 mL brine. The combined organic phases were dried over Na_2SO_4, then the solvent was removed under vacuum and the crude product was purified by column chromatography (EtOAc/MeOH 89:10) to yield the pure product.

(*Z*)-2,3-di(*tert*-butoxycarbonyl)-1-{4-{4-{[4-(phenoxymethyl)cyclohexyl]methyl}piperazin-1-yl}but-1-yl}-1-(benzyl)guanidine (**10c**): $C_{40}H_{61}N_5O_5$. M=691.94. Colorless sticky oil. 74.05 % yield. R_F=0.38 (EtOAc/MeOH 89:10). ^{1}H NMR (600 MHz, *CDCl₃*) δ ppm 9.91 (1H, $N\underline{H}$), 7.33-7.31 (m, $2H^{arom.}$, $C(CHC\underline{H})_2CH$), 7.28-7.25 (m, 5H: $2H^{phenoxy.}$, $C(CHC\underline{H})_2CH$, $3H^{arom.}$, $C(C\underline{H}CH)_2C\underline{H}$), 6.93-6.98 (m, $3H^{phenoxy.}$, $C(C\underline{H}CH)_2C\underline{H}$), 4.65 (br, 2H, $PhC\underline{H}_2$), 3.85 (d, 2H, $OC\underline{H}_2$, *J*=6.94 Hz), 3.35 (br, 2H, $CH_2C\underline{H}_2C(N)N$), 2.42-2.29 (m, 10H: $8H^{piperazine}$, $CH_2C\underline{H}_2N^{piperazine}$), 2.22 (d, 2H, $CHC\underline{H}_2N^{piperazine}$, *J*=7.31Hz), 2.01-1.97 (m, $1H^{cyclohexyl.}$, $OCH_2C\underline{H}$), 1.75-1.71 (m, $1H^{cyclohexyl.}$, $C\underline{H}CH_2N^{piperazine}$), 1.62-1.36 (m, 30H: $C\underline{H}_3$, $CH_2C\underline{H}_2CH_2$, $8H^{cyclohexyl.}$, $C\underline{H}_2$). ^{13}C NMR (150.95 MHz, *CDCl₃*) δ ppm 171.07 (1C, $\underline{C}$=O), 162.69 (1C, $\underline{C}$=O), 159.30 ($1C^{quat./phenoxy.}$, $\underline{C}O$), 156.96 ($1C^{quat.}$, $\underline{C}$=N), 136.43 ($1C^{quat./arom.}$, $\underline{C}CH_2$), 129.36 ($2C^{phenoxy.}$, $C(CH\underline{C}H)_2CH$), 128.63 ($2C^{arom.}$), 127.88 ($2C^{arom.}$), 127.56 ($1C^{arom.}$, $C(CHCH)_2\underline{C}H$), 120.43($1C^{phenoxy}$, $C(CHCH)_2\underline{C}H$), 114.59 ($2C^{phenoxy}$, $C(\underline{C}HCH)_2CH$), 81.90 ($1C^{quat.}$ Boc), 79.35

(1C$^{quat.}$ Boc), 71.17 (1C, OC<u>H</u>$_2$), 62.54 (1C, CH<u>C</u>H$_2$N^{piperazine}), 58.02 (1C, CH$_2$<u>C</u>H$_2$N^{piperazine}), 53.57 (2C^{piperazine}), 53.20 (2C^{piperazine}), 50.56 (1C, PhC<u>H</u>$_2$), 47.39 (1C, CH$_2$<u>C</u>H$_2$C(N)N), 35.38 (1C$^{cyclohexyl.}$, OCH$_2$<u>C</u>H), 32.31 (1C$^{cyclohexyl.}$, <u>C</u>HCH$_2$N^{piperazine}), 28.24, 28.14 (6C, <u>C</u>H$_3$), 27.31 (2C$^{cyclohexyl.}$, <u>C</u>H$_2$), 25.72 (2C$^{cyclohexyl.}$, <u>C</u>H$_2$), 25.11 (1C, CH$_2$<u>C</u>H$_2$CH$_2$), 23.76 (1C, CH$_2$<u>C</u>H$_2$CH$_2$).

Preparation of (*Z*)-2,3-di(*tert*-butoxycarbonyl)-1-{4-{4-{[4-(phenoxymethyl)cyclohexyl]methyl}piperazin-1-yl}but-1-yl}-1-[4-(trifluoromethyl)benzyl]guanidine (**10d**)

1,3-bis(*tert*-butoxycarbonyl)-2-methylisothiourea (0.09 g; 2.97·10^{-4} mol) and mercury II chloride (0.08 g; 2.97·10^{-4} mol) were sequentially added to an ice-cooled mixture of (*Z*)-4-(4-((4-(phenoxymethyl)cyclohexyl)methyl)piperazin-1-yl)-*N*-(4-(trifluoromethyl)benzyl)butan-1-amine (**9d**) (0.14 g; 2.70·10^{-4} mol) and triethylamine (0.14 g; 1.35·10^{-3} mol) in 20 mL DCM. The ice bath was removed and the reaction was stirred for eighteen hours at room temperature, then filtered. The precipitate was discarded. The filtrate was washed sequentially twice with 15 mL H_2O and twice with 15 mL brine. The combined organic phases were dried over Na_2SO_4, then the solvent was removed under vacuum and the crude product was purified by column chromatography (EtOAc/MeOH 9:1) to yield the pure product.

(*Z*)-2,3-di(*tert*-butoxycarbonyl)-1-{4-{4-{[4-(phenoxymethyl)cyclohexyl]methyl}piperazin-1-yl}but-1-yl}-1-[4-(trifluoromethyl)benzyl]guanidine (**10d**): $C_{41}H_{60}F_3N_5O_5$. M=759.94. Colorless sticky oil. 84.36 % yield. R_F=0.37 (EtOAc/MeOH 9:1). ^{1}H NMR (600 MHz, *CDCl$_3$*) δ ppm 9.45 (1H, N<u>H</u>), 7.59-7.58 (m, 2H$^{arom.}$, C(CHC<u>H</u>)$_2$CCF$_3$), 7.43-7.41 (m, 2H$^{arom.}$, C(C<u>H</u>CH)$_2$CCF$_3$), 7.27-7.25 (m, 2H$^{phenoxy.}$, C(CHC<u>H</u>)$_2$CH), 6.93-6.88 (m, 3H$^{phenoxy.}$, C(C<u>H</u>CH)$_2$C<u>H</u>), 4.76 (br, 2H, CF$_3$PhC<u>H</u>$_2$), 3.85 (d, 2H, OC<u>H</u>$_2$, *J*=6.94 Hz), 3.33 (br, 2H, CH$_2$C<u>H</u>$_2$C(N)N), 2.41 (br, 8H^{piperazine}), 2.29-2.27 (m, 2H, CH$_2$C<u>H</u>$_2$N^{piperazine}), 2.22 (d, 2H, CHC<u>H</u>$_2$N^{piperazine}, *J*=7.32Hz), 2.01-1.97 (m, 1H$^{cyclohexyl.}$, OCH$_2$C<u>H</u>), 1.75-1.71 (m, 1H$^{cyclohexyl.}$, C<u>H</u>CH$_2$N^{piperazine}), 1.62-1.36 (m, 30H: 8H$^{cyclohexyl.}$: CH$_2$^{cyclohexyl}; CH$_2$C<u>H</u>$_2$CH$_2$, C<u>H</u>$_3$). ^{13}C NMR (150.95 MHz, *CDCl$_3$*) δ ppm 162.59 (1C, <u>C</u>=O), 159.31 (1C$^{quat/phenoxy.}$, <u>C</u>O), 156.16 (1C, <u>C</u>=N), 150.94 (1C, <u>C</u>=O), 140.74 (1C$^{quat./arom.}$, <u>C</u>CH$_2$NC(N)), 130.09, 129.87, 129.65, 129.43 (1C, <u>C</u>CF$_3$), 129.36 (2C$^{phenoxy.}$, C(CH<u>C</u>H)$_2$CH), 128.01 (2C$^{arom.}$, C(<u>C</u>HCH)$_2$CCF$_3$), 126.82, 125.01, 123.21, 121.43 (1C, <u>C</u>F$_3$), 125.59, 125.56 (2C$^{arom.}$, C(CH<u>C</u>H)$_2$CCF$_3$), 120.43 (1C$^{phenoxy.}$, C(CHCH)$_2$<u>C</u>H), 114.59 (2C$^{phenoxy.}$, C(<u>C</u>HCH)$_2$CH), 82.13 (1C$^{quat.}$ Boc), 79.62 (1C$^{quat.}$ Boc), 71.17 (1C O<u>C</u>H$_2$), 62.55 (1C, CH<u>C</u>H$_2$N^{piperazine}), 57.94 (1C, CH$_2$<u>C</u>H$_2$N^{piperazine}), 53.61 (2C$^{pip.}$), 53.25 (2C$^{pip.}$), 50.95 (1C, Ph<u>C</u>H$_2$), 48.12 (1C, CH$_2$<u>C</u>H$_2$C(N)N), 35.38 (1C^{cyclohexyl}, OCH$_2$<u>C</u>H), 32.32 (1C^{cyclohexyl}, <u>C</u>HCH$_2$N^{piperazine}), 28.17 (6C, <u>C</u>H$_3$), 27.29 (2C$^{cyclohexyl.}$, <u>C</u>H$_2$), 25.72 (2C$^{cyclohexyl.}$, <u>C</u>H$_2$), 25.24 (1C, CH$_2$<u>C</u>H$_2$CH$_2$), 23.82 (1C, CH$_2$<u>C</u>H$_2$CH$_2$).

Preparation of (*E)*-1-{4-{4-{[4-(phenoxymethyl)cyclohexyl]methyl}piperazin-1-yl}but-1-yl}-1-(benzyl)guanidine (**ADS-10283**)

4M solution HCl-dioxan (3.32 mL; 1.33·10^{-2} mol) was added dropwise to a solution of the (*E)*-2,3-di(*tert*-butoxycarbonyl)-1-{4-{4-{[4-(phenoxymethyl)cyclohexyl]methyl}piperazin-1-yl}but-1-yl}-1-(benzyl)guanidine (**10a**) (0.46 g; 6.65·10^{-4} mol) in 40 mL chloroform. The reaction was stirred overnight at room temperature, then the solvent was removed under vacuum. The crude product was evaporated twice from chloroform and twice from EtOAc, then recrystallized from anhydrous ethanol to yield the pure product.

(*E)*-1-{4-{4-{[4-(phenoxymethyl)cyclohexyl]methyl}piperazin-1-yl}but-1-yl}-1-(benzyl)guanidine (**ADS-10283**): $C_{30}H_{45}N_5O·3HCl·0.5H_2O$. M=610.10. White solid. 98.00 %. mp: 228.9-230.9 °C with decomposition. ^{1}H NMR (600 MHz, *D$_2$O*) δ ppm 7.43-7.41 (m, 2H$^{arom.}$, C(CHC<u>H</u>)$_2$CH), 7.37-7.32 (m, 3H: 2H$^{phenoxy.}$, C(CHC<u>H</u>)$_2$CH, 1H$^{arom.}$, C(CHCH)$_2$C<u>H</u>), 7.27-7.26 (m, 2H$^{arom.}$, C(C<u>H</u>CH)$_2$CH), 7.02-6.97 (m, 3H$^{phenoxy.}$, C(C<u>H</u>CH)$_2$C<u>H</u>), 4.61 (br, 2H, PhC<u>H</u>$_2$), 3.86 (d, 2H, OC<u>H</u>$_2$, *J*=6.50Hz), 3.62 (br, 8H^{piperazine}), 3.42-3.39 (m, 2H, CH$_2$C<u>H</u>$_2$C(N)N, *J*=7.03Hz), 3.22-3.19 (m, 2H, CH$_2$C<u>H</u>$_2$N^{piperazine}), 3.13 (d, 2H, CHC<u>H</u>$_2$N^{piperazine}, *J*=6.92Hz), 1.89-1.87 (2H$^{cyclohexyl.}$, C<u>H</u>$_2$), 1.84-1.83 (m, 1H$^{cyclohexyl.}$, C<u>H</u>CH$_2$N^{piperazine}), 1.81-1.79 (m, 2H$^{cyclohexyl.}$, C<u>H</u>$_2$), 1.73-1.67 (m, 5H: 1H^{cyclohexyl}, OCH$_2$C<u>H;</u> CH$_2$C<u>H</u>$_2$CH$_2$), 1.15.1.01 (m, 4H$^{cyclohexyl.}$, C<u>H</u>$_2$). ^{13}C NMR (150.95 MHz, *D$_2$O*) δ ppm 158.22 (1C$^{quat/phenoxy.}$, <u>C</u>O),

156.59 (1C, $\underline{C}$=N), 134.76 ($1C^{arom.}$, $\underline{C}CH_2NC(N)$), 129.89 ($2C^{phenoxy}$, $C(CH\underline{C}H)_2CH$), 129.09 (m, $2C^{arom.}$, $C(CH\underline{C}H)_2CH$), 128.09 ($1C^{arom.}$, $C(CHCH)_2\underline{C}H$), 126.66 ($2C^{arom.}$, $C(\underline{C}HCH)_2CH$), 121.46 ($1C^{phenoxy}$, $C(CHCH)_2\underline{C}H$), 115.02 ($2C^{phenoxy}$, $C(\underline{C}HCH)_2CH$), 73.54 (1C $O\underline{C}H_2$), 62.67 (1C, $CH\underline{C}H_2N^{piperazine}$), 56.31 (1C, $CH_2\underline{C}H_2N^{piperazine}$), 51.81 (1C, $Ph\underline{C}H_2$), 48.90 ($2C^{piperazine}$), 48.36 (1C, $CH_2\underline{C}H_2C(N)N$), 48.31 ($2C^{piperazine}$), 36.31 ($1C^{cyclohexyl}$, $OCH_2\underline{C}H$), 32.10 ($1C^{cyclohexyl}$, $\underline{C}HCH_2N^{piperazine}$), 29.09 ($2C^{cyclohexyl.}$, $\underline{C}H_2$), 27.95 ($2C^{cyclohexyl.}$, $\underline{C}H_2$), 23.52 (1C, $CH_2\underline{C}H_2CH_2$), 20.63 (1C, $CH_2\underline{C}H_2CH_2$). Anal. Calcd: C 59.06%; H 8.10%; N 11.48%. Found: C, 58.78%; H 8.34%; N 11.26%.

Preparation of (*E)*-1-{4-{4-{[4-(phenoxymethyl)cyclohexyl]methyl}piperazin-1-yl}but-1-yl}-1-[4-(trifluoromethyl)benzyl]guanidine (**ADS-10207**)

4M solution HCl-dioxan (0.5 mL; $2{\cdot}10^{-3}$ mol) was added dropwise to a solution of the (*E)*-2,3-di(*tert*-butoxycarbonyl)-1-{4-{4-{[4-(phenoxymethyl)cyclohexyl]methyl}piperazin-1-yl}but-1-yl}-1-[4-(trifluoromethyl)benzyl]guanidine (**10b**) (0.12 g; $1.58{\cdot}10^{-4}$ mol) in 10 mL chloroform. The reaction was stirred overnight at room temperature, then the solvent was removed under vacuum. The crude product was evaporated twice from chloroform and twice from EtOAc, then recrystallized from anhydrous ethanol to yield the pure product.

(*E)*-1-{4-{4-{[4-(phenoxymethyl)cyclohexyl]methyl}piperazin-1-yl}but-1-yl}-1-[4-(trifluoromethyl)benzyl]guanidine (**ADS-10207**): $C_{31}H_{44}F_3N_5O{\cdot}3HCl{\cdot}0.5H_2O$. M=678.12. White solid. 59.81 %. mp: 248.4-250.4 °C with decomposition. ^{1}H NMR (600 MHz, *D₂O*) δ ppm 7.71-7.70 (m, $2H^{arom.}$, $C(CHC\underline{H})_2CCF_3$), 7.40-7.39 (m, $2H^{arom.}$, $(C\underline{H}CH)_2CCF_3$), 7.33-7.31 (m, $2H^{phenoxy.}$, $C(CHC\underline{H})_2CH$), 7.00-6.96 (m, $3H^{phenoxy.}$, $C(C\underline{H}CH)_2C\underline{H}$), 4.65 (s, 2H, $CF_3PhC\underline{H}_2$), 3.86 (d, 2H, $OC\underline{H}_2$, *J*=6.44Hz), 3.57 (m, $8H^{piperazine}$), 3.41 (m, 2H, $CH_2C\underline{H}_2NC(N)$), 3.17 (m, 2H, $CH_2C\underline{H}_2N^{piperazine}$), 3.11 (d, 2H, $CHC\underline{H}_2N^{piperazine}$, *J*=6.61Hz), 1.88-1.67 (m, 10H: $6H^{cyclohexyl.}$ $C\underline{H}_2$, $C\underline{H}$; $CH_2C\underline{H}_2CH_2$), 1.11-1.02 ($4H^{cyclohexyl.}$, $C\underline{H}_2$). ^{13}C NMR (150.95 MHz, *D₂O*) δ ppm 158.27 ($1C^{quat/phenoxy.}$, $\underline{C}O$), 156.76 (1C, $\underline{C}$=N), 139.11 ($1C^{quat./arom.}$, $\underline{C}CH_2NC(N)$), 129.91 ($2C^{phenoxy}$, $CH(\underline{C}HCH)_2C$), 129.72, 129.57, 129.36, 129.19 ($1C^{quat./arom.}$, $\underline{C}CF_3$), 125.93 125.91 (4C, $C(\underline{C}H\underline{C}H)_2CCF_3$), 126.06, 124.64, 123.22, 121.79 (1C, $\underline{C}F_3$), 121.51 ($1C^{phenoxy}$, $C(CHCH)_2\underline{C}H$), 115.10 ($2C^{phenoxy}$, $C(\underline{C}HCH)_2CH$), 73.63 (1C $O\underline{C}H_2$), 62.68 (1C, $CH\underline{C}H_2N^{piperazine}$), 56.30 (1C, $CH_2\underline{C}H_2N^{piperazine}$), 51.54 (1C, $CF_3Ph\underline{C}H_2$), 49.02 ($2C^{piperazine}$), 48.63 (1C, $CH_2\underline{C}H_2C(N)N$), 48.38 ($2C^{piperazine}$), 36.35 ($1C^{cyclohexyl}$, $OCH_2\underline{C}H$), 32.18 ($1C^{cyclohexyl}$, $OCH_2\underline{C}H$), 29.15 ($2C^{cyclohexyl.}$, $\underline{C}H_2$), 27.97 ($2C^{cyclohexyl.}$, $\underline{C}H_2$), 23.59 (1C, $CH_2\underline{C}H_2CH_2$), 20.73 (1C, $CH_2\underline{C}H_2CH_2$). Anal. Calcd: C 54.91%; H 7.13%; N 10.33%. Found: C, 54.76%; H 7.35%; N 10.29%.

Preparation of (*Z*)-1-{4-{4-{[4-(phenoxymethyl)cyclohexyl]methyl}piperazin-1-yl}but-1-yl}-1-(benzyl)guanidine (**ADS-10227**)

4M solution HCl-dioxan (1.81 mL; $7.24{\cdot}10^{-3}$ mol) was added dropwise to a solution of the (*Z*)-2,3-di(*tert*-butoxycarbonyl)-1-{4-{4-{[4-(phenoxymethyl)cyclohexyl]methyl}piperazin-1-yl}but-1-yl}-1-(benzyl)guanidine (**10c**) (0.25 g; $3.61{\cdot}10^{-4}$ mol) in 20 mL chloroform. The reaction was stirred overnight at room temperature, then the solvent was removed under vacuum. The crude product was evaporated twice from chloroform and twice from EtOAc, then recrystallized from anhydrous ethanol to yield the pure product.

(*Z*)-1-{4-{4-{[4-(phenoxymethyl)cyclohexyl]methyl}piperazin-1-yl}but-1-yl}-1-(benzyl)guanidine (**ADS-10227**): $C_{30}H_{45}N_5O{\cdot}3HCl{\cdot}H_2O$. M=619.12. White solid. 65.93 %. mp: 213.9-215.9 °C with decomposition. ^{1}H NMR (600 MHz, *D₂O*) δ ppm 7.46-7.44 (m, $2H^{arom.}$, $C(CHC\underline{H})_2CH$), 7.41-7.36 (m, 3H: $2H^{phenoxy.}$, $C(CHC\underline{H})_2CH$; $1H^{arom.}$, $C(CHCH)_2C\underline{H}$), 7.30-7.29 (m, $2H^{arom.}$, $C(C\underline{H}CH)_2CH$), 7.05-7.02 (m, $3H^{phenoxy.}$, $C(C\underline{H}CH)_2C\underline{H}$), 4.64 (br, 2H, $PhC\underline{H}_2$), 4.00 (d, 2H, $OC\underline{H}_2$, *J*=7.11 Hz), 3.74-3.58 (br, $8H^{piperazine}$), 3.45-3.43 (m, 2H, $CH_2C\underline{H}_2C(N)N$, *J*=7.16Hz), 3.27 (d, 2H, $CHC\underline{H}_2N^{piperazine}$, *J*=7.17Hz), 3.24-3.21 (m, 2H, $CH_2C\underline{H}_2N^{piperazine}$), 2.14-2.08 (m, $1H^{cyclohexyl.}$, $OCH_2C\underline{H}$), 2.04-2.00 (m, $1H^{cyclohexyl.}$, $C\underline{H}CH_2N^{piperazine}$), 1.75-1.62 (m, 8H: $4H^{cyclohexyl.}$, $C\underline{H}_2$;

$CH_2C\underline{H}_2CH_2$), 1.54-1.43 (m, $4H^{cyclohexyl.}$, $C\underline{H}_2$). ^{13}C NMR (150.95 MHz, *D_2O*) δ ppm 158.32 ($1C^{quat./phenoxy.}$, $\underline{C}O$), 156.71 ($1C^{quat.}$, $\underline{C}$=N), 134.83 ($1C^{quat./arom.}$, $\underline{C}CH_2$), 129.16 ($2C^{phenoxy.}$, $C(CH\underline{C}H)_2CH$), 129.97 ($2C^{arom.}$,$C(CH\underline{C}H)_2CH$), 128.18 ($1C^{arom.}$, $C(CHCH)_2\underline{C}H$), 126.75 ($2C^{arom.}$, $C(\underline{C}HCH)_2CH$), 121.57 ($1C^{phenoxy}$, $C(CHCH)_2\underline{C}H$), 115.16 ($2C^{phenoxy}$, $C(\underline{C}HCH)_2CH$), 71.32 (1C, $OC\underline{H}_2$), 60.80 (1C, $CH\underline{C}H_2N^{piperazine}$), 56.39 (1C, $CH_2\underline{C}H_2N^{piperazine}$), 51.90 (1C, $PhC\underline{H}_2$), 49.03 ($2C^{piperazine}$), 48.99 ($2C^{piperazine}$), 48.43 (1C, $\underline{C}H_2C(N)N$), 33.92 ($1C^{cyclohexyl.}$, $OCH_2\underline{C}H$), 29.99 ($1C^{cyclohexyl.}$, $\underline{C}HCH_2N^{piperazine}$), 25.79 ($2C^{cyclohexyl.}$, $\underline{C}H_2$), 24.44 ($2C^{cyclohexyl.}$, $\underline{C}H_2$), 23.59 (1C, $CH_2\underline{C}H_2CH_2$), 20.72(1C, $CH_2\underline{C}H_2CH_2$). Anal. Calcd: C 58.20%; H 8.14%; N 11.31%. Found: C, 58.05%; H 8.12%; N 11.08%.

Preparation of (*Z*)-1-{4-{4-{[4-(phenoxymethyl)cyclohexyl]methyl}piperazin-1-yl}but-1-yl}-1-[4-(trifluoromethyl)benzyl]guanidine (**ADS-10239**)

4M solution HCl-dioxan (0.75 mL; $3{\cdot}10^{-3}$ mol) was added dropwise to a solution of the (*Z*)-2,3-di(*tert*-butoxycarbonyl)-1-{4-{4-{[4-(phenoxymethyl)cyclohexyl]methyl}piperazin-1-yl}but-1-yl}-1-[4-(trifluoromethyl)benzyl]guanidine (**10d**) (0.11 g; $1.45{\cdot}10^{-4}$ mol) in 20 mL chloroform. The reaction was stirred overnight at room temperature, then the solvent was removed under vacuum. The crude product was evaporated twice from chloroform and twice from EtOAc, then recrystallized from anhydrous 2-propanol to yield the pure product.

(*Z*)-1-{4-{4-{[4-(phenoxymethyl)cyclohexyl]methyl}piperazin-1-yl}but-1-yl}-1-[4-(trifluoromethyl)benzyl]guanidine (**ADS-10239**): $C_{31}H_{44}F_3N_5O{\cdot}3HCl{\cdot}0.5H_2O$. M=678.12. White solid. 99.00 %. mp: 226.6-229.0 °C with decomposition. ^1H NMR (600 MHz, *D_2O*) δ ppm 7.72-7.71 (m, $2H^{arom.}$, $C(CHC\underline{H})_2CCF_3$), 7.41-7.39 (m, $2H^{arom.}$, $C(C\underline{H}CH)_2CCF_3$), 7.34-7.32 (m, $2H^{phenoxy.}$, $C(CHC\underline{H})_2CH$), 7.02-6.98 (m, $3H^{phenoxy.}$, $C(C\underline{H}CH)_2C\underline{H}$), 4.67 (s, 2H, $CF_3PhC\underline{H}_2$), 3.97 (d, 2H, $OC\underline{H}_2$, *J*=7.11 Hz), 3.60 (m, $8H^{piperazine}$), 3.44-3.41 (m, 2H, $CH_2C\underline{H}_2NC(N)$), 3.23-3.17 (m, 4H, $CH_2C\underline{H}_2N^{piperazine}$, $CHC\underline{H}_2N^{piperazine}$), 2.09-2.05 (m, $1H^{cyclohexyl.}$, $OCH_2C\underline{H}$), 2.00-1.98 (m, $1H^{cyclohexyl.}$, $C\underline{H}CH_2N^{piperazine}$), 1.71-1.58 (m, 8H: $4H^{cyclohexyl.}$ $C\underline{H}_2$, $4H^{alif.}$, $CH_2C\underline{H}_2CH_2$), 1.50-1.39 ($4H^{cyclohexyl.}$, $C\underline{H}_2$). ^{13}C NMR (150.95 MHz, *$CDCl_3$*) δ ppm 158.30 ($1C^{quat/phenoxy.}$ $\underline{C}O$), 156.79 (1C, $\underline{C}$=N), 139.14 ($1C^{quat./arom.}$, $\underline{C}CH_2NC(N)$), 129.95 ($2C^{phenoxy}$, $CH(\underline{C}HCH)_2C$), 129.82, 129.59, 129.38, 129.16 (1C, $\underline{C}CF_3$), 127.54, 126.34, 125.05, 123.59 (1C, $\underline{C}F_3$), 127.01 ($2C^{arom.}$, $CH_2C(\underline{C}HCH)_2$), 125.96 125.94 ($2C^{arom.}$, $C(CH_2\underline{C}H_2)_2CCF_3$), 121.56 ($1C^{phenoxy}$, $C(CHCH)_2\underline{C}H$), 115.14 ($2C^{phenoxy}$, $C(\underline{C}HCH)_2CH$), 71.30 (1C $O\underline{C}H_2$), 60.80 (1C, $CH\underline{C}H_2N^{piperazine}$), 56.34 (1C, $CH_2\underline{C}H_2N^{piperazine}$), 51.56 (1C, $CF_3Ph\underline{C}H_2$), 49.01 ($2C^{piperazine}$), 48.65 (1C, $CH_2\underline{C}H_2C(N)N$),48.41 ($2C^{piperazine}$), 33.89 ($1C^{cyclohexyl}$, $OCH_2\underline{C}H$), 29.97 ($1C^{cyclohexyl}$, $OCH_2\underline{C}H$), 25.77 ($2C^{cyclohexyl.}$, $\underline{C}H_2$), 24.41 ($2C^{cyclohexyl.}$, $\underline{C}H_2$), 23.59 (1C, $CH_2\underline{C}H_2CH_2$), 20.72 (1C, $CH_2\underline{C}H_2CH_2$). Anal. Calcd: C 54.91%; H 7.13%; N 10.33%. Found: C, 55.01%; H 7.38%; N 10.20%.

Preparation of 1-(bromomethyl)-4-(phenoxymethyl)benzene (**11**)

Phenol (1.79 g; $1.90{\cdot}10^{-2}$ mol) was added to a sodium (0.44 g; $1.90{\cdot}10^{-2}$ mol) dissolved in anhydrous ethanol (50 mL) and the mixture was stirred for thirty minutes at room temperature. The solvent was removed under vacuum and the residue was dissolved in 50 mL of THF. The previously prepared sodium phenoxide solution was added dropwise to a solution of 1,4-Bis(bromomethyl)benzene (5.01 g; $1.90{\cdot}10^{-2}$ mol) in 100 mL anhydrous THF heated to 66 °C. The reaction was stirred overnight at 66 °C. The precipitate was discarded. The solvent was removed under vacuum and the crude product was purified by column chromatography (hexane/ EtOAc 70:1) to yield the pure product.

1-(bromomethyl)-4-(phenoxymethyl)benzene (**11**): $C_{14}H_{13}BrO$. M=277.16. White solid. 48.60 % yield. R_F=0.49 (hexane/EtOAc 70:1). mp: 59.3-61.3 °C. ^1H NMR (600 MHz, *$CDCl_3$*) δ ppm 7.40 (m, $4H^{benz}$), 7.29-7.27 (m, $2H^{phenoxy.}$, $C(CHC\underline{H})_2CH$), 6.97-6.95 (m, $3H^{phenoxy.}$, $C(C\underline{H}CH)_2C\underline{H}$), 5.05 (s, 2H, $OC\underline{H}_2$), 4.49 (s, 2H, $BrC\underline{H}_2$). ^{13}C NMR (150.95 MHz, *$CDCl_3$*) δ ppm 158.67 ($1C^{quat./phenoxy.}$, $\underline{C}O$), 137.49 ($1C^{quat./benz.}$), 137.45 ($1C^{quat./benz.}$), 129.51 ($2C^{phenoxy.}$, $C(CH\underline{C}H)_2CH$), 129.27 ($2C^{benz}$), 127.78 ($2C^{benz}$), 121.08($1C^{phenoxy.}$, $C(CHCH)_2\underline{C}H$), 114.86 ($2C^{phenoxy.}$, $C(\underline{C}HCH)_2CH$), 69.47 (1C, $O\underline{C}H_2$), 33.13 (1C, $Br\underline{C}H_2$).

Preparation of 1-(4-(phenoxymethyl)benzyl)piperazine (**12**)

1-(bromomethyl)-4-(phenoxymethyl)benzene (**11**) (1.00 g; $3.61\cdot10^{-3}$ mol) in 10 mL THF was added dropwise to a solution of piperazine (1.55 g; $1.80\cdot10^{-2}$ mol) in 20 mL THF heated to 66 °C. The reaction was stirred overnight at 66 °C. The solvent was removed under vacuum and the residue was diluted with 25 mL water, alkalized with 5 % NaOH solution and extracted 3x20 mL with DCM. The combined organic phases were dried over anhydrous Na_2SO_4. The solvent was removed under vacuum and the crude product was purified by column chromatography (DCM/MeOH/25% NH_3aq. 89:10:1) to yield the pure product.

1-(4-(phenoxymethyl)benzyl)piperazine (**12**): $C_{18}H_{22}N_2O$. M=282.39. White solid. 89.07 % yield. R_F=0.74 (DCM/MeOH/25% NH_3aq. 89:10:1). mp: 106.3-107.0 °C. ^{1}H NMR (600 MHz, *CDCl$_3$*) δ ppm 7.39-7.37 (m, 2H^{benz}), 7.34-7.33 (m, 2H^{benz}), 7.29-7.27 (m, 2H$^{phenoxy.}$, $C(CHC\underline{H})_2CH$), 6.98-6.94 (m, 3H$^{phenoxy.}$, $C(C\underline{H}CH)_2C\underline{H}$), 5.04 (s, 2H, $OC\underline{H}_2$), 3.49 (s, 2H, $PhC\underline{H}_2N^{piperazine}$), 2.91-2.89 (m, 4H^{piperazine}), 2.43 (br, 4H^{piperazine}), 2.38 (br, 1H, N$\underline{H}$*). ^{13}C NMR (150.95 MHz, *CDCl$_3$*) δ ppm 158.86 (1C$^{quat./phenoxy.}$, $\underline{C}$O), 137.94 (1C$^{quat./benz.}$), 135.86 (1C$^{quat./benz.}$), 129.46 (2C$^{phenoxy.}$, $C(CH\underline{C}H)_2CH$), 129.37 (2C^{benz}), 127.46 (2C^{benz}), 120.93 (1C$^{phenoxy.}$, $C(CHCH)_2\underline{C}H$), 114.88 (2C$^{phenoxy.}$, $C(\underline{C}HCH)_2CH$), 69.82 (1C, $O\underline{C}H_2$), 63.28 (1C, $Ph\underline{C}H_2N^{piperazine}$), 54.27 (2C^{piperazine}), 45.99 (2C^{piperazine}).

Preparation of 4-(4-(4-(phenoxymethyl)benzyl)piperazin-1-yl)butanenitrile (**13**)

Potassium carbonate (5.11 g; $3.70\cdot10^{-2}$ mol) and 4-bromobutyronitrile (1.42 g; $9.62\cdot10^{-3}$ mol) was added to a solution of 1-(4-(phenoxymethyl)benzyl)piperazine (**12**) (2.09 g; $7.40\cdot10^{-3}$ mol) in 90 mL acetonitrile. The reaction was stirred overnight at 80 °C, then filtered. The precipitate was discarded. The solvent was removed under vacuum and the crude product was purified by column chromatography (DCM/MeOH 24:1) to yield the pure product.

4-(4-(4-(phenoxymethyl)benzyl)piperazin-1-yl)butanenitrile (**13**): $C_{22}H_{27}N_3O$. M=349.48. White solid. 88.40 % yield. R_F=0.32 (DCM/MeOH 24:1). mp: 104.9-105.4 °C. ^{1}H NMR (600 MHz, *CDCl$_3$*) δ ppm 7.38-7.37 (m, 2H$^{benz.}$), 7.33-7.32 (m, 2H$^{benz.}$), 7.29-7.27 (m, 2H$^{phenoxy.}$, $C(CHC\underline{H})_2CH$), 6.98-6.94 (m, 3H$^{phenoxy.}$, $C(C\underline{H}CH)_2C\underline{H}$), 5.04 (s, 2H, $OC\underline{H}_2$), 3.50 (s, 2H, $PhC\underline{H}_2N^{piperazine}$), 2.45-2.39 (m, 12H: 8H^{piperazine}; $CH_2C\underline{H}_2N^{piperazine}$; $C\underline{H}_2CN$), 1.80 (qt, 2H, $CH_2C\underline{H}_2CH_2$, *J*=6.98 Hz). ^{13}C NMR (150.95 MHz, *CDCl$_3$*) δ ppm 158.85 (1C$^{quat./phenoxy.}$, $\underline{C}$O), 137.99 (1C$^{quat./benz.}$), 135.86 (1C$^{quat./benz.}$), 129.46 (2C$^{phenoxy.}$, $C(CH\underline{C}H)_2CH$), 129.35 (2C^{benz}), 127.45 (2C^{benz}), 120.93 (1C$^{phenoxy.}$, $C(CHCH)_2\underline{C}H$), 119.74 (1C, CN), 114.87 (2C$^{phenoxy.}$, $C(\underline{C}HCH)_2CH$), 69.81 (1C, $O\underline{C}H_2$), 62.72 (1C, $Ph\underline{C}H_2N^{piperazine}$), 56.29 (1C, $CH_2\underline{C}H_2N^{piperazine}$), 53.09 (2C^{piperazine}), 53.06 (2C^{piperazine}), 22.82 (1C, $CH_2\underline{C}H_2CH_2$), 14.91 (1C, $\underline{C}H_2CN$).

Preparation of 4-(4-(4-(phenoxymethyl)benzyl)piperazin-1-yl)butan-1-amine (**14**)

$LiAlH_4$ (1.22 g; $3.22\cdot10^{-2}$ mol) was added to a solution of 4-(4-(4-(phenoxymethyl)benzyl)piperazin-1-yl)butanenitrile (**13**) (2.81 g; $8.04\cdot10^{-3}$ mol) in 100 mL anhydrous diethyl ether. The reaction was stirred overnight at room temperature, then the mixture was quenched by dropwise addition of water (16 eq.) and 10 % NaOH solution (16 eq.) stirred for two hours, then filtered. The precipitate was discarded. The organic layer was dried over Na_2SO_4, then the solvent was removed under vacuum and the crude product was purified by column chromatography (DCM/MeOH/25% NH_3aq. 49:10:1) to yield the pure product.

4-(4-(4-(phenoxymethyl)benzyl)piperazin-1-yl)butan-1-amine (**14**): $C_{22}H_{31}N_3O$. M=353.48. Yellowish waxy solid. 99.8 % yield. R_F=0.49 (DCM/MeOH/25% NH_3 aq. 49:10:1). mp: 85.5-87.5 °C. ^{1}H NMR (600 MHz, *CDCl$_3$*) δ ppm 7.38-7.37 (m, 2H^{benz}), 7.34-7.33 (m, 2H^{benz}), 7.31-7.26 (m, 2H$^{phenoxy.}$, $C(CHC\underline{H})_2CH$), 6.99-6.93 (m, 3H$^{phenoxy.}$, $C(C\underline{H}CH)_2C\underline{H}$), 5.04 (s, 2H, $OC\underline{H}_2$), 3.51 (s, 2H, $PhC\underline{H}_2N^{piperazine}$), 3.47 (s, 2H, $N\underline{H}_2$*), 2.69 (t, 2H, $C\underline{H}_2NH_2$, *J*=6.98Hz), 2.47-2.38 (br, 8H^{piperazine}), 2.36-2.31 (m, 2H·, $CH_2C\underline{H}_2N^{piperazine}$), 1.56-1.48 (m, 2H, $CH_2C\underline{H}_2CH_2$), 1.45-1.42 (m, 2H, $CH_2C\underline{H}_2CH_2$). ^{13}C NMR (150.95 MHz, *CDCl$_3$*) δ ppm 158.84 (1C$^{quat./phenoxy.}$, $\underline{C}$O), 138.02 (1C$^{quat./benz.}$), 135.81 (1C$^{quat./benz.}$), 129.44 (2C$^{phenoxy.}$, $C(CH\underline{C}H)_2CH$), 129.37 (2C^{benz}), 127.44 (2C^{benz}), 120.90 (1C$^{phenoxy.}$, $C(CHCH)_2\underline{C}H$), 114.86 (2C$^{phenoxy.}$, $C(\underline{C}HCH)_2CH$), 69.81 (1C, $O\underline{C}H_2$), 62.75 (1C, $Ph\underline{C}H_2N^{piperazine}$), 58.48 (1C, $CH_2\underline{C}H_2N^{piperazine}$), 53.22 (2C^{piperazine}), 53.08 (2C^{piperazine}), 42.04 (1C, $\underline{C}H_2NH_2$), 31.70(1C, $CH_2\underline{C}H_2CH_2$), 24.31(1C, $CH_2\underline{C}H_2CH_2$).

Preparation of *N*-(4-(4-(4-(phenoxymethyl)benzyl)piperazin-1-yl)butyl)benzamide (**15a**)

Benzoyl chloride (0.35 g; $2.49\cdot10^{-3}$ mol) in 10 mL DCM was added dropwise to a solution of 4-(4-(4-(phenoxymethyl)benzyl)piperazin-1-yl)butan-1-amine (**14**) (0.80 g; $2.26\cdot10^{-3}$ mol) and triethylamine (0.92 g; $9.05\cdot10^{-3}$ mol) in 40 mL DCM. The reaction was stirred for three hours at room temperature. The mixture was washed 3-times with 20 mL water and dried over Na_2SO_4. The solvent was removed under vacuum and the crude product was purified by column chromatography (DCM/MeOH/Triethylamine 144:5:1) to yield the pure product.

N-(4-(4-(4-(phenoxymethyl)benzyl)piperazin-1-yl)butyl)benzamide (**15a**): $C_{29}H_{35}N_3O_2$. M=457.60. White solid. 81.45 % yield. R_F=0.19 (DCM/MeOH/Triethylamine 144:5:1). mp: 110.8-112.3 °C. ^{1}H NMR (600 MHz, *CDCl3)* δ ppm 7.77-7.72 (m, m, $2H^{arom.}$), 7.50-7.46 (m, $1H^{arom.}$, $C(CHCH)_2C\underline{H}$), 7.44-7.39 (m, $2H^{arom.}$), 7.38-7.37 (m, $2H^{benz}$), 7.33-7.26 (m, 4H: $2H^{benz}$; $2H^{phenoxy.}$, $C(CHC\underline{H})_2CH$), 6.99-6.93 (m, $3H^{phenoxy.}$, $C(C\underline{H}CH)_2C\underline{H}$), 6.67 (br, 1H, $N\underline{H}$), 5.04 (s, 2H, $OC\underline{H_2}$), 3.48-3.45 (m, 4H, $C\underline{H_2}NH$, $PhC\underline{H_2}N^{piperazine}$), 2.44-2.37 (m, 10H: $8H^{piperazine}$, $CH_2\underline{C}H_2N^{piperazine}$), 1.67 (qt, 2H, $CH_2C\underline{H_2}CH_2$), 1.61 (qt, 2H, $CH_2C\underline{H_2}CH_2$) . ^{13}C NMR (150.95 MHz, *CDCl3)* δ ppm 167.71 ($1C^{quat.}$, C=O), 158.86 ($1C^{quat./phenoxy.}$, $\underline{C}O$), 137.98 ($1C^{quat./benz.}$), 135.88 ($1C^{quat./benz.}$), 135.14 ($1C^{quat./arom.}$), 131.20 ($1C^{arom.}$, $C(CHCH)_2\underline{C}H$), 129.46 ($2C^{phenoxy.}$, $C(CH\underline{C}H)_2CH$), 129.37 ($2C^{benz}$), 128.49 ($2C^{arom.}$, $C(CH\underline{C}H)_2CH$), 127.46 ($2C^{benz}$), 126.96 ($2C^{arom.}$, $C(\underline{C}HCH)_2CH$), 120.94 ($1C^{phenoxy.}$, $C(CHCH)_2\underline{C}H$), 114.89 ($2C^{phenoxy.}$, $C(\underline{C}HCH)_2CH$), 69.83 (1C, $O\underline{C}H_2$), 62.71 (1C, $Ph\underline{C}H_2N^{piperazine}$), 57.98 (1C, $CH_2\underline{C}H_2N^{piperazine}$), 53.23 ($2C^{piperazine}$), 52.93 ($2C^{piperazine}$), 39.96 (1C, $\underline{C}H_2NH$), 27.49 (1C, $CH_2\underline{C}H_2CH_2$), 24.51(1C, $CH_2\underline{C}H_2CH_2$).

Preparation of *N*-(4-(4-(4-(phenoxymethyl)benzyl)piperazin-1-yl)butyl)-4-(trifluoromethyl)benzamide (**15b**)

4-(Trifluoromethyl)benzoyl chloride (0.52 g; $2.49\cdot10^{-3}$ mol) in 10 mL DCM was added dropwise to a solution of 4-(4-(4-(phenoxymethyl)benzyl)piperazin-1-yl)butan-1-amine (**14**) (0.80 g; $2.26\cdot10^{-3}$ mol) and triethylamine (0.92 g; $9.05\cdot10^{-3}$ mol) in 40 mL DCM. The reaction was stirred for three hours at room temperature. The mixture was washed 3-times with 20 mL water and dried over Na_2SO_4. The solvent was removed under vacuum and the crude product was purified by column chromatography (DCM/MeOH/ Triethylamine 169:5:1) to yield the pure product.

N-(4-(4-(4-(phenoxymethyl)benzyl)piperazin-1-yl)butyl)-4-(trifluoromethyl)benzamide (**15b**): $C_{30}H_{34}F_3N_3O_2$. M=525.61. White solid. 64.10 % yield. R_F=0.25 (DCM/MeOH/Triethylamine 169:5:1). mp: 123.8-124.5 °C. ^{1}H NMR (600 MHz, *CDCl3)* δ ppm 7.89-7.86 (m, $2H^{arom.}$, $C(C\underline{H}CH)_2CCF_3$), 7.69-7.67 (m, $2H^{arom.}$, $C(CHC\underline{H})_2CCF_3$), 7.39-7.37 (m, $2H^{benz}$), 7.31-7.27 (m, 4H: $2H^{benz}$; $2H^{phenoxy.}$, $C(CHC\underline{H})_2CH$), 7.14 (br, 1H, $N\underline{H}$*), 6.98-6.96 (m, $3H^{phenoxy.}$, $C(C\underline{H}CH)_2C\underline{H}$), 5.04 (s, 2H, $OC\underline{H_2}$), 3.49-3.46 (m, 2H, $C\underline{H_2}NH$), 3.45 (s, 2H, $PhC\underline{H_2}N^{piperazine}$), 2.44-2.38 (m, 10H: $8H^{piperazine}$, $CH_2\underline{C}H_2N^{piperazine}$), 1.69 (qt, 2H, $C\underline{H_2}CH_2N^{piperazine}$), 1.62 (qt, 2H, $NHCH_2C\underline{H_2}$). ^{13}C NMR (150.95 MHz, *CDCl3)* δ ppm 166.53 ($1C^{quat.}$, C=O), 158.75 ($1C^{quat./phenoxy.}$, $\underline{C}O$), 138.42 ($1C^{quat./arom.}$, $\underline{C}C(O)$), 137.68 ($1C^{quat./benz.}$), 135.86 ($1C^{quat./benz.}$), 133.24, 133.02, 132.81, 132.59 ($1C^{quat./arom.}$, $\underline{C}CF_3$), 129.45 ($2C^{phenoxy.}$, $C(CH\underline{C}H)_2CH$), 129.34 ($2C^{benz}$), 127.51 ($2C^{arom.}$, $(\underline{C}HCH)_2CCF_3$), 127.50 ($2C^{benz}$), 126.39, 124.59, 122.78, 120.98 (1C, $\underline{C}F_3$), 125.52, 125.49 ($2C^{arom.}$, $(CH\underline{C}H)_2CCF_3$), 120.90 ($1C^{phenoxy.}$, $C(CHCH)_2\underline{C}H$), 114.77 ($2C^{phenoxy.}$, $C(\underline{C}HCH)_2CH$), 69.70 (1C, $O\underline{C}H_2$), 62.62 (1C, $Ph\underline{C}H_2N^{piperazine}$), 57.81 (1C,$CH_2\underline{C}H_2N^{piperazine}$), 53.11 ($2C^{piperazine}$), 52.65 ($2C^{piperazine}$), 42.00 (1C, $\underline{C}H_2NH$), 27.22 (1C, $\underline{C}H_2CH_2N^{piperazine}$), 24.33 (1C, $NHCH_2\underline{C}H_2$).

Preparation of 4-(4-(4-(phenoxymethyl)benzyl)piperazin-1-yl)-*N*-(benzyl)butan-1-amine (**16a**)

$LiAlH_4$ (0.24 g; $6.21\cdot10^{-3}$ mol) was added to a solution of *N*-(4-(4-(4-(phenoxymethyl)benzyl)piperazin-1-yl)butyl)benzamide (**15a**) (0.71 g; $1.55\cdot10^{-3}$ mol) in 50 mL anhydrous diethyl ether. The reaction was stirred overnight at room temperature, then the mixture was quenched by dropwise addition of water (16 eq.) and 10 % NaOH solution (16 eq.) stirred for two hours, then filtered. The precipitate was discarded. The organic layer was dried over Na_2SO_4, then the solvent was removed under vacuum and the crude product was purified by column chromatography (EtOAc/MeOH/Triethylamine 49:10:1) to yield the pure product.

4-(4-(4-(phenoxymethyl)benzyl)piperazin-1-yl)-*N*-(benzyl)butan-1-amine (**16a**): $C_{29}H_{37}N_3O$. M=443.60. Sticky oil. 72.80 % yield. R_F=0.28 (EtOAc/MeOH/Triethylamine 49:10:1). ^{1}H NMR (600 MHz, *CDCl3)* δ ppm 7.38-7.36 (m, $2H^{benz}$), 7.34-7.22 (m, 9H: $2H^{phenoxy.}$, $C(CHC\underline{H})_2CH$; $2H^{benz.}$; $5H^{arom.}$, $C(C\underline{H}C\underline{H})_2C\underline{H}$), 6.99-6.93 (m, $3H^{phenoxy.}$, $C(C\underline{H}CH)_2C\underline{H}$), 5.04 (s, 2H, $OC\underline{H_2}$), 3.79 (s, 2H, $PhC\underline{H_2}NH$), 3.49 (s, 2H, $PhC\underline{H_2}N^{piperazine}$), 2.65 (t, 2H, $NHC\underline{H_2}CH_2$, *J*=6.49Hz), 2.46-2.33 (m, 10H: $8H^{piperazine}$; $CH_2C\underline{H_2}N^{piperazine}$), 1.96 (br. 1H, $N\underline{H}$*), 1.54-1.53 (m, 4H, $CH_2C\underline{H_2}CH_2$). ^{13}C NMR (150.95 MHz, *CDCl3)* δ ppm 158.85 ($1C^{quat./phenoxy.}$, $\underline{C}O$), 140.56 ($1C^{quat./arom.}$), 138.08 ($1C^{quat./benz.}$), 135.78 ($1C^{quat./benz.}$), 129.43 ($2C^{phenoxy.}$, $C(CH\underline{C}H)_2CH$), 129.35 ($2C^{benz}$), 128.34 ($2C^{arom.}$,

$C(CH\underline{C}H)_2CH)$, 128.06 ($2C^{arom.}$, $C(\underline{C}HCH)_2CH)$, 127.42 ($2C^{benz}$), 126.83 ($1C^{arom.}$, $C(CHCH)_2\underline{C}H)$, 120.89 ($1C^{phenoxy.}$, $C(CHCH)_2\underline{C}H)$, 114.86 ($2C^{phenoxy.}$, $C(\underline{C}HCH)_2CH)$, 69.81 (1C, $O\underline{C}H_2$), 62.76 (1C, $Ph\underline{C}H_2N^{piperazine}$), 58.55 (1C, $CH_2\underline{C}H_2NH$), 54.04 (1C, $Ph\underline{C}H_2NH$), 53.25 ($2C^{piperazine}$), 53.13 ($2C^{piperazine}$), 49.32 (1C,$CH_2\underline{C}H_2N^{piperazine}$), 28.14 (1C, $\underline{C}H_2CH_2N^{piperazine}$), 24.76 (1C, $NHCH_2\underline{C}H_2$).

Preparation of 4-(4-(4-(phenoxymethyl)benzyl)piperazin-1-yl)-*N*-(4-(trifluoromethyl)benzyl)butan-1-amine (**16b**)

$LiAlH_4$ (0.22 g; $5.78\cdot10^{-3}$ mol) was added to a solution of *N*-(4-(4-(4-(phenoxymethyl)benzyl)piperazin-1-yl)butyl)-4-(trifluoromethyl)benzamide (**15b**) (0.76 g; $1.45\cdot10^{-3}$ mol) in 50 mL anhydrous diethyl ether. The reaction was stirred overnight at room temperature, then the mixture was quenched by dropwise addition of water (16 eq.) and 10 % NaOH solution (16 eq.) stirred for two hours, then filtered. The precipitate was discarded. The organic layer was dried over Na_2SO_4, then the solvent was removed under vacuum and the crude product was purified by column chromatography (DCM/MeOH/Triethylamine 144:5:1) to yield the pure product.

4-(4-(4-(phenoxymethyl)benzyl)piperazin-1-yl)-*N*-(4-(trifluoromethyl)benzyl)butan-1-amine (**16b**): $C_{30}H_{36}F_3N_3O$. M=511.61. White solid. 84.52 % yield. R_F=0.30 (DCM/MeOH/Triethylamine 144:5:1). mp: 68.5-70.5 °C. ^1H NMR (600 MHz, *CDCl₃*) δ ppm 7.58-7.57 (m, $2H^{arom.}$, $C(CHC\underline{H})_2CCF_3)$, 7.46-7.45 (m, $2H^{arom.}$, $C(C\underline{H}CH)_2CCF_3)$, 7.38-7.37 (m, $2H^{benz}$), 7.33-7.31 (m, $2H^{benz}$), 7.30-7.3-27 (m, $2H^{phenoxy.}$, $C(CHC\underline{H})_2CH)$, 6.98-6.94 (m, $3H^{phenoxy.}$, $C(C\underline{H}CH)_2C\underline{H})$, 5.04 (s, 2H, $OC\underline{H_2}$), 3.85 (s, 2H, $CF_3PhC\underline{H_2}$), 3.50 (s, 2H, $PhC\underline{H}_2N^{piperazine}$), 2.71-2.28 (m, 12H: $8H^{piperazine}$; $CH_2C\underline{H}_2N^{piperazine}$; $NHC\underline{H_2}CH_2$), 2.06 (br. 1H, $N\underline{H}$*), 1.55 (m, 4H, $CH_2C\underline{H_2}CH_2$). ^{13}C NMR (150.95 MHz, *CDCl₃*) δ ppm 158.77 ($1C^{quat./phenoxy.}$, $\underline{C}O$), 144.63 ($1C^{quat./arom.}$, $\underline{C}CH_2$), 137.96 ($1C^{quat./benz.}$), 135.74 ($1C^{quat./benz.}$), 129.43 ($2C^{phenoxy.}$, $C(CH\underline{C}H)_2CH)$, 129.42, 129.20, 128.99, 128.77 ($1C^{quat./arom.}$, $\underline{C}CF_3$), 129.39 ($2C^{benz}$), 128.21 ($2C^{arom.}$, $C(\underline{C}HCH)_2CCF_3)$, 127.46 ($2C^{benz}$), 126.94, 125.14, 123.34, 121.53 (1C, $\underline{C}F_3$), 125.25, 125.23 ($2C^{arom.}$, $C(CH\underline{C}H)_2CCF_3)$, 120.87 ($1C^{phenoxy.}$, $C(CHCH)_2\underline{C}H)$, 114.78 ($2C^{phenoxy.}$, $C(\underline{C}HCH)_2CH)$, 69.73 (1C, $O\underline{C}H_2$), 62.75 (1C, $Ph\underline{C}H_2N^{piperazine}$), 58.49 (1C, $CH_2\underline{C}H_2NH$), 53.44 (1C, $CF_3Ph\underline{C}H_2$), 53.21 ($2C^{piperazine}$), 53.06 ($2C^{piperazine}$), 49.31 (1C,$CH_2\underline{C}H_2N^{piperazine}$), 28.08 (1C, $\underline{C}H_2CH_2N^{piperazine}$), 24.69 (1C, $NHCH_2\underline{C}H_2$).

Preparation of 2,3-di(*tert*-butoxycarbonyl)-1-(4-(4-(4-(phenoxymethyl)benzyl)piperazin-1-yl)butyl)-1-(benzyl)guanidine (**17a**)

1,3-bis(*tert*-butoxycarbonyl)-2-methylisothiourea (0.39 g; $1.34\cdot10^{-3}$ mol) and mercury II chloride (0.36 g; $1.34\cdot10^{-3}$ mol) were sequentially added to an ice-cooled mixture of 4-(4-(4-(phenoxymethyl)benzyl)piperazin-1-yl)-*N*-(benzyl)butan-1-amine (**16a**) (0.54 g; $1.22\cdot10^{-3}$ mol) and triethylamine (0.62 g; $6.09\cdot10^{-3}$ mol) in 15 mL DCM. The ice bath was removed and the reaction was stirred for eighteen hours at room temperature, then filtered. The precipitate was discarded. The filtrate was washed sequentially twice with 15 mL H_2O and twice with 15 mL brine. The combined organic phases were dried over Na_2SO_4, then the solvent was removed under vacuum and the crude product was purified by column chromatography (EtOAc/MeOH/Triethylamine 239:10:1) to yield the pure product.

2,3-di(*tert*-butoxycarbonyl)-1-(4-(4-(4-(phenoxymethyl)benzyl)piperazin-1-yl)butyl)-1-(benzyl)guanidine (**17a**): $C_{40}H_{55}N_5O_5$. M=753.89. Sticky oil. 87.40 % yield. R_F=0.33 (EtOAc/MeOH/Triethylamine 239:10:1). ^1H NMR (600 MHz, *CDCl₃*) δ ppm 9.90 (br, 1H, $N\underline{H}$), 7.39-7.36 (m, $2H^{benz}$), 7.34-7.22 (m, 9H: $2H^{phenoxy.}$, $C(CHC\underline{H})_2CH$; $2H^{benz.}$; $5H^{arom.}$, $C(C\underline{H}C\underline{H})_2C\underline{H})$, 6.98-6.94 (m, $3H^{phenoxy.}$, $C(C\underline{H}CH)_2C\underline{H})$, 5.04 (s, 2H, $OC\underline{H_2}$), 4.65 (br, 2H, $NC\underline{H_2}Ph$), 3.49 (s, 2H, $PhC\underline{H}_2N^{piperazine}$), 3.34 (m, 2H, $C\underline{H}_2NC(N)N$), 2.44 (m, $8H^{piperazine}$), 2.30-2.24 (m, 2H, $CH_2C\underline{H}_2N^{piperazine}$), 1.59-1.53 (m, 2H, $CH_2C\underline{H_2}CH_2$), 1.52-1.46 (m, 18H, $C\underline{H_3}$), 1.42 (m, 2H, $CH_2C\underline{H_2}CH_2$). ^{13}C NMR (150.95 MHz, *CDCl₃*) δ ppm 162.73 (1C, $\underline{C}$=O), 158.88 ($1C^{quat./phenoxy.}$, $\underline{C}O$), 155.96 ($1C^{quat.}$, $\underline{C}$=N), 150.98 (1C, $\underline{C}$=O), 138.11 ($1C^{quat./benz.}$), 136.47 ($1C^{quat./arom.}$), 135.81 ($1C^{quat./benz.}$), 129.46 ($2C^{phenoxy.}$, $C(CH\underline{C}H)_2CH)$, 129.37 ($2C^{benz}$), 128.64 ($2C^{arom.}$, $C(CH\underline{C}H)_2CH)$, 127.89 ($2C^{arom.}$, $C(\underline{C}HCH)_2CH)$, 127.56 ($1C^{arom.}$, $C(CHCH)_2\underline{C}H)$, 127.45 ($2C^{benz}$), 120.92 ($1C^{phenoxy.}$, $C(CHCH)_2\underline{C}H)$, 114.89 ($2C^{phenoxy.}$, $C(\underline{C}HCH)_2CH)$, 81.89 ($1C^{quat.}$ Boc), 79.37 ($1C^{quat.}$ Boc), 69.84 (1C, $O\underline{C}H_2$), 62.78 (1C, $Ph\underline{C}H_2N^{piperazine}$), 58.00 (1C, $CH_2\underline{C}H_2N^{piperazine}$), 53.17 ($2C^{piperazine}$), 53.13 ($2C^{piperazine}$), 31.26 (1C, $Ph\underline{C}H_2$), 28.61 (1C, $CH_2\underline{C}H_2NC(N)$), 28.26 (6C, $\underline{C}H_3$), 25.15 (1C, $CH_2\underline{C}H_2CH_2$), 23.91 (1C, $CH_2\underline{C}H_2CH_2$).

Preparation of 2,3-di(*tert*-butoxycarbonyl)-1-(4-(4-(4-(phenoxymethyl)benzyl)piperazin-1-yl)butyl)-1-(4-(trifluoromethyl)benzyl)guanidine (**17b**)

1,3-bis(*tert*-butoxycarbonyl)-2-methylisothiourea (0.29 g; $9.89\cdot10^{-4}$ mol) and mercury II chloride (0.27 g; $9.89\cdot10^{-4}$ mol) were sequentially added to an ice-cooled mixture of 4-(4-(4-(phenoxymethyl)benzyl)piperazin-1-yl)-*N*-(4-(trifluoromethyl)benzyl)butan-1-amine (**16b**) (0.46 g; $8.99\cdot10^{-4}$ mol) and triethylamine (0.45 g; $4.49\cdot10^{-3}$ mol) in 15 mL DCM. The ice bath was removed and the reaction was stirred for eighteen hours at room temperature, then filtered. The precipitate was discarded. The filtrate was washed sequentially twice with 15 mL H_2O and twice with 15 mL brine. The combined organic phases were dried over Na_2SO_4, then the solvent was removed under vacuum and the crude product was purified by column chromatography (EtOAc/MeOH/Triethylamine 239:10:1) to yield the pure product.

2,3-di(*tert*-butoxycarbonyl)-1-(4-(4-(4-(phenoxymethyl)benzyl)piperazin-1-yl)butyl)-1-(4-(trifluoromethyl)benzyl)guanidine (**17b**): $C_{41}H_{54}F_3N_5O_5$. M=753.89. Sticky oil. 82.20 % yield. R_F=0.36 (EtOAc/MeOH/Triethylamine 239:10:1). ^{1}H NMR (600 MHz *CDCl$_3$*) δ ppm 9.93 (br, 1H, N$\underline{H}$), 7.59-7.58 (m, 2H$^{arom.}$, C(CHC$\underline{H}$)$_2$CCF$_3$), 7.44-7.41 (m, 2H$^{arom.}$, C(C$\underline{H}$CH)$_2$CCF$_3$), 7.38-7.37 (m, 2H^{benz}), 7.33-7.31 (m, 2H^{benz}), 7.30-7.26 (m, 2H$^{phenoxy.}$, C(CHC$\underline{H}$)$_2$CH), 7.00-6.93 (m, 3H$^{phenoxy.}$, C(C$\underline{H}$CH)$_2$C$\underline{H}$), 5.04 (s, 2H, OC$\underline{H}_2$), 4.76 (br, 2H, CF$_3$PhC$\underline{H}_2$), 3.49 (s, 2H, PhC$\underline{H}_2$N^{piperazine}), 3.32 (m, 2H, CH$_2$C$\underline{H}_2$C(N)N), 2.44-2.43 (m, 8H^{piperazine}), 2.28 (t, 2H, CH$_2$C$\underline{H}_2$N^{piperazine}, J = 7.09 Hz), 1.59-1.54 (m, 2H, CH$_2$C$\underline{H}_2$CH$_2$), 1.49 (s, 18H, C$\underline{H}_3$), 1.43-1.40 (m, 2H, CH$_2$C$\underline{H}_2$CH$_2$). ^{13}C NMR (150.95 MHz, *CDCl$_3$*) δ ppm 162.59 (1C, $\underline{C}$=O), 158.77 (1C$^{quat./phenoxy.}$, $\underline{C}$O), 156.20 (1C$^{quat.}$, $\underline{C}$=N), 150.86 (1C, $\underline{C}$=O), 140.66 (1C$^{quat./arom.}$, $\underline{C}$CH$_2$NC(N)), 137.96 (1C$^{quat./benz.}$), 135.74 (1C$^{quat./benz.}$), 129.44 (2C$^{phenoxy.}$, C(CH$\underline{C}$H)$_2$CH), 130.01, 129.79, 129.57, 129.35 (1C$^{quat./arom.}$, $\underline{C}$CF$_3$), 129.37 (2C^{benz}), 127.96 (2C$^{arom.}$, C($\underline{C}$HCH)$_2$CCF$_3$), 127.45 (2C^{benz}), 126.78, 124.98, 123.17, 121.38 (1C, $\underline{C}$F$_3$), 125.56, 125.54 (2C$^{arom.}$, C(CH$\underline{C}$H)$_2$CCF$_3$), 120.88 (1C$^{phenoxy.}$, C(CHCH)$_2\underline{C}$H), 114.78 (2C$^{phenoxy.}$, C($\underline{C}$HCH)$_2$CH), 82.11 (1C$^{quat.}$ Boc), 79.59 (1C$^{quat.}$ Boc), 69.73 (1C, O$\underline{C}$H$_2$), 62.73 (1C, Ph$\underline{C}$H$_2$N^{piperazine}), 57.88 (1C, CH$_2\underline{C}$H$_2$N^{piperazine}), 53.12 (2C^{piperazine}), 53.04 (2C^{piperazine}), 50.77 (1C, CF$_3$Ph$\underline{C}$H$_2$), 48.01 (1C, CH$_2\underline{C}$H$_2$NC(N)), 28.11 (6C, $\underline{C}$H$_3$), 25.19 (1C, CH$_2\underline{C}$H$_2$CH$_2$), 23.84 (1C, CH$_2\underline{C}$H$_2$CH$_2$).

Preparation of 1-(4-(4-(4-(phenoxymethyl)benzyl)piperazin-1-yl)butyl)-1-(benzyl)guanidine (**ADS-10185**)

4M solution HCl-dioxan (4.84 g; $1.94\cdot10^{-2}$ mol) was added dropwise to a solution of the 2,3-di(*tert*-butoxycarbonyl)-1-(4-(4-(4-(phenoxymethyl)benzyl)piperazin-1-yl)butyl)-1-(benzyl)guanidine (**17a**) (0.73 g; $9.68\cdot10^{-4}$ mol) in 20 mL chloroform. The reaction was stirred overnight at room temperature, then the solvent was removed under vacuum. The crude product was evaporated twice from chloroform and twice from EtOAc, then recrystallized from anhydrous ethanol to yield the pure product.

1-(4-(4-(4-(phenoxymethyl)benzyl)piperazin-1-yl)butyl)-1-(benzyl)guanidine (**ADS-10185**): $C_{30}H_{39}N_5O\cdot3HCl\cdot H_2O$. M=613.08. White solid. 94.10 %. mp: 179.4-180.4 °C with decomposition. ^{1}H NMR (600 MHz *D$_2$O*) δ ppm 7.56-7.55 (m, 2H$^{benz.}$), 7.49-7.48 (m, 2H$^{benz.}$), 7.40-7.39 (m, 2H$^{arom.}$), 7.35-7.31 (m, 3H: 2H$^{phenoxy.}$, C(CHC$\underline{H}$)$_2$CH, 1H$^{arom.}$, C(CHCH)$_2$C$\underline{H}$), 7.26-7.25 (m, 2H$^{arom.}$), 7.03-7.02 (m, 3H$^{phenoxy.}$, C(C$\underline{H}$CH)$_2$C$\underline{H}$), 5.17 (s, 2H, OC$\underline{H}_2$), 4.59 (s, 2H, NC$\underline{H}_2$Ph), 4.38 (s, 2H, PhC$\underline{H}_2$N^{piperazine}), 3.59-3.39 (m, 10H: 8H^{piperazine}, CH$_2$C$\underline{H}_2$NC(N)), 3.16 (m, 2H, CH$_2$C$\underline{H}_2$N^{piperazine}) 1.65 (m, 4H, CH$_2$C$\underline{H}_2$CH$_2$). ^{13}C NMR (150.95 MHz, *D$_2$O*) δ ppm 157.69 (1C$^{quat./phenoxy.}$, $\underline{C}$O), 156.67 (1C$^{quat.}$, $\underline{C}$=N), 139.24 (1C$^{quat./arom.}$, $\underline{C}$(CHCH)$_2$CH), 134.79 (1C$^{quat./benz.}$), 131.55 (2C$^{benz.}$), 129.95 (2C$^{arom.}$, C(CH$\underline{C}$H)$_2$CH), 129.12 (2C$^{phenoxy.}$, C(CH$\underline{C}$H)$_2$CH), 128.75 (2C$^{benz.}$), 128.13 (1C$^{arom.}$, C(CHCH)$_2\underline{C}$H), 127.75 (1C$^{quat./benz.}$), 126.72 (m, 2H$^{arom.}$), 121.89 (1C$^{phenoxy.}$, C(CHCH)$_2\underline{C}$H), 115.34 (2C$^{phenoxy.}$, C($\underline{C}$HCH)$_2$CH), 69.57 (1C, OC$\underline{H}_2$), 60.18 (1C, Ph$\underline{C}$H$_2$N^{piperazine}), 56.25 (1C, CH$_2\underline{C}$H$_2$N^{piperazine}), 51.88 (1C, Ph$\underline{C}$H$_2$), 48.72 (1C, CH$_2\underline{C}$H$_2$NC(N)), 48.40 (2C^{piperazine}), 48.18 (2C^{piperazine}), 23.56 (1C, CH$_2\underline{C}$H$_2$CH$_2$), 20.69 (1C, CH$_2\underline{C}$H$_2$CH$_2$). Anal. Calcd: C 58.77%; H 7.23%; N 11.42%. Found: C, 58.86%; H 7.39%; N 11.18%.

Preparation of 1-(4-(4-(4-(phenoxymethyl)benzyl)piperazin-1-yl)butyl)-1-(4-(trifluoromethyl)benzyl)guanidine (**ADS-10183**)

4M solution HCl-dioxan (3.65 mL; $1.46 \cdot 10^{-2}$ mol) was added dropwise to a solution of the 2,3-di(*tert*-butoxycarbonyl)-1-(4-(4-(4-(phenoxymethyl)benzyl)piperazin-1-yl)butyl)-1-(4-(trifluoromethyl)benzyl)guanidine (**17b**) (0.55 g; $7.30 \cdot 10^{-4}$ mol) in 15 mL chloroform. The reaction was stirred overnight at room temperature, then the solvent was removed under vacuum. The crude product was evaporated twice from chloroform and twice from EtOAc, then recrystallized from anhydrous ethanol to yield the pure product.

1-(4-(4-(4-(phenoxymethyl)benzyl)piperazin-1-yl)butyl)-1-(4-(trifluoromethyl)benzyl)guanidine (**ADS-10183**): $C_{31}H_{38}F_3N_5O \cdot 3HCl$. M=633.06. White solid. 64.50 %. mp: 194.8-195.5 °C with decomposition. ^1H NMR (600 MHz *D_2O*) δ ppm 7.71-7.69 (m, $2H^{arom.}$, $C(CH\underline{CH})_2CCF_3$), 7.56-7.54 (m, $2H^{benz.}$), 7.49-7.48 (m, $2H^{benz.}$), 7.40-7.39 (m, $2H^{arom.}$,$C(C\underline{H}CH)_2CCF_3$), 7.32-7.31 (m, $2H^{phenoxy.}$, $C(CHC\underline{H})_2CH$), 7.03-7.01 (m, $3H^{phenoxy.}$, $C(C\underline{H}CH)_2C\underline{H}$), 5.17 (s, 2H, $OC\underline{H_2}$), 4.65 (s, 2H, $CF_3PhC\underline{H_2}$), 4.10 (s, 2H, $PhC\underline{H}_2N^{piperazine}$), 3.56 (br, $8H^{piperazine}$), 3.41 (m, 2H, $CH_2C\underline{H}_2NC(N)$), 3.19 (m, 2H, $CH_2C\underline{H}_2N^{piperazine}$) 1.67 (m, 4H, $CH_2C\underline{H}_2CH_2$). ^{13}C NMR (150.95 MHz, *D_2O*) δ ppm 157.66 ($1C^{quat./phenoxy.}$, $\underline{C}O$), 156.76 ($1C^{quat.}$, $\underline{C}$=N), 139.29 ($1C^{quat./arom.}$, $\underline{C}CH_2NC(N)$), 139.11 ($1C^{quat./benz.}$), 131.57 ($2C^{benz.}$), 129.95 ($2C^{phenoxy.}$, $C(CH\underline{C}H)_2CH$), 129.77, 129.56, 129.34, 129.12 ($1C^{arom}$, $\underline{C}CF_3$), 128.75 ($2C^{benz.}$), 127.79, 126.31, 125.01, 123.22 (1C, $\underline{C}F_3$), 127.53 ($1C^{quat./benz.}$), 126.99 ($2C^{arom.}$, $C(\underline{C}HCH)_2CCF_3$), 125.93, 125.91 ($2C^{arom.}$, $C(CH\underline{C}H)_2CCF_3$), 121.88 ($1C^{phenoxy.}$, $C(CHCH)_2\underline{C}H$), 115.33 ($2C^{phenoxy.}$, $C(\underline{C}HCH)_2CH$), 69.55 (1C, $OC\underline{H_2}$), 60.16 (1C, $Ph\underline{C}H_2N^{piperazine}$), 56.26 (1C, $CH_2\underline{C}H_2N^{piperazine}$), 51.55 (1C, $CF_3Ph\underline{C}H_2$), 48.67 ($2C^{piperazine}$), 48.63 ($2C^{piperazine}$), 48.13 (1C, $CH_2\underline{C}H_2NC(N)$), 23.56 (1C, $CH_2\underline{C}H_2CH_2$), 20.68 (1C, $CH_2\underline{C}H_2CH_2$). Anal. Calcd: C 56.16%; H 6.23%; N 10.56%. Found: C, 55.86%; H 6.32%; N 10.26%.

Preparation of 1-(4-phenoxybenzyl)piperazine (**18**)

1-(bromomethyl)-4-phenoxybenzene (0.50 g; $1.90 \cdot 10^{-3}$ mol) in 20 mL THF was added dropwise to a solution of piperazine (0.82 g; $9.50 \cdot 10^{-3}$ mol) in 30 mL anhydrous THF heated to 66 °C. The reaction was stirred overnight at 66 °C. The precipitate was discarded. The solvent was removed under vacuum and the residue was diluted by 40 mL of water, alkalized with 5 % NaOH solution and extracted 4x30 mL with DCM. The combined organic phases were dried over anhydrous Na_2SO_4. The solvent was removed under vacuum and the crude product was purified by column chromatography (DCM/MeOH/25% NH_3aq. 89:10:1) to yield the pure product.

1-(4-phenoxybenzyl)piperazine (**18**): $C_{17}H_{20}N_2O$. M=268.36. White solid. 98.05 % yield. R_F=0.20 (DCM/MeOH/25% NH_3aq. 139:10:1). mp: 43.0-45.0 °C. ^1H NMR (600 MHz, *$CDCl_3$*) δ ppm 7.36-7.29 (m, $2H^{phenoxy.}$, $C(CHC\underline{H})_2CH$), 7.27-7.26 (m, $2H^{benz.}$,$C(C\underline{H}CH)_2CO$), 7.09-7.07 (m, $1H^{phenoxy.}$, $C(CHCH)_2C\underline{H}$), 7.00-6.99 (m, $2H^{phenoxy.}$, $C(C\underline{H}CH)_2CH$), 6.96-6.94 (m, $2H^{benz.}$, $C(CHC\underline{H})_2CO$), 3.46 (s, 2H, $PhC\underline{H}_2N^{piperazine}$), 2.90 (t, 4 $H^{piperazine}$, *J*=4.85Hz), 2.43 (br, 4H $H^{piperazine}$), 2.19 (s, 1H, $N\underline{H}$). ^{13}C NMR (150.95 MHz, *$CDCl_3$*) δ ppm 157.33 ($1C^{quat./benz.}$, $\underline{C}O$), 156.22 ($1C^{quat./phenoxy.}$, $\underline{C}O$), 132.83 ($1C^{quat./benz.}$, $\underline{C}CH_2$), 130.47 ($2C^{benz.}$, $C(\underline{C}HCH)_2CO$), 129.64 ($2C^{phenoxy}$, $C(CH\underline{C}H)_2CH$), 123.07 ($1C^{phenoxy}$, $C(CHCH)_2\underline{C}H$), 118.72 ($2C^{phenoxy}$, $C(\underline{C}HCH)_2CH$), 118.59 ($2C^{benz.}$, $C(CH\underline{C}H)_2CO$), 62.94 (1C, $Ph\underline{C}H_2N^{piperazine}$), 54.20 ($2C^{piperazine}$), 45.93 ($2C^{piperazine}$).

Preparation of 4-(4-(4-phenoxybenzyl)piperazin-1-yl)butanenitrile (**19**)

Potassium carbonate (1.26 g; $9.13 \cdot 10^{-3}$ mol) and 4-bromobutyronitrile (0.35 g; $2.37 \cdot 10^{-3}$ mol) was added to a solution of 1-(4-phenoxybenzyl)piperazine (**18**) (0.49 g; $1.83 \cdot 10^{-3}$ mol) in 20 mL acetonitrile. The reaction was stirred overnight at 80 °C, then filtered. The precipitate was discarded. The solvent was removed under vacuum and the crude product was purified by column chromatography (EtOAc/MeOH/Triethylamine 89:10:1) to yield the pure product.

4-(4-(4-phenoxybenzyl)piperazin-1-yl)butanenitrile (**19**): $C_{21}H_{25}N_3O$. M=335.44. Light yellow oil. 94.69 % yield. R_F=0.54 (EtOAc/MeOH/Triethylamine 89:10:1). ^1H NMR (600 MHz, *$CDCl_3$*) δ ppm 7.33-7.31 (m, $2H^{phenoxy.}$, $C(CHC\underline{H})_2CH$), 7.27-7.26 (m, $2H^{benz.}$ $C(C\underline{H}CH)_2CO$), 7.09-7.07 (m, $1H^{phenoxy.}$, $C(CHCH)_2C\underline{H}$), 7.01-6.99 (m, $2H^{phenoxy,}$, $C(C\underline{H}CH)_2CH$), 6.97-6.93 (m, $2H^{benz.}$, $C(CHC\underline{H})_2CO$), 3.48 (s, 2H, $PhC\underline{H}_2N^{piperazine}$), 2.46-2.39 (m, 12H: $8H^{piperazine}$; $CH_2C\underline{H}_2N^{piperazine}$; $C\underline{H}_2CN$), 1.81 (qt, 2H, $CH_2C\underline{H}_2CH_2$, *J*=6.98Hz). ^{13}C NMR (150.95 MHz, *$CDCl_3$*) δ ppm 157.37 ($1C^{quat./benz.}$, $\underline{C}O$), 156.29 ($1C^{quat./phenoxy.}$, $\underline{C}O$), 132.97 ($1C^{quat./benz.}$, $\underline{C}CH_2$), 130.47 ($2C^{benz.}$, $C(\underline{C}HCH)_2CO$), 129.69 ($2C^{phenoxy}$, $C(CH\underline{C}H)_2CH$), 123.13 ($1C^{phenoxy}$, $C(CHCH)_2\underline{C}H$), 119.72 (1C, $\underline{C}N$), 118.78

($2C^{phenoxy}$, $C(\underline{C}HCH)_2CH$), 118.64 ($2C^{benz.}$, $C(\underline{C}HCH)_2CH$), 62.36 (1C, $Ph\underline{C}H_2N^{piperazine}$), 56.29 (1C, $CH_2\underline{C}H_2N^{piperazine}$), 53.09 ($2C^{piperazine}$), 52.99 ($2C^{piperazine}$), 22.82 (1C, $CH_2\underline{C}H_2CH_2$), 14.91 (1C, $\underline{C}H_2CN$).

Preparation of 4-(4-(4-phenoxybenzyl)piperazin-1-yl)butan-1-amine (**20**)

$LiAlH_4$ (0.24 g; $6.20\cdot10^{-3}$ mol) was added to a solution of 4-(4-(4-phenoxybenzyl)piperazin-1-yl)butanenitrile (**19**) (0.52 g; $1.55\cdot10^{-3}$ mol) in 50 mL anhydrous diethyl ether. The reaction was stirred overnight at room temperature, then the mixture was quenched by dropwise addition of water (16 eq.) and 10 % NaOH solution (16 eq.) stirred for two hours, then filtered. The precipitate was discarded. The organic layer was dried over Na_2SO_4, then the solvent was removed under vacuum and the crude product was purified by column chromatography (DCM/MeOH/25% NH_3 aq. 89:10:1) to yield the pure product.

4-(4-(4-phenoxybenzyl)piperazin-1-yl)butan-1-amine (**20**): $C_{21}H_{29}N_3O$. M=339.48. Sticky oil. 89.31 % yield. R_F=0.22 (DCM/MeOH/25% NH_3aq. 89:10:1). ^1H NMR (600 MHz, $CDCl_3$) δ ppm 7.35-7.29 (m, $2H^{phenoxy.}$, $C(CHC\underline{H})_2CH$), 7.29-7.24 (m, $2H^{benz.}$, $C(C\underline{H}CH)_2CO$), 7.09-7.07 (m, $1H^{phenoxy.}$, $C(CHCH)_2C\underline{H}$), 7.00-6.99 (m, $2H^{phenoxy.}$, $C(C\underline{H}CH)_2CH$), 6.96-6.94 (m, $2H^{benz.}$, $C(CHC\underline{H})_2CO$), 3.48 (s, 2H, $PhC\underline{H}_2N^{piperazine}$), 2.70 (t, 2H, $C\underline{H}_2NH_2$, *J*=6.89Hz), 2.48 (br, $8H^{piperazine}$), 2.36-2.33 (t, 2H, $CH_2C\underline{H}_2N^{piperazine}$), 1.54-1.49 (qt, 2H, $CH_2C\underline{H}_2CH_2$), 1.45 (qt, 2H, $CH_2C\underline{H}_2CH_2$), 1.33 (br, 2H, $N\underline{H}_2$ *). ^{13}C NMR (150.95 MHz, *$CDCl_3$*) δ ppm 157.42 ($1C^{quat./benz.}$, $\underline{C}O$), 156.22 ($1C^{quat./phenoxy.}$, $\underline{C}O$), 133.08 ($1C^{quat./benz.}$, $\underline{C}CH_2$), 130.51 ($2C^{benz.}$, $C(\underline{C}HCH)_2CO$), 129.68 ($2C^{phenoxy}$, $C(CH\underline{C}H)_2CH$), 123.09 ($1C^{phenoxy}$, $C(CHCH)_2\underline{C}H$), 118.75 ($2C^{phenoxy}$, $C(\underline{C}HCH)_2CH$), 118.65 ($2C^{benz.}$, $C(\underline{C}HCH)_2CH$), 62.44 (1C, $Ph\underline{C}H_2N^{piperazine}$), 58.56 (1C, $CH_2\underline{C}H_2N^{piperazine}$), 53.29 ($2C^{piperazine}$), 53.08 ($2C^{piperazine}$), 42.19 (1C, $\underline{C}H_2NH_2$), 31.91 (1C, $CH_2\underline{C}H_2CH_2$), 24.37 (1C, $CH_2\underline{C}H_2CH_2$) .

Preparation of *N*-(4-(4-(4-phenoxybenzyl)piperazin-1-yl)butyl)-4-(trifluoromethyl)benzamide (**21**)

4-(Trifluoromethyl)benzoyl chloride (0.16 g; $7.45\cdot10^{-4}$ mol) in 5 mL DCM was added dropwise to a solution of 4-(4-(4-phenoxybenzyl)piperazin-1-yl)butan-1-amine (**20**) (0.23 g; $6.77\cdot10^{-4}$ mol) and triethylamine (0.34 g; $3.39\cdot10^{-3}$ mol) in 10 mL DCM. The reaction was stirred for three hours at room temperature. The mixture was washed 3-times with 10 mL water and dried over Na_2SO_4. The solvent was removed under vacuum and the crude product was purified by column chromatography (EtOAc/MeOH/Triethylamine 89:10:1) to yield the pure product.

N-(4-(4-(4-phenoxybenzyl)piperazin-1-yl)butyl)-4-(trifluoromethyl)benzamide (**21**): $C_{29}H_{32}F_3N_3O_2$. M=511.58. White solid. 80.78 % yield. R_F=0.33 (EtOAc/MeOH/Triethylamine 89:10:1). mp: 119.0-121.0 °C. ^1H NMR (600 MHz, *$CDCl_3$*) δ ppm 7.88-7.86 (m, $2H^{arom.}$, $C(C\underline{H}CH)_2CCF_3$), 7.69-7.68 (m, $2H^{arom.}$, $C(CHC\underline{H})_2CCF_3$), 7.33-7.31 (m, $2H^{phenoxy.}$, $C(CHC\underline{H})_2CH$), 7.24-7.23 (m, $2H^{benz.}$, $C(C\underline{H}CH)_2CO$), 7.10-7.08 (m, $1H^{phenoxy.}$, $C\underline{H}CHCHC$), 7.04 (br, 1H, $N\underline{H}$), 7.00-6.99 (m, $2H^{phenoxy.}$, $C(C\underline{H}CH)_2CH$), 6.95-6.94 (m, $2H^{benz.}$, $C(CHC\underline{H})_2CO$), 3.49-3.46 (m, 2H, $C\underline{H}_2NH$), 3.42 (s, 2H, $PhC\underline{H}_2N^{piperazine}$), 2.49-2.42 (m, 10H: $8H^{piperazine}$, $CH_2C\underline{H}_2N^{piperazine}$), 1.72-1.62 (m, 4H, $CH_2C\underline{H}_2CH_2$). ^{13}C NMR (150.95 MHz, *$CDCl_3$*) δ ppm 166.52 ($1C^{quat.}$, C=O), 157.34 ($1C^{quat./benz.}$, $\underline{C}O$), 156.37 ($1C^{quat./phenoxy.}$, $\underline{C}O$), 138.50 ($1C^{quat./arom.}$, $\underline{C}C(O)$), 133.36, 133.14, 132.92, 132.57 ($1C^{quat./arom.}$, $\underline{C}CF_3$), 132.75 ($1C^{quat./benz.}$, $\underline{C}CH_2$), 130.48 ($2C^{benz.}$, $C(\underline{C}HCH)_2CO$), 129.72 ($2C^{phenoxy}$, $C(CH\underline{C}H)_2CH$), 127.55 ($2C^{arom.}$, $C(\underline{C}HCH)_2CCF_3$), 126.45, 124.65, 122.84, 121.05 (1C, $\underline{C}F_3$), 125.58, 125.56 ($2C^{arom.}$, $C(CH\underline{C}H)_2CCF_3$), 123.19 ($1C^{phenoxy.}$, $C(CHCH)_2\underline{C}H$), 118.81 ($2C^{phenoxy}$, $C(\underline{C}HCH)_2CH$), 118.67 ($2C^{benz.}$, $C(\underline{C}HCH)_2CH$), 62.31 (1C, $Ph\underline{C}H_2N^{piperazine}$), 57.83 (1C $CH_2\underline{C}H_2N^{piperazine}$), 53.18 ($2C^{piperazine}$), 52.63 ($2C^{piperazine}$), 40.01 (1C, $\underline{C}H_2NH$), 27.29 (1C, $CH_2\underline{C}H_2CH_2$), 24.33 (1C, $CH_2\underline{C}H_2CH_2$).

Preparation of 4-(4-(4-phenoxybenzyl)piperazin-1-yl)-*N*-(4-(trifluoromethyl)benzyl)butan-1-amine (**22**)

$LiAlH_4$ (0.07 g; $1.79\cdot10^{-3}$ mol) was added to a solution of *N*-(4-(4-(4-phenoxybenzyl)piperazin-1-yl)butyl)-4-(trifluoromethyl)benzamide (**21**) (0.23 g; $4.49\cdot10^{-4}$ mol) in 30 mL anhydrous diethyl ether. The reaction mixture was stirred overnight at room temperature, then the mixture was quenched by dropwise addition of water (16 eq.) and 10 % NaOH solution (16 eq.) stirred for two hours, then filtered. The precipitate was discarded. The organic

layer was dried over Na_2SO_4, then the solvent was removed under vacuum and the crude product was purified by column chromatography (EtOAc/MeOH/Triethylamine 49:10:1) to yield the pure product.

4-(4-(4-phenoxybenzyl)piperazin-1-yl)-*N*-(4-(trifluoromethyl)benzyl)butan-1-amine (**22**): $C_{29}H_{34}F_3N_3O$. M=497.61. Sticky oil. 76.20 % yield. R_F=0.26 (EtOAc/MeOH/Triethylamine 49:10:1). ^{1}H NMR (600 MHz, *CDCl3)* δ ppm 7.59-7.58 (m, 2H$^{arom.}$, $C(CHC\underline{H})_2CCF_3$), 7.51-7.49 (m, 2H$^{arom.}$, $C(C\underline{H}CH)_2CCF_3$), 7.33-7.31 (m, 2H$^{phenoxy.}$, $C(CHC\underline{H})_2CH$), 7.26-7.24 (m, 2H$^{benz.}$, $C(C\underline{H}CH)_2CO$), 7.09-7.07 (m, 1H$^{phenoxy.}$, $C(CHCH)_2C\underline{H}$), 6.98-6.97 (m, 2H$^{phenoxy.}$, $C(C\underline{H}CH)_2CH$), 6.95-6.94 (m, 2H$^{benz.}$, $C(CHC\underline{H})_2CO$), 3.87 (s, 2H, $CF_3PhC\underline{H}_2$), 3.46 (s, 2H, $PhC\underline{H}_2N^{piperazine}$), 2.66 (t, 2H, $NHC\underline{H}_2CH_2$, *J*=6.37 Hz), 2.50-2.38 (m, 11H: 8H^{piperazine}; $CH_2C\underline{H}_2N^{piperazine}$, N$\underline{H}$*), 1.59-1.57 (m, 4H, $CH_2C\underline{H}_2CH_2$). ^{13}C NMR (150.95 MHz, *CDCl3*) δ ppm 157.35 (1C$^{quat./benz.}$, $\underline{C}O$), 156.27 (1C$^{quat./phenoxy.}$, $\underline{C}O$), 132.87 (1C$^{quat./benz.}$, $\underline{C}(CHCH)_2CO$), 130.49 (2C$^{benz.}$, $C(\underline{C}HCH)_2CO$), 129.71, 129.49, 129.28, 129.06 (1C$^{quat./arom.}$, $\underline{C}CF_3$), 129.68 (2C$^{phenoxy.}$, $C(CH\underline{C}H)_2CH$), 128.59 (1C$^{quat./arom.}$, $\underline{C}CH_2$), 128.42 (2C$^{arom.}$, $C(\underline{C}HCH)_2CCF_3$), 126.92, 125.12, 123.32, 121.51 (1C, $\underline{C}F_3$), 125.35, 125.32 (2C$^{arom.}$, $C(CH\underline{C}H)_2CCF_3$), 123.11 (1C$^{phenoxy.}$, $C(CHCH)_2\underline{C}H$), 118.75 (2 C^{benz}, $C(\underline{C}HCH)_2CH$), 118.63 (2C^{phenoxy}, $C(\underline{C}HCH)_2CH$), 62.32 (1C, $Ph\underline{C}H_2N^{piperazine}$), 58.37 (1C, $CH_2\underline{C}H_2N^{piperazine}$), 53.16 (1C, $CH_2CH_2N(H)\underline{C}H_2$), 52.82 (4C^{piperazine}), 49.13 (1C, $CH_2\underline{C}H_2N(H)CH_2$), 27.87 (1C, $CH_2\underline{C}H_2CH_2$), 24.61 (1C, $CH_2\underline{C}H_2CH_2$).

Preparation of 2,3-di(*tert*-butoxycarbonyl)-1-(4-(4-(4-phenoxybenzyl)piperazin-1-yl)butyl)-1-(4-(trifluoromethyl)benzyl)guanidine (**23**)

1,3-bis(*tert*-butoxycarbonyl)-2-methylisothiourea (0.30 g; $1.04\cdot10^{-3}$ mol) and mercury II chloride (0.28 g; $1.04\cdot10^{-3}$ mol) were sequentially added to an ice-cooled mixture of 4-(4-(4-phenoxybenzyl)piperazin-1-yl)-*N*-(4-(trifluoromethyl)benzyl)butan-1-amine (**22**) (0.47 g; $9.44\cdot10^{-4}$ mol) and triethylamine (0.48 g; $4.72\cdot10^{-3}$ mol) in 50 mL DCM. The ice bath was removed and the reaction was stirred for eighteen hours at room temperature, then filtered. The precipitate was discarded. The filtrate was washed sequentially twice with 20 mL H_2O and twice with 20 mL brine. The combined organic phases were dried over Na_2SO_4, then the solvent was removed under vacuum and the crude product was purified by column chromatography (EtOAc/MeOH/Triethylamine 189:10:1) to yield the pure product.

2,3-di(*tert*-butoxycarbonyl)-1-(4-(4-(4-phenoxybenzyl)piperazin-1-yl)butyl)-1-(4-(trifluoromethyl)benzyl)guanidine (**23**): $C_{40}H_{52}F_3N_5O_5$. M=739.87. White solid. 98.57 % yield. R_F=0.50 (EtOAc/MeOH/Triethylamine 189:10:1). mp: 105.1-107.0 °C. ^{1}H NMR (600 MHz, *CDCl3)* δ ppm 9.95 (br, 1H, N$\underline{H}$), 7.60-7.58 (m, 2H$^{arom.}$, $C(CHC\underline{H})_2CCF_3$), 7.42-7.41 (m, 2H$^{arom.}$, $C(C\underline{H}CH)_2CCF_3$), 7.34-7.28 (m, 2H$^{phenoxy.}$, $C(CHC\underline{H})_2CH$), 7.28-7.23 (m, 2H$^{benz.}$, $C(C\underline{H}CH)_2CO$), 7.11-7.06 (m, 1H$^{phenoxy.}$, $C(CHCH)_2C\underline{H}$), 7.02-6.99 (m, 2H$^{phenoxy.}$, $C(C\underline{H}CH)_2CH$), 6.96-6.94 (m, 2H$^{benz.}$, $C(CHC\underline{H})_2CO$), 4.76 (br, 2H, $CF_3PhC\underline{H}_2$), 3.46 (s, 2H, $PhC\underline{H}_2N^{piperazine}$), 3.32 (m, 2H, $CH_2C\underline{H}_2C(N)N$), 2.44-2.42 (br, 8H^{piperazine}), 2.28 (t, 2H, $CH_2C\underline{H}_2N^{piperazine}$), 1.58-1.54 (m, 2H, $CH_2C\underline{H}_2CH_2$), 1.49 (s, 18H, $C\underline{H}_3$), 1.40 (qt, 2H, $CH_2C\underline{H}_2CH_2$). ^{13}C NMR (150.95 MHz, *CDCl3*) δ ppm 162.60 (1C, $\underline{C}$=O), 157.41 (1C$^{quat./benz.}$, $\underline{C}O$), 156.23 (1C$^{quat./phenoxy.}$, $\underline{C}O$), 156.15 (1C$^{quat.}$, $\underline{C}$=N), 150.93 (1C, $\underline{C}$=O), 140.76 (1C$^{quat./arom.}$, $\underline{C}CH_2NC(N)$)), 133.03 (1C$^{quat./benz.}$, $\underline{C}(CHCH)_2CO$), 130.48 (2C$^{benz.}$, $C(\underline{C}HCH)_2CO$), 130.09, 129.87, 129.66, 129.45 (1C$^{arom.}$, $\underline{C}CF_3$), 129.67 (2C$^{phenoxy.}$, $C(CH\underline{C}H)_2CH$), 128.01 (2C$^{arom.}$, $C(\underline{C}HCH)_2CCF_3$), 126.82, 125.01, 123.21, 121.41 (1C, $\underline{C}F_3$), 125.58, 125.56 (2C$^{arom.}$, $C(CH\underline{C}H)_2CCF_3$), 123.21 (1C$^{phenoxy.}$, $C(CHCH)_2\underline{C}H$), 118.73 (2C$^{benz.}$, $C(CH\underline{C}H)_2CO$), 118.64 (2C^{phenoxy}, $C(\underline{C}HCH)_2CH$), 82.09 (1C$^{quat.}$ Boc), 79.59 (1C$^{quat.}$ Boc), 62.39 (1C, $Ph\underline{C}H_2N^{piperazine}$), 57.89 (1C, $CH_2\underline{C}H_2N^{piperazine}$), 53.17, 53.03 (4C^{piperazine}), 50.98 (1C, $CF_3Ph\underline{C}H_2$), 48.13 (1C, $CH_2\underline{C}H_2NC(N)$)), 28.18, 28.17 (6C, $\underline{C}H_3$), 25.25 (1C, $CH_2\underline{C}H_2CH_2$), 23.89 (1C, $CH_2\underline{C}H_2CH_2$).

Preparation of 1-(4-(4-(4-phenoxybenzyl)piperazin-1-yl)butyl)-1-(4-(trifluoromethyl)benzyl)guanidine (**ADS-10210**)

4M solution HCl-dioxan (2.03 mL; $8.11\cdot10^{-3}$ mol) was added dropwise to a solution of the 2,3-di(*tert*-butoxycarbonyl)-1-(4-(4-(4-phenoxybenzyl)piperazin-1-yl)butyl)-1-(4-(trifluoromethyl)benzyl)guanidine (**23**) (0.30 g; $4.05\cdot10^{-4}$ mol) in 30 mL chloroform. The reaction was stirred overnight at room temperature, then the solvent was removed under vacuum. The crude product was evaporated twice from chloroform and twice from EtOAc, then dried to yield the pure product.

1-(4-(4-(4-phenoxybenzyl)piperazin-1-yl)butyl)-1-(4-(trifluoromethyl)benzyl)guanidine (**ADS-10210**): $C_{30}H_{36}F_3N_5O\cdot 3HCl$. M=649.01. White solid. 98.86 %. mp: 218.5-220.0 °C with decomposition. ^{1}H NMR (600 MHz, *CD₃OD*) δ ppm 7.69-7.68 (m, $2H^{arom.}$, $C(CHC\underline{H})_2CCF_3$), 7.58-7.56 (m, $2H^{benz.}$, $C(C\underline{H}CH)_2CO$), 7.45-7.43 (m, $2H^{arom.}$, $C\underline{H}CHCCF_3$), 7.38-7.35 (m, $2H^{phenoxy.}$, $C(CHC\underline{H})_2CH$), 7.17-7.14 (m, $1H^{phenoxy.}$, $C(CHCH)_2C\underline{H}$), 7.03-7.00 (m, 4H: $2H^{phenoxy.}$, $C(C\underline{H}CH)_2CH$; $2H^{benz.}$, $C(CHC\underline{H})_2CO$), 4.77 (br, 2H, $CF_3PhC\underline{H}_2$), 4.45 (m, 2H, (s, 2H, $PhC\underline{H}_2N^{piperazine}$), 3.84-3.57 (br, $8H^{piperazine}$), 3.42 (t, 2H, $CH_2C\underline{H}_2N^{piperazine}$), 3.28 (m, 2H, $CH_2C\underline{H}_2NC(N)$), 1.81-1.75 (m, 4H, $CH_2C\underline{H}_2CH_2$). ^{13}C NMR (150.95 MHz, *CD₃OD*) δ ppm 159.58 (1C, $\underline{C}$=N), 157.05 ($1C^{quat./benz.}$, $\underline{C}O$), 156.13 ($1C^{quat./phenoxy.}$, $\underline{C}O$), 139.68 ($1C^{quat./arom.}$, $\underline{C}CH_2NC(N)$), 133.11 ($2C^{benz.}$, $C(\underline{C}HCH)_2CO$), 129.86, 129.64, 129.43, 129.21 ($1C^{arom.}$, $\underline{C}CF_3$), 129.76 (3C: $2C^{phenoxy.}$, $C(CH\underline{C}H)_2CH$; $1C^{quat./benz.}$, $\underline{C}(CHCH)_2CO$), 127.01 ($2C^{arom.}$, $C(\underline{C}HCH)_2CCF_3$), 126.84, 125.06, 123.26, 121.50 (1C, $\underline{C}F_3$), 125.54, 125.51 ($2C^{arom.}$, $C(CH\underline{C}H)_2CCF_3$), 124.01 ($1C^{phenoxy.}$, $C(CHCH)_2\underline{C}H$), 119.36 ($2C^{benz.}$, $C(CH\underline{C}H)_2CO$), 118.32 ($2C^{phenoxy.}$, $C(\underline{C}HCH)_2CH$), 59.41 (1C, $CH_2\underline{C}H_2N^{piperazine}$), 56.09 (1C, $CH_2\underline{C}H_2NC(N)$), 48.49 ($4C^{piperazine}$), 50.40 (1C, $CF_3Ph\underline{C}H_2$), 48.28 (1C, $CH_2\underline{C}H_2N^{piperazine}$), 23.87 (1C, $CH_2\underline{C}H_2CH_2$), 20.56 (1C, $CH_2\underline{C}H_2CH_2$). Anal. Calcd: C 55.52%; H 6.06%; N 10.79%. Found: C, 55.16%; H 6.35%; N 10.89%.

2. NMR spectra

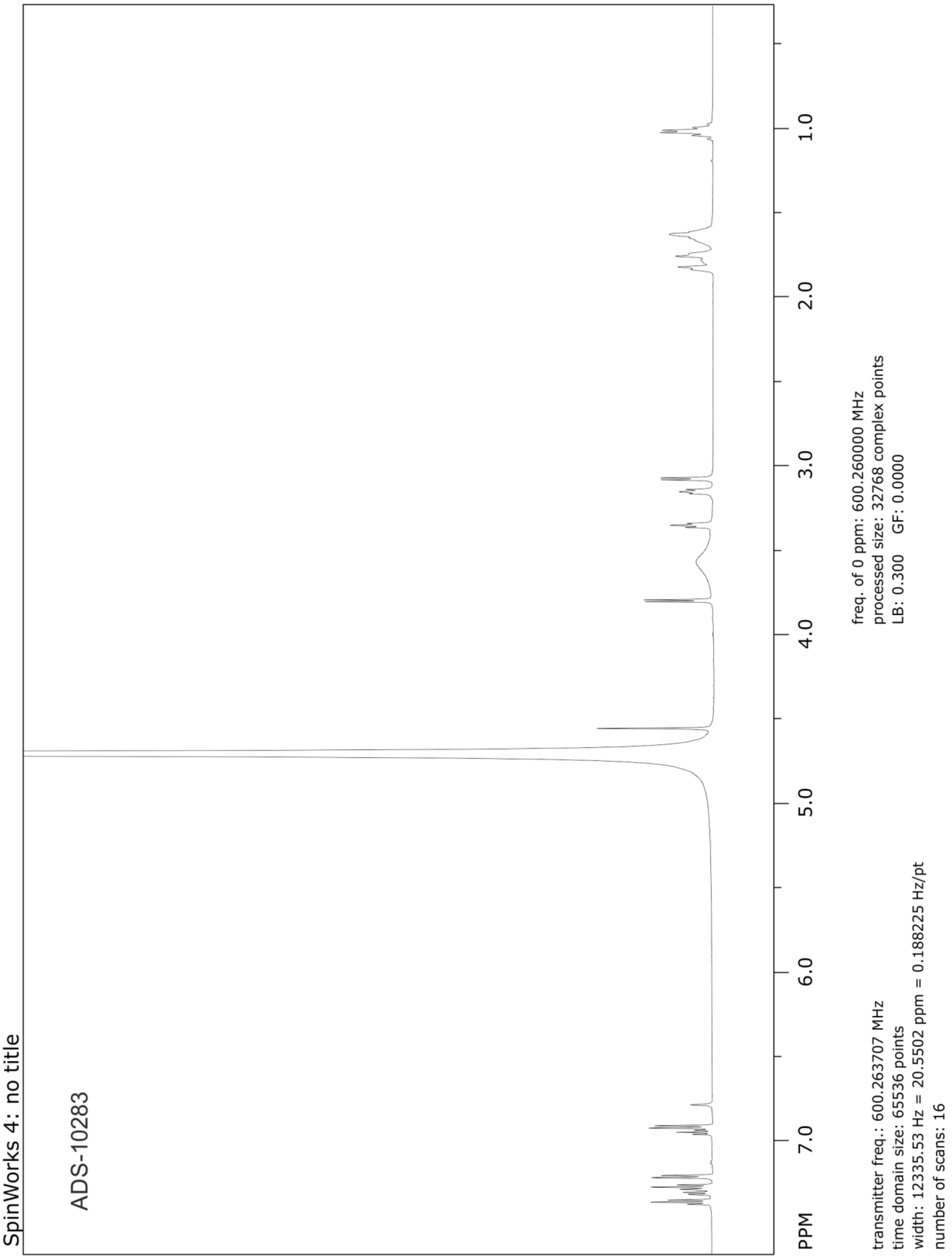

Figure S1.^{1}H NMR spectra of compound ADS10283.

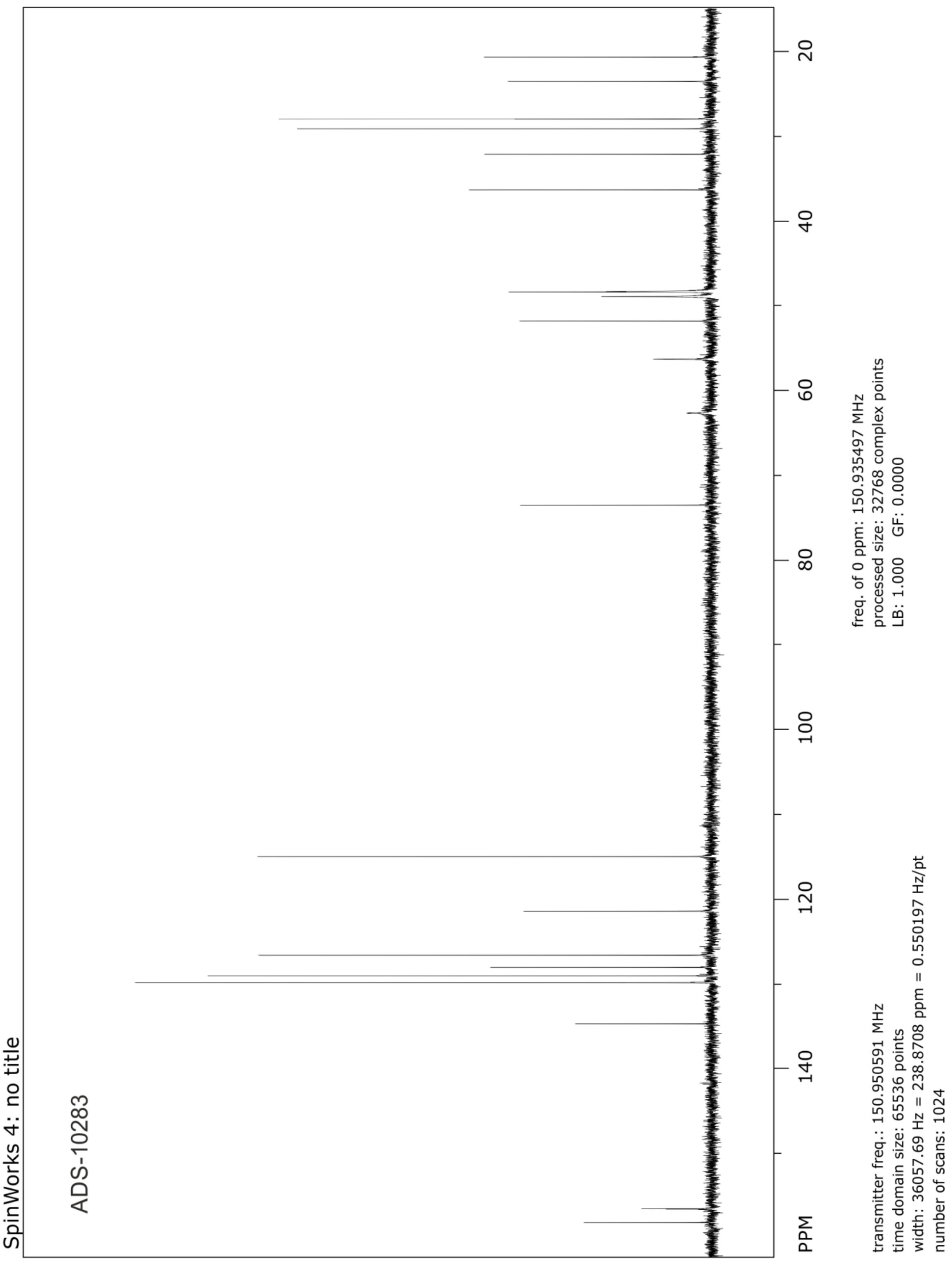

Figure S2.^{13}C NMR spectra of compound ADS10283

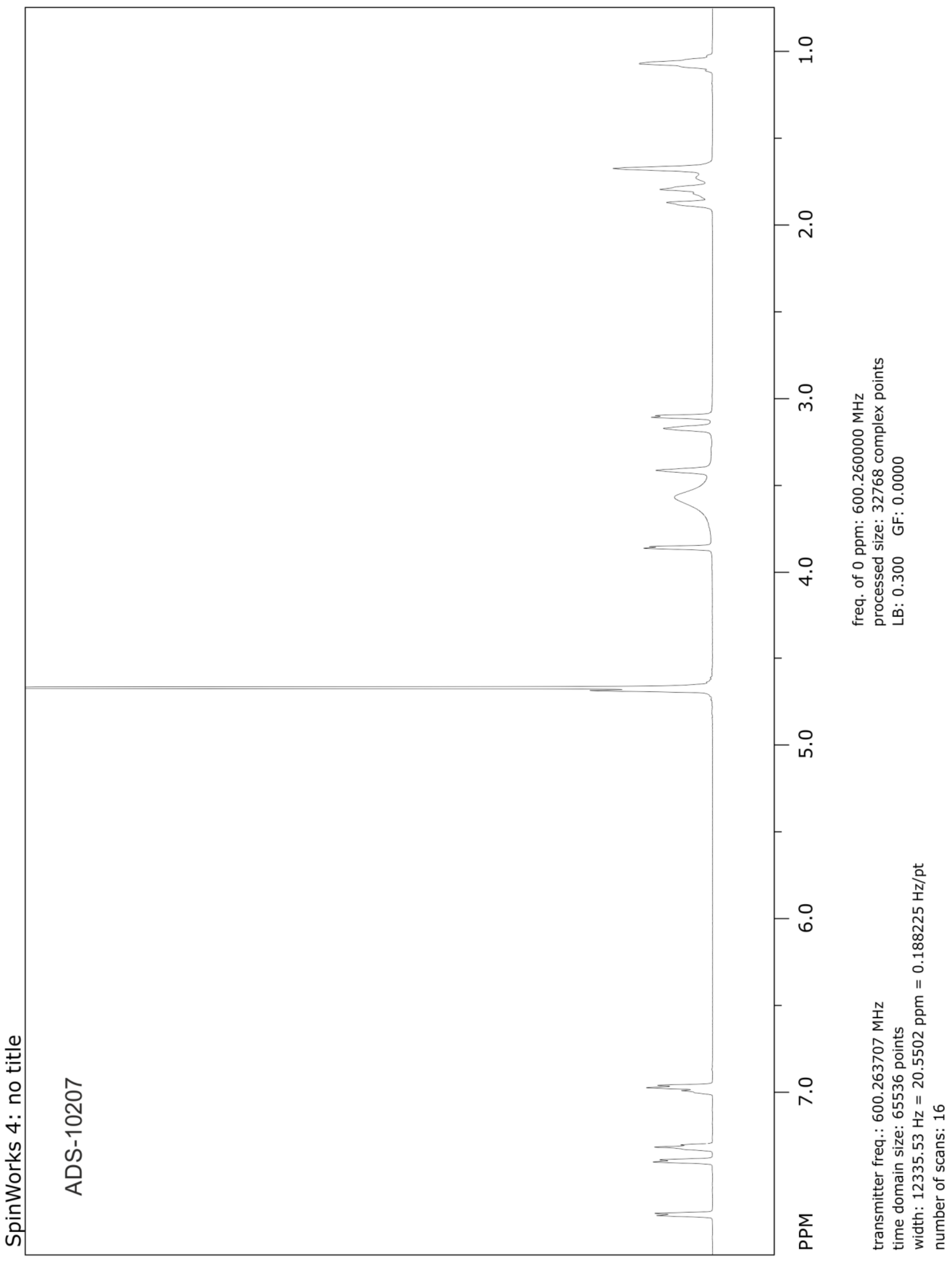

Figure S3.^{1}H NMR spectra of compound ADS10207

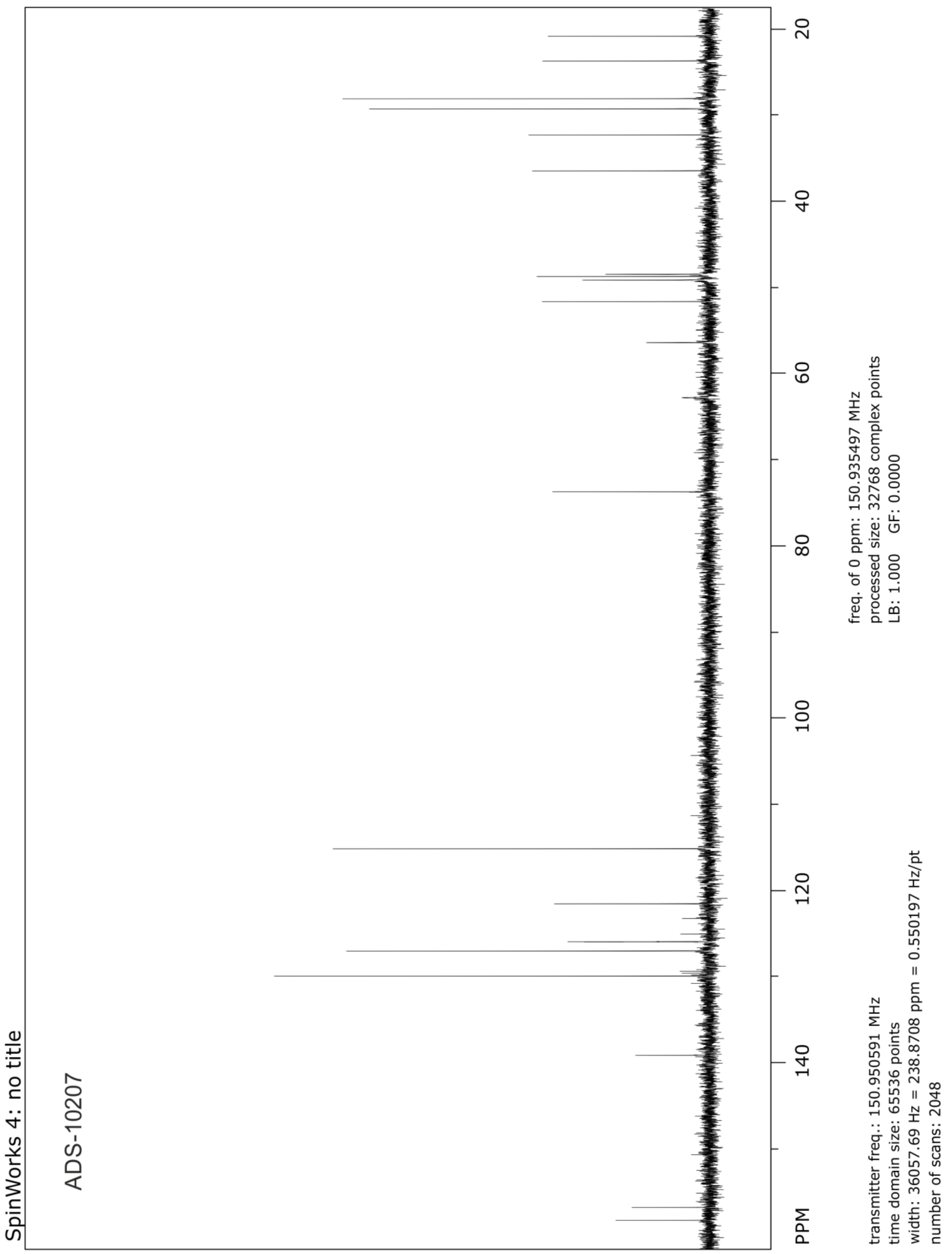

Figure S4.^{13}C NMR spectra of compound ADS10207

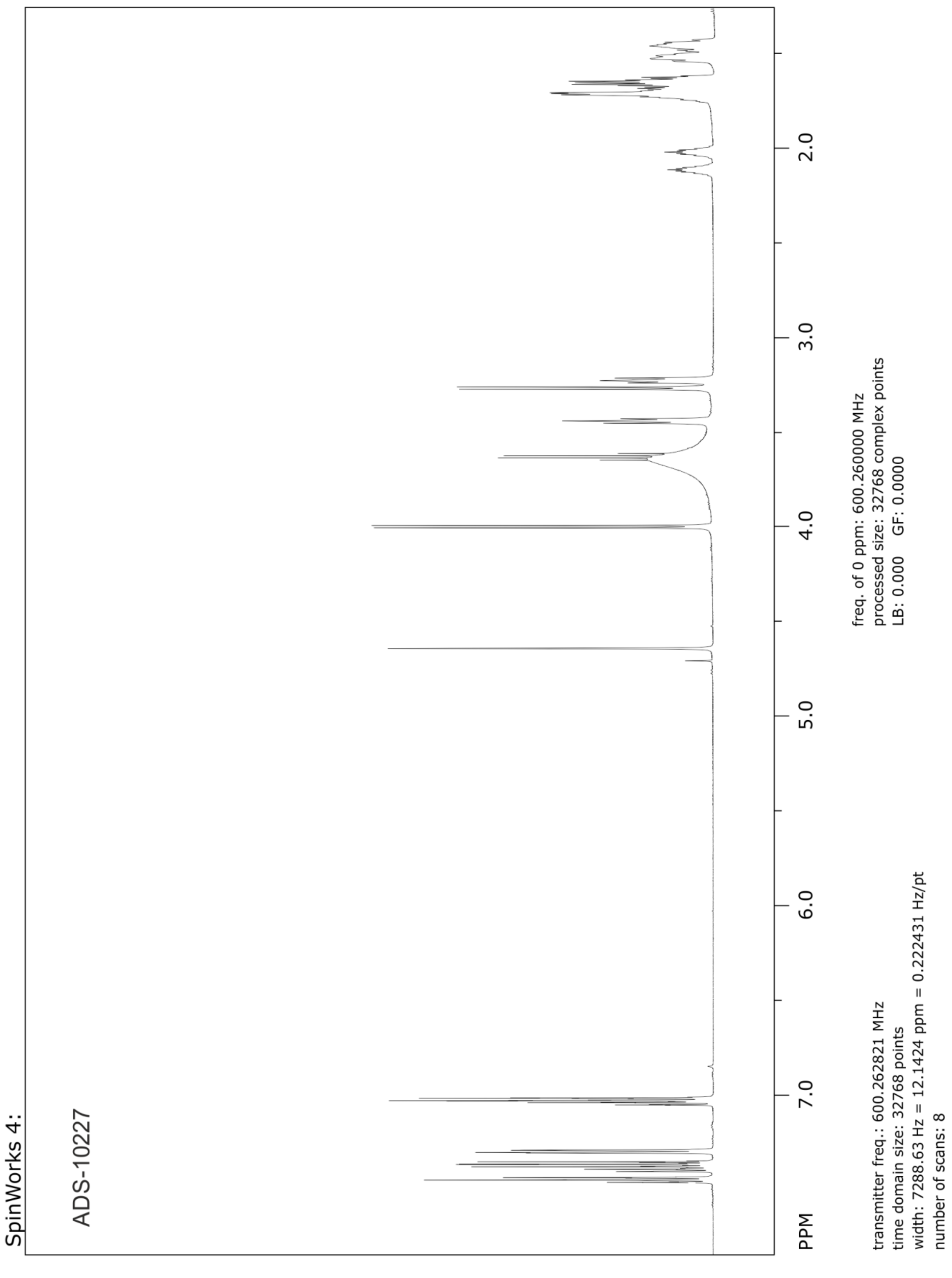

Figure S5.^{1}H NMR spectra of compound ADS10227

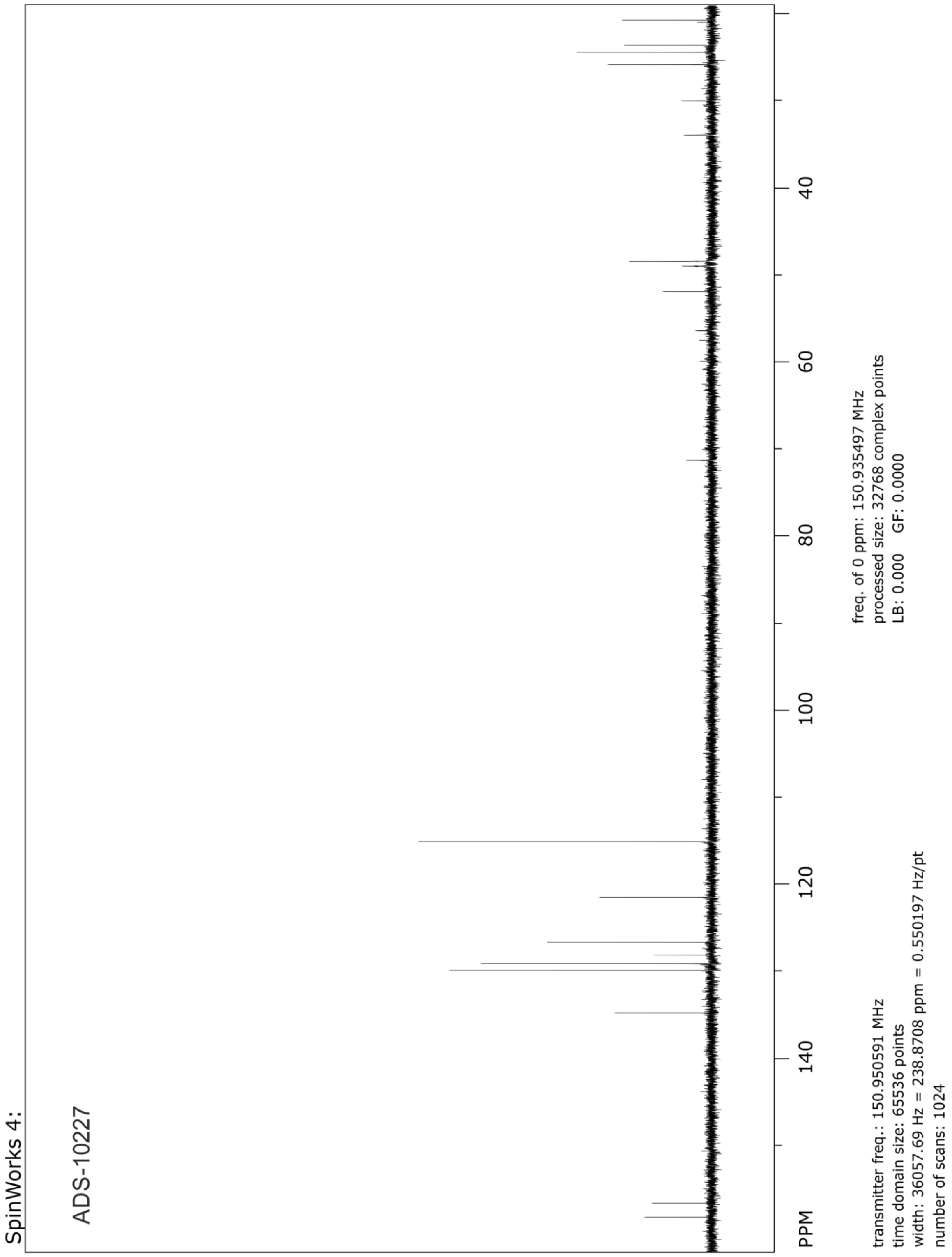

Figure S6.^{13}C NMR spectra of compound ADS10227

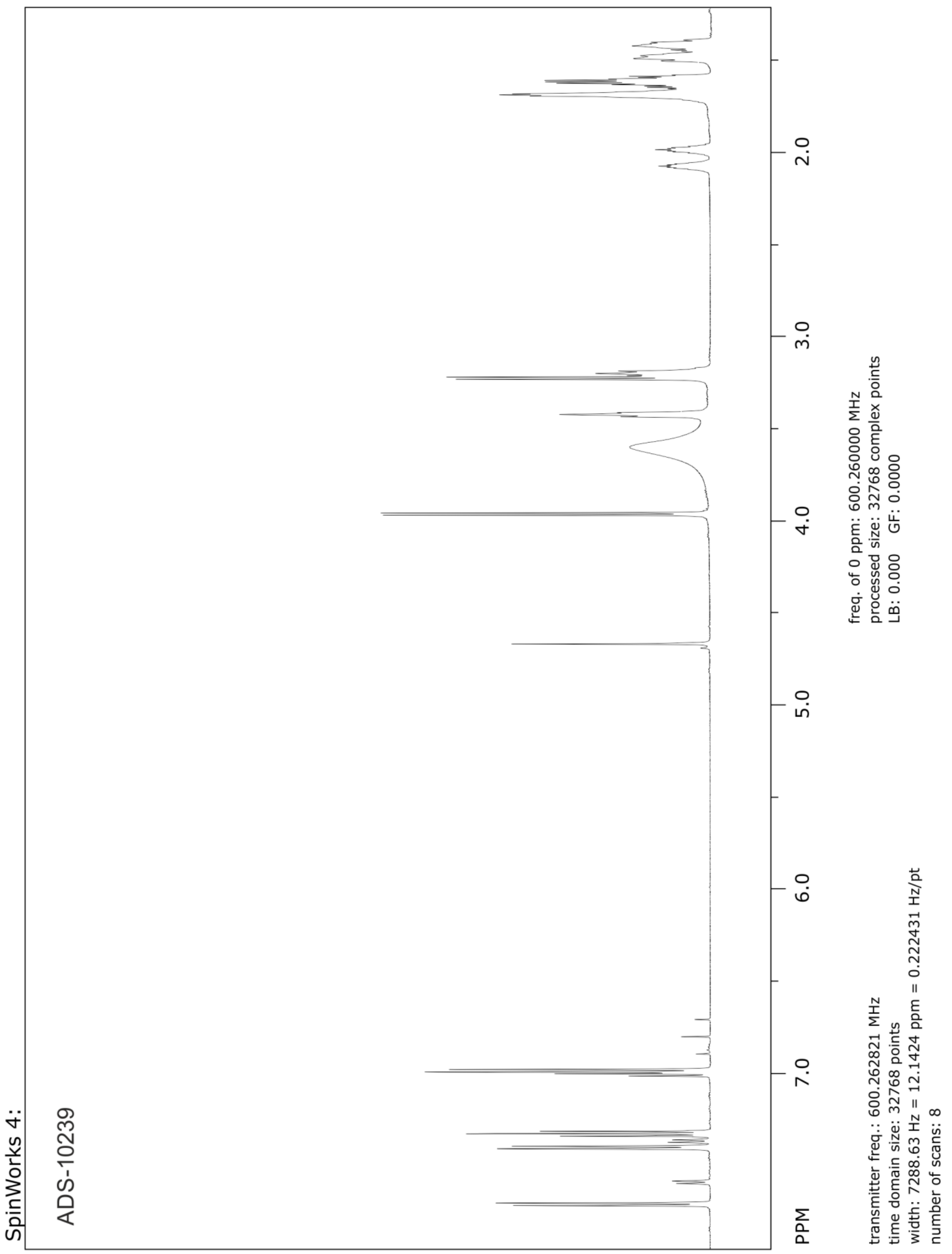

Figure S7.^{1}**H NMR spectra of compound ADS10239**

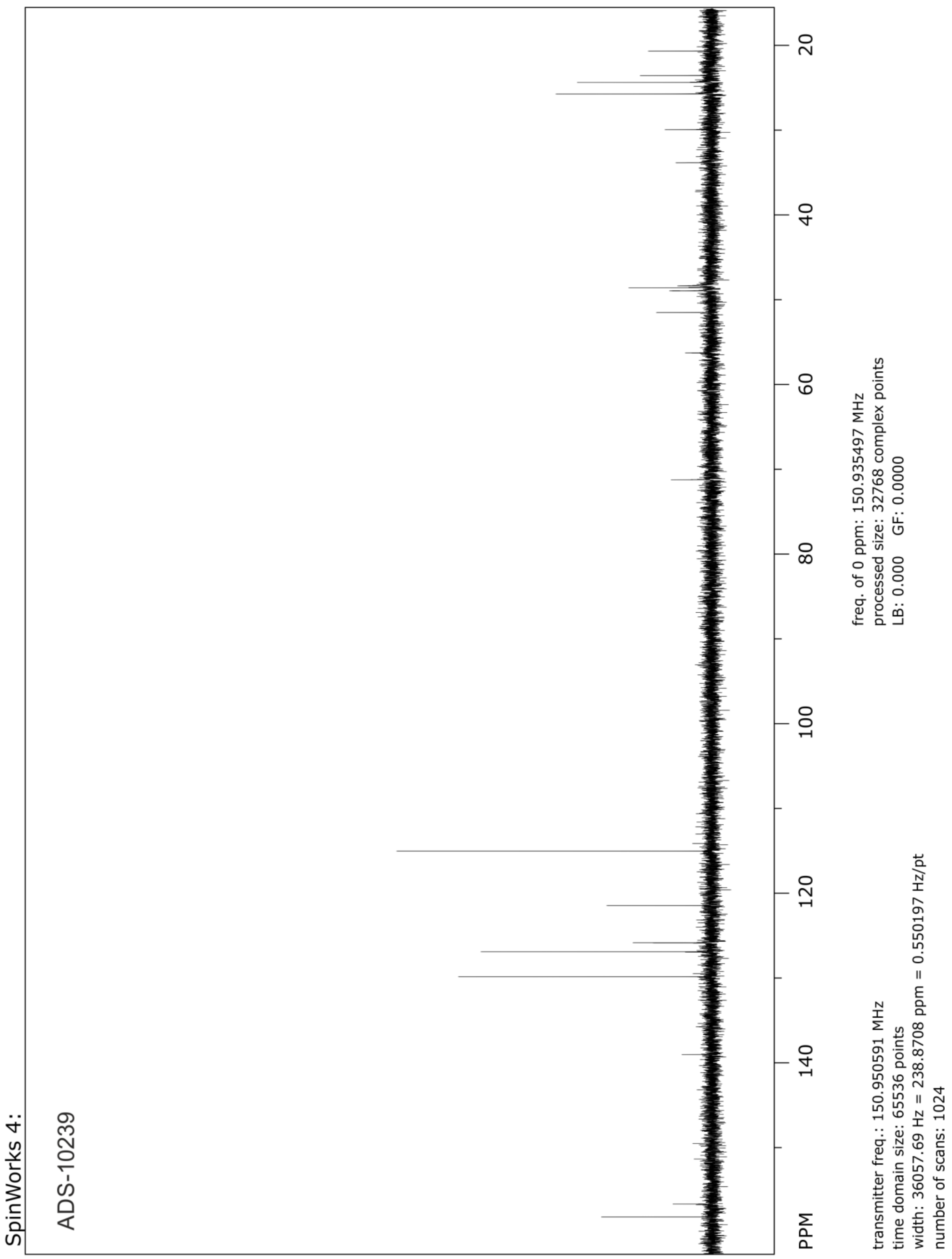

Figure S8.^{13}C NMR spectra of compound ADS10239

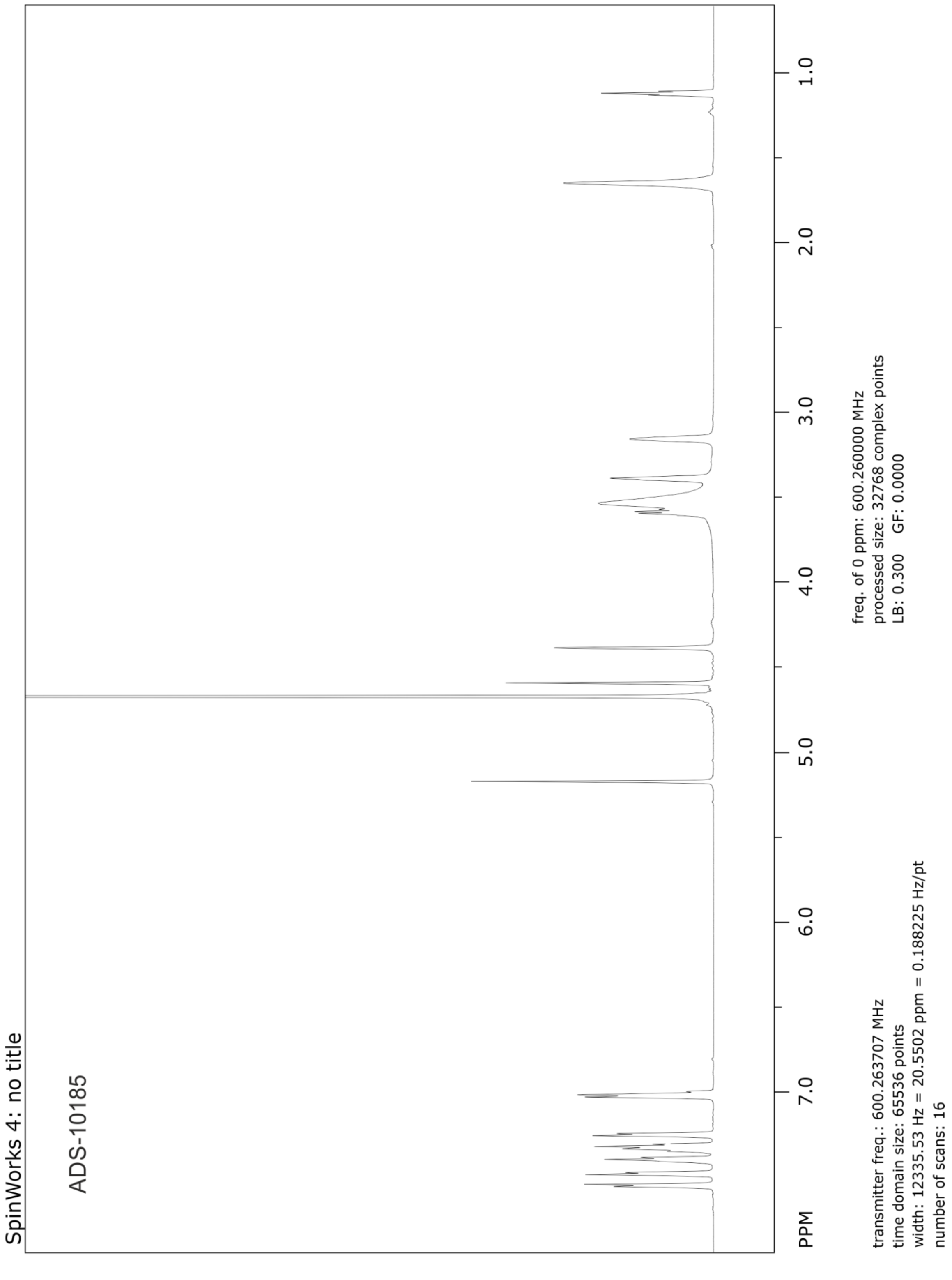

Figure S9.^{1}H NMR spectra of compound ADS10185

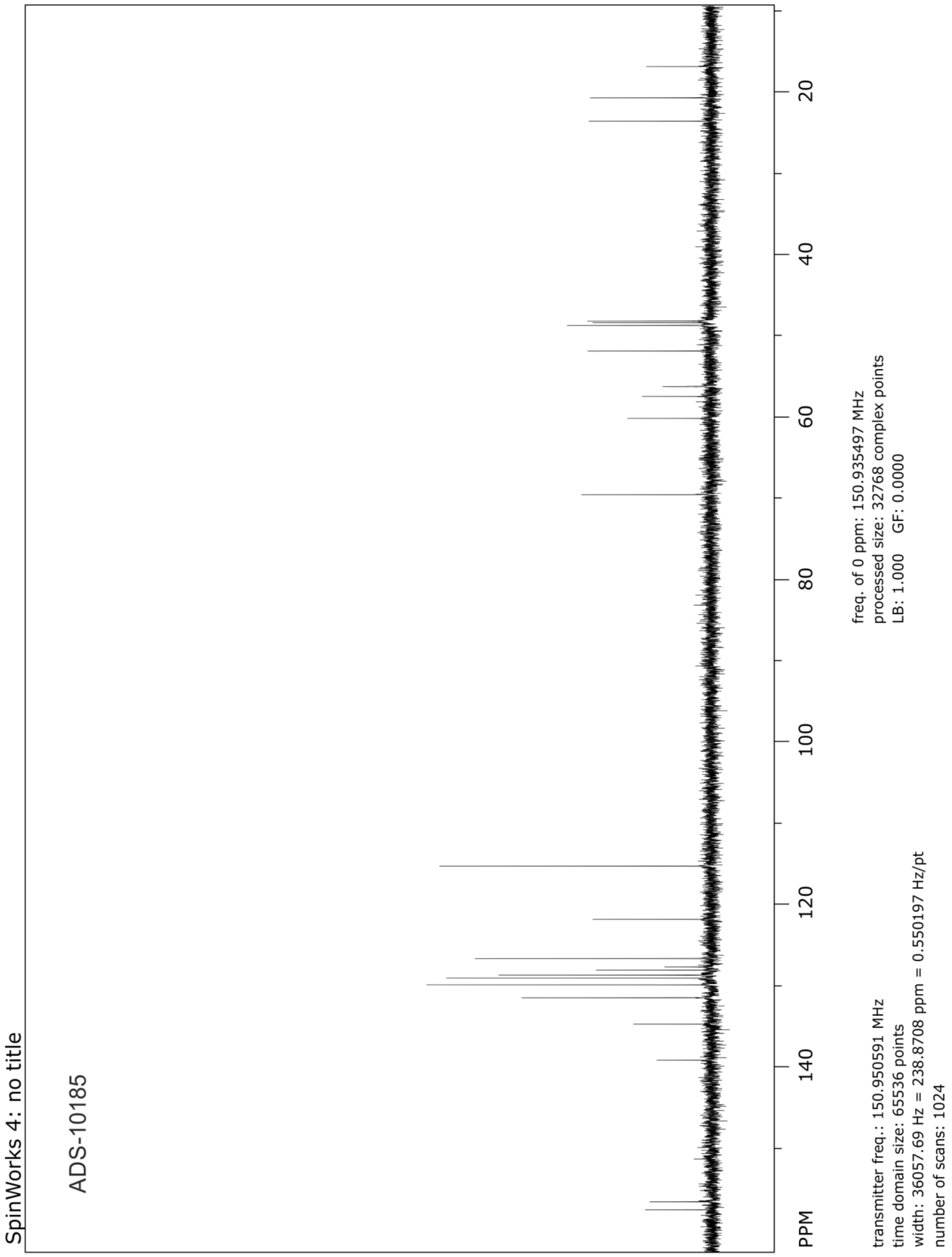

Figure S10.^{13}C NMR spectra of compound ADS10185

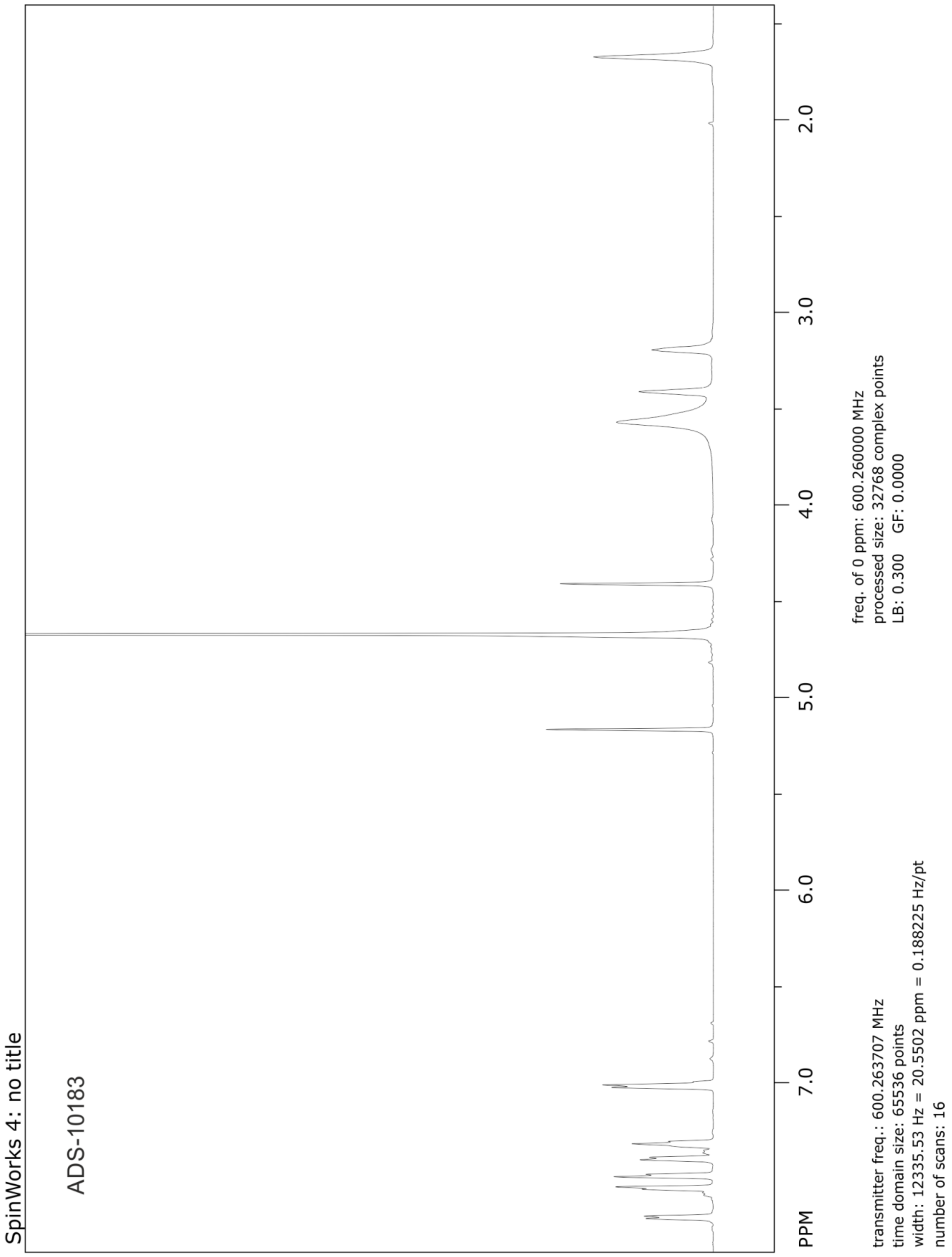

Figure S11.^{1}H NMR spectra of compound ADS10183

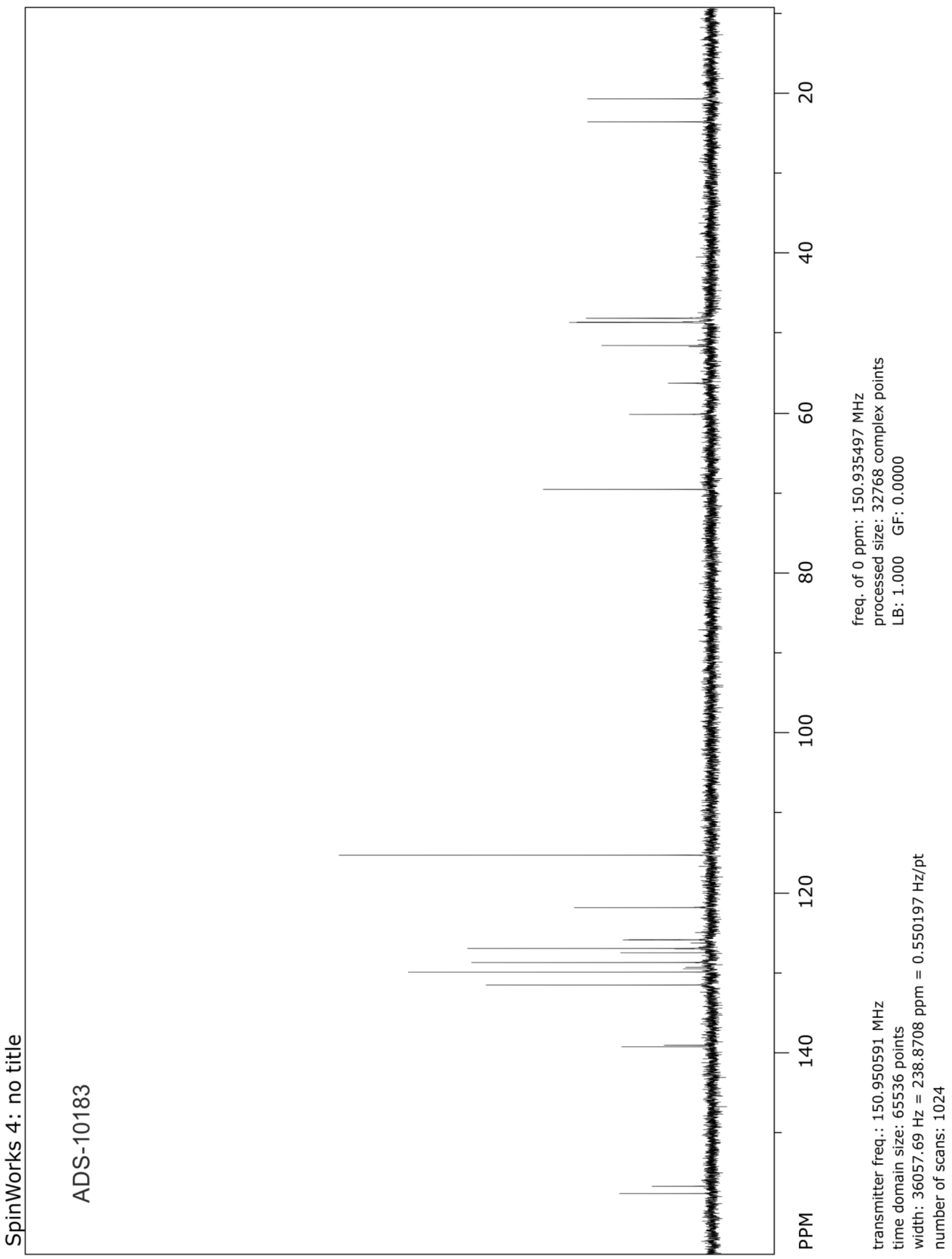

Figure S12. ^{13}**C NMR spectra of compound ADS10183**

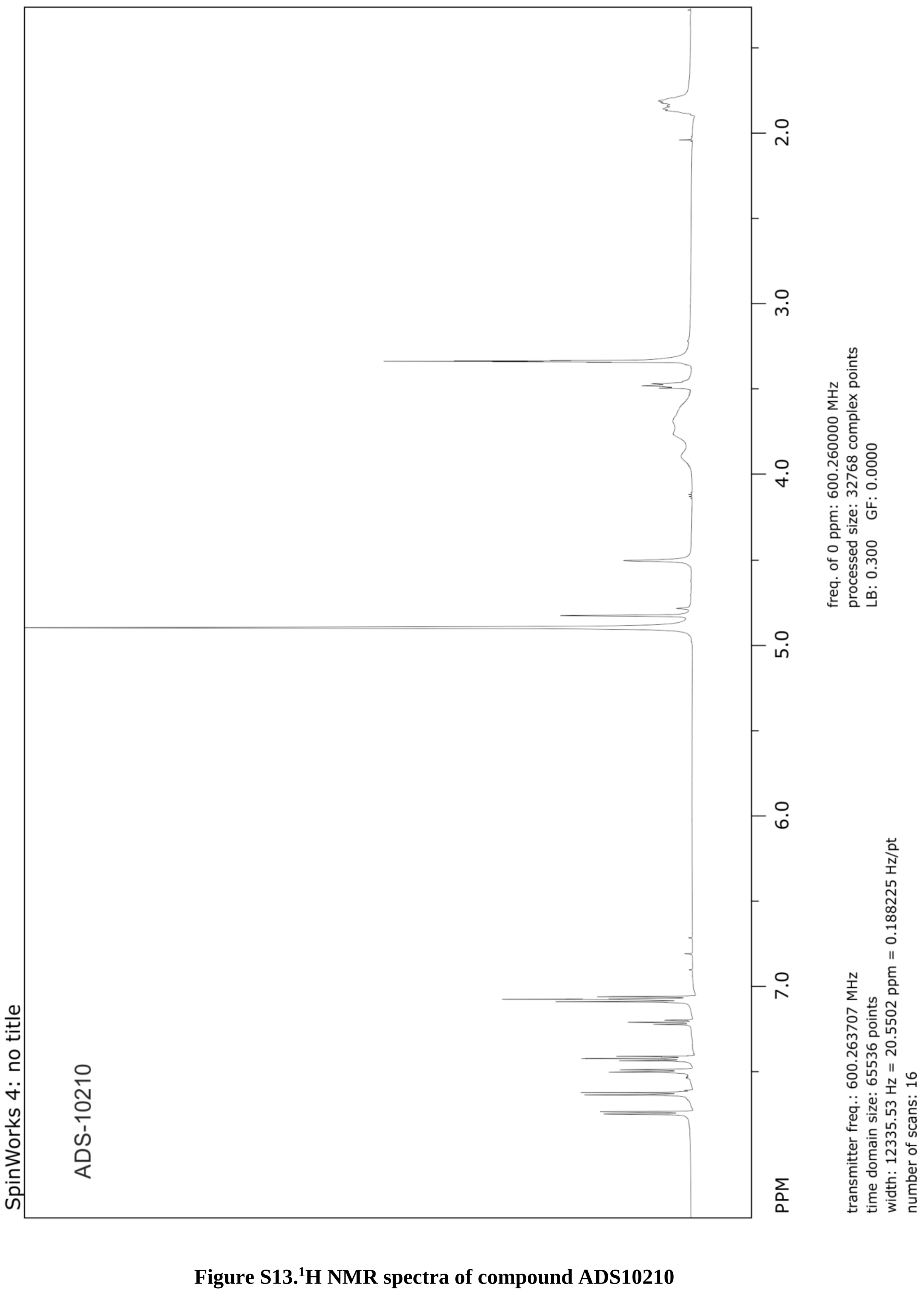

Figure S13.[1]**H NMR spectra of compound ADS10210**

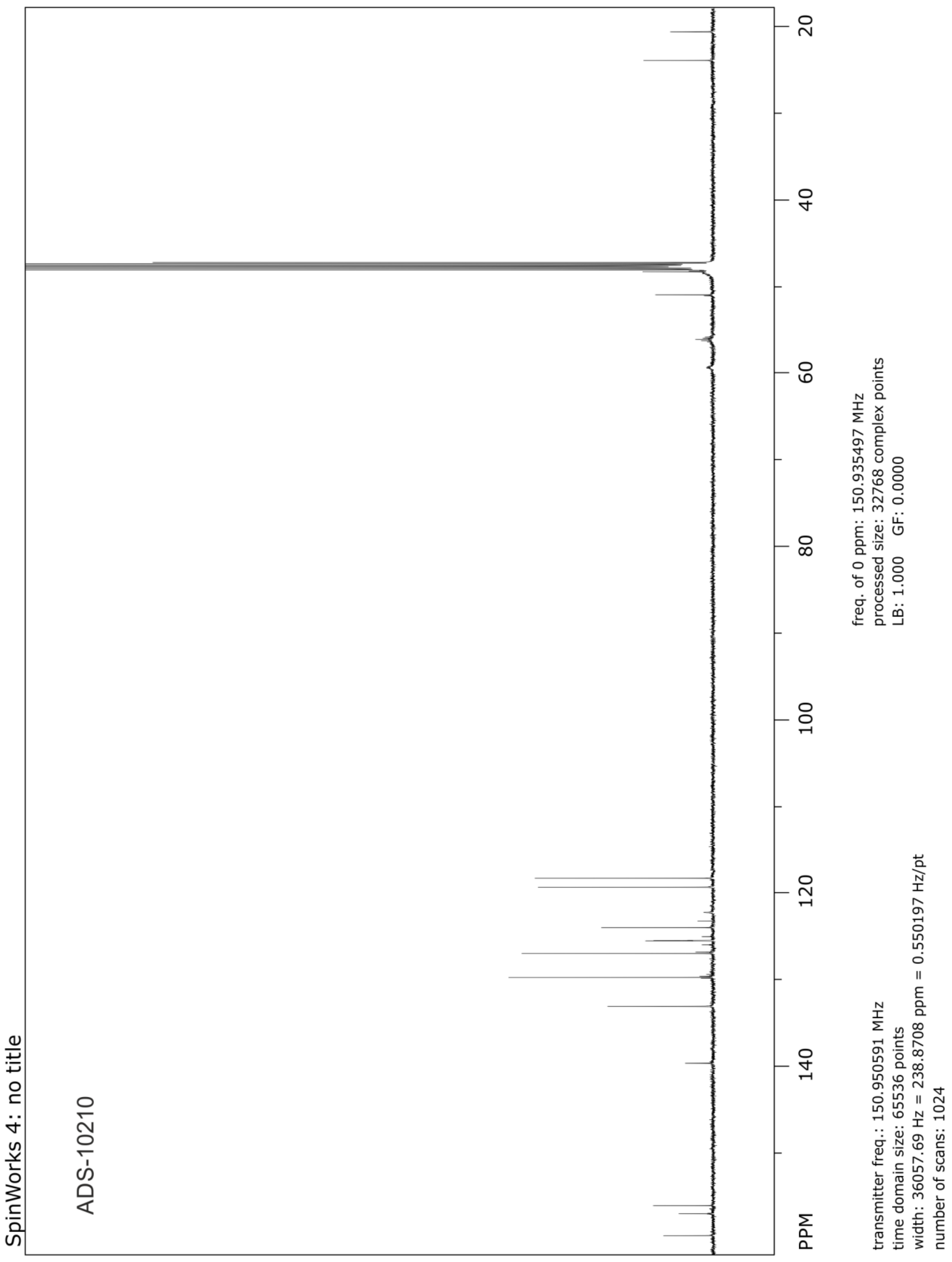

Figure S14.^{13}C NMR spectra of compound ADS10210

3. Pharmacological assay results.

3.1 *Ex vivo* assay for histamine H_1R receptor antagonists (variant with 0.05 μM atropine addition).

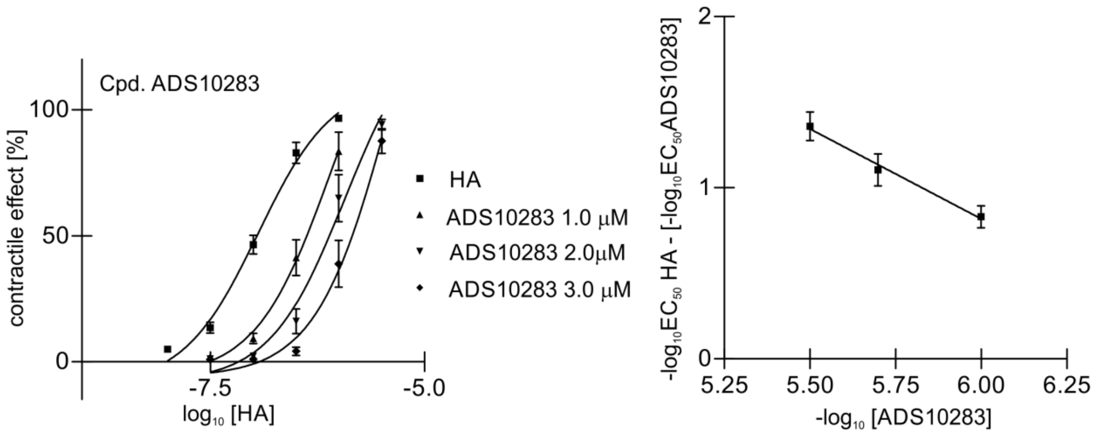

Figure S15. Contraction of guinea-pig ileum by histamine in the absence (■) and presence (▲,▼, ♦) of compound ADS10283

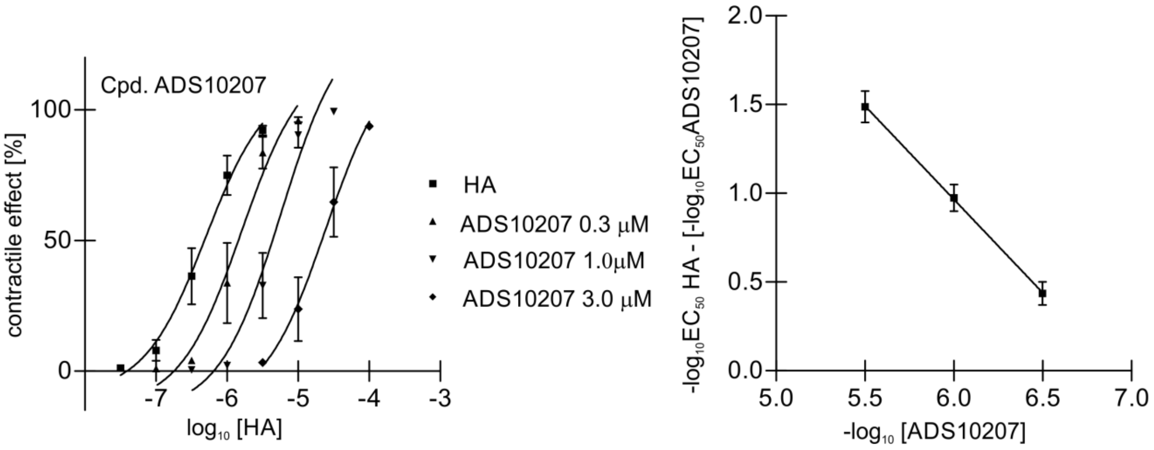

Figure S16. Contraction of guinea-pig ileum by histamine in the absence (■) and presence (▲,▼, ♦) of compound ADS10207

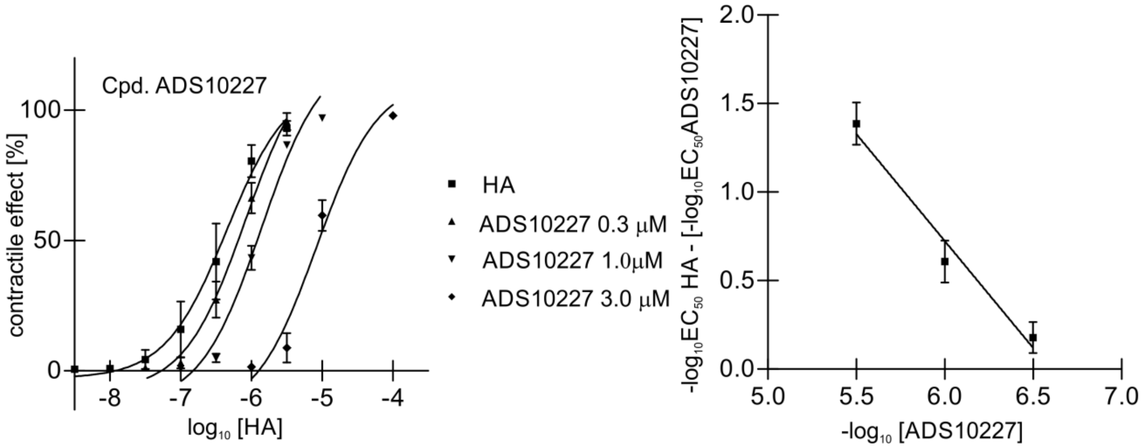

Figure S17. Contraction of guinea-pig ileum by histamine in the absence (■) and presence (▲,▼, ♦) of compound ADS10227

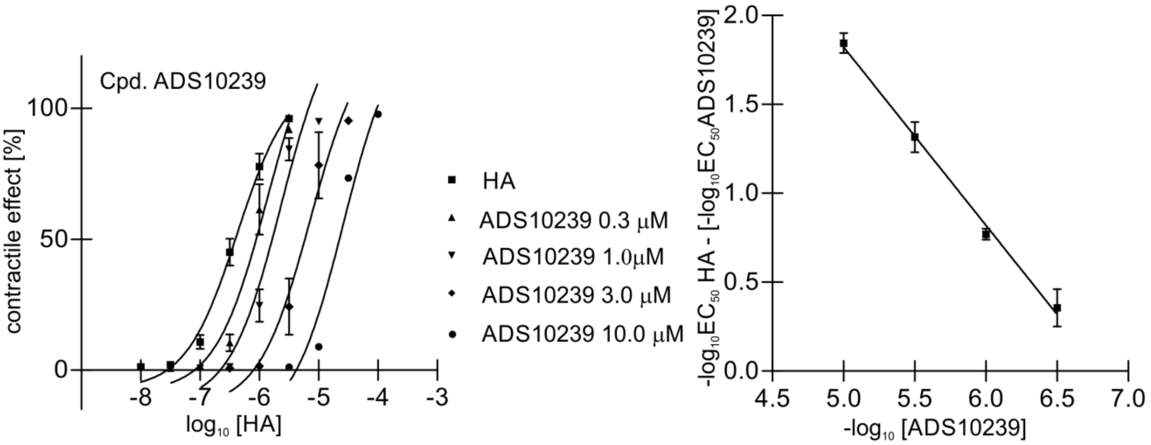

Figure S18. Contraction of guinea-pig ileum by histamine in the absence (■) and presence (▲,▼, ♦, ●) of compound ADS10239

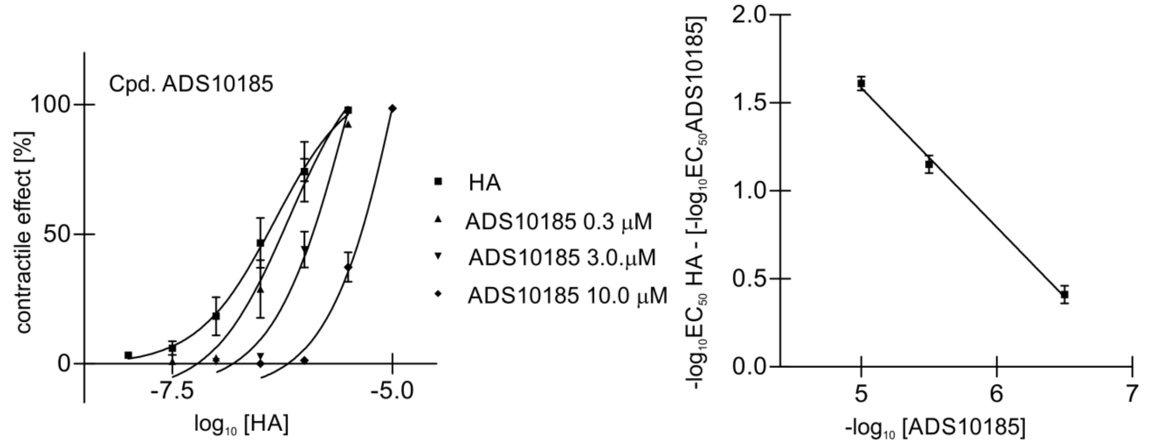

Figure S19. Contraction of guinea-pig ileum by histamine in the absence (■) and presence (▲,▼, ♦) of compound ADS10185

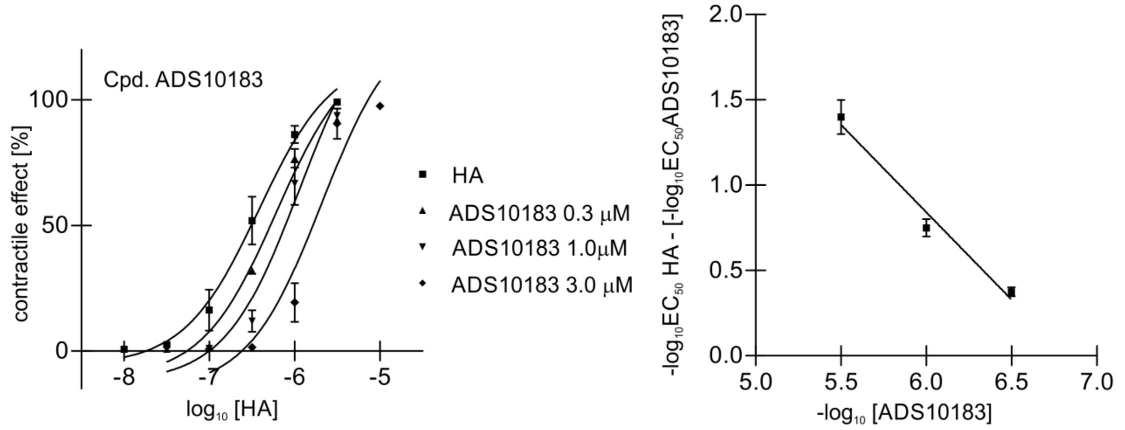

Figure S20. Contraction of guinea-pig ileum by histamine in the absence (■) and presence (▲,▼, ♦) of compound ADS10183

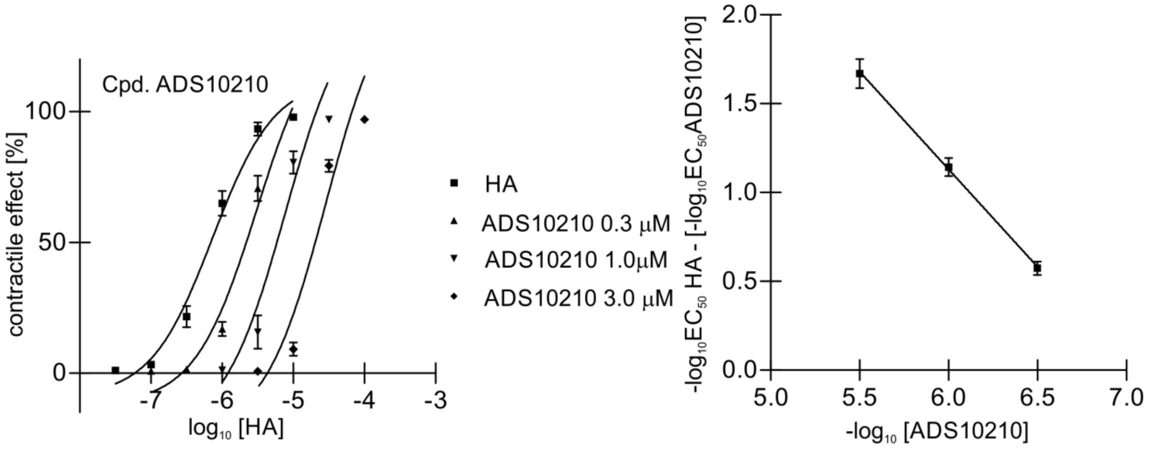

Figure S21. Contraction of guinea-pig ileum by histamine in the absence (■) and presence (▲,▼, ♦) of compound ADS10210

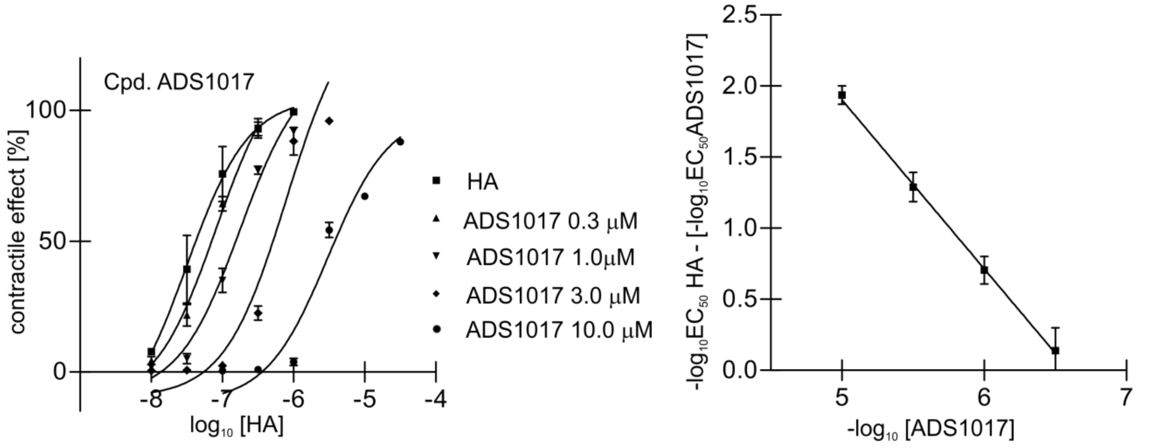

Figure S22. Contraction of guinea-pig ileum by histamine in the absence (■) and presence (▲,▼, ♦, ●) of compound ADS1017

3.2 *Ex vivo* assay for histamine H_1R receptor antagonists (variant without atropine addition).

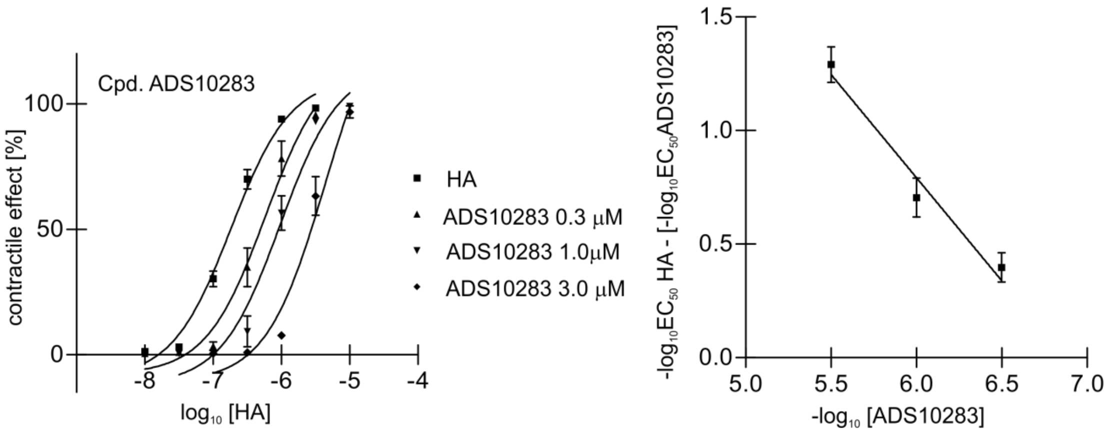

Figure S23. Contraction of guinea-pig ileum by histamine in the absence (■) and presence (▲,▼, ♦) of compound ADS10283

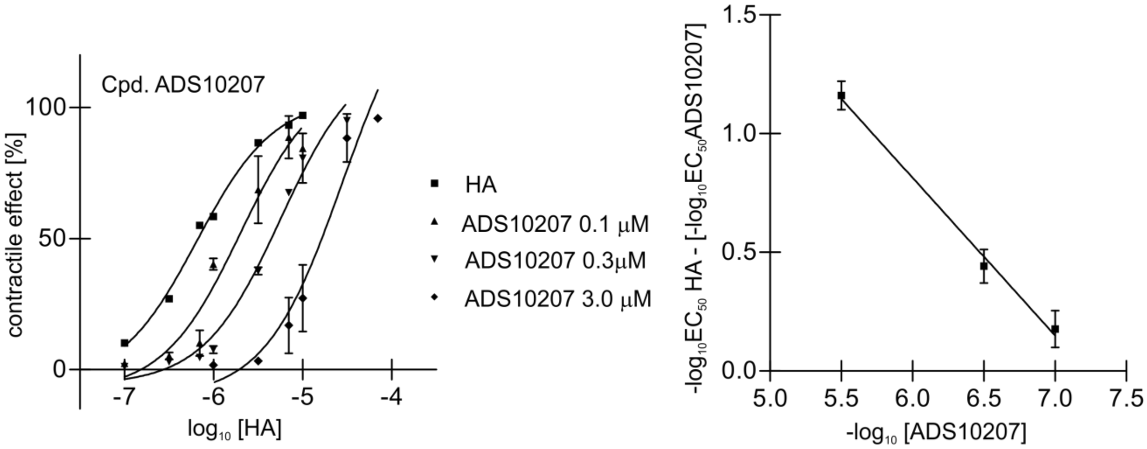

Figure S24. Contraction of guinea-pig ileum by histamine in the absence (■) and presence (▲,▼, ♦) of compound ADS10207

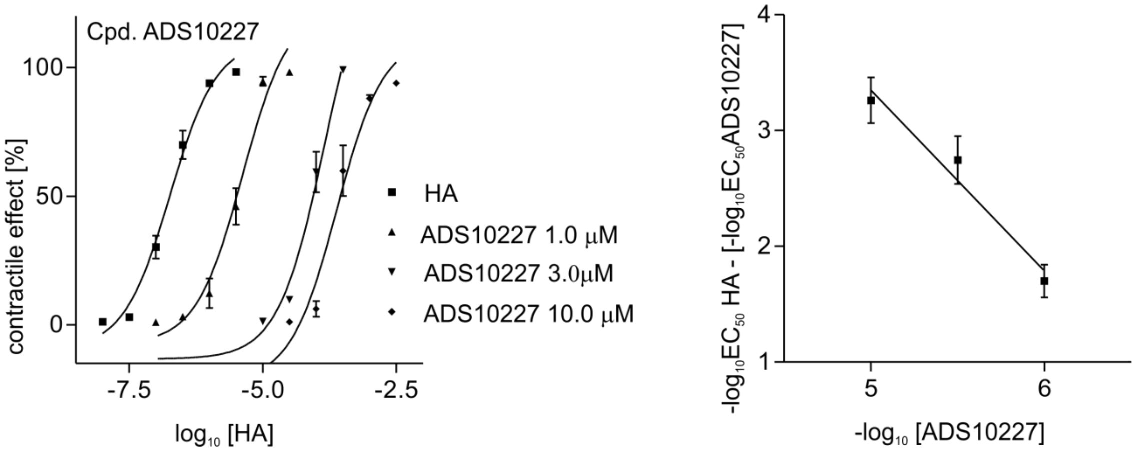

Figure S25. Contraction of guinea-pig ileum by histamine in the absence (■) and presence (▲,▼, ♦) of compound ADS10227

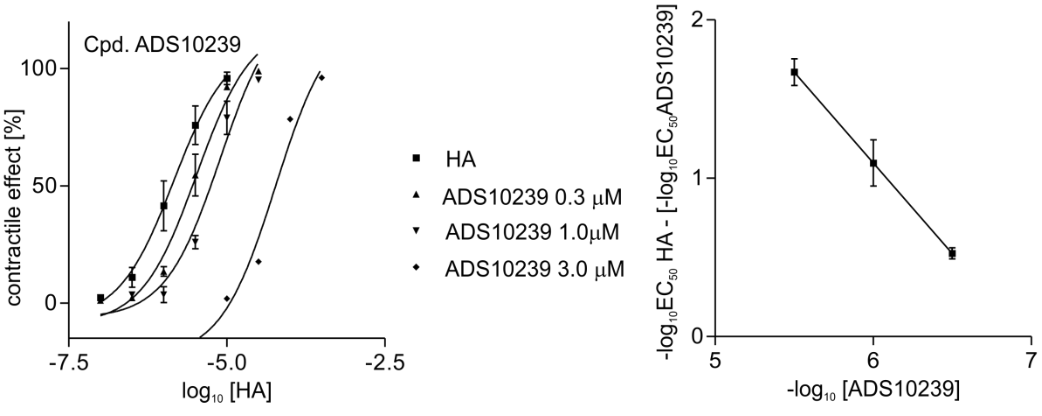

Figure S26. Contraction of guinea-pig ileum by histamine in the absence (■) and presence (▲,▼, ♦) of compound ADS10239

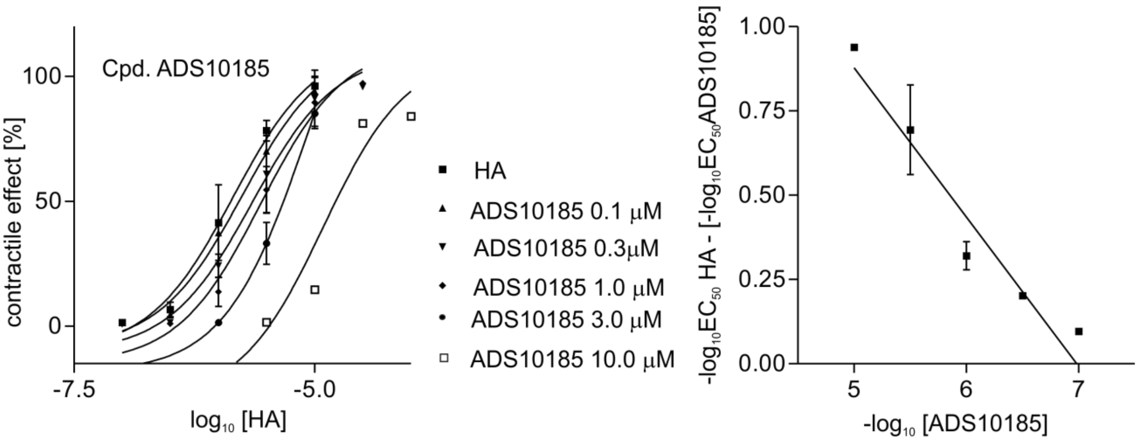

Figure S27. Contraction of guinea-pig ileum by histamine in the absence (■) and presence (▲,▼, ♦, ●, □) of compound ADS10185

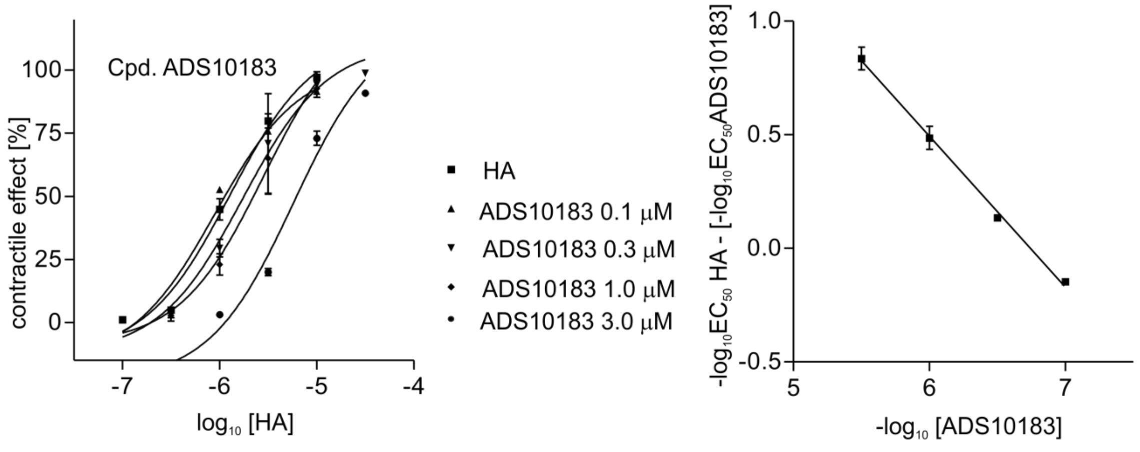

Figure S28. Contraction of guinea-pig ileum by histamine in the absence (■) and presence (▲,▼, ♦, ●) of compound ADS10183

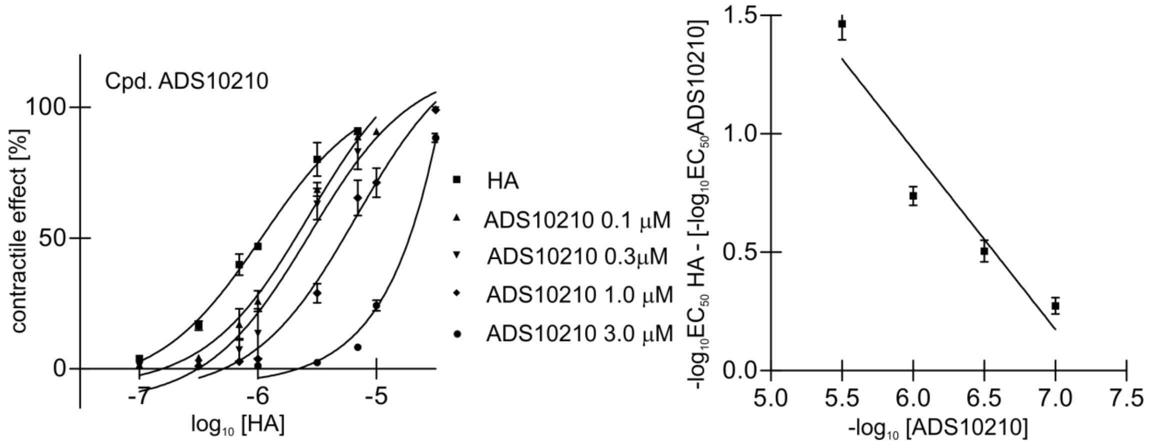

Figure S29. Contraction of guinea-pig ileum by histamine in the absence (■) and presence (▲,▼, ♦, ●) of compound ADS10210

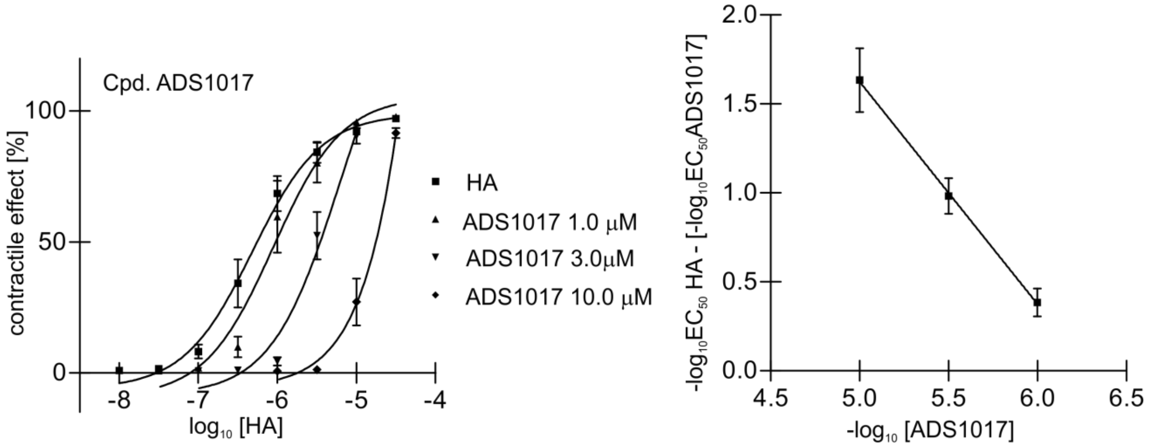

Figure S30. Contraction of guinea-pig ileum by histamine in the absence (■) and presence (▲,▼, ♦) of compound ADS1017

3.3 *Ex vivo* assay for histamine M_2R/M_3R receptor antagonists.

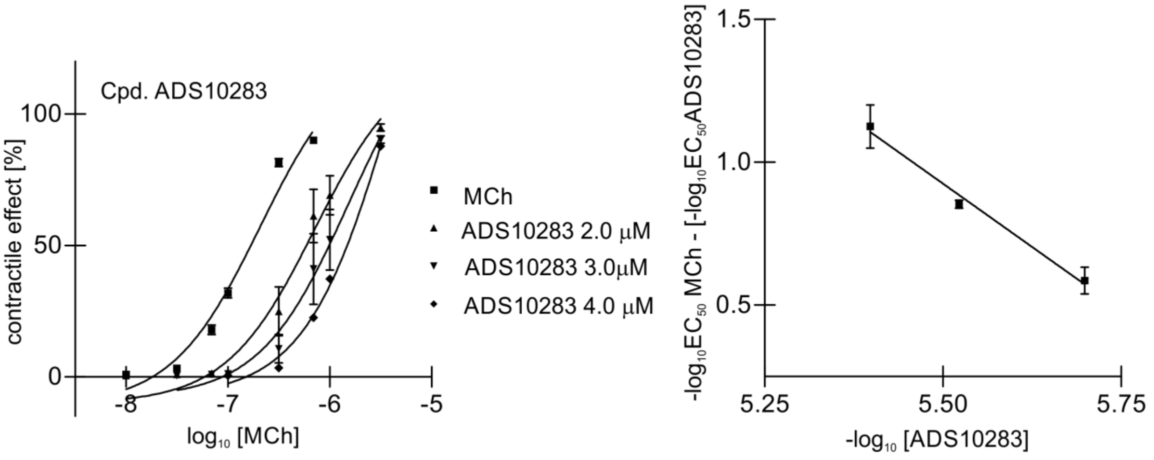

Figure S31. Contraction of guinea-pig ileum by methacholine in the absence (■) and presence (▲,▼, ♦) of compound ADS10283

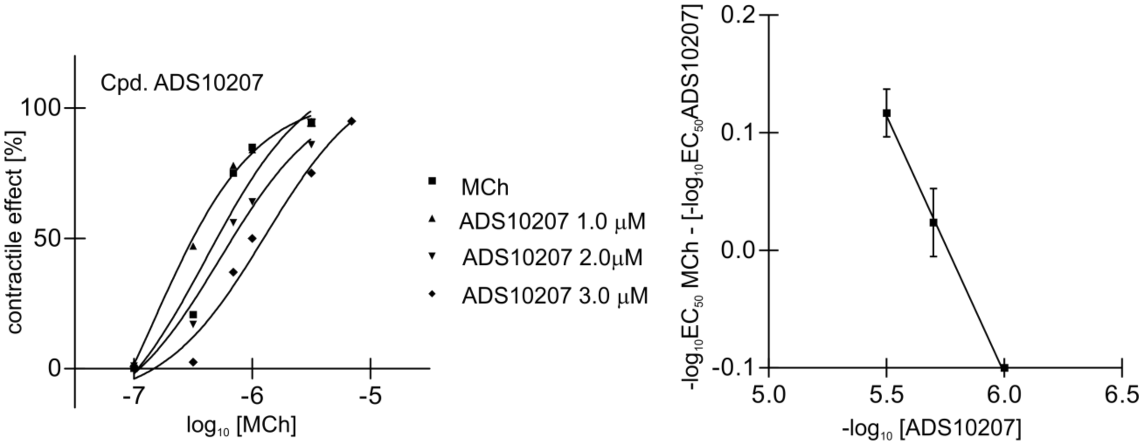

Figure S32. Contraction of guinea-pig ileum by methacholine in the absence (■) and presence (▲,▼, ♦) of compound ADS10207

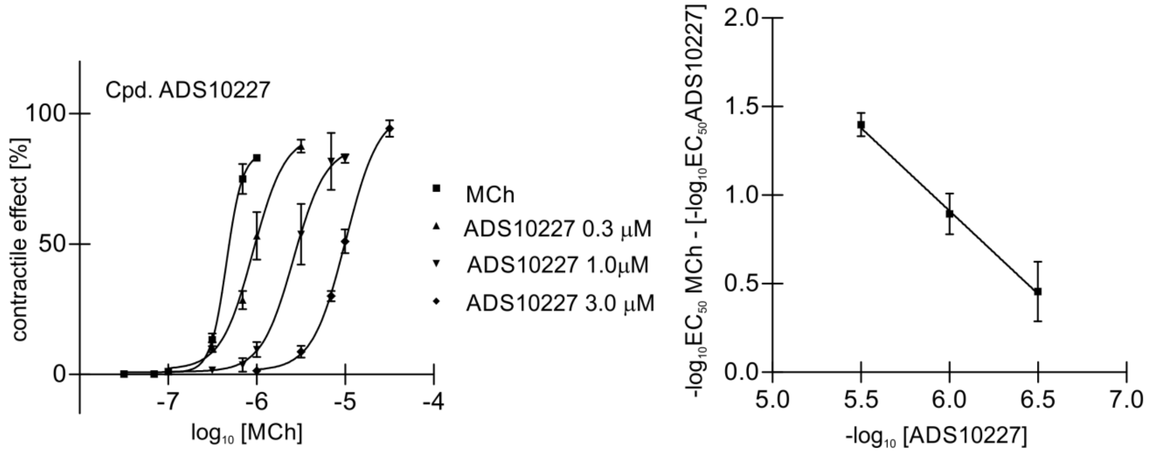

Figure S33. Contraction of guinea-pig ileum by methacholine in the absence (■) and presence (▲,▼, ♦) of compound ADS10227

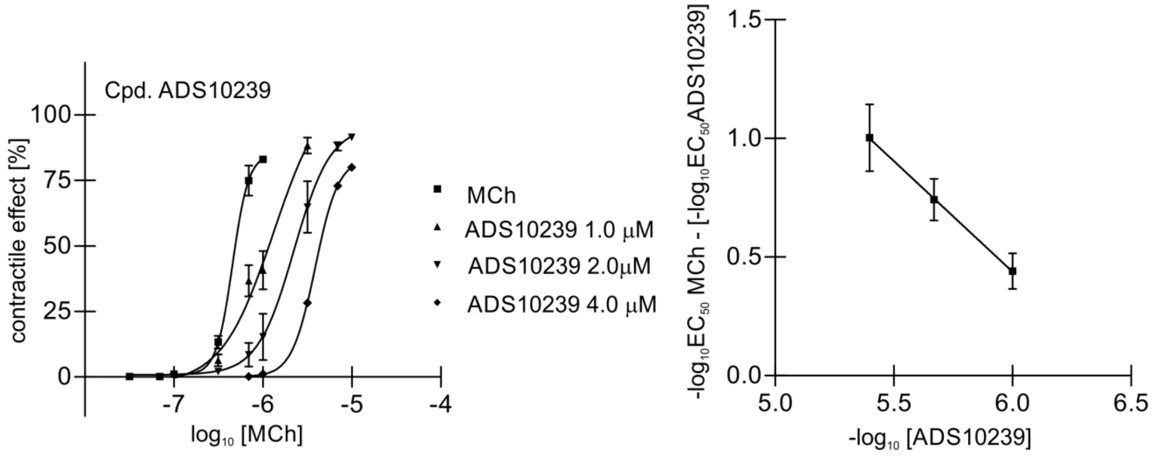

Figure S34. Contraction of guinea-pig ileum by methacholine in the absence (■) and presence (▲,▼, ♦) of compound ADS10239

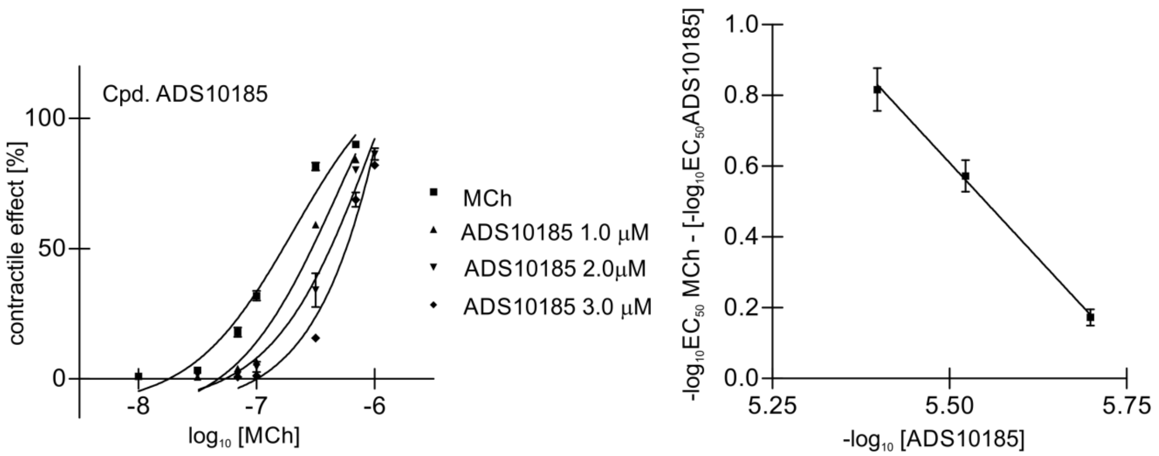

Figure S35. Contraction of guinea-pig ileum by methacholine in the absence (■) and presence (▲,▼, ♦) of compound ADS10185

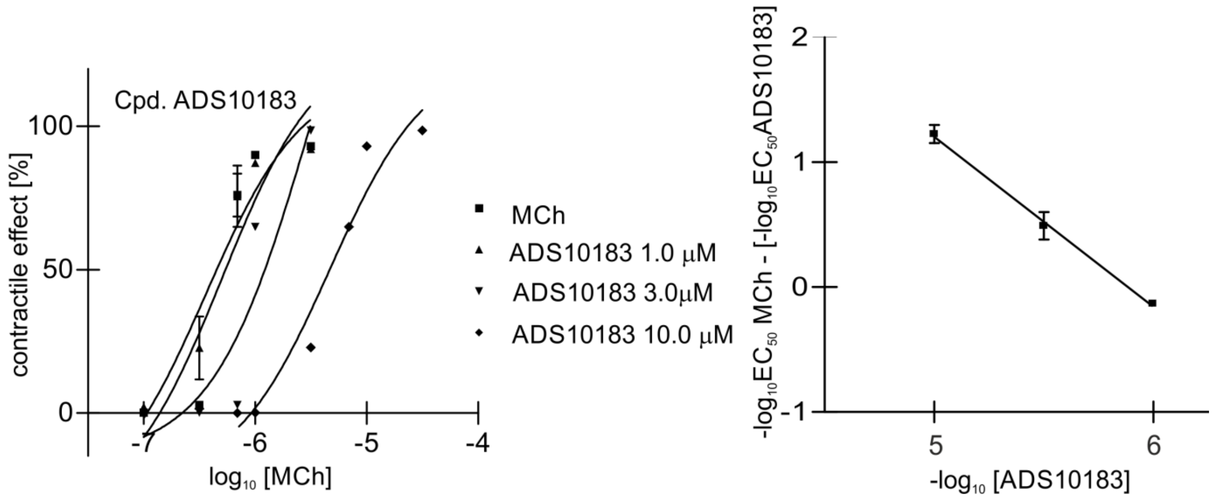

Figure S36. Contraction of guinea-pig ileum by methacholine in the absence (■) and presence (▲,▼, ♦) of compound ADS10183

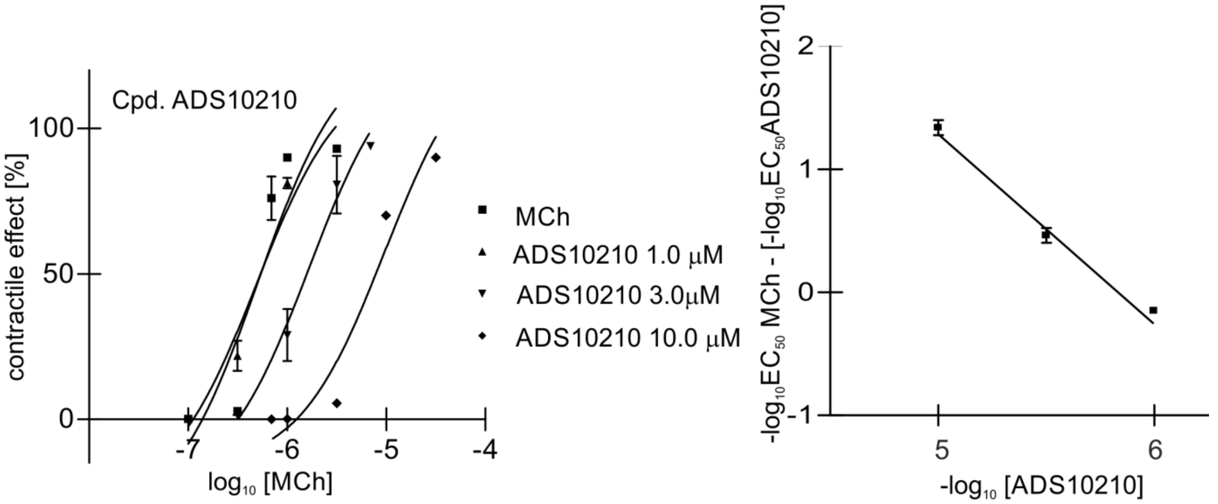

Figure S37. Contraction of guinea-pig ileum by methacholine in the absence (■) and presence (▲,▼, ♦) of compound ADS10210

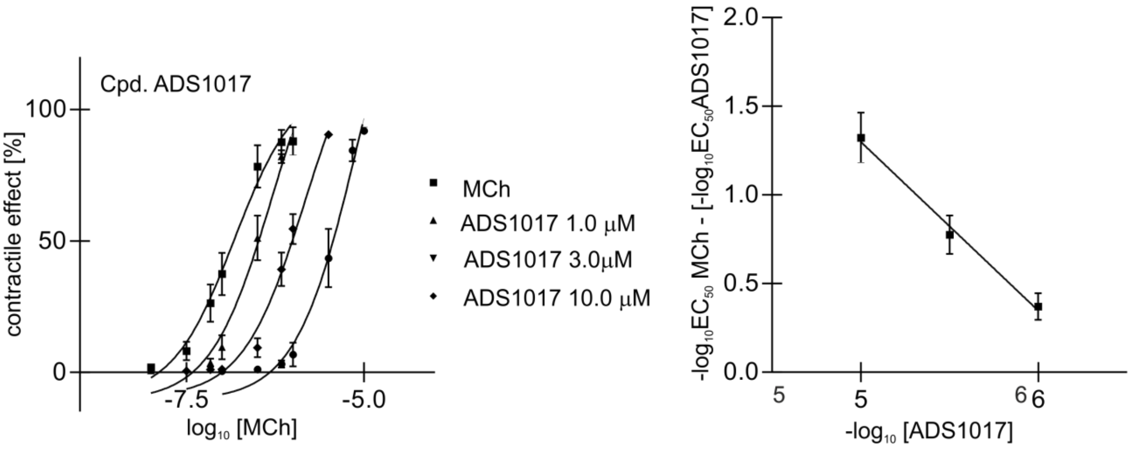

Figure S38. Contraction of guinea-pig ileum by methacholine in the absence (■) and presence (▲,▼, ♦) of compound ADS1017

3.4 Decrease of contractility in electrically-stimulated guinea pig ileum - determination of the -logEC50 coefficient.

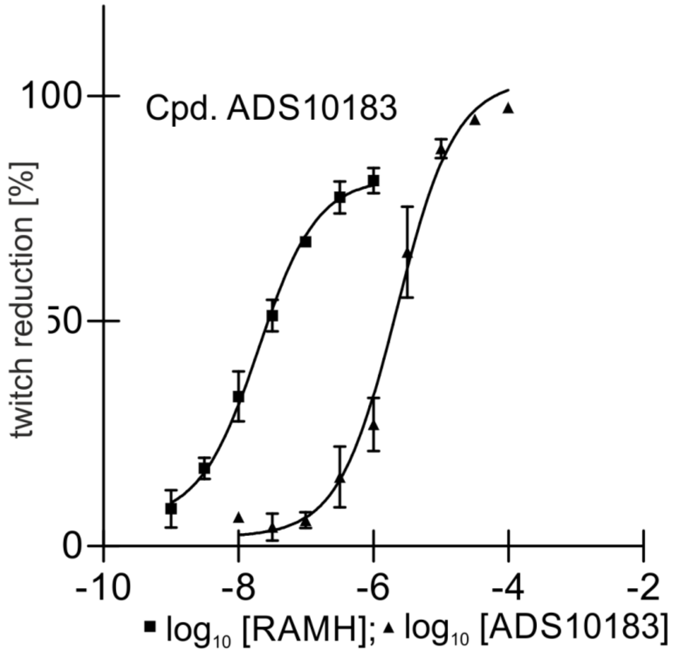

Figure S39. Twitch reduction of electrically evoked guinea-pig ileum by R-(α)-methylhistamine (RAMH) (■) and compound ADS10183 (▲)

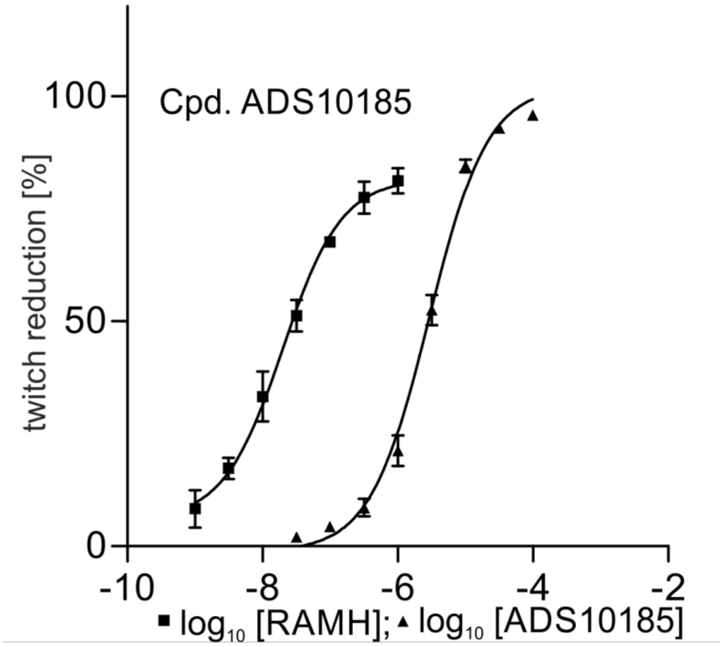

Figure S40. Twitch reduction of electrically evoked guinea-pig ileum by R-(α)-methylhistamine (RAMH) (■) and compound ADS10185 (▲)

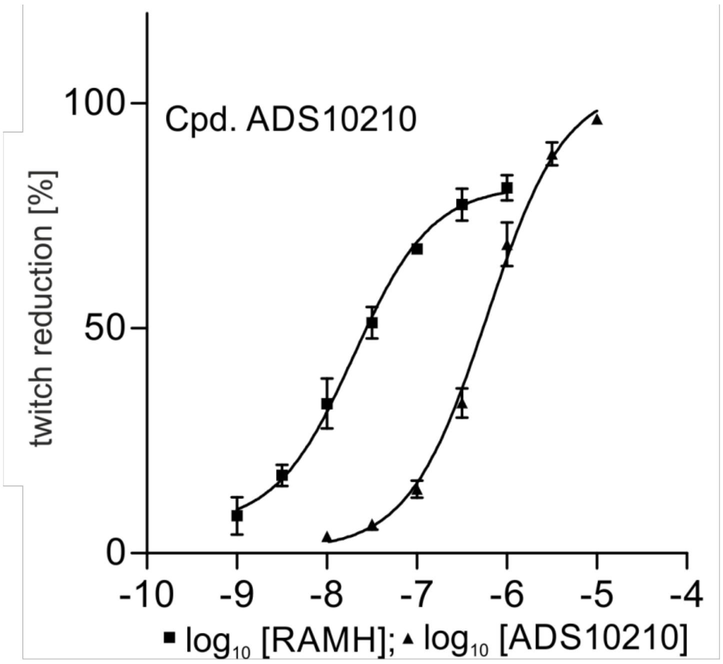

Figure S41. Twitch reduction of electrically evoked guinea-pig ileum by R-(α)-methylhistamine (RAMH) (■) and compound ADS10210 (▲)

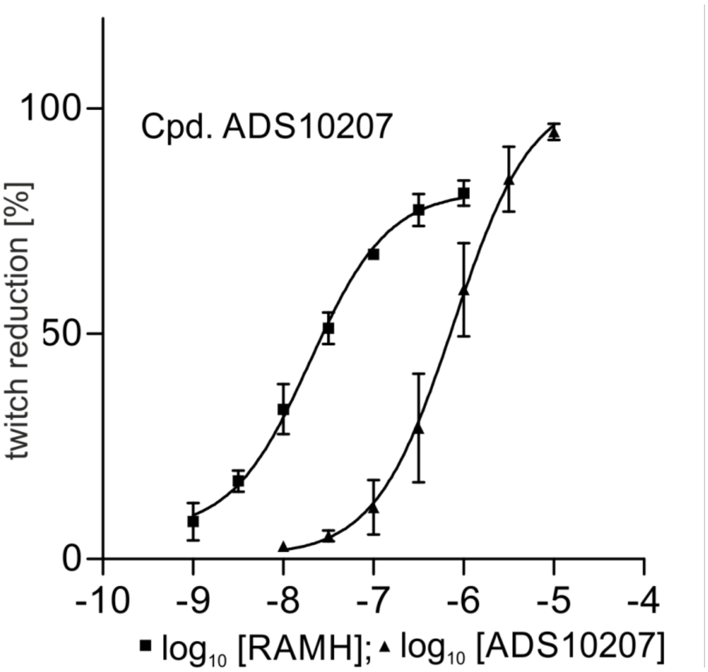

Figure S42. Twitch reduction of electrically evoked guinea-pig ileum by R-(α)-methylhistamine (RAMH) (■) and compound ADS10207 (▲)

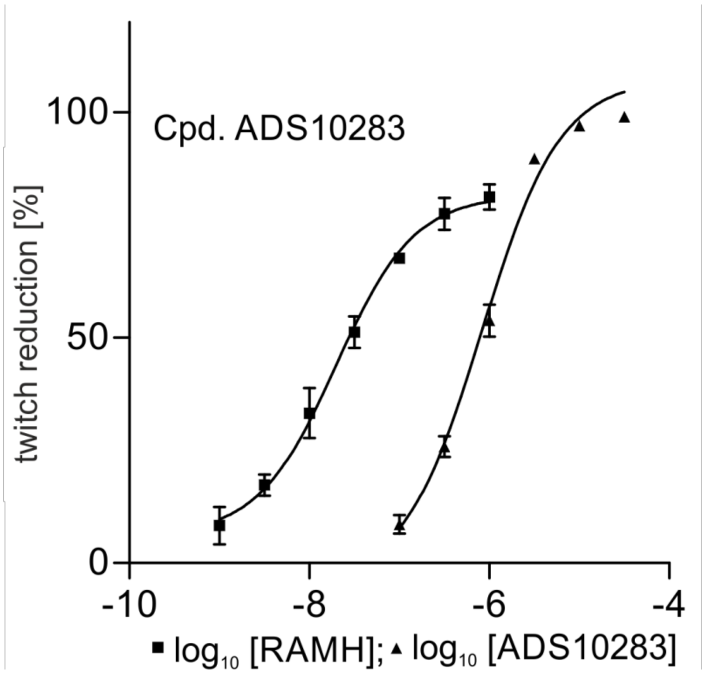

Figure S43. Twitch reduction of electrically evoked guinea-pig ileum by R-(α)-methylhistamine (RAMH) (■) and compound ADS10283 (▲)

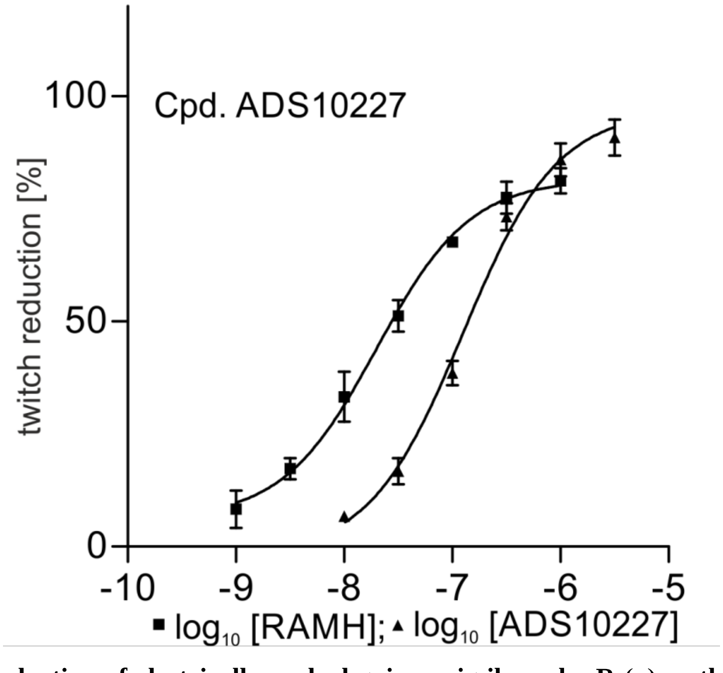

Figure S44. Twitch reduction of electrically evoked guinea-pig ileum by R-(α)-methylhistamine (RAMH) (■) and compound ADS10227 (▲)

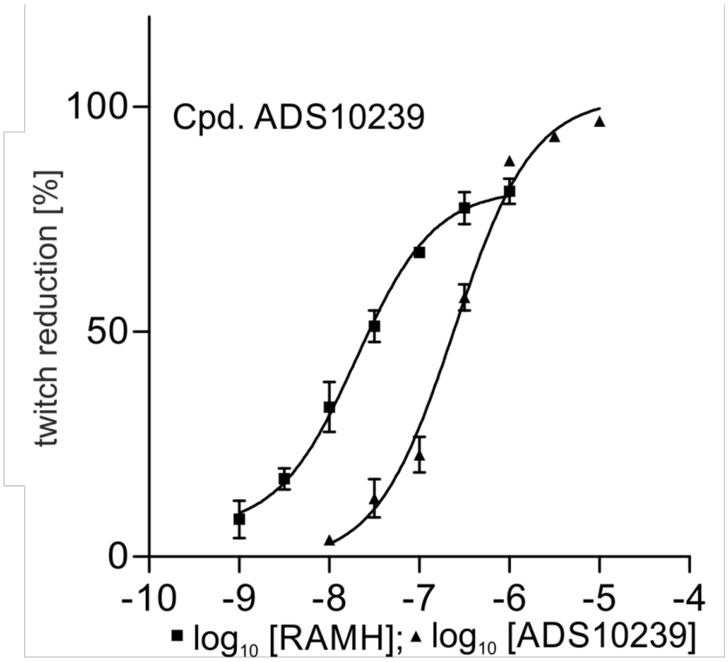

Figure S45. Twitch reduction of electrically evoked guinea-pig ileum by R-(α)-methylhistamine (RAMH) (■) and compound ADS10239 (▲)

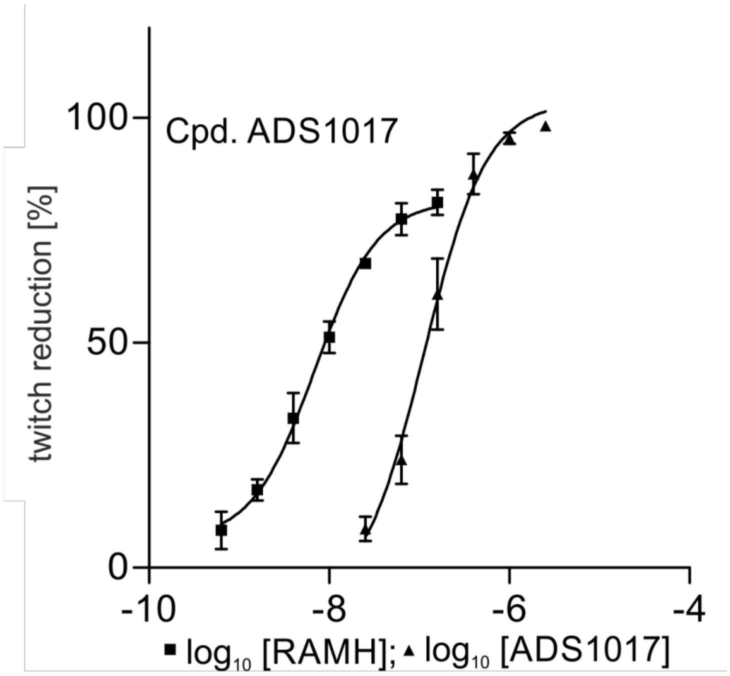

Figure S46. Twitch reduction of electrically evoked guinea-pig ileum by R-(α)-methylhistamine (RAMH) (■) and compound ADS1017 (▲)

3.5 hH_3R radioligand binding assay.

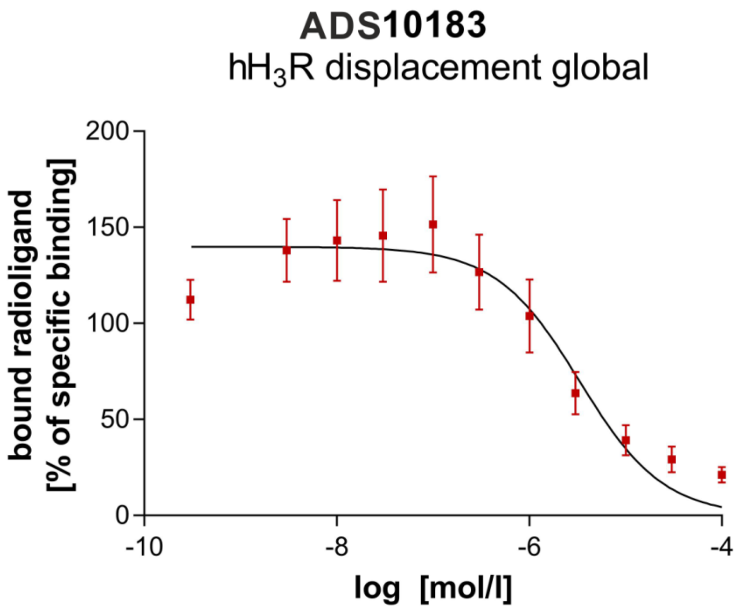

Figure S47. hH_3 competition binding curve of compound ADS10183.

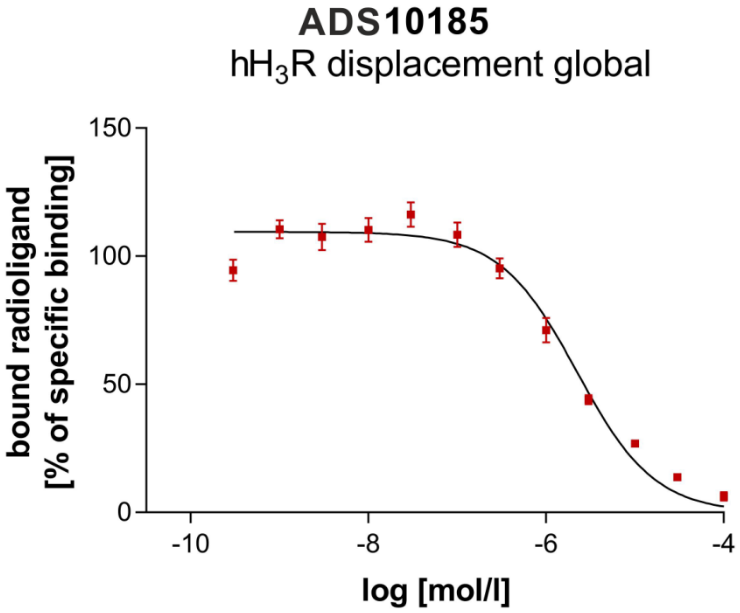

Figure S48. hH_3 competition binding curve of compound ADS10185.

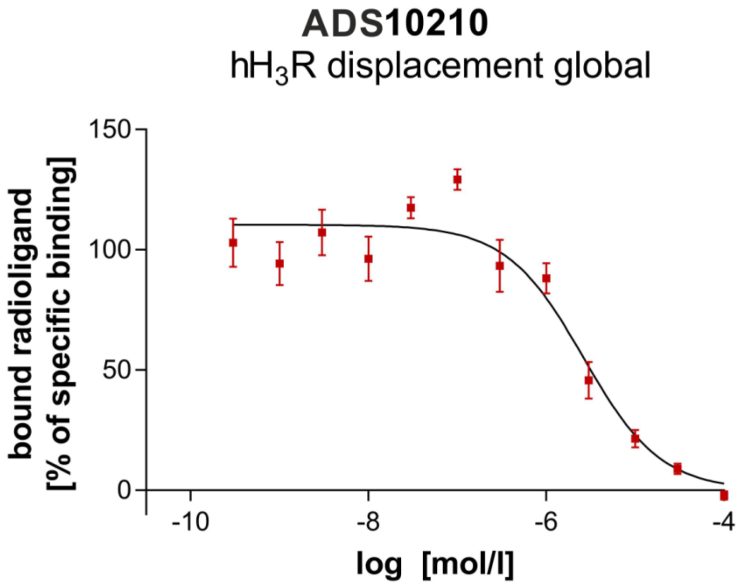

Figure S49. hH$_3$ competition binding curve of compound ADS10210.

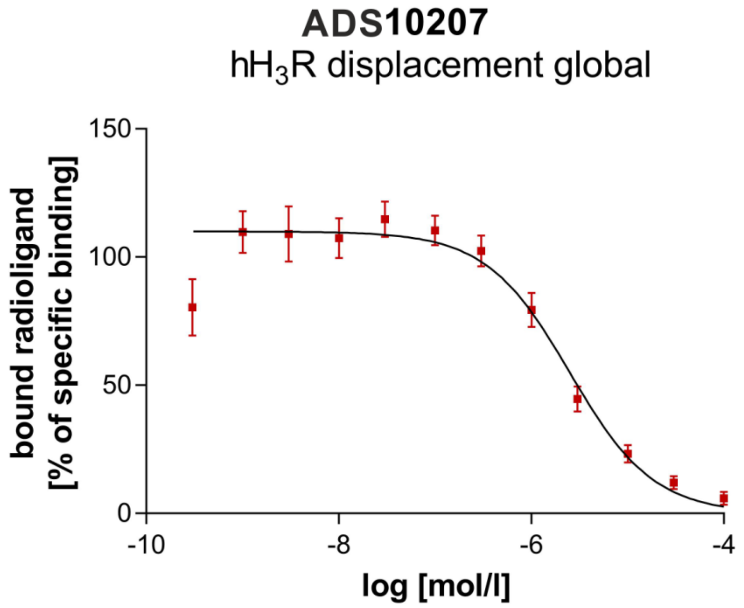

Figure S50. hH$_3$ competition binding curve of compound ADS10207.

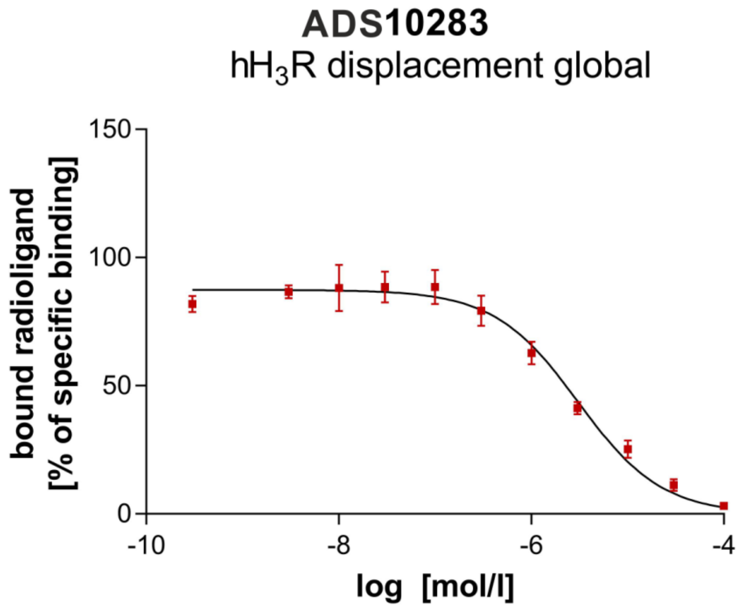

Figure S51. hH3 competition binding curve of compound ADS10283.

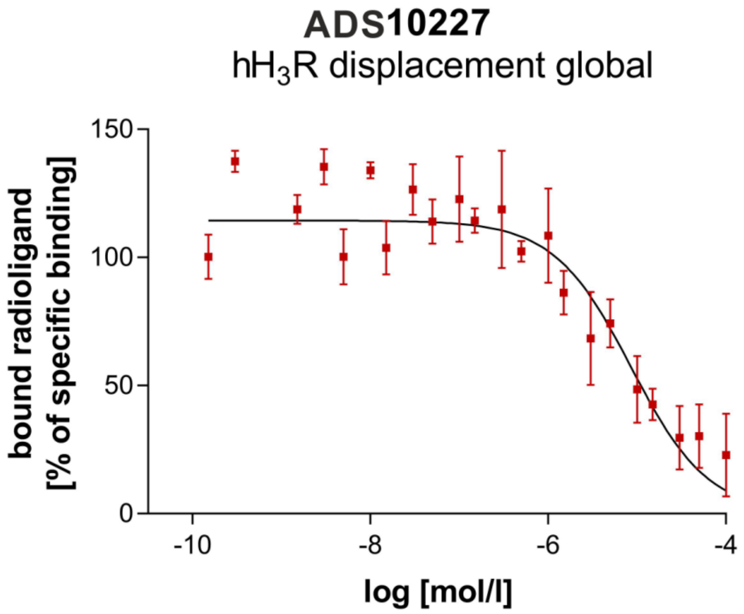

Figure S52. hH3 competition binding curve of compound ADS10227.

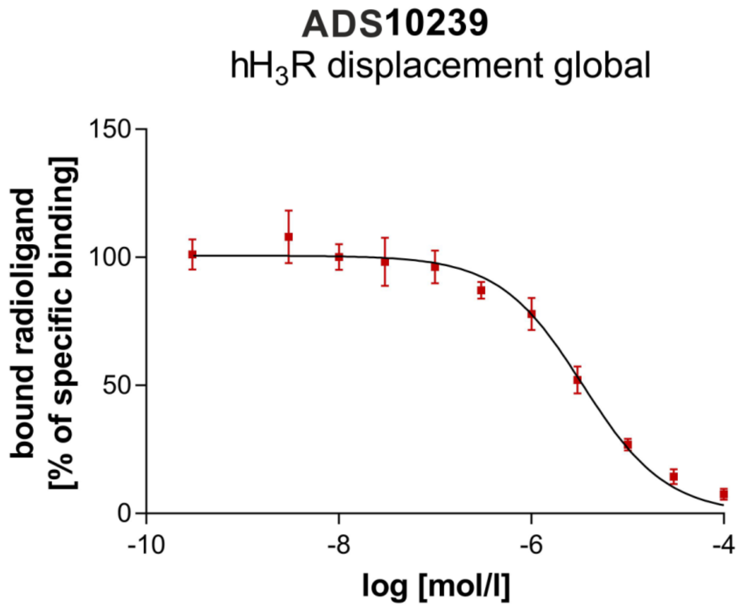

Figure S53. hH3 competition binding curve of compound ADS10239.

3.6 hM1R-hM5R radioligand binding assay.

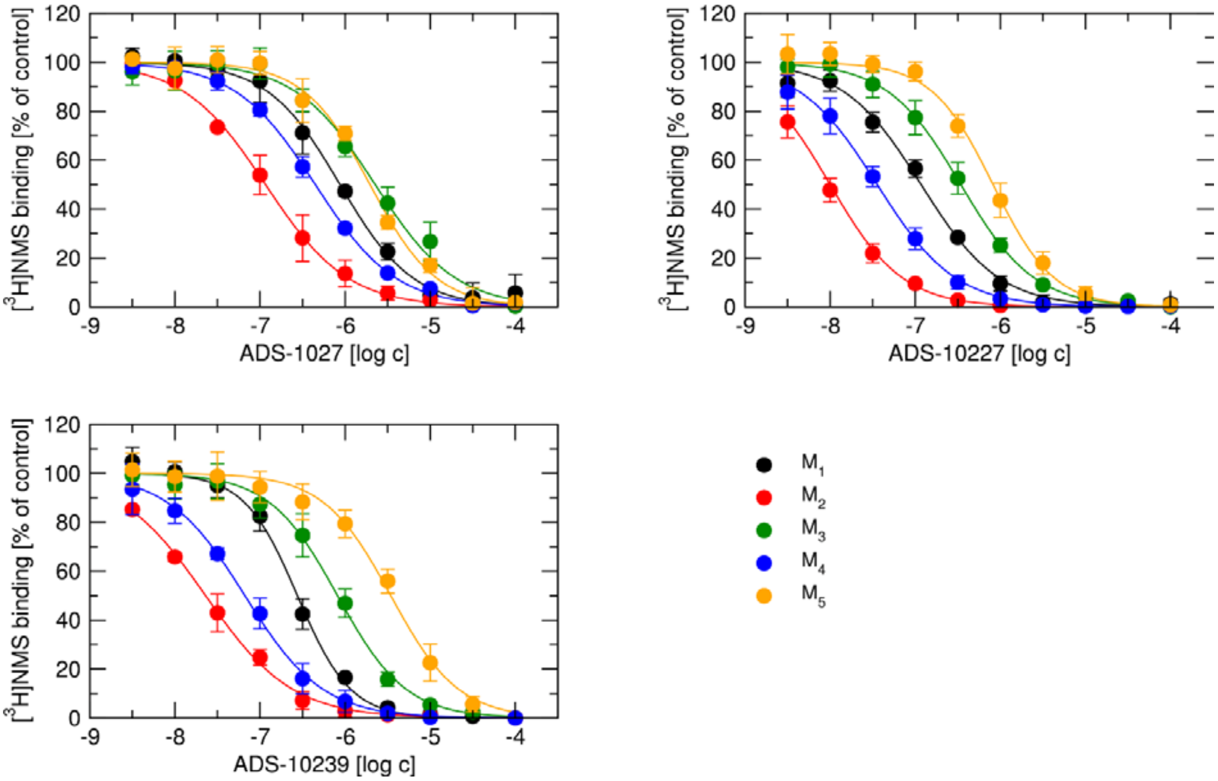

Figure S54 Radioligand binding. Competition binding between ADS-compounds in concentration indicated on x-axis and 100 pM [^{3}H]NMS at individual subtypes of muscarinic receptors indicated in legend.

3.7 Intracellular Ca^{2+} measurement.

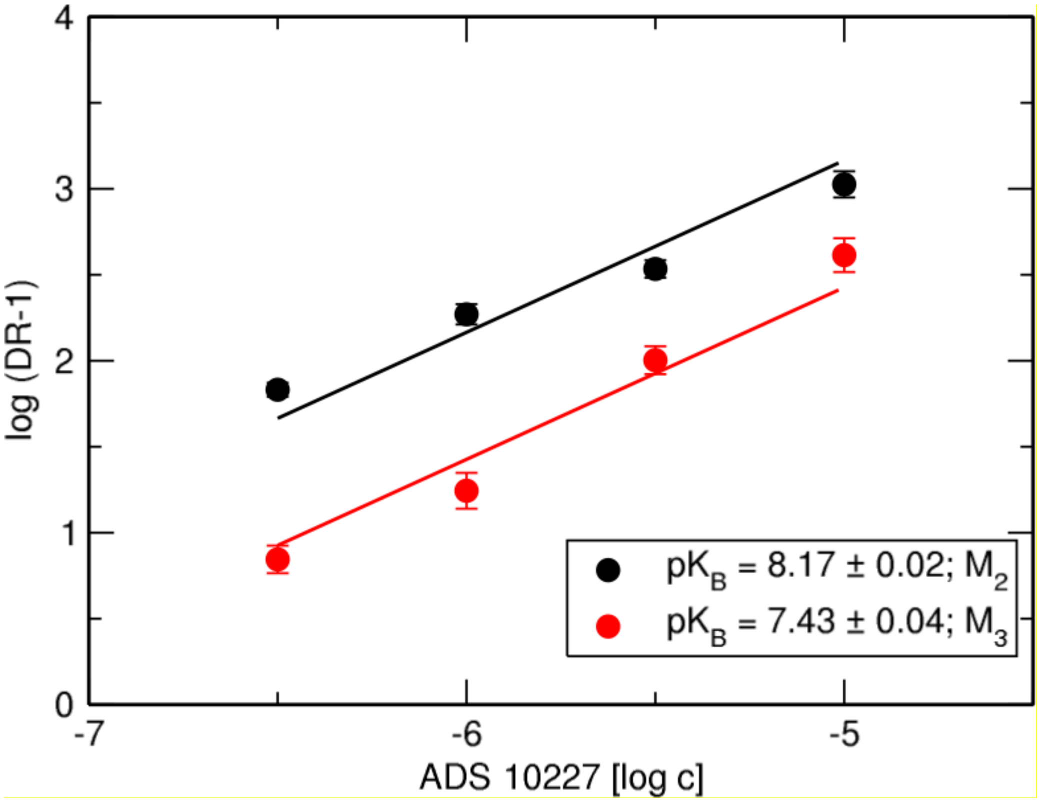

Figure S55. Schild plot of ADS10227 inhibition of intracellular Ca^{2+} mobilization upon activation of M_2R or M_3R by acetylcholine. Data are means ± sem from 4 experiments performed in quadruplicates.

4. *In silico* assay results.

4.1 Comparison of active sites of histamine and muscarinic receptors to H_3 receptor

Table S1. Comparison of active sites of histamine and muscarinic receptors to H_3 receptor with BLAST GPCRdb alignment for Class A GPCRs ligand binding pocket residue sets.

RECEPTOR	% IDENTICAL AMINO ACIDS	% SIMILAR AMINO ACIDS	ALIGNMENT SCORE
***H*H$_4$**	52	66	547
***H*M$_3$**	34	62	430
***H*M$_5$**	34	61	426
***H*M$_1$**	35	60	427
***H*M$_4$**	33	60	419
***H*H$_1$**	35	60	417
***H*M$_2$**	32	58	405
***H*H$_2$**	32	54	384

4.2 Change of amino acid conformation in the H_3 receptor binding site on the example of the homology model

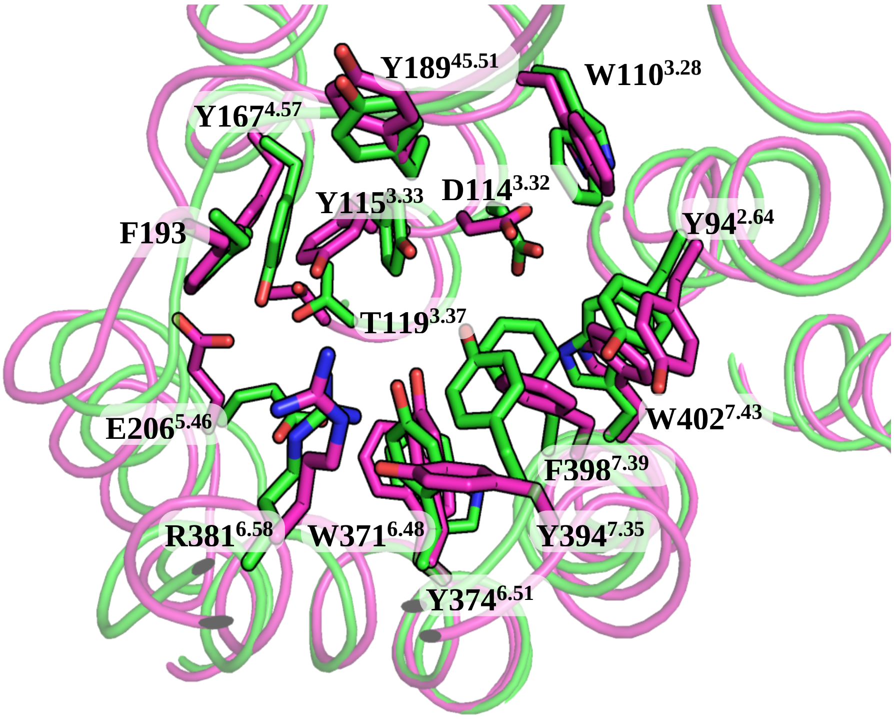

Figure S56. Change of amino acid conformation in the H_3 receptor binding site on the example of the homology model before optimization (green) and after optimization (magenta).

4.3 Two-dimensional map of interactions between the ADS1017 and ADS10227 ligands and H_3, M_2 and M_4 receptors

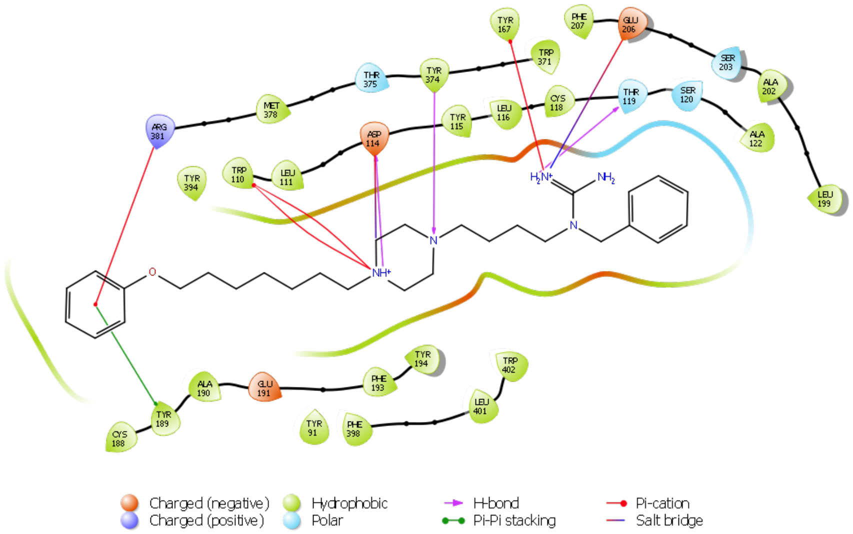

Figure S57. Two-dimensional map of interactions between the ADS1017 and H_3 receptor.

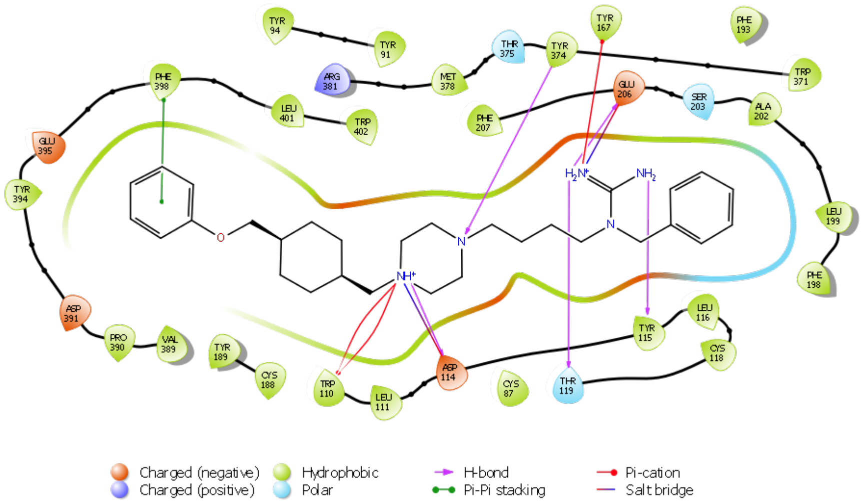

Figure S58. Two-dimensional map of interactions between the ADS10227 and H_3 receptor.

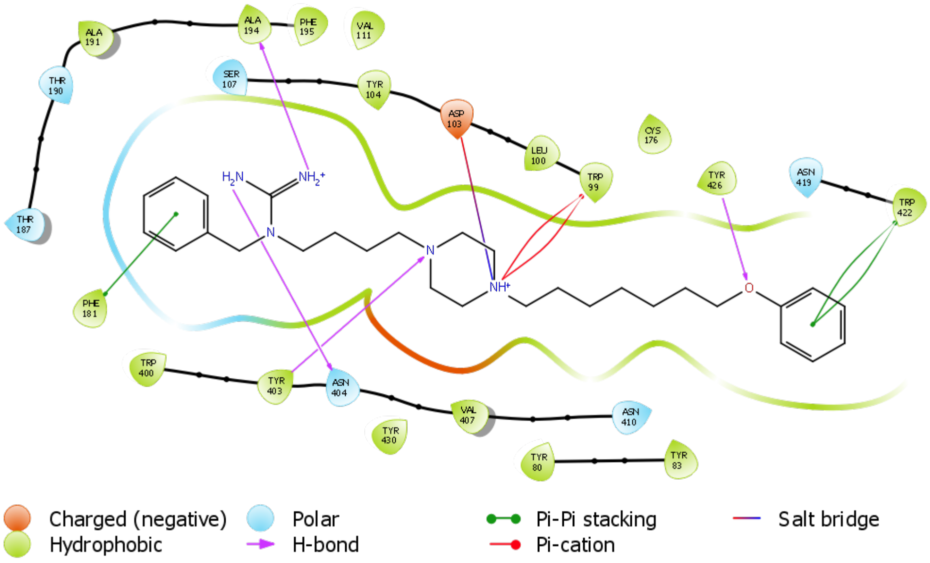

Figure S59. Two-dimensional map of interactions between the ADS1017 and M$_2$ receptor.

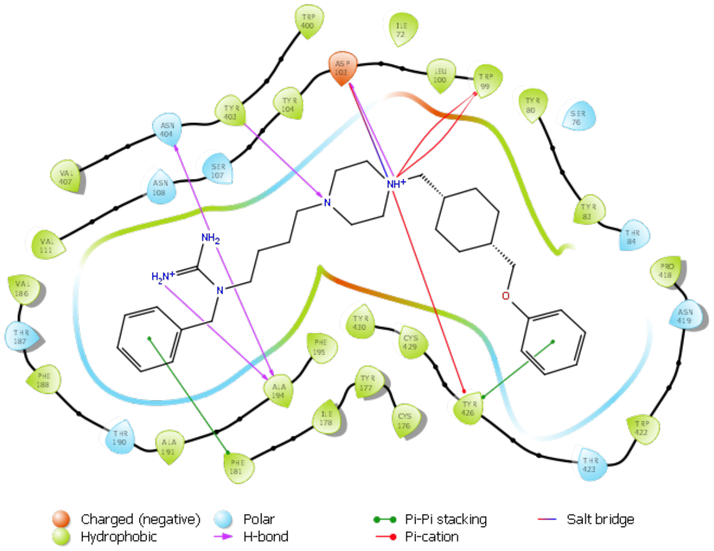

Figure S60. Two-dimensional map of interactions between the ADS10227 and M$_2$ receptor.

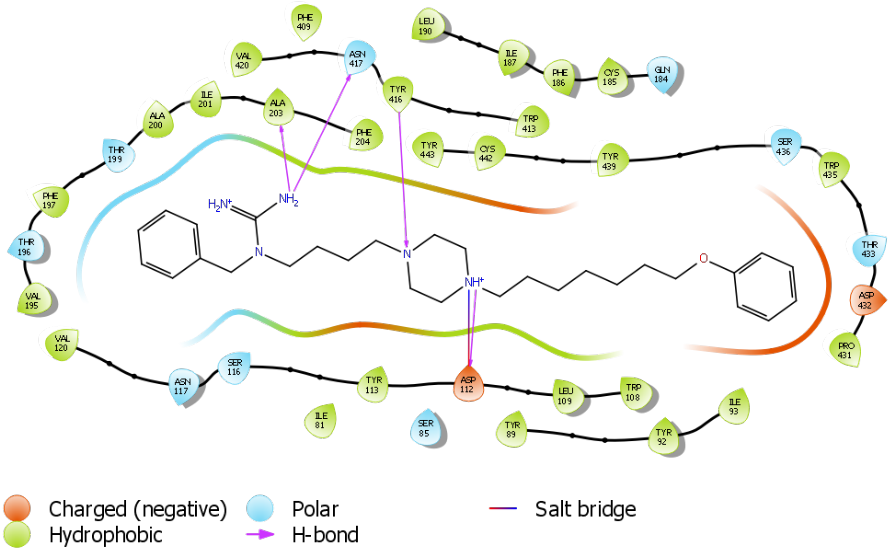

Figure S61. Two-dimensional map of interactions between the ADS1017 and M4 receptor.

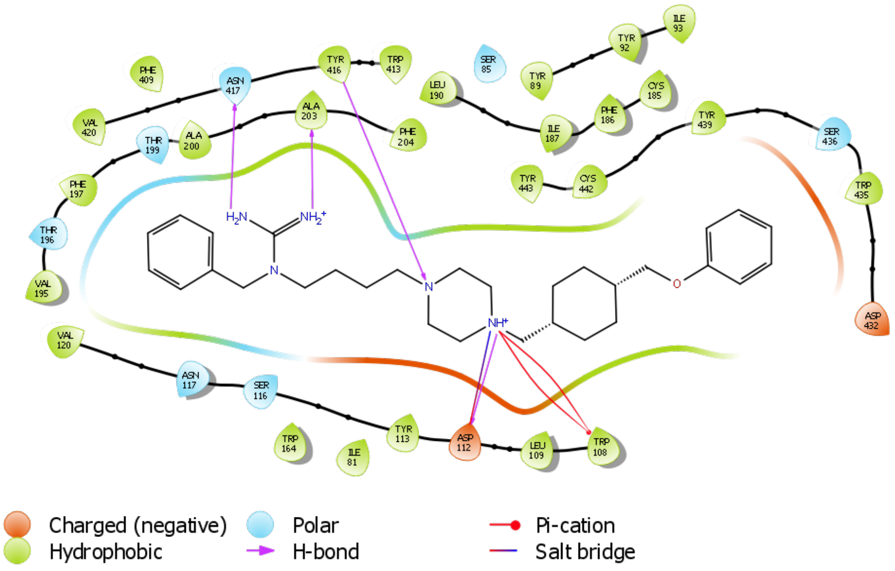

Figure S62. Two-dimensional map of interactions between the ADS10227 and H4 receptor.
